# Standardized tissue sampling guidelines for histopathological and molecular analyses of rainbow trout (*Oncorhynchus mykiss*) in ecotoxicological studies

**DOI:** 10.1371/journal.pone.0288542

**Published:** 2023-07-13

**Authors:** Sonja Fiedler, Hannah Schrader, Natalie Theobalt, Isabel Hofmann, Tobias Geiger, Daniela Arndt, Rüdiger Wanke, Julia Schwaiger, Andreas Blutke

**Affiliations:** 1 Institute of Veterinary Pathology at the Center for Clinical Veterinary Medicine, Ludwig-Maximilians-Universität München, Munich, Germany; 2 Unit 73 Aquatic Ecotoxicology, Microbial Ecology, Bavarian Environment Agency, Wielenbach, Germany; 3 Institute of Experimental Genetics, Helmholtz Zentrum Munich, Neuherberg, Germany; Benha University, EGYPT

## Abstract

In ecotoxicology, evaluation of toxicities and *no observed effect concentrations* (NOEC) of test compounds in experimental fish is commonly based on molecular-, biochemical- and analytical chemistry analyses of organ/tissue samples and the assessment of (histo-) pathological lesions. Standardization of organ/tissue sampling locations, sample numbers, and sample processing contributes to warrant the reproducibility and inter- and intra-study comparability of analysis results. The present article provides the first comprehensive tissue sampling guidelines specifically adapted to rainbow trout (*Oncorhynchus mykiss*) as a frequently used fish species in ecotoxicological studies. A broad spectrum of ~40 different organs and tissues is covered. Appropriate sampling locations, sample sizes and sample numbers for subsequent routine histopathological evaluation (all organs/tissue) and for molecular analyses (~30 organs/tissues) are described in detail and illustrated with schematic drawings and representative macroscopic and histological images. These field-proven sampling guidelines were developed based on the pertinent literature and practical experience in ecotoxicological fish studies. They are intended to serve as a standard reference for any routine ecotoxicological study using rainbow trout as a test system. A broad application of the featured tissue sampling procedures will help to improve the reproducibility of analyses and to reduce inter- and intra-study variability induced by sampling bias and (normal) inter-sample morphological variation, and will therefore provide a robust basis for reliable characterization of toxicity and NOEC identification of diverse test substances and aquatic pollutants.

## Introduction

In ecotoxicological exposure studies, the rainbow trout (*O*. *mykiss*) is a frequently used test system to examine toxic effects of diverse surface water pollutants [1–4]. With regard to environmental risk assessment, the experimental results and evaluated toxicological endpoints such as the *no observed effect concentration* (NOEC) are used for the assessment of the ecotoxicological potential of the given test substance and its classification as relevant for the (aquatic) environment, and may therefore provide the basis for the restriction of emissions/discharges or even the ban of hazardous substances [5,6]. Given the far-reaching consequences, the reproducibility of the analyses results, as well as the comparability of the results of different studies examining the same test substance, are essential. The applied mode of sampling and processing of organ/tissue specimens is an important factor affecting the unbiasedness, reproducibility, and comparability of analysis results that must be considered in the experimental design of any study. Here, application of standardized sampling guidelines can contribute to limit the intra- and inter-study variability by definition of organ/tissue-specific sampling locations, sample numbers, sample sizes, and, where applicable, their orientation(s), providing comparable organ/tissue specimens whose representativeness is warranted. Therefore, the use of standardized organ/tissue sampling protocols has become a generally accepted and expected standard in diverse life-sciences disciplines, such as toxicologic pathology or translational medicine, and standardized sampling guides have been established for several experimental animal species [7–14]. For fish of the size of the rainbow trout considered in the present guidelines, such standard sampling guides are missing to date, but they are urgently required in ecotoxicological studies. The present article provides the first comprehensive standardized sampling guidelines specifically adapted to rainbow trout of body weights between 300–2000 g, for the reproducible generation of tissue samples for histopathological examinations and a broad spectrum of molecular analyses.

### Experimental fish, ethics statement

For development, demonstration and validation of the methods shown in the present study, eight healthy rainbow trout of both sexes with body weights ranging from 300–2000 g were sacrificed. The use of the fish in this study was performed in accordance with the relevant legal regulations and with permission of the local authorities, and was approved by the institutional ethics committee of the Institute of Veterinary Pathology of the Ludwig-Maximilians-Universität Munich via verbal consent. The fish were obtained from the breeding facility of the Bavarian Environment Agency in Wielenbach, Germany. After initial health status check, fish were sacrificed either by stunning (concussion) and exsanguination or with tricaine methanesulphonate solution (500 mg/l, Tricaine Pharmaq^®^ 1000 mg/g, Pharmaq Ltd., United Kingdom) and subsequent brain destruction after circulatory arrest. In none of the examined fish, clinical, macroscopic, and histological examination revealed indications of disease or pathological alterations.

### Standardized sampling guidelines for rainbow trout organs and tissues

The present guidelines (**S1 File**) contain sampling protocols for ~40 different organs and tissues (**Table 1**) of rainbow trout of 300–2000 g body weight. For each featured organ/tissue, detailed sampling schedules are provided for the generation of standard formalin-fixed and paraffin-embedded (FF-PE) samples for light-microscopic histopathological evaluation, as well as for the generation of snap-frozen tissue specimens, suitable for a broad spectrum of downstream molecular and biochemical analyses, such as *e*.*g*., DNA-, RNA-, protein-, lipid-, and small molecule metabolite analysis as well as analytical chemistry. The samples are taken from defined anatomical locations, the recommended sample sizes and sample numbers, as well as sectioning directions and sample orientations (if applicable) are indicated. For convenience, hereinafter the broad entirety of downstream analyses such as molecular, biochemical and analytical chemistry analyses are collectively referred to as “molecular analyses”.

**Table 1:** List of rainbow trout organs and tissues covered by the present sampling guidelines for histopathological examination and molecular analyses.

Organ System	Organ/Tissue	Histo-pathological analyses^1^	Molecular analyses^2^	Chapter (Suppl. material)
**Respiratory system**	Gills	✓	✓	2.1
**Cardiovascular system**	Heart	✓	✓	2.2
	Blood vessels	✓	-	2.2
**Digestive system**	Tongue	✓	-	2.3.1
	Teeth	✓	-	2.3.1
	Liver and gallbladder	✓	✓	2.3.2
	Gastrointestinal tract^3^	✓	✓	2.3.3
	Pancreas (exocrine & endocrine)	✓	✓^4^	2.3.4
	Swim bladder	✓	✓	2.3.5
**Adipose tissue**	Visceral and subcutaneous adipose tissue	✓	✓^5^	2.4
**Hematopoietic and immune system**	Spleen	✓	✓	2.5
**Reproductive system**	Testes and ovaries	✓	✓	2.6
**Urinary and hematopoietic system**	Kidneys (head- and trunk kidney)	✓	✓	2.7
**Central nervous system**	Brain	✓	✓^6^	2.8
	Spinal cord	✓	✓	2.8
**Integument**	Scaled and non-scaled skin	✓	✓	2.9
**Locomotor system**	White and red skeletal musculature	✓	✓	2.10.1
	Bone	✓	✓^7^	2.10.2
	Cartilage	✓	-	2.10.2
	Fins	✓	✓	2.10.3
**Pseudobranchs**	Pseudobranchs	✓	✓	2.11
**Sensory system**	Olfactory rosettes	✓	✓	2.12.1
	Inner ears	✓	-	2.12.2
	Lateral line canal	✓	-	2.12.3
	Eyes	✓	✓^8^	2.12.4
**Endocrine system**	Pituitary gland	✓	✓^9^	2.13.1
	Endocrine pancreas	✓	✓^9^	2.13.2
	Thyroid gland	✓	-	2.13.3
	Inter- and suprarenal tissue	✓	-	2.13.4
	Corpuscles of Stannius	✓	✓^9^	2.13.5
	Pineal gland (epiphysis)	✓	✓^9^	2.13.6
	Urophysis	✓	-	2.13.7
	Ultimobranchial gland	✓	-	2.13.8

^1^Standard light-microscopic histopathological examinations of sections of FF-PE tissue samples. ^2^Snap-frozen samples suitable for different downstream molecular, biochemical or analytical chemistry analyses. ^3^Gastrointestinal tract samples include: Esophagus, stomach, pyloric ceca, mid intestine, and posterior intestine. ^4^The Brockmann body is sampled for molecular analyses of the (exocrine and endocrine) pancreas. ^5^The specimen for molecular analyses of adipose tissue is generated from the visceral adipose tissue (VAT). ^6^Three brain samples for molecular analyses are generated: Telencephalon, diencephalon and mesencephalon, and rhombencephalon. ^7^The vertebral centrum is generated as bone tissue specimen for molecular analyses. ^8^Vitreous humour, cornea, lens, and retina are generated as specimens for molecular eye analyses. ^9^If the study design requires molecular analyses, it is recommended to sample the corresponding organ/tissue in toto.

A brief introduction summarizes the general necropsy- and tissue sample processing methods and explains the pictograms and symbols used to illustrate the sampling locations and sample types for the different downstream analyses, the sectioning directions and sample orientations, as well as subsequent sample processing steps and storage conditions.

**Fig 1:**
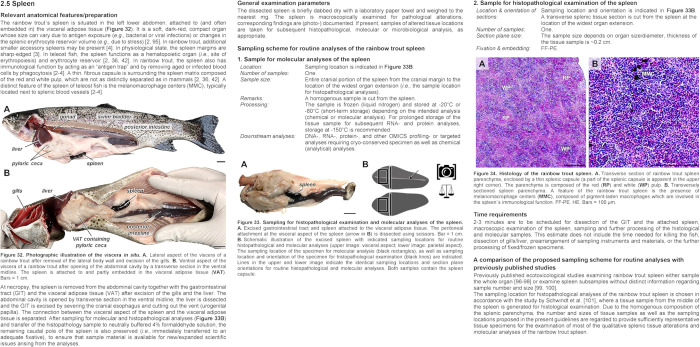
Illustration of the spleen sampling protocol as a representative example of the rainbow trout sampling guidelines. Each sampling protocol contains a brief summary of the relevant trout-specific anatomical, functional and histological features and recommendations for dissection/preparation (**Relevant anatomical features/preparations**), a brief instruction on the general examination procedure (*e*.*g*., weighing, macroscopic examination) (**General examination parameters**) as well as sample locations/numbers/sizes and subsequent sample processing steps and storage conditions for histopathological and molecular analyses (**Sampling scheme for routine analyses of the rainbow trout spleen**). For histopathological analyses, the protocols additionally contain recommendations on sample section plane orientations. The protocols further include an estimate of time required for sampling (**Time requirements**) and a concluding comparison of the proposed sampling scheme with previously published ecotoxicological studies using (rainbow) trout (**A comparison of the proposed sampling scheme for routine analyses with previously published studies**). Each protocol is illustrated with comprehensible schematic drawings and representative macroscopic and histological images.

The sampling protocols for the individual organs/tissues (see **Fig 1** for a representative example) each contain particular information about the following subjects:

- Relevant trout-specific anatomical, functional, and histological organ/tissue features.
- Practical recommendations for the preparation/dissection of the respective organ/tissue.
- Recommended sampling locations, sample numbers, and individual sample sizes for histopathological and molecular analyses.
- Recommended section plane orientations of samples for histopathological analyses.
- Specific tissue processing methods for subsequent histopathological and molecular analyses.
- Comprehensible schematic illustrations and representative histological images.
- Estimates of the time requirement for sample collection.
- A comparison of the proposed sampling scheme with previously published ecotoxicological studies using (rainbow) trout.

## Discussion

In ecotoxicological exposure studies, test item related findings indicating toxicity/adverse effects on organ/tissue level (with a dose/effect relationship) are commonly assessed by a variety of analyses of organ- and tissue samples, taken from susceptible species exposed to defined concentrations of the test compound over a specific period of time. The wide range of analyses includes *e*.*g*., clinical-chemical analyses, hematological investigations, molecular and biochemical analyses as well as histopathological evaluation [2,15,16]. With regard to environmental risk assessment, the experimental results serve for the assessment of the ecotoxicological potential of the given test substance and its relevance for the (aquatic) environment. Toxicological end points based on the experimental results, such as the NOEC of the test substance, are an important part of the scientific basis for the definition of the *predicted no effect concentration* (PNEC) [2,5,16,17]. The PNEC has an important and legally anchored role in the environmental risk assessment and authorization of anthropogenic substances, such as chemicals or pharmaceuticals [18–21] as well as in the restriction of environmentally relevant priority substances in the water bodies (mainly surface waters) of the European Union [6,22] and may therefore provide the basis for emission/discharge limitations or even the ban of hazardous substances [5,6]. Usually, ecotoxicity data derived from standard biotests on aquatic organisms (including fish) (*e*.*g*., OECD test guidelines) are used for the environmental risk/hazard assessment of a chemical test substance [1,20–23]. The test item related effect data collected in these biotests (*e*.*g*., mortality or reproductive abnormality) are not always sufficiently sensitive to reliably determine the potential adverse contaminant effects on fish health. Non-standard biotests, such as histopathological, molecular or biochemical studies, have proven to be sensitive tools for detecting (sublethal) contaminant effects in fish and therefore can significantly contribute to the environmental risk assessment of test items [2,15,16,23–25].

A review of previously published ecotoxicology studies on various test substances using rainbow trout (RBT) reveals that tissue sampling locations and examined sample numbers are, if mentioned at all, generally considerably variable (**S1 File**). In parallel, it becomes evident that the methods and results of different studies examining identical test compounds are occasionally remarkably divergent, as there is no valid guideline to use. Prime examples are the NOECs determined in different studies analyzing the (histo-) morphological effects of the exposure of RBT to diclofenac (an analgesic which is regularly detectable in surface waters), which differ over multiple orders of magnitude from 0.1 μg/l to 320 μg/l [17,26–30].

Insufficient reproducibility and comparability of analytical results in ecotoxicology studies may result from underreporting of a study as well as from various confounding variables, such as different exposure concentrations and -systems, different ages, sexes or genetic background of the examined fish, or differing technical procedures applied in necropsy and sample processing [17,23,30]. Additionally, histopathological diagnoses, and particularly the use of ordinally scaled grading systems for assessment of the severity of histopathological lesions (such as +, ++, +++), may also considerably vary between different observers and studies due to the subjective nature of histopathological interpretation and the sampling- and observational bias [17,31–34]. This is especially relevant, if only subtle alterations are present, which do not manifest in all individual fish of a cohort (*e*.*g*., due to exposure to low concentrations of a test substance). In this context, the general experience in life science disciplines examining test animals (mammalian and fish species) is, that standardization of the locations, as well as numbers, sizes, and orientations of samples generated from distinct organs/tissues for routine histopathological and molecular analyses is useful to limit sampling bias, to streamline the experimental study designs, and thus to strengthen the reliability and comparability of the analysis results [7,8,10,29–31,33]. Therefore, standardized sampling guides have been established for different experimental animal species, including mice, rats, pigs, dogs, monkeys and also small fish species such as fathead minnow (*Pimephales promelas*), zebrafish (*Danio rerio*) or Japanese medaka (*Oryzias latipes*) [7–14]. For data collected in non-standard biotests to be considered in regulatory risk assessment and the derivation of safe concentrations such as the PNEC, ecotoxicological studies must meet some scientific quality criteria for the collection of reliable and reproducible data, and all important information regarding the study design, methodology, test organisms *etc*. should be reported [23,35,36]. Standardized sampling and sample processing protocols help to improve the reporting of ecotoxicological studies and aid to ensure that test results can be reproduced in other studies. This is especially valid for histopathological data, whose interpretation, in addition to the researcher`s expertise, *inter alia* depends on the sampling strategy, the sample processing or the chosen section plane. Next to measures like blinded evaluation, the formation of a Pathology Working Group or the use of quantitative morphological analysis methods, therefore the quality, accuracy and reproducibility of histopathological data strongly benefit from standardized, detailed and user-friendly sampling and sample processing protocols, addressing the organ-/tissue-specific properties (*e*.*g*., tissue fragility or tendency to autolysis) and (histo-) morphology [31–33].

The sampling guidelines presented here are the first standardized tissue sampling guidelines for routine ecotoxicology studies in RBT. They were specifically designed for RBT of 300–2000 g body weight, which are frequently used in routine ecotoxicological exposure studies and whose size allows the simultaneous generation of samples of multiple organs and tissues. The featured protocols are based on the pertinent literature (specified for each organ/tissue in **S1 File**), as well as on own investigations and practical experience in ecotoxicological studies [16,28,29,37–39]. The guidelines aim to provide a standard reference for the reproducible sampling of appropriate RBT organ/tissue specimens for standard histopathological examinations and molecular analyses. To warrant comprehensible, fast, and reproducible sampling procedures, the sampling protocols schedule the collection of a fixed number of samples with uniform sizes, taken from precisely determined locations and in predefined orientations (if applicable). This sampling regime is considered adequate for the demands of typical ecotoxicological studies, as it facilitates screening of a broad set of different organs/tissues for identification of qualitative histopathological changes and of organ/tissue-specific alterations of *e*.*g*., biochemical- or molecular analysis parameters, using robust, standard analysis methods with acceptable sampling efforts. Depending on the objectives and the experimental design of a given study, the number of organs and tissues to be sampled can individually be adjusted. In studies scheduling advanced analyses requiring special sampling regimes (*e*.*g*., systematic uniform random sampling) or sample processing procedures (*e*.*g*., for electron microscopic analyses), however, additional sampling efforts and different tissue sample processing methods may be necessary. Also, if macroscopically evident lesions are present, additional samples should be taken from the altered sites for histopathology and microbiological/parasitological/molecular *etc*. analyses, as appropriate.

Generally, the appropriate sampling locations and the adequate numbers of samples depend on a variety of different factors. These factors include the size of the tissue samples and scheduled subsequent analysis methods, as well as the composition, heterogeneity and size of the respective organ/tissue, the pattern and extent of pathological lesions, particular susceptibilities of specific organ sites to development of pathological alterations, and biological/individual variances [7]. The proposed sampling locations, sample sizes and sample numbers indicated in the present sampling guidelines were chosen to effectively generate samples that are likely representative for the entire organ/tissue they were taken from, without redundant, time- and work-consuming oversampling. The indicated sample sizes for molecular and histopathological analyses provide sufficient sample volumes/section areas, ensure a fast snap-freezing- or fixation process, and are adapted to the size of commonly used test tubes or embedding cassettes, respectively.

## Conclusions

A broad application of consistent and carefully considered organ/tissue sampling protocols will enhance the quality, significance, and reproducibility of ecotoxicology studies using rainbow trout as test systems. The sampling guidelines presented here provide a robust basis for the generation of standardized rainbow trout tissue samples for routine histopathological and molecular analyses, which will contribute to the validity of inter- and intra-study comparisons of ecotoxicology studies. Due to the provided step-by-step protocol allowing the sampling of all ecotoxicologically relevant organs and tissues from a single rainbow trout, also unnecessary repetition of experiments might be avoided, thus limiting the number of fish sacrificed in ecotoxicological exposure studies.

## Supporting information

S1 File Standardized sampling guidelines for rainbow trout organs and tissues. (PDF)

## References

[1] OECD. Fish Toxicity Testing Framework—OECD Series on Testing and Assessment, No. 177. Paris: OECD Publishing; 2014.

[2] van der Oost R, Beyer J, Vermeulen NP. Fish bioaccumulation and biomarkers in environmental risk assessment: a review. Environmental Toxicology and Pharmacology. 2003;13(2): 57–149. doi: 10.1016/s1382-6689(02)00126-6 21782649

[3] Thorgaard GH, Bailey GS, Williams D, Buhler DR, Kaattari SL, Ristow SS, et al. Status and opportunities for genomics research with rainbow trout. Comparative Biochemistry and Physiology Part B, Biochemistry & Molecular Biology. 2002;133(4): 609–646. doi: 10.1016/s1096-4959(02)00167-7 12470823

[4] Depiereux S, Liagre M, Danis L, De Meulder B, Depiereux E, Segner H, et al. Intersex Occurrence in Rainbow Trout (Oncorhynchus mykiss) Male Fry Chronically Exposed to Ethynylestradiol. PLoS ONE. 2014;9(7): e98531. doi: 10.1371/journal.pone.0098531 25033040 PMC4102465

[5] Amiard J-C, Amiard-Triquet C. Conventional Risk Assessment of Environmental Contaminants. In: Amiard-Triquet C, Amiard J-C, Mouneyrac C, editors. Aquatic Ecotoxicology—Advancing Tools for Dealing with Emerging Risks. London: Academic Press; 2015. pp. 25–49.

[6] Directive 2000/60/EC of the European Parliament and of the Council of 23 October 2000 establishing a framework for Community action in the field of water policy. Official Journal of the European Union. 2000. Available from: http://data.europa.eu/eli/dir/2000/60/oj.

[7] Albl B, Haesner S, Braun-Reichhart C, Streckel E, Renner S, Seeliger F, et al. Tissue Sampling Guides for Porcine Biomedical Models. Toxicologic Patholology. 2016;44(3): 414–420. doi: 10.1177/0192623316631023 26883152

[8] Blutke A, Wanke R. Sampling Strategies and Processing of Biobank Tissue Samples from Porcine Biomedical Models. Journal of Visualized Experiments. 2018(133): e57276. doi: 10.3791/57276 29578524 PMC5931442

[9] Keenan CM, Vidal JD. Standard morphologic evaluation of the heart in the laboratory dog and monkey. Toxicologic Pathology. 2006;34(1): 67–74. doi: 10.1080/01926230500369915 16507546

[10] Ruehl-Fehlert C, Kittel B, Morawietz G, Deslex P, Keenan C, Mahrt CR, et al. Revised guides for organ sampling and trimming in rats and mice-Part 1. A joint publication of the RITA and NACAD groups. Experimental and Toxicologic Pathology. 2003;55(2–3): 91–106. doi: 10.1078/0940-2993-00311 14620530

[11] Kittel B, Ruehl-Fehlert C, Morawietz G, Klapwijk J, Elwell MR, Lenz B, et al. Revised guides for organ sampling and trimming in rats and mice-Part 2. A joint publication of the RITA and NACAD groups. Experimental and Toxicologic Pathology. 2004;55(6): 413–431. doi: 10.1078/0940-2993-00349 15384248

[12] Morawietz G, Ruehl-Fehlert C, Kittel B, Bube A, Keane K, Halm S, et al. Revised guides for organ sampling and trimming in rats and mice-Part 3. A joint publication of the RITA and NACAD groups. Experimental and Toxicologic Pathology. 2004;55(6): 433–449. doi: 10.1078/0940-2993-00350 15384249

[13] Johnson R, Wolf J, Braunbeck T. OECD Guidance Document for the Diagnosis of Endocrine-Related Histopathology of Fish Gonads. Paris: Organization for Economic Co-operation and Development; 2009.

[14] Palate BM, Denoël SR, Roba JL. A Simple Method for Performing Routine Histopathological Examination of the Cardiac Conduction Tissue in the Dog. Toxicologic Pathology. 1995;23(1): 56–62. doi: 10.1177/019262339502300107 7770700

[15] Connon RE, Geist J, Werner I. Effect-Based Tools for Monitoring and Predicting the Ecotoxicological Effects of Chemicals in the Aquatic Environment. Sensors. 2012;12(9): 12741–12771. doi: 10.3390/s120912741 23112741 PMC3478868

[16] Wünnemann H, Weiß K, Arndt D, Baumann M, Weiß R, Ferling H, et al. Umweltqualitätsnormen für Binnengewässer: Überprüfung der Gefährlichkeit neuer bzw. prioritärer Substanzen. Dessau-Roßlau: Umweltbundesamt; 2020.

[17] Wolf JC, Ruehl-Fehlert C, Segner HE, Weber K, Hardisty JF. Pathology working group review of histopathologic specimens from three laboratory studies of diclofenac in trout. Aquatic Toxicology. 2014;146: 127–136. doi: 10.1016/j.aquatox.2013.10.033 24292026

[18] Directive 2001/83/EC of the european parliament and of the council of 6 November 2001 on the Community code relating to medicinal products for human use. Official Journal of the European Union. 2001. Available from: http://data.europa.eu/eli/dir/2001/83/oj.

[19] Regulation (EC) No 1907/2006 of the European Parliament and of the Council of 18 December 2006 concerning the Registration, Evaluation, Authorisation and Restriction of Chemicals (REACH), establishing a European Chemicals Agency, amending Directive 1999/45/EC and repealing Council Regulation (EEC) No 793/93 and Commission Regulation (EC) No 1488/94 as well as Council Directive 76/769/EEC and Commission Directives 91/155/EEC, 93/67/EEC, 93/105/EC and 2000/21/EC. Official Journal of the European Union. 2006. Available from: http://data.europa.eu/eli/reg/2006/1907/oj.

[20] EMA. Guideline on the environmental risk assessment of medicinal products for human use. London: European Medicines Agency; 2006.

[21] ECHA. Guidance on information requirements and chemical safety assessment—Chapter R.10: Characterisation of dose [concentration]-response for environment. Helsinki: European Chemicals Agency; 2008.

[22] Expert Group of the European Commission. Technical Guidance for Deriving Environmental Quality Standards—Guidance Document No. 27, Updated version 2018. Luxembourg: Publications Office of the European Union; 2018.

[23] Ågerstrand M, Breitholtz M, Rudén C. Comparison of four different methods for reliability evaluation of ecotoxicity data: a case study of non-standard test data used in environmental risk assessments of pharmaceutical substances. Environmental Sciences Europe. 2011;23: 17. doi: 10.1186/2190-4715-23-17

[24] Wester PW, van der Ven LTM, Vethaak AD, Grinwis GCM, Vos JG. Aquatic toxicology: opportunities for enhancement through histopathology. Environmental Toxicology and Pharmacology. 2002;11(3–4): 289–295. doi: 10.1016/S1382-6689(02)00021-2 21782612

[25] Rašković B, Poleksić V. Fish Histopathology as Biomarker in Ecotoxicology. In: Berillis P, editor. Trends in Fisheries and Aquatic Animal Health. Sharjah: Bentham Science Publishers; 2017. pp. 155–181.

[26] Mehinto AC, Hill EM, Tyler CR. Uptake and biological effects of environmentally relevant concentrations of the nonsteroidal anti-inflammatory pharmaceutical diclofenac in rainbow trout (Oncorhynchus mykiss). Environmental Science & Technology. 2010;44(6): 2176–2182. doi: 10.1021/es903702m 20175546

[27] Memmert U, Peither A, Burri R, Weber K, Schmidt T, Sumpter JP, et al. Diclofenac: New data on chronic toxicity and bioconcentration in fish. Environmental Toxicology and Chemistry. 2013;32(2): 442–452. doi: 10.1002/etc.2085 23325530 PMC3674524

[28] Schwaiger J, Ferling H, Mallow U, Wintermayr H, Negele RD. Toxic effects of the non-steroidal anti-inflammatory drug diclofenac. Part 1: histopathological alterations and bioaccumulation in rainbow trout. Aquatic Toxicology. 2004;68(2): 141–150. doi: 10.1016/j.aquatox.2004.03.014 15145224

[29] Birzle CF. Etablierung und Validierung quantitativ-morphologischer Parameter bei Regenbogenforellen im Rahmen ökotoxikologischer Fragestellungen. Doctoral Thesis, Ludwig-Maximilians-Universität München. 2015. Available from: https://www.dr.hut-verlag.de/978-3-8439-2059-9.html.

[30] Wolf JC. A Critical Review of Morphologic Findings and Data From 14 Toxicological Studies Involving Fish Exposures to Diclofenac. Toxicologic Pathology. 2021;49(5): 1024–1041. doi: 10.1177/0192623321989653 33596776

[31] Wolf JC, Baumgartner WA, Blazer VS, Camus AC, Engelhardt JA, Fournie JW, et al. Nonlesions, Misdiagnoses, Missed Diagnoses, and Other Interpretive Challenges in Fish Histopathology Studies: A Guide for Investigators, Authors, Reviewers, and Readers. Toxicologic Pathology. 2015;43(3): 297–325. doi: 10.1177/0192623314540229 25112278

[32] Wolf JC. Fish toxicologic pathology: the growing credibility gap and how to bridge it. Bulletin of the European Association of Fish Pathologists. 2018;38(2): 51–64.

[33] Crissman JW, Goodman DG, Hildebrandt PK, Maronpot RR, Prater DA, Riley JH, et al. Best Practices Guideline: Toxicologic Histopathology. Toxicologic Pathology. 2004;32(1): 126–131. doi: 10.1080/01926230490268756 14713558

[34] Gundersen HJG, Mirabile R, Brown D, Boyce RW. Chapter 8—Stereological Principles and Sampling Procedures for Toxicologic Pathologists. In: Haschek WM, Rousseaux CG, Wallig MA, editors. Haschek and Rousseaux’s Handbook of Toxicologic Pathology. Boston: Academic Press; 2013. pp. 215–286.

[35] Klimisch HJ, Andreae M, Tillmann U. A Systematic Approach for Evaluating the Quality of Experimental Toxicological and Ecotoxicological Data. Regulatory Toxicology and Pharmacology. 1997;25(1): 1–5. doi: 10.1006/rtph.1996.1076 9056496

[36] Moermond CTA, Kase R, Korkaric M, Ågerstrand M. CRED: Criteria for reporting and evaluating ecotoxicity data. Environmental Toxicology and Chemistry. 2016;35(5): 1297–1309. doi: 10.1002/etc.3259 26399705

[37] Arndt D, Fux R, Blutke A, Schwaiger J, El-Matbouli M, Sutter G, et al. Proliferative Kidney Disease and Proliferative Darkening Syndrome are Linked with Brown Trout (Salmo trutta fario) Mortalities in the Pre-Alpine Isar River. Pathogens. 2019;8(4): 177. doi: 10.3390/pathogens8040177 31590460 PMC6963635

[38] Fiedler S, Wünnemann H, Hofmann I, Theobalt N, Feuchtinger A, Walch A, et al. A practical guide to unbiased quantitative morphological analyses of the gills of rainbow trout (Oncorhynchus mykiss) in ecotoxicological studies. PLoS ONE. 2020;15(12): e0243462. doi: 10.1371/journal.pone.0243462 33296424 PMC7725368

[39] Geiger T. Untersuchung zur Wirkung oral aufgenommener PVC-Mikroplastikpartikel bei Regenbogenforellen (Oncorhynchus mykiss). Doctoral Thesis, Ludwig-Maximilians-Universität München. 2021. Available from: https://edoc.ub.uni-muenchen.de/28405/.

