# Supplementary material for: Standardized tissue sampling guidelines for histopathological and molecular analyses of rainbow trout (Oncorhynchus mykiss) in ecotoxicological studies
Source: PLoS One. 2023 Jul 13;18(7):e0288542. doi: 10.1371/journal.pone.0288542 (PMC10343068; doi:10.1371/journal.pone.0288542)
Supplement: S1 File — (PDF) [file pone.0288542.s001.pdf]

Standardized tissue sampling guidelines for histopathological and molecular analyses of rainbow trout (*Oncorhynchus mykiss*) in ecotoxicological studies

Sonja Fiedler[1*], Hannah Schrader[2], Natalie Theobalt[1,#a], Isabel Hofmann[1,#b], Tobias Geiger[2,#c], Daniela Arndt[2,#d], Rüdiger Wanke[1], Julia Schwaiger[2], Andreas Blutke[3,#e]

[1]Institute of Veterinary Pathology at the Center for Clinical Veterinary Medicine, Ludwig-Maximilians-Universität München, Munich, Germany

[2]Unit 73 Aquatic Ecotoxicology, Microbial Ecology, Bavarian Environment Agency, Wielenbach, Germany

[3]Institute of Experimental Genetics, Helmholtz Zentrum Munich, Neuherberg, Germany

[#a]Current Address: Bavarian Health and Food Safety Authority, Oberschleißheim, Germany

[#b]Current Address: Veterinary Inspection Office, Fürstenfeldbruck District Office, Fürstenfeldbruck, Germany

[#c]Current Address: Veterinary practice for fish, KoiDoc, Lauingen, Germany

[#d]Current Address: Pre Clinical Safety (PCS) Consultants Ltd., Basel, Switzerland

[#e]Current Address: Institute of Veterinary Pathology at the Center for Clinical Veterinary Medicine, Ludwig-Maximilians-Universität München, Munich, Germany

* Corresponding author:

E-mail: Sonja.Fiedler@patho.vetmed.uni-muenchen.de (SF)

Supplementary material

Contents

1. Introduction 3
2. Sampling guides for organs and tissues of rainbow trout used in ecotoxicological studies. 6
2.1 Respiratory system (Gills) 6
2.2 Cardiovascular system: Heart and major blood vessels 11
2.3 Digestive system 15
2.3.1 Tongue and teeth 15
2.3.2 Liver 18
2.3.3 Gastrointestinal tract 22
2.3.4 Pancreas (Exocrine and endocrine pancreas) 27
2.3.5 Swim bladder 31
2.4 Adipose tissue 34
2.5 Spleen 38
2.6 Reproductive system 41
2.6.1 Testes 41
2.6.2 Ovaries 45
2.7 Kidneys 49
2.8 Central nervous system: Brain and spinal cord 54
2.9 Integument: Scaled and non-scaled skin 60
2.10 Locomotor system 65
2.10.1 Skeletal musculature 65
2.10.2 Bones and cartilage 69
2.10.3 Fins 74
2.11 Pseudobranchs 78
2.12 Sensory system 81
2.12.1 Olfactory system 81
2.12.2 Inner ears 85
2.12.3 Lateral line system 88
2.12.4 Eyes 91
2.13 Endocrine system 96
2.13.1 Pituitary gland (Hypophysis) 97
2.13.2 Endocrine pancreas 98
2.13.3 Thyroid gland 99
2.13.4 Interrenal and suprarenal tissue 100
2.13.5 Corpuscles of Stannius 101
2.13.6 Pineal gland (Epiphysis) 103
2.13.7 Urophysis 104
2.13.8 Ultimobranchial gland 105
3. References 106

1. Introduction

The general (clinical and pathological) examination procedures of teleost fish including collection of body liquids, clinical samples and specimen for parasitological and microbiological analyses, as well as anesthesia/killing methods, necropsy and dissection techniques have previously been described in detail [1-4]. In the present guidelines, standardized tissue sampling protocols are presented for ~40 organs and tissues. The guidelines are specifically adapted to rainbow trout of 300-2000 g body weight, which are frequently used in ecotoxicological studies [5-12]. The abundantly illustrated protocols will ensure an efficient sampling of high-quality specimens, adequately representing the organs and tissues of rainbow trout used in ecotoxicological studies[*]. The proposed necropsy- and sampling protocol is hierarchically structured with regard to the fragility of organs and a conclusive, efficient necropsy- and sampling process. For each organ/tissue, a step-by-step protocol is provided, allowing the sampling of all ecotoxicologically relevant organs and tissues from a single rainbow trout. The sampling and processing of **specimens for (histo-) morphological as well as molecular analyses** from one organ/tissue location allows for the performance of different analysis methods on the same organ/tissue location for the detection of toxic effects of test substances, which may manifest in morphological, molecular and/or functional organ/tissue alterations. The proposed sampling regime and sample processing are adjusted to subsequent routine analyses. These include histopathology, molecular and biochemical analyses of DNA, RNA, proteins, lipids or metabolites as well as analytical chemistry analyses (for convenience, molecular, biochemical and analytical chemistry analyses are hereinafter combined under the term “molecular analyses”). If macroscopically evident lesions are present, additional samples should be taken from the altered sites for histopathology and microbiological, parasitological, molecular *etc.* analyses, as appropriate. In the present guidelines, the cutting directions, section planes, and processing steps for the downstream analyses of organ/tissue samples are defined and indicated by pictograms (**Figures 1-3**).

---

[*]***Ethics statement***

For development, demonstration and validation of the methods shown in the present study, eight healthy rainbow trout of both sexes with body weights ranging from 300-2000 g were sacrificed. The use of the fish in this study was performed in accordance with the relevant legal regulations and with permission of the local authorities, and was approved by the institutional ethics committee of the Institute of Veterinary Pathology of the Ludwig-Maximilians-Universität Munich via verbal consent. The fish were obtained from the breeding facility of the Bavarian Environment Agency in Wielenbach, Germany. After initial health status check, fish were sacrificed either by stunning (concussion) and exsanguination or with tricaine methanesulphonate solution (500 mg/l, Tricaine Pharmaq® 1000 mg/g (Pharmaq Ltd., United Kingdom)) and subsequent brain destruction after circulatory arrest, using a sharp 14 gauge cannula (Braun® Sterican®, B. Braun Melsungen AG, Germany). In none of the examined fish, clinical, macroscopic, and histological examination revealed indications of disease or pathological alterations.

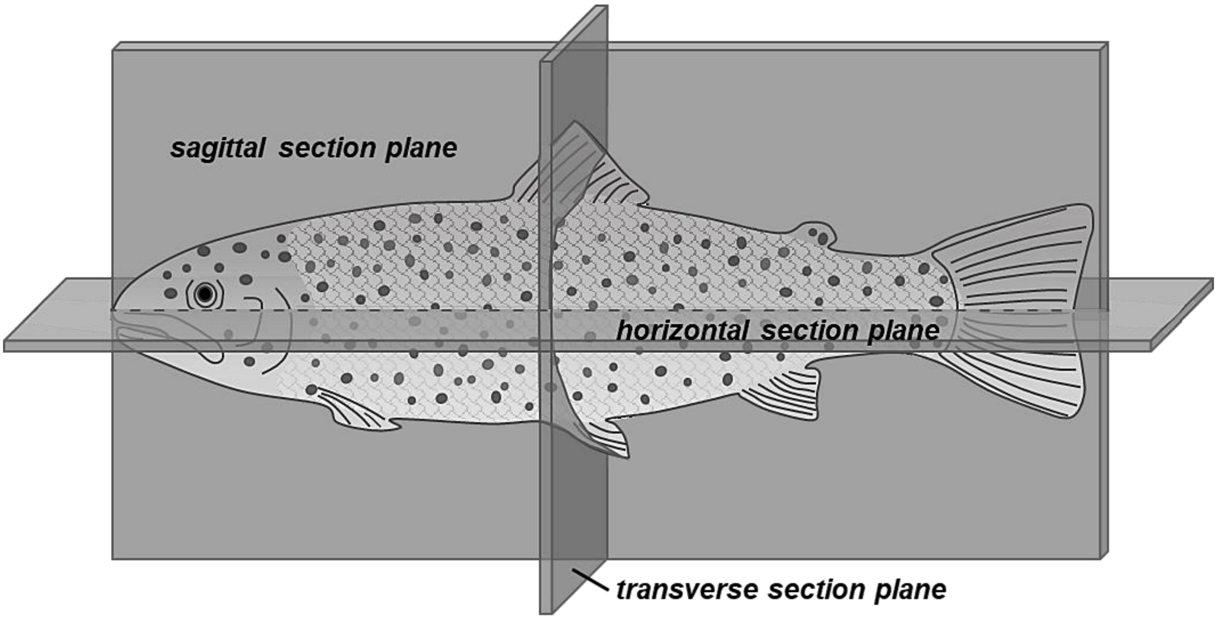

Figure 1. Schematic illustration of the section plane orientations. The section plane orientations/directions used in the present guidelines are the (mid-) sagittal (*i.e.,* vertical) section plane, the horizontal section plane and the transverse (*i.e.,* frontal or cross section in elongated or hollow organs) section plane.

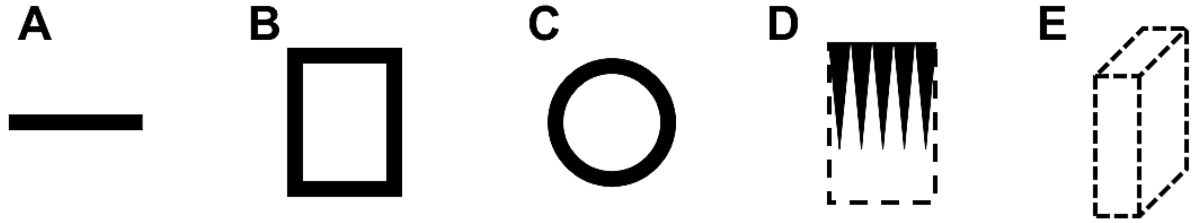

Figure 2. Symbols used in the present guidelines regarding the sampling of histopathological and molecular specimens. A. Section plane of the histological section orthogonal to the picture plane. **B.** Specimen for molecular analyses, generated using scalpel or scissors. **C.** Specimen for molecular analyses, generated using a biopsy punch. **D.** Section plane of the histological section parallel to the image plane. **E.** Three-dimensional (3D) cutting level for histopathological analyses.

In general, **specimens for histopathological analyses** are immersion-fixed in neutrally buffered 4% formaldehyde solution (formalin-fixed, FF) and embedded in paraffin (paraffin-embedded, PE). The histological sections are routinely stained with hematoxylin and eosin (HE). If other fixatives, embedding media or histological stains are more suitable, this is explicitly mentioned in the corresponding chapters. If appropriate, immersion fixation can be preceded by vascular perfusion fixation as illustrated in **Chapter 2.3.2** and described earlier [13]. For most organs, next to sampling for histopathological analyses also sampling for subsequent **molecular analyses** is scheduled. For proper tissue preservation, samples for molecular analyses are frozen (liquid nitrogen or dry ice) and stored at -20°C or -80°C (short-term storage) depending on the intended analysis (analytical chemistry or molecular analysis). For prolonged storage of tissue samples, storage at -150°C is recommended [14].

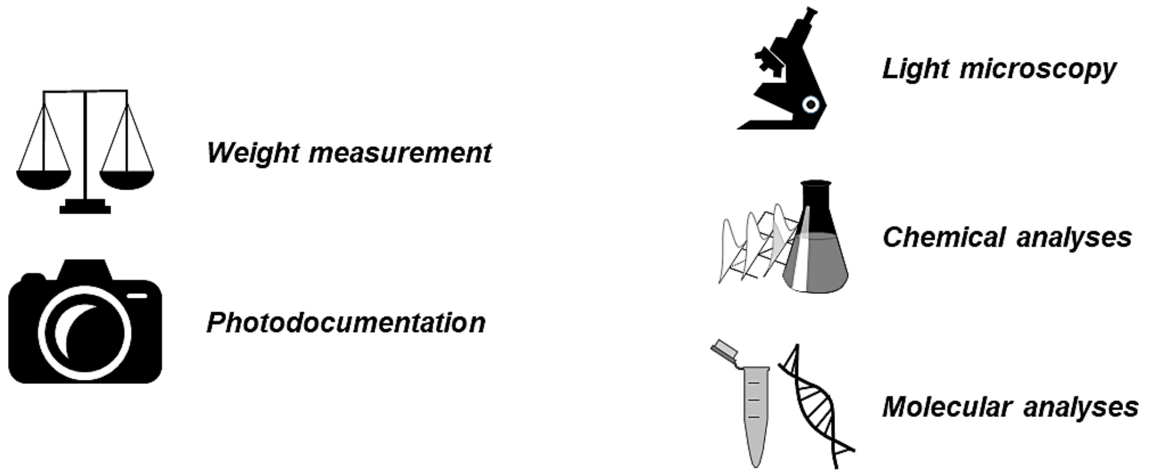

Figure 3. Pictograms indicating initial and further organ-/tissue sample analyses. Weight measurements are performed to the nearest g (fish body weight) or nearest mg (organs or tissue samples), using a precision scale. For photodocumentation, information on the identity of the test fish as well as a size scale are to be prepared and presented on the photograph. Specimens for routine light microscopic histopathological analyses are fixed in neutrally buffered 4% formaldehyde solution and embedded in paraffin (FF-PE), specimens for molecular analyses are snap-frozen using liquid nitrogen or dry ice and stored at -20°C or -80°C for short-term storage (analytical chemistry or molecular analysis), or at -150°C for prolonged storage of tissue samples (molecular analyses).

The proposed sampling regime is considered adequate for the demands of routine ecotoxicology studies, whereas the generation of samples for more advanced analyses, such as immunohistochemistry, *in situ* hybridization, electron microscopy or quantitative stereological analyses are not in the scope of the present guidelines. In studies scheduling advanced analyses of distinct organs/tissues, however, additional sampling efforts and different tissue sample processing methods may be necessary. Depending on the objectives and the experimental design of a given study, the number of organs and tissues to be sampled as well as the applied sampling scheme and the sample numbers has to be adapted accordingly. For **quantitative stereological analyses**, the interested reader is referred to the standard textbooks of quantitative stereology [15-17] as well as standardized organ/tissue sampling guides established for different experimental animal species and organ systems [13, 18-22].
The **development of a structured, study-specific sampling protocol in advance to the planned necropsy** is highly recommended, *inter alia* considering the organ`s/tissue`s tendency to degrade or the technical and material requirements for organ/tissue sampling and sample processing [21, 23].

2. Sampling guides for organs and tissues of rainbow trout used in ecotoxicological studies

2.1 Respiratory system (Gills)

Relevant anatomical features/preparation

Gills are multifunctional organs. Next to their role in aquatic gas exchange they are involved in excretion of nitrogenous waste, osmotic- and ionic- regulation processes or the regulation of the acid-base balance [24]. Rainbow trout possess four pairs of gills (holobranchs I-IV), bilaterally located in the opercular chamber and covered by the opercula [2, 24, 25]. The pseudobranch (*i.e.,* a vestigial gill arch), which is covered by opercular epithelium, does not participate in gas- or ion exchange [26] and is not considered in the present gill tissue sampling protocol. The sampling of the pseudobranch for molecular and histopathological analyses is illustrated in detail in **Chapter 2.11.** All hemibranchs are composed of a gill arch with the interbranchial septum and the gill filaments. The gill arch consists of the gill arch skeleton, musculature and vasculature. The gill arch bears gill rakers at its concave margin and supports the two rows of gill filaments (hemibranchs) extending from its convex margin [2, 24, 25, 27]. The hemibranchs consist of the macroscopically visible gill filaments (primary lamellae, PL), which are supported by cartilaginous rods. Respiratory lamellae (secondary lamellae, SL) originate from the ventral and dorsal surfaces of the PL [2, 24, 25]. In trout, the proximal one to two thirds of the gill filaments are supported by an interbranchial septum, whereas the distal portions of the gill filaments are not embedded in the septal tissue [2, 27]. The secondary lamellae are the functional base unit of the gills. Histologically, they are composed of vascular spaces which are delimited by the pillar cells (*i.e.,* modified endothelial cells, specific to fish gills and defining the vascular blood spaces with their cell flanges) and an epithelium comprised mainly of pavement cells, but also *e.g.,* chloride- and goblet cells [2, 4, 27].

A detailed description of the complex three-dimensional gill (histo-) architecture is provided in Evans et al. [24] or Fiedler et al. [13]. Adequate killing methods for gill analyses as well as a detailed description of the excision of gill samples is also provided in Fiedler et al. [13]. If considered beneficial for the study purpose, vascular perfusion fixation may be performed, the technique of perfusion fixation of rainbow trout tissue is described in **Chapter 2.3.2** and elsewhere [13]. Briefly, killing of fish by overdosed anesthetic is advantageous, since a blow on the head may lead to gill hemorrhages. At necropsy, the gills are dissected immediately after killing of the fish in order to preserve the fragile gill structures. After removal of the left body wall and the opercula, the anterior portion of the esophagus is transected and gills are excised by severing the cleithrum and the dorsal and ventral connection between the gill basket and the skull. The gill basket is extracted from the head by gently pulling in ventral direction and adhering organs and tissues are removed, subsequently the gill basket is carefully divided in the midline (**Figure 4B**). The holobranchs of the left side, which are sampled for routine histopathological analysis (**Figures 5B** and **6B**), are immediately transferred to neutrally buffered 4% formaldehyde solution for immersion fixation to prevent autolysis and deterioration of tissue morphology. It is highly recommended to remove the bony gill arches before sample embedding for histopathological analyses, otherwise the holobranchs should be decalcified in advance [28, 29]. The gills of the right body side are separated for weighing, macroscopic examination and sampling of fresh (*i.e.,* unfixed) tissue specimens for molecular analyses (**Figures 5A** and **6A**). Remaining tissue is preserved (*i.e.,* immediately transferred to adequate fixative) to ensure that sufficient sample material is available for new or expanded scientific issues arising from the analyses.

2.1 Respiratory system (Gills)

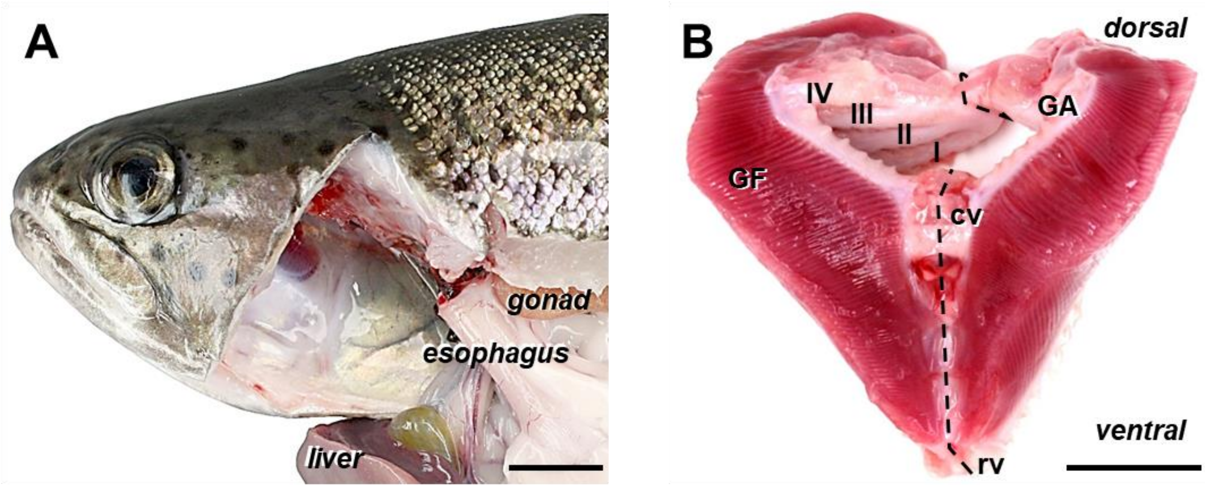

Figure 4. Excision of the rainbow trout gill basket. A. Lateral aspect of the head after removal of the left operculum and the gill basket. The gills are excised by severing the cleithrum, removal of the operculum, transection of the esophagus and severing the dorsal and ventral connection between the gill basket and the skull. The gill basket is removed from the head by gently pulling it in ventral direction (refer to Fiedler et al. [13]). **B.** Caudo-ventral aspect of the excised gill basket. Adhering organs and tissues (*i.e.,* the cranial aspect of the esophagus, heart and parts of the flank) are carefully removed for subsequent separation and immersion fixation. The gill basket is divided in the midline (black dashed line), the fresh (*i.e.,* unfixed) gills of the right body side are subject to the macroscopic examination and sampling for molecular analyses, whereas the left half of the gill basket is immediately immersion-fixed for standard histopathological analyses. The four holobranchs are numbered (**I-IV**) and important morphological structures/orientations are indicated: **GF:** Gill filaments; **GA:** Gill arches; **rv:** Rostro-ventral; **cv:** Caudo-ventral. Bars = 1 cm.

General examination parameters

The holobranchs are macroscopically examined for pathological alterations and corresponding findings are (photo-) documented before separation of left and right side of the gill basket and immersion fixation of the left side of the gill apparatus. The four freshly excised and separated holobranchs of the right body side are briefly dabbed dry using laboratory paper towel and the weight is recorded. If appropriate, samples of altered gill tissue regions are generated from the holobranchs for histopathological, molecular, parasitological or microbiological analyses.

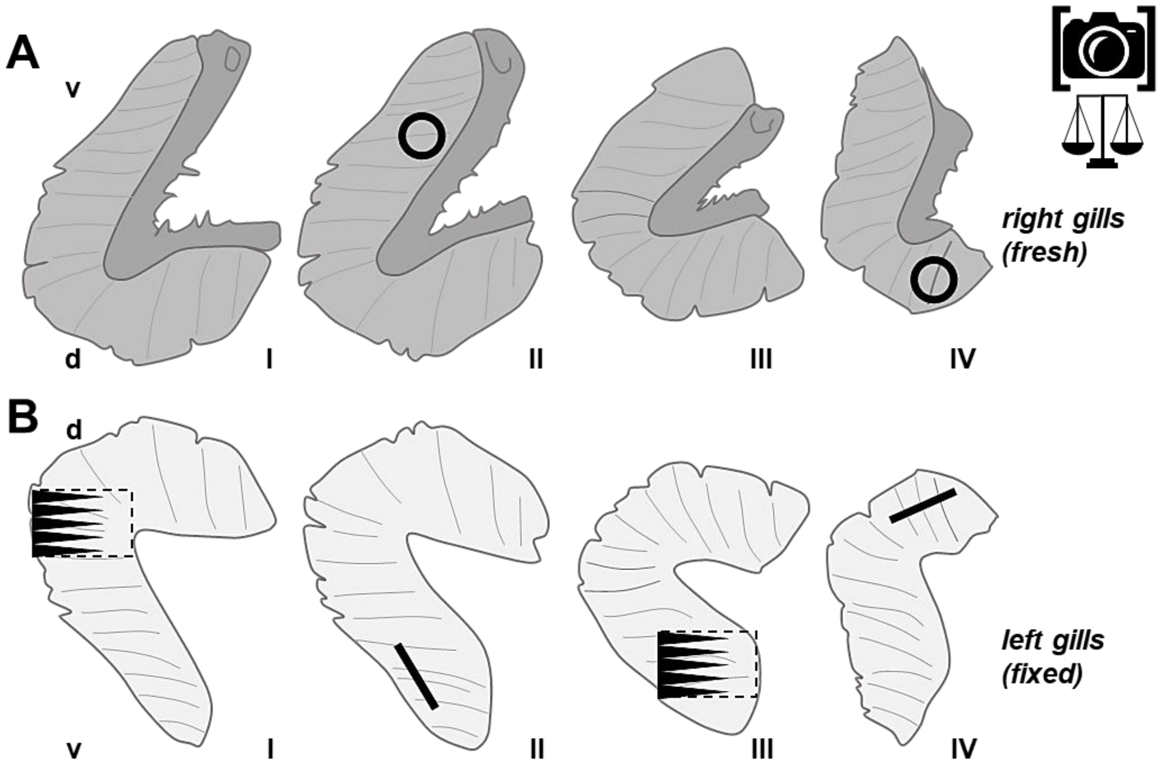

Figure 5. Schematic illustration of the medial aspects of the separated holobranchs with indicated sampling locations for routine histopathological and molecular analyses. A. Unfixed holobranchs with indicated sampling locations for molecular analyses (black circles). The freshly excised gills of the right body side are separated and briefly dabbed dry with laboratory paper towel for macroscopic examination and weighing. Gill filament tissue samples for molecular analyses are excised, using a biopsy punch of 0.6 cm diameter. **B.** Immersion-fixed holobranchs with indicated sampling locations for histopathological analyses. The gills of the left body side are immediately transferred to fixative solution after excision and dividing of the gill basket. The fixed gills are separated and the bony gill arches are removed before sampling and embedding of the holobranch specimens. **I-IV:** Number of the corresponding gill arch; **v:** Ventral; **d:** Dorsal.

Sampling scheme for routine analyses of rainbow trout gills

1. Samples for molecular analyses of the gills

Location:	Sampling locations are indicated in **Figures 5A** and **6A**.
Number of samples:	Two.
Sample size:	Full thickness tissue cylinder of 0.6 cm diameter, punched out by biopsy punch.
Remarks:	Specimens containing interbranchial septum and gill filament tissue are taken.
Processing:	Samples are frozen (liquid nitrogen) and stored at -20°C or -80°C (short-term storage) depending on the intended analysis (chemical or molecular analysis). For prolonged storage of tissue samples for subsequent RNA- and protein analyses, storage at -150°C is recommended.
Downstream analyses:	DNA-, RNA-, protein-, and other OMICS profiling- or targeted analyses requiring cryo-conserved specimen as well as chemical (analytical) analyses.

2. Samples for histopathological examination of the gills

Location & orientation of sections:	Sampling locations and orientations are indicated in **Figures 5B** and **6B**. Two transverse sections (relative to the orientation of the sectioned gill filaments) are generated, containing gill filaments and interbranchial septum. Two sagittal sections, containing both distal gill filaments (*i.e.,* not embedded in septal tissue) and gill filaments embedded in septal tissue, are generated.
Number of samples:	Four.
Section plane size:	Frontal section plane: approximately 0.5 cm x 0.2 cm, sagittal section plane: approximately 0.6 cm x 0.6 cm (length x width).
Fixation & embedding:	FF-PE.

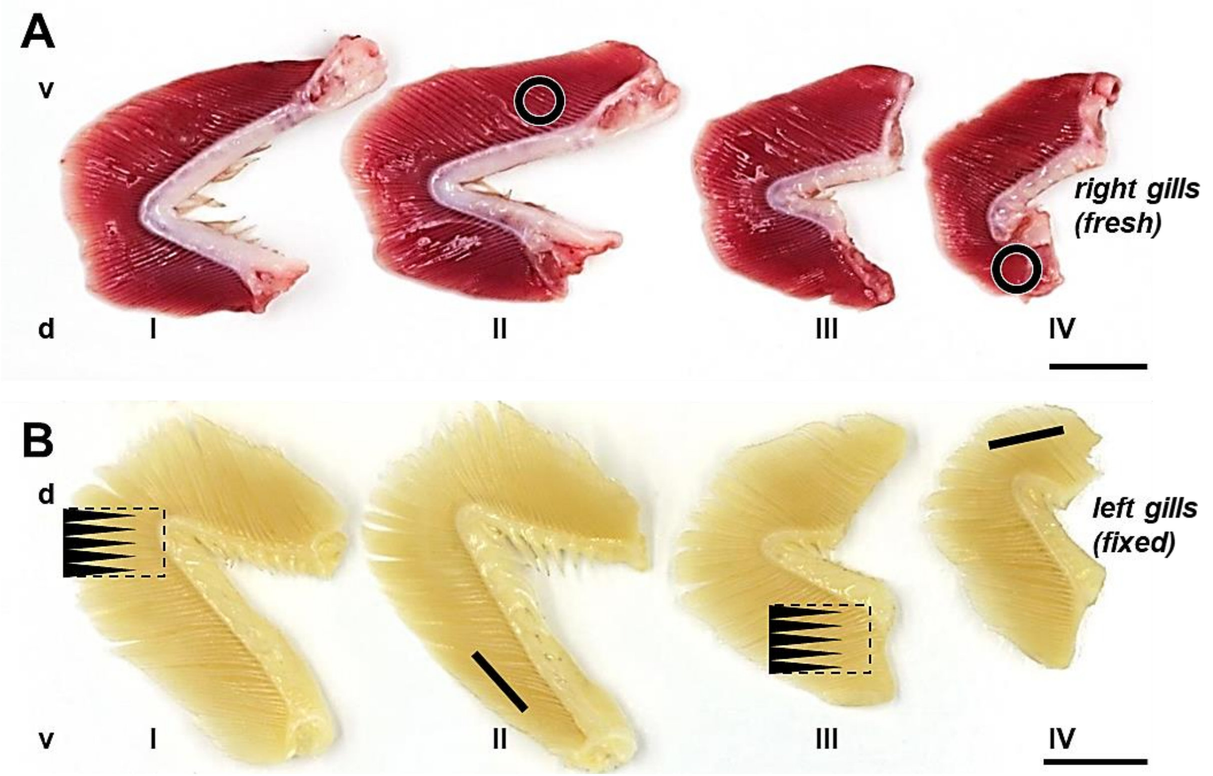

Figure 6. Sampling of standard gill tissue specimens in routine ecotoxicological studies. **A.** Medial aspects of freshly excised (unfixed) gills of the right body side. Sampling locations for molecular analysis specimens are indicated (black circles). **B.** Medial aspects of formalin-fixed gills of the left body side. Sampling locations and section plane orientations for histopathological samples are indicated (black lines for transverse sections, black dashed rectangle for sagittal sections, with black triangles indicating the section orientation parallel to the picture plane). For demonstration purposes the gill arches of the displayed gills are not removed, but their removal is recommended for histopathological analyses. **I-IV:** Number of the corresponding gill arch; **v:** Ventral; **d:** Dorsal. Bars = 1 cm.

Time requirements

Approximately 15 minutes are to be scheduled for dissection of the gills, macroscopic examination, sampling and further processing of gill tissue samples. This estimate does not include the time needed for killing the fish, prearrangement of sampling instruments and materials, or the further processing of fixed/frozen specimens.

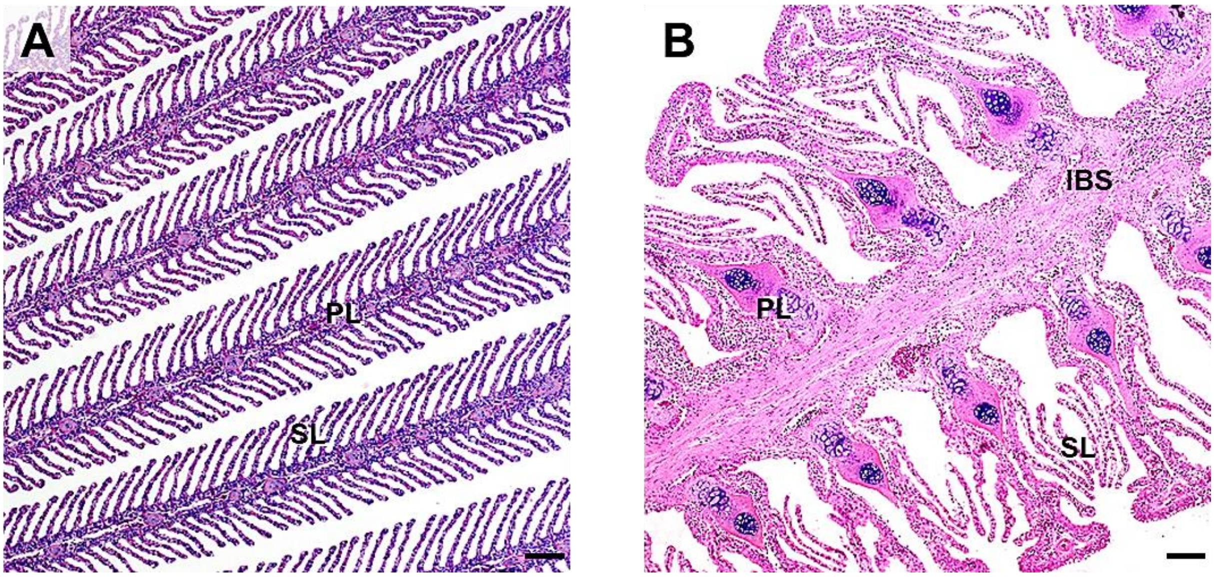

Figure 7. Gill histology. A. Sagittal section of the distal (*i.e.,* free) gill filaments. This section plane orientation is preferred for histopathological analysis of the secondary lamellae as the site of gas- and ion exchange. **B.** Transverse section of gill filaments supported by septal tissue. This section plane orientation is preferred for the investigation of filamental vasculature and examination of distinct cell compartments which are more prevalent in the epithelium of the gill filament edges, *e.g.,* chloride cells [2]. Important morphological structures are indicated: **PL:** Primary lamella; **SL:** Secondary lamella; **IBS:** Interbranchial septum. FF-PE. HE. Bars = 100 µm.

A comparison of the proposed sampling scheme for routine analyses with previously published studies

Only few published ecotoxicological studies examining rainbow trout gill samples provide a more explicit description of the sampling locations, such as the four gills of one side [30], a sample of ~50 gill filaments from the second left gill arch [31], sampling of only the gill filament tips [32] or the gill filament tissue of the first and/or second gill arch [33, 34]. In previous ecotoxicological studies examining rainbow trout, the number of analyzed gill tissue samples per fish, if indicated, ranges between one [31] to four samples [30].

Assuming that gill alterations, experimentally induced by exposure to aquatic pollutants, do not significantly differ from one body side to the other, one side of the gill apparatus is processed and sampled for routine histopathological analyses and the other for molecular analyses. A previous study in rainbow trout has also shown that relevant quantitative morphological gill parameters, such as the volume density of the secondary lamellae in the gill filaments, do not significantly differ in right *vs.* left gills [13]. Related to the arrangement of the gills in four pairs of holobranchs and the function of the gill as a sensitive target organ in ecotoxicological studies, the number and sizes of gill tissue samples, as well as the sampling locations proposed in the present guidelines are regarded to provide sufficiently representative gill tissue specimens for examination of most (qualitative) morphological gill tissue alterations and molecular analyses of trout gill tissue. Note that the sampling protocol may need to be modified, if there is evidence of unilateral lesions (*e.g.,* abnormal necropsy findings) since histopathological and molecular examination of only side of the gill apparatus could falsify the assessment of unilaterally occurring tissue alterations. Furthermore, the sampling protocol has to be adapted, if additional analyses of the thyroid gland or cartilage tissue are planned. If it is intended to examine thyroid tissue, the ventral aspect of the gill apparatus is not separated in the midline but the ventral portions of the gill arches are separated from the gill basked floor individually, so that the gill basket floor is preserved in total (**Chapter 2.13.3**; care must be taken to preserve the gill filaments). If histopathological analysis of cartilaginous tissue is intended, the gill arch of the second right holobranch is separated from the gill filaments as illustrated in **Chapter 2.10.2** for immersion fixation.

2.2 Cardiovascular system: Heart and major blood vessels

Relevant anatomical features/preparation

The rainbow trout`s heart is situated in the pericardial cavity, anterior to the fibrous transverse septum separating the pericardial and the peritoneal cavity, and ventral to the pharynx and esophagus. Laterally, the heart is adjacent to the ventro-caudal branchial skeleton and the pectoral girdle [2-4]. The heart morphology and thickness of the outer compact myocardium may vary due to several factors, such as age, sex, life stage or environmental conditions [4, 35-37]. The heart is composed of four chambers: the sinus venosus, the atrium, the ventricle and the bulbus arteriosus (sequence according to blood flow direction) (**Figures 8** and **9**) [4]. The atrium and the ventricle each consist of a single chamber. The apex of the muscular pyramidal ventricle of the rainbow trout`s heart points caudo-ventrally [2, 4, 36]. Via the central venous system (*i.e.,* the main axial veins and the paired duct of Cuvier), venous blood enters the thin-walled sinus venosus, which is embedded in the transverse septum. The lumen of the sinus venosus is separated from the atrial lumen by sinoatrial valves. The atrium is irregularly shaped and numerous trabeculae extend from the walls of the atrium into the atrial lumen (**Figure 9C**). The atrium and ventricle are connected via the valved atrioventricular ostium. The thick ventricle wall is composed of an outer compact myocardium (which in trout accounts for ~1/3rd of the myocardium) and an inner spongy myocardium, forming numerous trabeculae (**Figure 10A-C**) [2-4]. The ventricular systole pumps blood into the pale, highly elastic bulbus arteriosus (**Figure 8**). Semilunar valves are located at the ventriculobulbar junction [2].
Via the ventral aorta (**Figure 10D**) and the afferent branchial arteries, the blood is pumped from the heart to the gills and the blood is oxygenated within the blood spaces of the secondary lamellae [2-4]. The post-gill or systemic arteries (such as the dorsal aorta and its segmental and visceral artery branches) provide the systemic oxygen supply. In the venous system of teleost fish, two portal systems (*i.e.,* the renal and hepatic portal system) are evident; deoxygenated venous blood from head and trunk is collected in the central venous system and transferred to the sinus venosus [2]. At necropsy, the heart, the transverse septum and the caudal portion of the ventral aorta are removed together with the gills and then isolated from the detached gill apparatus, as previously described in detail in Fiedler et al. [13]. After external macroscopic examination, the histopathological sample of the ventral aorta is transferred to neutrally buffered 4% formaldehyde solution. Subsequently the heart is opened in the midline parallel to the base-apex axis, dividing the bulbus arteriosus, the atrium and the ventricle (**Figure 9A**&**B**). After internal macroscopic examination, the right half of the heart is transferred to neutrally buffered 4% formaldehyde solution as specimen for histopathological analyses (**Figure 9C**). After sampling for molecular analyses (**Figure 9C**) the remaining heart tissue is also preserved (*i.e.,* immediately transferred to an adequate fixative), to ensure that sufficient extra sample material is available for new/expanded scientific issues arising from the analyses.

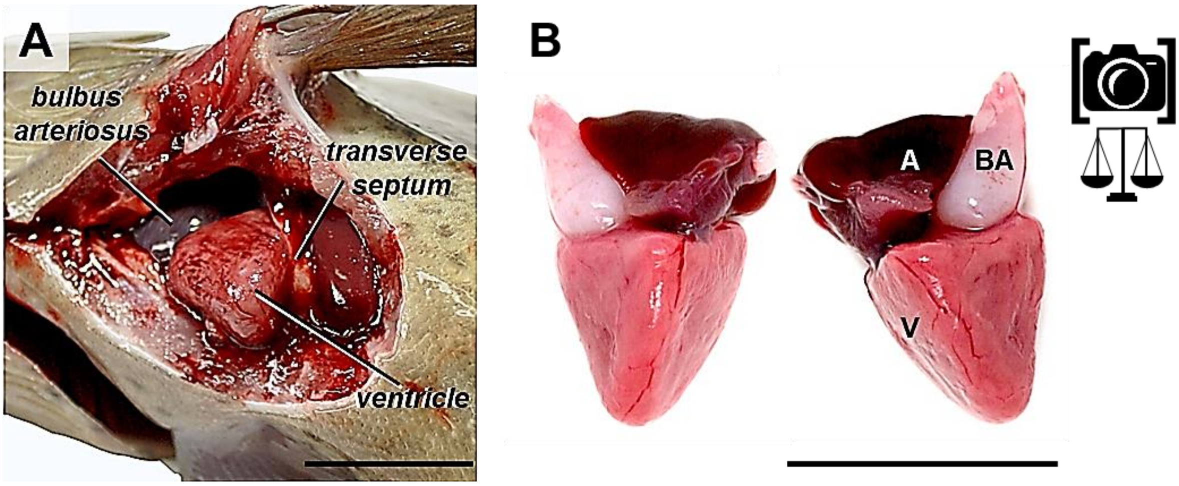

Figure 8. Photographic illustration of the rainbow trout`s heart. A. Ventral aspect of the rainbow trout heart *in situ*. The skin and musculature of the anterior part of the ventral midline are severed, the pericardial cavity is opened and the ventricle is exposed. The heart is situated within the pericardial cavity, anterior to the transverse septum. **B.** Left (left image) and right aspect of the freshly excised rainbow trout heart. Note the pyramidal shape of the ventricle, which is usually found in active, fast-swimming fish such as trout. Morphological structures are indicated: **A:** Atrium; **BA:** Bulbus arteriosus; **V:** Ventricle. Bars = 1 cm.

General examination parameters

The dissected heart is briefly dabbed dry with a laboratory paper towel and weighed to the nearest mg. The heart is macroscopically examined for pathological alterations, corresponding findings are (photo-) documented. If present, samples of altered tissue locations are taken for subsequent histopathological, molecular or microbiological analysis, as appropriate.

Sampling scheme for routine analyses of rainbow trout cardiovascular system

1. Sample for molecular analyses of the heart

Location:	The sampling location is indicated in **Figure 9C**.
Number of samples:	One.
Sample size:	Approximately 0.3 cm x 0.3 cm x 0.2 cm (length x width x height).
Remarks:	A homogenous sample is cut from the apex of the left ventricle wall, containing the endocardium, myocardium and epicardium.
Processing:	Sample is frozen (liquid nitrogen) and stored at -20°C or -80°C (short-term storage) depending on the intended analysis (chemical or molecular analysis). For prolonged storage of the tissue sample for subsequent RNA- and protein analyses, storage at -150°C is recommended.
Downstream analyses:	DNA-, RNA-, protein-, and other OMICS profiling- or targeted analyses requiring cryo-conserved specimen as well as chemical (analytical) analyses.

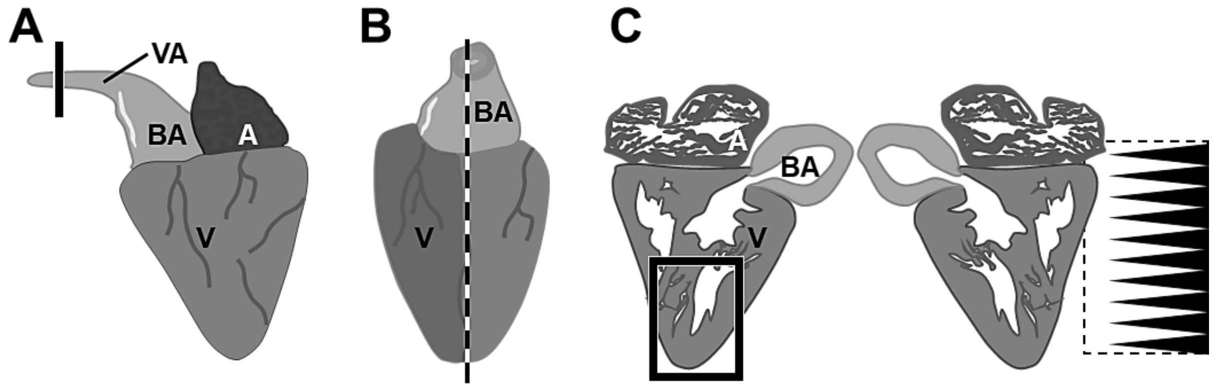

Figure 9. Schematic illustration of the sampling for routine analyses of the rainbow trout cardiovascular system. A. Excised heart and caudal portion of the ventral aorta. The sampling location for routine histopathological examination of the rainbow trout vessels (black line) and important morphological structures are indicated: **VA:** Ventral aorta; **BA:** Bulbus arteriosus; **A:** Atrium; **V:** Ventricle. **B.** Schematic illustration of the cranial aspect of the excised heart. For sampling for routine analyses of the rainbow trout heart, the heart is divided in two halves along the sagittal midline (the section plane is indicated by a dotted line). **C.** Sampling for histopathological examination and molecular analyses of the rainbow trout`s heart. The sampling location of the specimen for molecular analysis (black rectangle), as well as sampling location and orientation of the specimen for histopathological examination (black dotted rectangle with black triangles, indicating the cutting level parallel to the picture plane) are indicated.

2. Samples for histopathological examination of the heart and vessels

Heart

Location & orientation of sections:	Sampling location and orientation is indicated in **Figure 9B**&**C**. A sagittal section is cut from the right side of the heart. The section plane location parallel to the base-apex axis (dividing the left and right halves of the heart) is indicated by the black dashed line in **Figure 9B**.
Number of samples:	One.
Section plane size:	The entire profile of the midline section plane, showing the walls and lumen of the ventricle, the atrium and the bulbus arteriosus.
Fixation & embedding:	FF-PE.

Ventral aorta

Location & orientation of sections:	Sampling location and orientation is indicated in **Figure 9A**. A cross section is cut from the caudal portion of the ventral aorta, approximately 0.5 cm cranial to the bulbus arteriosus.
Number of samples:	One.
Section plane size:	Cross sections of the entire diameter of the ventral aorta, height of the circular specimens is ~0.2 cm.
Fixation & embedding:	FF-PE.

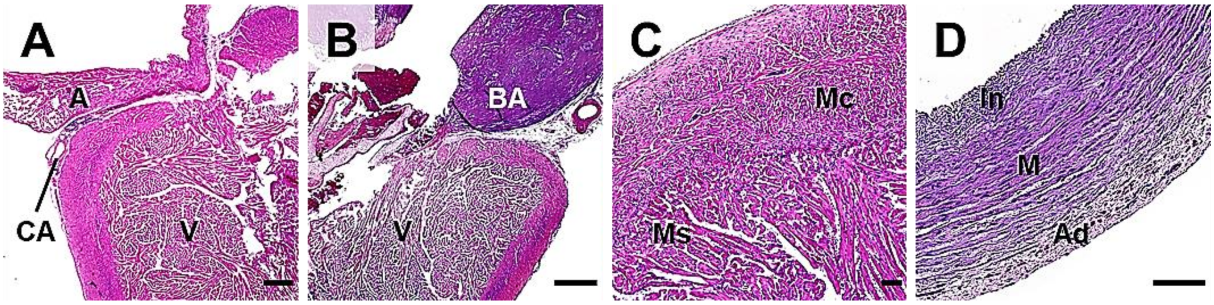

Figure 10. Histology of the rainbow trout heart and wall of the ventral aorta. A. Sagittal section of the ventricle (**V**) and the atrium (**A**). The atrium wall is thinner compared to the ventricle wall, both form trabeculae extending into the lumen. The ventricle wall is supplied with blood via coronary vessels (**CA:** Coronary artery), which run in the ventricle wall periphery. **B.** Sagittal section of the ventricle (**V**) and the bulbus arteriosus (**BA**). **C.** Histology of the sagittally sectioned ventricle myocardium. It is composed of an outer compact myocardium (**Mc**) and an inner spongy myocardium (**Ms**), which forms numerous trabeculae. **D.** Histology of the cross-sectioned wall of the ventral aorta. As in other vertebrates, the wall of the teleostean arteries (and veins) is composed of three major layers: the intima (**In**) as the innermost layer with the endothelium lining the lumen of the blood vessel, the media (**M**), the middle layer mainly composed of circular elastic fibers and some muscle cells, and the outer adventitia (**Ad**), which is mainly composed of collagen fibers. FF-PE. HE. Bar = 500 µm in **A**&**B** and = 100 µm in **D**&**C**.

Time requirements

Approximately 10 minutes are to be scheduled for dissection of the heart (together with the gills), isolation of the heart and ventral aorta from the gill apparatus, macroscopic examination, sampling and further processing of the histopathological and molecular samples. This estimate does not include the time needed for killing the fish, prearrangement of sampling instruments and materials, or the further processing of fixed/frozen specimens.

A comparison of the proposed sampling scheme for routine analyses with previously published studies

Previously published ecotoxicological studies that provide more detailed information on the number or size of heart tissue samples of the rainbow trout, either sample the whole organ [38] or divide the heart in two parts [39] for subsequent analyses.

The sampling location and section plane orientation for histopathological examination of the rainbow trout heart is chosen according to the ecotoxicological study by Incardona et al. [40], illustrating the sampling for the histopathological examination of the heart of pink salmon (*Oncorhynchus gorbuscha*) exposed to crude oil. The sampling of the ventral aorta for the histopathological examination of the blood vessel wall of the rainbow trout is chosen, since the three major layers of the blood vessel walls are well developed in the ventral aorta [36], which additionally can directly be removed together with the heart. The number and sizes of tissue samples as well as the sampling locations proposed in the present guidelines are regarded to provide sufficiently representative tissue specimens for the examination of the most (qualitative) morphological alterations of the structures of the circulatory system and molecular analyses of the rainbow trout heart.

2.3 Digestive system

2.3.1 Tongue and teeth

Relevant anatomical features/preparation

In rainbow trout, tongue and teeth, as the entire gastrointestinal tract, are adapted to a predatory diet. The **tongue** of fish is far less developed than that of other vertebrates. It is of flattened pyramidal shape and can be divided in apex, body and root (**Figure 11**). All regions have different roles in the food intake, *e.g.,* catching or retaining of nourishments [1, 2, 41]. The entire dorsal surface of the tongue is covered by a non-keratinized stratified pavement epithelium. Next to taste buds and fungiform-like papillae, teeth are present, supporting the mechanical ingestion functions of the tongue [2, 4, 41]. The teeth are located at the lateral edges of the apex and body of the tongue and are permanently replaced (polyphyodonty) [41, 42]. In contrast to many other bony fish and higher vertebrates, the muscular component of the rainbow trout tongue is not strongly developed. Instead, several tissue types (including adipose tissue, different types of connective tissue, cartilage or bone) perform different mechanical functions (**Figure 12A**). Depending on the region of the tongue, the tissue compartments are differently developed and distributed [41]. With several types of taste buds located on the tongue (**Figure 12B**), it is also part of the gustatory system and responsible for differentiation, selection and assessment of food. The rainbow trout is estimated to have a more sensitive sense of taste than many other fish species [41, 43, 44].
In rainbow trout, **teeth** are associated with the tongue and the upper and lower jaws [42, 45]. The teeth of the rainbow trout are under permanent tooth exchange, which follows a certain scheme that maintains a functionally integrated dentition [42, 45]. The tips of the conical teeth point posteriorly, and the teeth lack dental roots [2, 42]. In teleosts, in the center of the tooth a mesenchymal dental papilla is present, with dentin-secreting odontoblasts in its periphery (**Figure 12C**). In the mature tooth, the dental pulp is surrounded by dentine. The upper free part of the dentine core is covered by enameloid, which, in contrast to higher vertebrates, is innervated and pervaded by odontoblast processes [1, 2, 36, 42].
At necropsy, the tongue is excised together with the gill apparatus as previously described in detail [13]. The tongue is separated from the gill apparatus and immersion-fixed in neutrally buffered 4% formaldehyde solution. The immersion-fixed tongue is parasagittally (and optionally also crossly) sectioned (**Figure 11**) for histopathological analysis of the tongue and teeth. After sampling, the remaining tissue of the tongue is preserved (*i.e.,* immediately transferred to an adequate fixative), to ensure that sample material is available for new/expanded scientific issues arising from the analyses.

General examination parameters

After excision and separation from the gill apparatus, the tongue is macroscopically examined for pathological alterations and corresponding findings are (photo-) documented, if appropriate. Samples for subsequent histopathological or molecular analysis are taken from the altered locations, if required.

2.3.1 Tongue and teeth

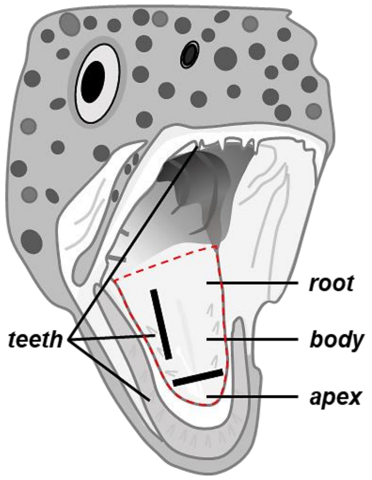

Figure 11. Schematic illustration of the sampling of the tongue and teeth for histopathological analyses. Rostro-dorsal aspect of the opened orobranchial cavity of the rainbow trout. Note that for demonstration purposes the tongue and gill apparatus are illustrated as *in situ*. For sampling for histopathological analyses (and in case of the gills, also for molecular analyses), the tongue and gill apparatus are removed from the orobranchial chamber. The tongue is situated on the floor of the oral cavity (outlines are indicated by the red line). It can be divided in apex, body and root, all parts with different functions in food intake. Next to taste buds and papillae, the tongue also bears teeth on its dorsal surface. The sample locations and section plane orientations of the samples for histopathological analyses are indicated (black lines). For tongue- and teeth analyses a parasagittal section is generated, the additional generation of a cross section of the apex is optional (**Figure 12C**).

Sampling scheme for routine histopathological analyses of rainbow trout tongue and teeth

Location & orientation of sections:	Sampling locations and orientations are indicated in **Figure 11**. A parasagittal section of the right side of the tongue including all three tongue regions and teeth is generated. Optionally, a cross section of the apex is generated for more extensive analyses of tongue- or teeth tissue.
Number of samples:	One parasagittal section, optional: one cross section.
Section plane size:	Approximately 1 cm x 0.7 cm (length x width).
Fixation & embedding:	FF-PE.

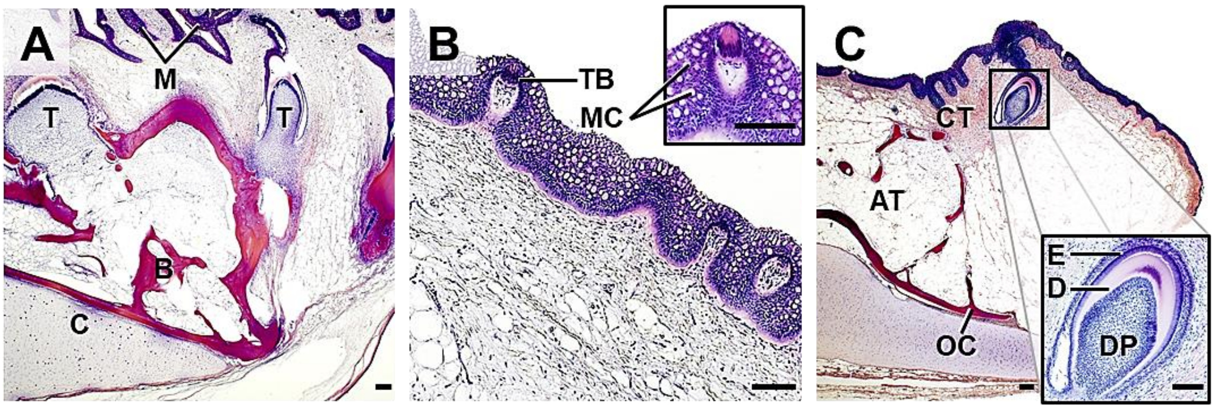

Figure 12. Histology of the rainbow trout tongue and teeth. A. Parasagittal section of the body of the tongue. Dorsally, the mucosa (**M**) is present, which covers the entire surface and the body of the tongue. In deeper layers, developing teeth (**T**) and osteo-cartilaginous tissue (hyaline cartilage (**C**) and bone tissue (**B**)) are present. **B.** Histology of the mucosa covering the dorsal surface of the tongue. The mucosa is a stratified pavement epithelium (non-keratinized), containing mucous cells (**MC**) and taste buds (**TB**) (detail enlargement). **C.** Cross section of the apex of the tongue, with a developing tooth (detail enlargement). The apex is mainly comprised of irregular connective tissue (**CT**), adipose tissue (**AT**), in the more caudal parts of the apex also osteo-cartilaginous tissue (**OC**) is present. In developing teeth, the dental papilla (**DP**) with odontoblasts in its periphery is surrounded by dentin (**D**) and the enamel organ (**E**). FF-PE. HE. Bars = 100 µm.

Time requirements

5-10 minutes are to be scheduled for dissection of the gill apparatus and the tongue, the macroscopic examination and sampling of the tongue and further processing of the rainbow trout tongue sample(s) for histopathological analyses. This estimate does not include the time needed for killing the fish, prearrangement of sampling instruments and materials, or the further processing of fixed specimens.

A comparison of the proposed sampling scheme for routine analyses with previously published studies

According to the authors knowledge, the study of rainbow trout tongue and teeth has received little attention in ecotoxicological research so far and is no standard practice in ecotoxicological studies. However, since the function and structure of taste buds as compartment of the gustatory system of teleost fish may be negatively affected by the exposure to several aquatic contaminants (*e.g.,* pesticides or heavy metals) [43] and the tongue and teeth are directly exposed to the aquatic environment, these guidelines recommend the generation of tongue and teeth samples for standard histopathological analyses. The generation of specimens for molecular analyses is not included but may be performed if deemed appropriate for the study design.

The present sampling protocol is suitable for the histological presentation of all tissues of the tongue, including dental tissue. However, if the main focus is on the taste buds of the tongue as part of the gustatory system, the protocol must be adapted accordingly. For this study objective, a cross section through the root of the tongue is the recommended section plane orientation respectively sample location, as taste buds are found especially on the root of the tongue [41]. In these guidelines, examination of teeth located on the tongue is recommended, since it is a time-saving method for the generation of tooth specimens. If further or more advanced analyses of the teeth are intended, the sampling protocol needs be adapted accordingly (*e.g.,* by sampling the teeth of the upper and lower jaws).

2.3.2 Liver

Relevant anatomical features/preparation

The rainbow trout liver is situated in the anterior abdominal cavity and presents as a solid organ without macroscopically evident lobulation. The liver size/weight as well as the (histopathological) hepatic morphology is highly variable, depending on *e.g.,* the season, the nutritional status or pollutant exposure of the fish [2, 4, 36, 42]. In contrast to the mammalian liver, the liver of rainbow trout is not divided into distinct liver lobules, a portal triad is missing, and hepatocytes are arranged in tubules [4, 46]. The functions of the fish liver resemble that of mammals, as the liver is involved in *e.g.,* protein synthesis, detoxification and immunological processes [2, 4].

At necropsy, the liver is removed from the abdominal cavity together with the gastrointestinal tract after opening of the abdominal cavity and gill dissection. During liver dissection, attention should be paid to not perforate the gall bladder. If deemed beneficial for the study purpose, vascular perfusion fixation may be performed before dissection of the liver. The technique of vascular perfusion fixation of rainbow trout tissue is described in the present chapter and elsewhere [13].

Vascular perfusion fixation

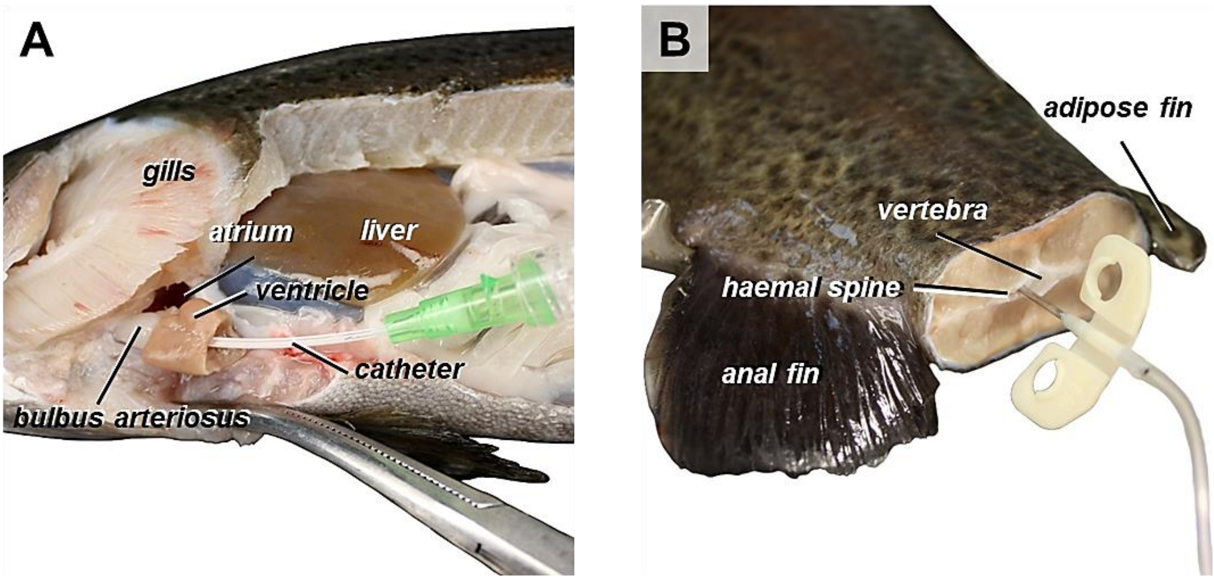

Figure 13. Vascular perfusion fixation of the rainbow trout. A. Cardiac vascular perfusion. After anesthesia and killing of the rainbow trout, the ventro-lateral body wall is promptly removed and the transverse septum and the pericard are incised to expose the heart ventricle and the bulbus arteriosus. The ventricle is punctured with a perfusion catheter cannula, after removal of the catheter cannula the flexible catheter is carefully pushed into the bulbus arteriosus. When perfusion with phosphate buffered saline (PBS) is started, the heart ventricle apex is punctured to create an outflow for the blood removed from the circulation and for the perfusate/fixative solution. Note the pale color of the liver and the gills after removal of the blood from the vascular system. **B.** Arterial retrograde vascular perfusion fixation. The caudal portion of the rainbow trout is transected with a transversal section immediately caudal to the adipose fin. The cannula is inserted into the lumen of the dorsal aorta, which is situated within the haemal canal of the haemal spine. An outflow for blood/perfusate/fixative solution is created via opening the ventricle of the exposed heart (as illustrated in **A**).

2.3.2 Liver

Vascular perfusion fixation of fish removes cellular blood compartments from the vasculature and allows for an excellent preservation of the morphological and cellular structure at light- and electron microscopic levels due to the *in situ* fixation even of tissues/organs difficult to assess [47, 48]. If deemed necessary or advantageous, a whole-body perfusion fixation of the rainbow trout can be performed. A cardiac vascular perfusion fixation method using a gravity feed perfusion apparatus is described in detail in Fiedler et al. [13]. Briefly, two containers are each connected with infusion tubes and 3-way walves, one container is filled with isosmotic buffer solution (*e.g.,* phosphate buffered saline of ~310 mosmol/kg and a pH of ~7.5) [1, 49] of ambient water temperature, the other contains fixative solution (*e.g.,* neutrally buffered 4% formaldehyde solution). According to the physiological blood pressure of the ventral aorta (~40 mmHg) [50, 51], the containers are placed at a height of about ~50 cm above the rainbow trout (referring to the level of liquid in the container). The heart and bulbus arteriosus are exposed, and the heart ventricle is punctured with a perfusion catheter cannula. The catheter is pushed into the bulbus arteriosus and connected to the perfusion tube. While starting the perfusion with PBS, the ventricle apex is pierced to create an outflow for the blood and perfusate. After successful removal of the blood from the circulation (indicated by a pale color of gills or liver (**Figure 13A**)) after approximately 3 minutes, perfusion is subsequently continued with fixative solution for additional 3 minutes. Well-fixed organs/tissues are characterized by a firm consistency [48]. By cannulating the ventral aorta, the entire systemic circulation is perfused [52], this perfusion fixation is recommended for analyses of cephalic tissues (*e.g.,* the gills or brain). Perfusion fixation can also be accomplished by retrograde perfusion fixation through the dorsal aorta (*e.g.,* for liver analyses) (**Figure 13B**) [53]. The heart is exposed as illustrated for cardiac vascular perfusion and the tail portion is removed by a cross section caudal to the adipose fin. The dorsal aorta is embedded in the haemal canal of the haemal spine at the ventral aspect of the vertebral column. A cannula is inserted into the lumen of the dorsal aorta and the outflow for the blood and perfusate is created by piercing the heart ventricle. It has to be considered that the dorsal aortic pressure differs from that of the ventral aorta and the height of the containers has to be adjusted accordingly. (In teleosts, the blood pressure of the dorsal aorta is between 15-30 mmHg [51, 52, 54]. Corresponding to a blood pressure of 30 mmHg, the containers are hung ~40 cm above the level of the fish.) The perfused organs/tissues are then post-fixed by immersion in neutrally buffered 4% formaldehyde solution until further processing. Note that inadequately high perfusion pressures and/or flow rates can easily cause severe (histo-) morphological artifacts in various organs and tissues and substantially impede subsequent qualitative (and quantitative) morphological analysis [13].

General examination parameters

The liver (including the gall bladder) is briefly dabbed dry with a laboratory paper towel and weighed to the nearest mg. The liver is macroscopically examined for pathological alterations, corresponding findings are (photo-) documented. If present, samples of altered tissue locations are taken for subsequent histopathological, molecular or microbiological analyses, as appropriate.

2.3.2 Liver

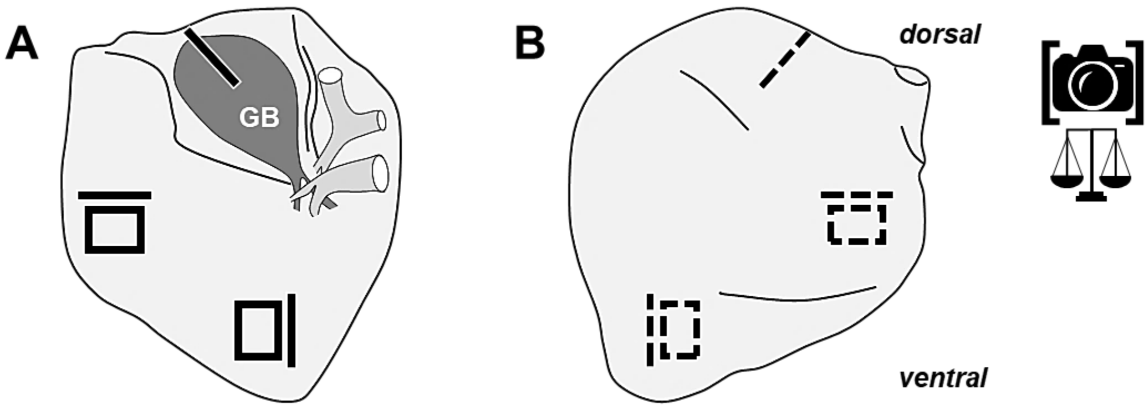

Figure 14. A schematic illustration of the visceral surface (A) and the parietal surface (B) of the liver with indicated sampling for routine histopathological and molecular analyses. Sample positions of molecular analysis samples (black rectangles) and sample locations and section plane orientations of samples for histopathological analyses (black lines) are indicated. The dotted lines/rectangles indicate the position of the identical sampling locations shown in **A**. **GB:** Gall bladder.

Sampling scheme for routine analyses of the rainbow trout liver

1. Samples for molecular analyses of liver tissue

Location:	Sampling locations are indicated in **Figures 14** and **15B**.
Number of samples:	Two.
Sample size:	Approximately 0.3 cm x 0.3 cm x 0.3 cm (length x width x height).
Remarks:	Homogenous specimens of liver parenchyma are taken.
Processing:	Samples are frozen (liquid nitrogen) and stored at -20°C or -80°C (short-term storage) depending on the intended analysis (chemical or molecular analysis). For prolonged storage of tissue samples for subsequent RNA- and protein analyses, storage at -150°C is recommended.
Downstream analyses:	DNA-, RNA-, protein-, and other OMICS profiling- or targeted analyses requiring cryo-conserved specimens as well as chemical (analytical) analyses.

2. Samples for histopathological examination of liver tissue

Location & orientation of sections:	Sampling locations and orientations are indicated in **Figures 14** and **15B**. One transverse and one horizontal sectional plane is generated, both containing liver capsule and parenchyma. Additionally, a longitudinal section through the gall bladder containing the gall bladder wall and the adjacent liver parenchyma is generated, as indicated in **Figure 15B**.
Number of samples:	Three.
Section plane size:	Approximately 0.5 cm x 0.5 cm (length x width).
Fixation & embedding:	FF-PE.

2.3.2 Liver

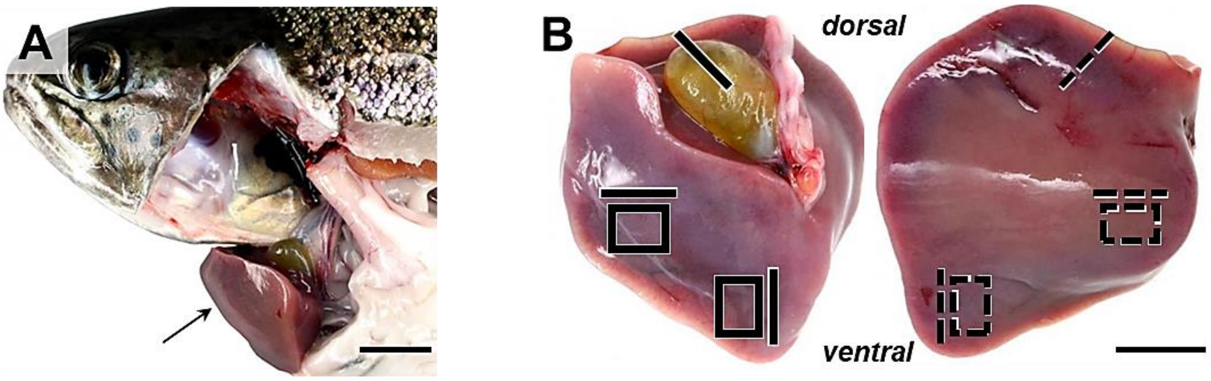

Figure 15. Sampling of standard liver specimens in routine ecotoxicological studies. A. Lateral (left) aspect of the necropsy situs after removal of the operculum and the gill basket, demonstrating the anatomical location of the liver (arrow). **B.** Visceral (left) and parietal (right) aspect of a freshly excised liver. Sampling locations and section plane orientations for histopathological and molecular analysis samples are indicated. Bar = 1 cm.

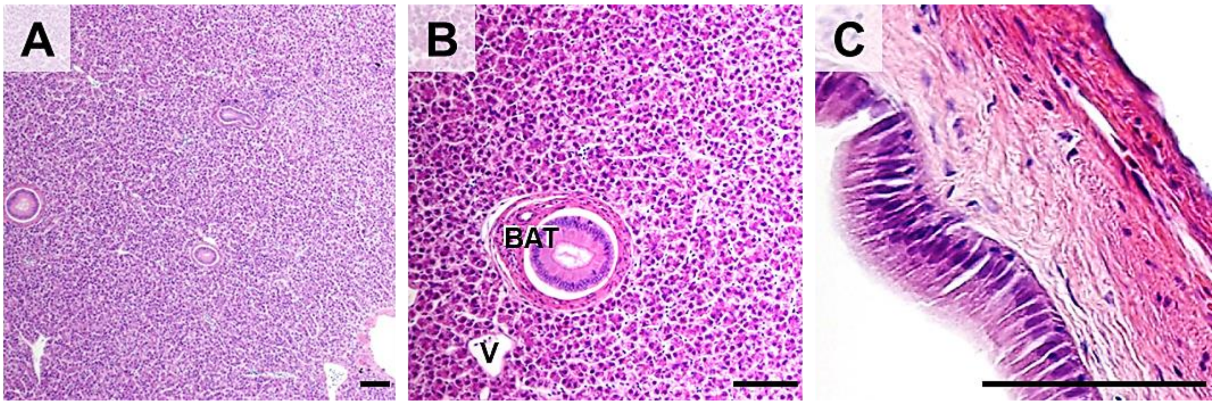

Figure 16. Liver and gall bladder histology. A. Liver parenchyma. Note that the trout liver displays no lobular organization pattern. **B.** Cross section of a biliary-arteriolar tract (**BAT**) and a venule (**V**). **C.** Gall bladder wall. The cells of the columnar epithelium are ciliated. Perfusion-fixed liver. FF-PE. HE. Bars = 100 µm.

Time and personal requirements

Approximately 5 minutes are to be scheduled for dissection, sampling and further processing of liver tissue samples for histopathological and molecular analyses. This estimate does not include the time needed for killing the fish, the prearrangement of sampling instruments and materials, or the further processing of fixed/frozen specimens. If vascular perfusion fixation is performed, at least additional 15 minutes should be scheduled.

A comparison of the proposed sampling scheme for routine analyses with previously published studies

Only few published ecotoxicological studies examining rainbow trout liver samples provide a specific description of the sampling locations, such as the anterior or posterior part of the liver [55, 56] or the liver tip [57]. In previously published ecotoxicological studies using rainbow trout, the number of analyzed liver samples per fish, if indicated, ranges between one (entire organ) [58] to four samples [59].

Considering the comparably homogenous composition of liver tissue, the numbers and sizes of liver samples as well as the sampling locations and section plane orientations proposed in the present guidelines are regarded to provide sufficiently representative specimen for the examination of the most (qualitative) morphological tissue alterations and molecular analyses of the rainbow trout liver.

2.3.3 Gastrointestinal tract

Relevant anatomical features/preparation

The multifunctional gastrointestinal tract (GIT) of the rainbow trout as a predatory species is relatively short and is composed of an esophagus, a stomach and the intestine. Compared to mammals, the digestive tract of teleost fish has a simple structure [1, 4]. The short esophagus ends in a thick-walled stomach, which is divided in cardia (non-secretory), fundus and pylorus (both secretory). The pyloric sphincter marks the transition of the stomach into the intestine [2]. The intestine can be divided into two (*i.e.,* the anterior small intestine and the posterior large intestine) [2, 36] or, somewhat more specifically, three different sections (*i.e.,* the anterior, mid and posterior intestine) (**Figure 17**) [60]. The anterior intestine (from the pyloric sphincter to the last pyloric cecum) possesses numerous blind-ending diverticula, *i.e.,* the pyloric ceca, which are unique to fish and have histological features identical to the ones of the anterior intestine [1, 2, 4, 60]. The mid intestine reaches from the most distal pyloric cecum to the beginning of the posterior intestine, which differs from the proximal gut by a darker color and annular rings (**Figure 18**). The posterior intestine ends at the urogenital papilla with a short rectum [2, 36, 60].

The GIT is removed after excision of the gills and the liver. During dissection of the gills, the abdominal cavity is opened by a transverse section in the ventral midline (the urogenital papilla remains intact) and the cranial aspect of the esophagus is cut immediately behind the transverse septum (**Figure 19A**, also compare to **Chapter 2.1**). After excision of the liver, the mesenterial attachment of the bowel is carefully severed without perforating the swim bladder, the vent-surrounding skin and muscle are severed and the GIT together with visceral adipose tissue, spleen and the urogenital papilla is removed from the peritoneal cavity (**Figure 19A**). If necessary, samples for histopathological adipose tissue analysis are generated from the visceral fat depot, subsequently spleen and visceral fat not containing pyloric ceca are separated (**Figure 19B**) and stored/sampled for further analysis, if required. Samples for histopathological and molecular analyses are taken from the unopened wall of the esophagus, stomach and intestine (**Figure 20**), adhering ingesta particles are removed by carefully swiveling the specimens in neutrally buffered 4% formaldehyde solution (specimens for histopathological analyses) or PBS buffer (specimens for molecular analyses). Subsequently, the digestive tube is longitudinally opened for macroscopic examination using scissors with rounded tips, if appropriate, samples of ingesta are appropriately stored and ingesta is carefully removed by rinsing the GIT with physiological saline or PBS buffer. After sampling and macroscopic examination, the remaining tissue is preserved (*i.e.,* immediately transferred to adequate fixative) to ensure that sufficient extra sample material is available for new/expanded scientific issues arising from the analyses.

2.3.3 Gastrointestinal tract

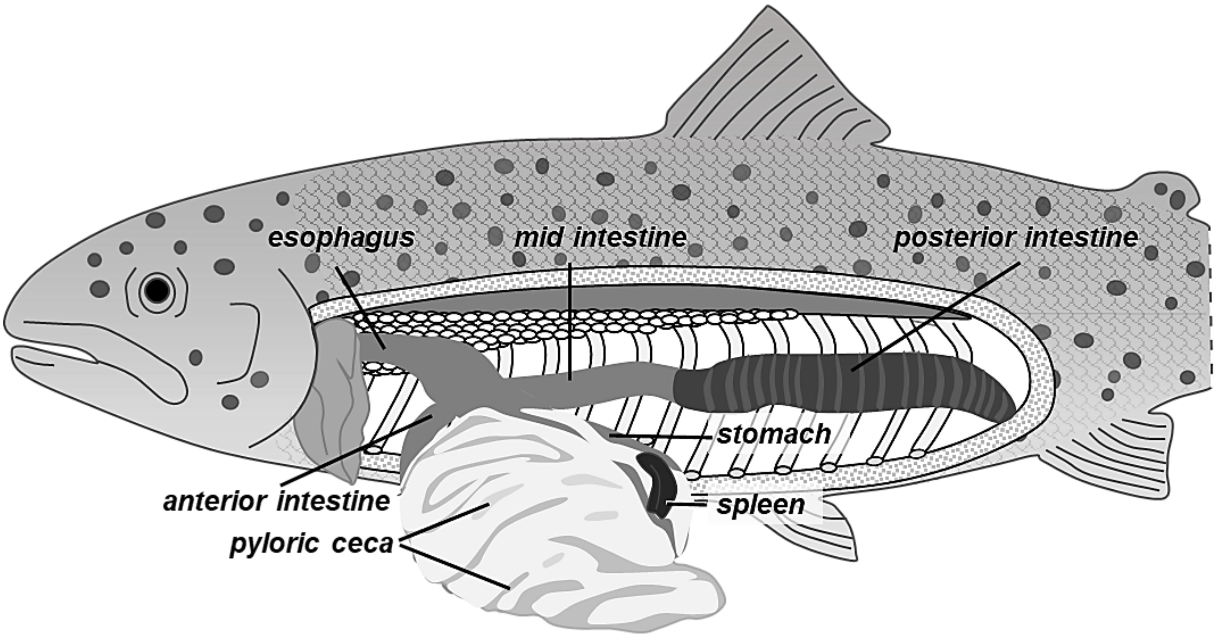

Figure 17. Schematic illustration of the gastrointestinal tract *in situ*. Lateral aspect of viscera of the rainbow trout after removal of the left abdominal wall.

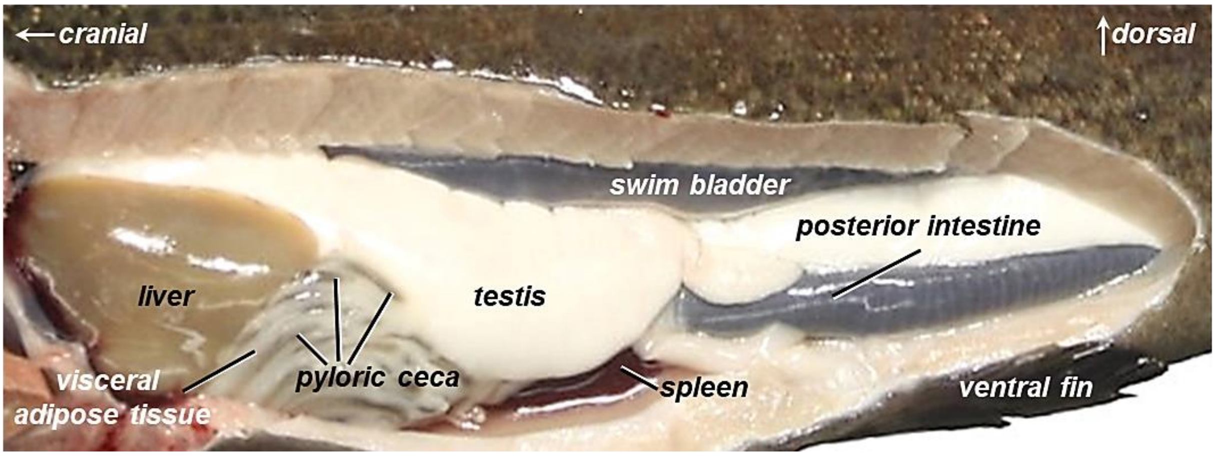

Figure 18. Photographic illustration of the viscera *in situ*. Lateral aspect of the viscera of an adult male rainbow trout after removal of the lateral (left) body wall. Pale color of the liver is due to a previous vascular perfusion fixation (not obligatory for analyses of the GIT). Note the dark color and the annular rings of the posterior intestine.

General examination parameters

Visceral fat and the spleen are removed from the freshly excised gastrointestinal tract and the GIT is briefly dabbed dry and the weight is recorded to the nearest mg. After samples of unopened stomach and intestinal parts are generated, the GIT is carefully opened using scissors (rounded tips). If no fasting period preceded the necropsy, remaining ingesta is gently removed rinsing the GIT with physiological saline or PBS buffer and GIT weight without ingesta is recorded, if necessary. The mucosal and serosal surface are macroscopically examined for pathological alterations and corresponding findings are (photo-) documented. If required, samples of altered gut tissue regions are generated for histopathological-, microbiological- or virological analyses.

2.3.3 Gastrointestinal tract

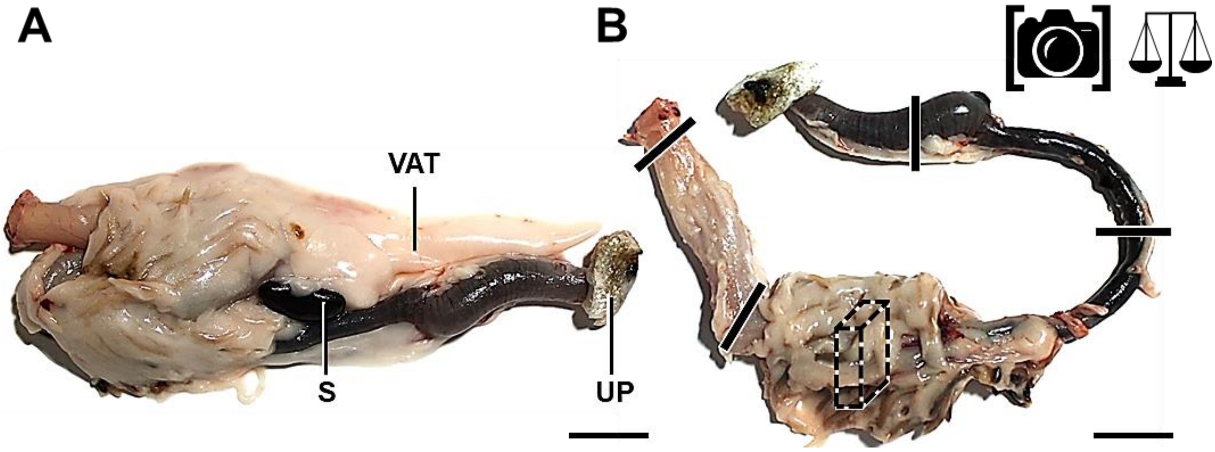

Figure 19. Photographic illustration of the excised gastrointestinal tract with indicated sampling locations for routine histopathological analyses. A. Unfixed gastrointestinal tract, freshly excised with attached visceral adipose tissue (**VAT**), spleen (**S**) and urogenital papilla (**UP**). **B.** Spleen and visceral adipose tissue not containing pyloric ceca are removed and the GIT is arranged at its full length for macroscopic examination and sampling of histopathological and molecular specimens (see **Figure 20**). Sampling locations and section plane orientations for histopathological analysis samples are indicated (black bars and black dotted box). Bars = 1 cm.

Sampling scheme for routine analyses of the gastrointestinal tract of the rainbow trout

1. Samples for molecular analyses of the gastrointestinal tract

Location:	Sampling locations are indicated in **Figure 20A**.
Number of samples:	Four.
Sample size:	Approximately 1 cm x 0.2 cm.
Remarks:	Circular samples are opened by scissors and carefully washed with PBS buffer.
Processing:	Samples are frozen (liquid nitrogen) and stored at -20°C or -80°C (short-term storage) depending on the intended analysis (chemical or molecular analysis). For prolonged storage of tissue samples for subsequent RNA- and protein analyses, storage at -150°C is recommended.
Downstream analyses:	DNA-, RNA-, protein-, and other OMICS profiling- or targeted analyses requiring cryo-conserved specimen as well as chemical (analytical) analyses.

2. Samples for histopathological examination of the gastrointestinal tract

Location & orientation of sections:	Sampling locations and orientations are indicated in **Figures 19B** and **20A**&**B**. Transverse sections of the distinct unopened gastrointestinal sections (*i.e.,* esophagus, stomach, pyloric ceca, mid intestine, posterior intestine) are generated:<br>**Esophagus**: ~0.5 cm behind the cranial esophagus transection line.<br>**Stomach**: ~1 cm proximal to the first pyloric cecum.<br>**Pyloric ceca:** Visceral adipose tissue containing pyloric ceca is cut transversely in the midline of the lateral aspect (transverse to the axis of the pyloric ceca).<br>**Mid intestine:** ~1-2 cm proximal to the beginning of the posterior intestine.<br>**Posterior intestine:** In the mid portion of the posterior intestine, ~2 cm proximal to the urogenital papilla.
Number of samples:	Five.
Section plane size:	All histopathological specimens are transverse sections of the entire bowel diameter, height of the circular specimens is ~0.2 cm. The sample containing pyloric ceca is ~1 cm x 1 cm x 0.2 cm (length x width x height). Section plane size of all samples is ~1 cm x 1 cm.
Fixation & embedding:	FF-PE.

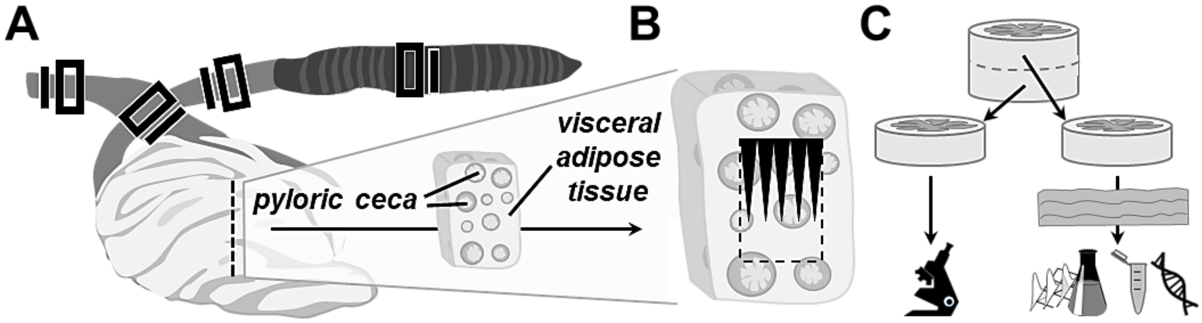

Figure 20. Sampling of gastrointestinal tract (GIT) tissue specimens in routine ecotoxicological studies. A. Lateral aspect of the freshly excised GIT, attached adipose tissue (not containing pyloric ceca) and spleen are removed. Sampling locations for histopathological and molecular analysis samples are indicated. **B.** For sampling of the pyloric ceca, a specimen of visceral adipose tissue containing pyloric ceca is cut out transversely as indicated in **A**&**B.** A histopathological sample of a size of ~1 cm x 1 cm x 0.2 cm is taken from the middle of the formation of pyloric ceca and adipose tissue. **C.** Of the four locations where both histopathological and molecular samples are taken, a cylindric gut sample of ~0.5 cm height is generated and separated in half by a cross section, using a sharp scalpel. One cylindric sample is processed for histopathological analyses, the other cylindric gut sample is opened using scissors, washed in PBS buffer and processed for subsequent molecular analyses.

2.3.3 Gastrointestinal tract

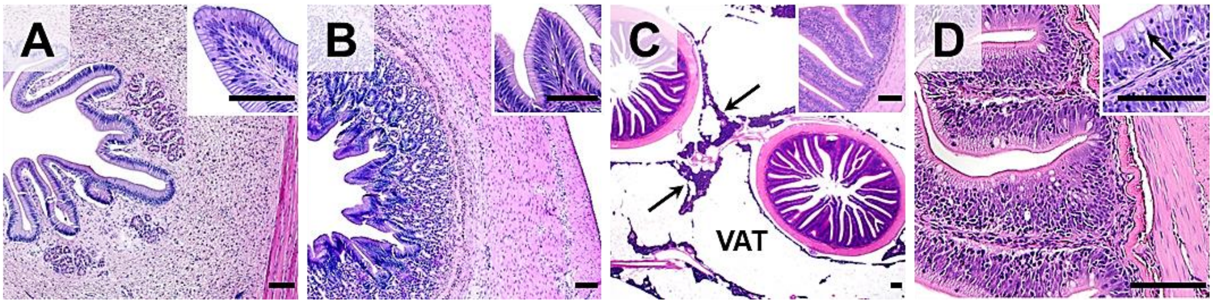

Figure 21. Gastrointestinal tract histology. A. Transverse section of esophagus wall. Inset: Esophagus mucosa. The esophagus is the only section of the digestive tract (besides the anal sphincter) of the GIT, where striated muscle is present. **B.** Transverse section of stomach wall. Note the prominent tunica muscularis and the gastric glands in the lamina propria mucosae. Inset: Gastric mucosa. **C.** Transverse sections of pyloric ceca, embedded in visceral adipose tissue (**VAT**). Between the pyloric ceca, there is pancreatic tissue randomly dispersed in the adipose tissue (arrows). Inset: Villi of pyloric ceca. Pyloric ceca are evaginations of the anterior intestine from which they differ in their smaller diameters, but not in their histoarchitecture. **D.** Transverse section of the posterior intestine. The mucosa contains numerous goblet cells (arrow). Inset: Posterior gut mucosa. FF-PE. HE. Bars = 100 µm.

Time requirements

Approximately 10 minutes are to be scheduled for dissection of the GIT, macroscopic examination, sampling and further processing of GIT tissue samples. This estimate does not include the time needed for killing the fish, dissection of gills/liver, prearrangement of sampling instruments and materials, or the further processing of fixed/frozen specimens.

A comparison of the proposed sampling scheme for routine analyses with previously published studies

Previous published ecotoxicological studies that specify the sampling procedure of rainbow trout gastrointestinal tract samples in more detail proceed according to varying sampling protocols. Either the small intestine is sampled [56], a ~8 cm section of the posterior intestine is sampled [61] or the three intestine sections and the stomach are sampled and analyzed separately [60, 62, 63]. In previous ecotoxicological studies examining rainbow trout, the number of analyzed GIT tissue samples per fish, if indicated, ranges between one [56, 61] to four [60, 62] samples.

Related to the size of the GIT and the histopathological composition of the different intestinal sections, the number and sizes of GIT tissue samples, as well as the sampling locations proposed in the present guidelines are regarded to provide sufficiently representative GIT tissue specimens for examination of most qualitative GIT tissue alterations and molecular analyses of trout GIT tissue.

2.3.4 Pancreas (Exocrine and endocrine pancreas)

Relevant anatomical features/preparation

The teleost pancreas comprises both exocrine and endocrine tissue. The exocrine pancreas cells secrete digestive enzymes (*e.g.,* proteases, lipase or amylase) and bicarbonates, the different cell types of the endocrine pancreatic tissue secrete glucagon, insulin, somatostatin and pancreatic polypeptide [2, 25]. Other than in mammals, the rainbow trout`s pancreas is grossly not clearly delimited from the surrounding visceral adipose tissue. The pancreatic tissue is diffusely scattered through the peritoneal cavity and is located within the mesentery, associated with the gastrointestinal tract, blood vessels, liver and gall bladder. In trout, it is mainly located within the adipose tissue surrounding the pyloric ceca [1, 2, 4, 36]. As an accessory organ of the gastrointestinal tract (GIT), the exocrine pancreas is connected to the proximal intestine and pyloric ceca via pancreatic ducts [1, 2, 36]. The endocrine pancreatic tissue is organized in one compact islet organ (*i.e.,* the Brockmann body), an aggregation of endocrine pancreatic islands in a specific location of the exocrine pancreatic tissue nearby the gall bladder (**Figure 23A**&**B**), and several smaller islets (*i.e.,* the Langerhans islets) scattered through the exocrine pancreas (**Figure 23C**) [25, 42, 64, 65]. In larger rainbow trout, the islet organ is subdivided in several smaller islets by exocrine pancreatic tissue [64].
At necropsy, the pancreatic tissue is removed from the abdominal cavity together with the GIT, the visceral adipose tissue and the spleen after excision of gills and liver. The abdominal cavity is opened by a transverse section in the ventral midline, the GIT is excised by severing the cranial portion of the esophagus and cutting out the vent (urogenital papilla). Spleen and visceral fat not containing pyloric ceca are separated and stored/sampled for analysis, if required. After sampling of the visceral adipose tissue with the diffusely distributed pancreas (**Figure 24**) and storage of the specimens for histopathological pancreas analysis in neutrally buffered 4% formaldehyde solution, the remaining adipose tissue and the GIT are preserved (*i.e.,* immediately transferred to an adequate fixative) to ensure that sufficient sample material is available for new/expanded scientific issues arising from the analyses. The Brockmann body is excised and adhering adipose tissue, connective tissue or blood vessels are carefully removed before freezing for subsequent molecular analyses.

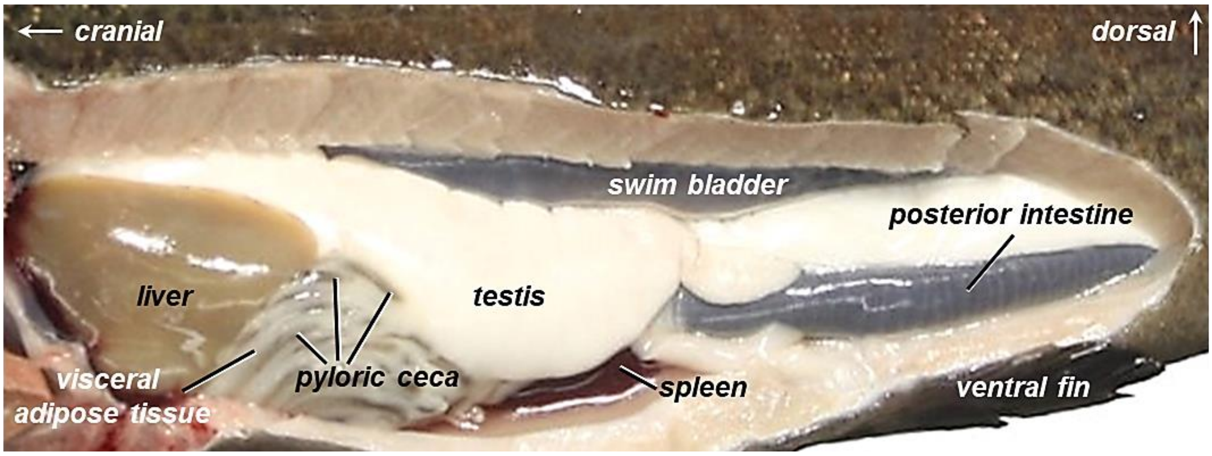

Figure 22. Photographic illustration of the viscera of the rainbow trout *in situ*. Lateral aspect of the viscera of an adult male rainbow trout after removal of the left lateral body wall. The pale color of the liver is due to a previous vascular perfusion fixation (not obligatory for analysis of the pancreatic tissue). The pancreas is diffusely scattered through the mesentery (mainly through the visceral adipose tissue surrounding the pyloric ceca) and not macroscopically visible.

General examination parameters

Gross findings in the visceral adipose tissue indicating pancreatic tissue involvement (*e.g.,* petechiae) may require (photo-) documentation and sampling of altered tissue regions for histopathology, microbiology or virology. The weight of the dissected principal island is recorded and the islet organ is macroscopically examined for pathological alterations, corresponding findings are (photo-) documented. Samples are taken for subsequent histopathological, molecular or microbiological analysis, if required.

Sampling scheme for routine analyses of rainbow trout pancreas

1. Sample for molecular analyses of the pancreas

Location:	Sampling location is indicated in **Figure 23A**&**B**.
Number of samples:	One.
Sample size:	Entire islet organ.
Remarks:	Oval, white-pink compact organ within the mesentery, located nearby the gall bladder. Adhering adipose tissue, connective tissue and blood vessels are removed.
Processing:	The sample is frozen (liquid nitrogen) and stored at -20°C or -80°C (short-term storage) depending on the intended analysis (chemical or molecular analysis). For prolonged storage of tissue sample for subsequent RNA- and protein analyses, storage at -150°C is recommended.
Downstream analyses:	DNA-, RNA-, protein-, and other OMICS profiling- or targeted analyses requiring cryo-conserved specimen as well as chemical (analytical) analyses.

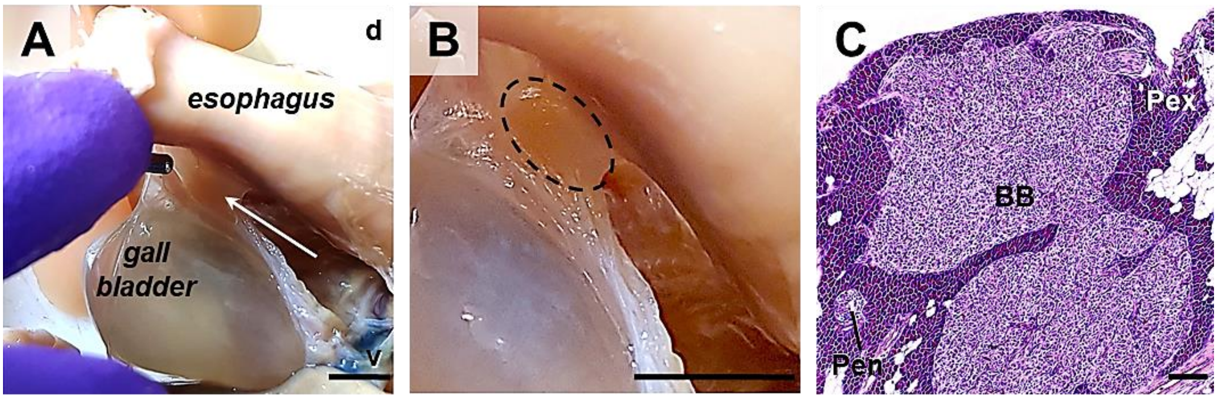

Figure 23. Photographic illustration of the *in situ* location and the histopathology of the Brockmann body. A. Lateral aspect of the dissected and formalin-fixed gall bladder and the esophagus. The Brockmann body is macroscopically visible within the mesentery (arrow). Note that the purpose of this figure is only to demonstrate the *in situ* location of the Brockmann body. For molecular analyses, a fresh and unfixed specimen must be generated. **v:** Ventral; **d:** Dorsal. Bar = 1 cm. **B.** Detail enlargement of the Brockmann body, encircled by the black dashed line. Bar = 1 cm. **C.** Histology of the Brockmann body. The islet organ (*i.e.,* the Brockmann body (**BB**)) is surrounded by exocrine pancreatic tissue (**Pex**). Note the size difference between the islet organ and an islet of Langerhans (**Pen**). FF-PE. HE. Bar = 100 µm.

2. Samples for histopathological examination of the pancreas

Location & orientation of sections:	Sampling locations and orientations are indicated in **Figure 24**. The anterior and posterior part of the lateral aspect of the visceral adipose tissue containing pyloric ceca and pancreatic tissue (exocrine and endocrine) is cut transversely.
Number of samples:	Two.
Section plane size:	The samples of the visceral adipose tissue surrounding the pyloric ceca and containing pancreatic tissue are ~1 cm x 1 cm x 0.2 cm (length x width x height). Section plane size is ~1 cm x 1 cm (length x width).
Fixation & embedding:	FF-PE.

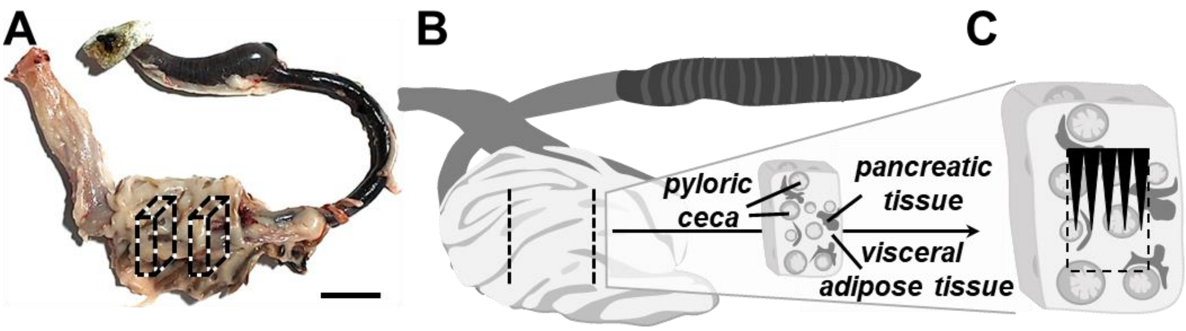

Figure 24. Sampling of pancreatic tissue specimen for histopathological analysis in routine ecotoxicological studies. A. Freshly excised GIT and visceral adipose tissue containing pancreas and pyloric ceca, arranged for macroscopic examination and sampling of histopathological specimens. Sampling locations for histopathological analysis samples are indicated (black dashed boxes). Bar = 1 cm. **B.** Schematic illustration of the lateral aspect of the excised gastrointestinal tract. Attached visceral adipose tissue and spleen are removed. Sampling locations for histopathological analysis samples are indicated (black dashed lines). **C.** For sampling of the exocrine and endocrine pancreas, two specimens of are cut out transversely from the cranial and caudal parts of the formation of pyloric ceca and visceral adipose tissue containing pancreatic tissue.

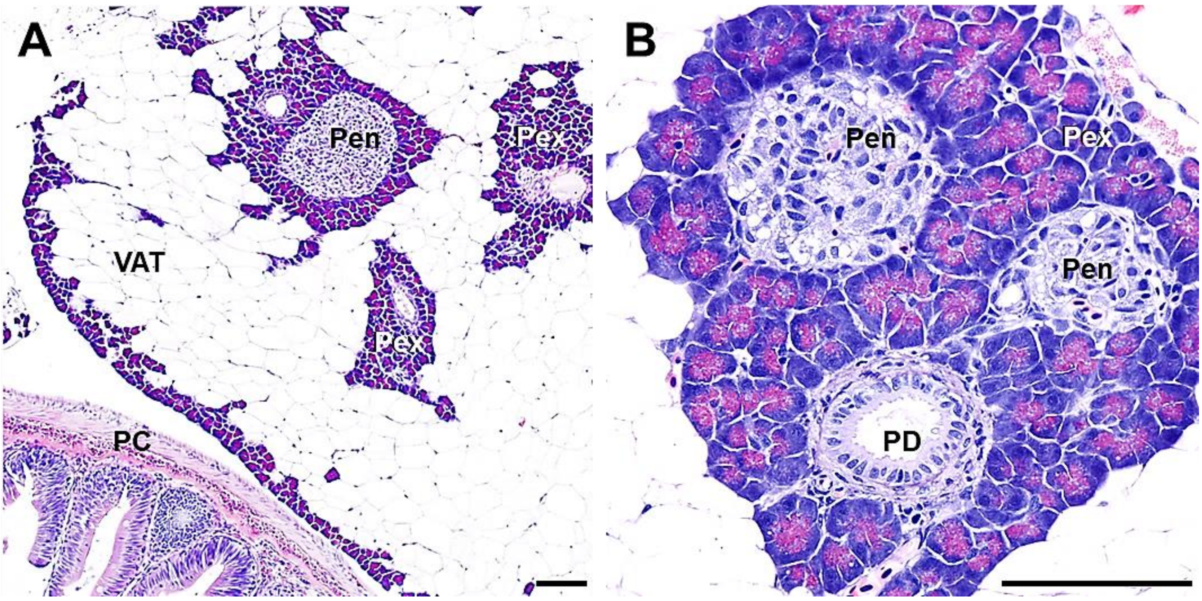

Figure 25. Histology of the endocrine and exocrine pancreas. A. Transverse sections of visceral adipose tissue (**VAT**), containing pyloric ceca (**PC**) and randomly dispersed exocrine (**Pex**) and endocrine (**Pen**) pancreatic tissue. **B.** Histology of the endocrine pancreas acini with an associated pancreatic duct (**PD**) and islets of Langerhans. FF-PE. HE. Bars = 100 µm.

Time requirements

Approximately 5 minutes are to be scheduled for excision, macroscopic examination and further processing of the of the Brockmann bodies for molecular analyses. Another 3-5 minutes are to be scheduled for the dissection of the GIT with attached spleen and adipose tissue containing pancreatic tissue, macroscopic examination of the visceral adipose tissue, sampling and further processing of the pancreas-containing tissue samples. This estimate does not include the time needed for killing the fish, dissection of gills/liver, prearrangement of sampling instruments and materials, or the further processing of fixed/frozen specimens.

A comparison of the proposed sampling scheme for routine analyses with previously published studies

To the authors knowledge, previously performed ecotoxicological studies examining salmonids do not precisely indicate the number or sampling location of analyzed pancreatic tissue specimens per fish, and the rainbow trout pancreatic tissue is the object of only few ecotoxicological studies examining the effects of environmental pollutants [66-68]. In other non-ecotoxicological studies examining the pancreas of rainbow trout, either the Brockmann bodies are removed for subsequent analyses of the endocrine pancreas [69-71] or pancreatic tissue located within the visceral adipose tissue is sampled [72-74].
Considering that the piscine pancreas (containing both exocrine and endocrine components) is scattered through the abdominal cavity within the mesentery and that there is an incorporation of endocrine and exocrine pancreatic tissue in one compact organ (*i.e.,* Brockmann bodies), the number and sizes of pancreatic tissue samples as well as the sampling locations proposed in the present guidelines are regarded to provide sufficiently representative tissue specimens for the examination of the most (qualitative) morphological exocrine and endocrine pancreatic tissue alterations and molecular/chemical analyses of trout`s pancreas. If only the endocrine pancreatic tissue is to be examined, the interested reader is referred to **Chapter 2.13.2** where a detailed protocol for the sampling for histopathological (and molecular) analyses of the endocrine pancreatic tissue is illustrated.

2.3.5 Swim bladder

Relevant anatomical features/preparation

Compared to other fish species, the salmonid swim bladder (also called gas bladder or air bladder) is a simple organ comprised of a single chamber. The rainbow trout`s swim bladder is an elongated, transparent, sac-like structure situated retroperitoneal and dorsal to the gastrointestinal tract (**Figure 26**) [1, 2, 4, 42]. The swim bladder is a derivate of the alimentary tract. In rainbow trout, the connection of the swim bladder and the esophagus is retained as the pneumatic duct. Therefore, the rainbow trout is referred to as a physostome [1, 2, 36, 75]. The rainbow trout`s swim bladder is an important hydrostatic organ involved in buoyancy adjustment [2, 4, 75].

At necropsy, the swim bladder is removed from the abdominal cavity after excision of the gills, the liver and the GIT (together with the visceral adipose tissue and the spleen). For removal, the cranial end of the pneumatic duct is grasped with forceps and the swim bladder is gently pulled in caudo-ventral direction. Depending on gonadal size, the ventro-laterally attached gonads are either removed together with the swim bladder (compare to **Figure 26B**&**C**) or (*e.g.,* during spawning season) the gonads are carefully separated from the swim bladder and removed from the peritoneal cavity in advance (refer to **Chapter 2.6**). If no fresh (*i.e.,* unfixed) tissue samples for molecular analyses are required, neutrally buffered 4% formaldehyde solution is injected into the lumen before immersion fixation for enhanced tissue fixation (**Figure 26B**).

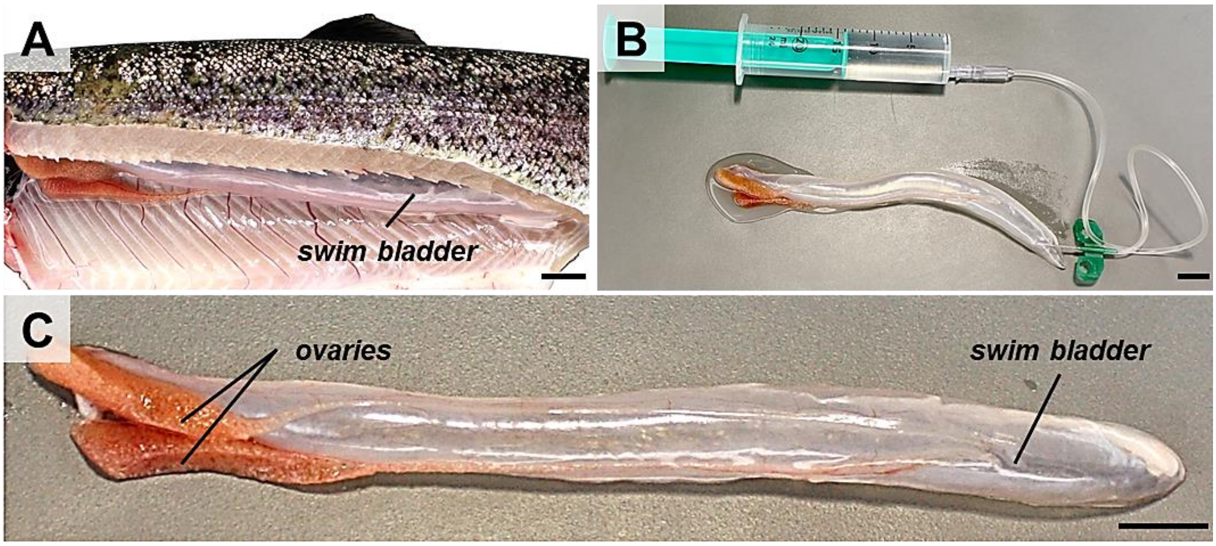

Figure 26. Photographic illustration of the swim bladder of the rainbow trout. A. Ventro-lateral aspect of the swim bladder and attached gonads (as *in situ)* after removal of the left lateral body wall, liver, and gastrointestinal tract (GIT) together with spleen and visceral adipose tissue. **B, C.** Dissected swim bladder with ventro-laterally attached ovaries. The swim bladder is dissected after excision of the other viscera by grasping the anterior end of the pneumatic duct using forceps and gently pulling in caudo-ventral direction. If no fresh (*i.e.,* unfixed) tissue samples are required, neutrally buffered 4% formaldehyde solution is carefully injected into the lumen before immersion fixation. Bars = 1 cm.

General examination parameters

After excision, attached gonads as well as adherent adipose- and connective tissue are carefully removed and the swim bladder is briefly dabbed dry with a laboratory paper towel. Subsequently, the swim bladder weight is recorded to the nearest mg. The swim bladder is macroscopically examined for pathological alterations, corresponding findings are (photo-) documented. If present, samples of altered tissue locations are taken for subsequent histopathological, molecular or microbiological analysis, as appropriate.

Sampling scheme for routine analyses of the rainbow trout swim bladder

1. Samples for molecular analyses of the swim bladder

Location:	Sampling locations are indicated in **Figure 27A**.
Number of samples:	Two.
Sample size:	Ring-shaped strips, height ~0.3 cm.
Remarks:	Homogenous specimens of swim bladder tissue are taken, ~2 cm apart from the anterior and posterior end of the swim bladder.
Processing:	Samples are frozen (liquid nitrogen) and stored at -20°C or -80°C (short-term storage) depending on the intended analysis (chemical or molecular analysis). For prolonged storage of tissue samples for subsequent RNA- and protein analyses, storage at -150°C is recommended.
Downstream analyses:	DNA-, RNA-, protein-, and other OMICS profiling- or targeted analyses requiring cryo-conserved specimen as well as chemical (analytical) analyses.

2. Samples for histopathological examination of the swim bladder

Location & orientation of sections:	Sampling locations and orientations are indicated in **Figure 27A**. Transversally oriented circular specimens of the swim bladder are taken, each with ~2 cm distance to the anterior and posterior end of the swim bladder and directly cranial to the sample site of the specimens for molecular analyses. For an enhanced presentation of the histopathology, the specimens are embedded in agar after immersion fixation to maintain the position of the swim bladder wall during further processing for paraffin embedding (**Figure 28A**).
Number of samples:	Two.
Section plane size:	Circular specimen, including the entire swim bladder wall diameter, height ~0.3 cm.
Fixation & embedding:	FF-PE.

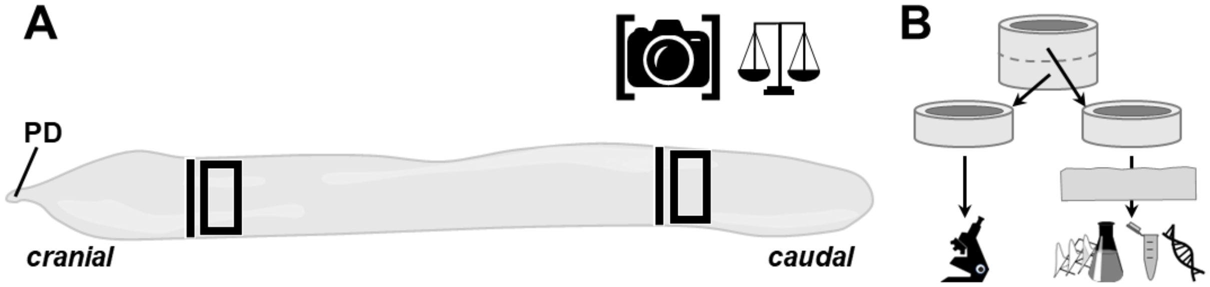

Figure 27. Schematic illustration of the sampling of the swim bladder for routine histopathological and molecular analyses. A. Schematic illustration of the swim bladder. Sample positions of molecular analysis specimens (black rectangles) and sample locations and section plane orientations of specimens for histopathological analyses (black lines) are indicated. **PD:** Pneumatic duct. **B.** Generation of the specimens for histopathological and molecular analyses. Both histopathological and molecular samples are taken from the same two sampling locations, ring-shaped tissue samples are excised using a microtome blade. One sample is processed for histopathological analyses, the other cylindrical specimen is opened using scissors and adequately processed for molecular analyses.

2.3.5 Swim bladder

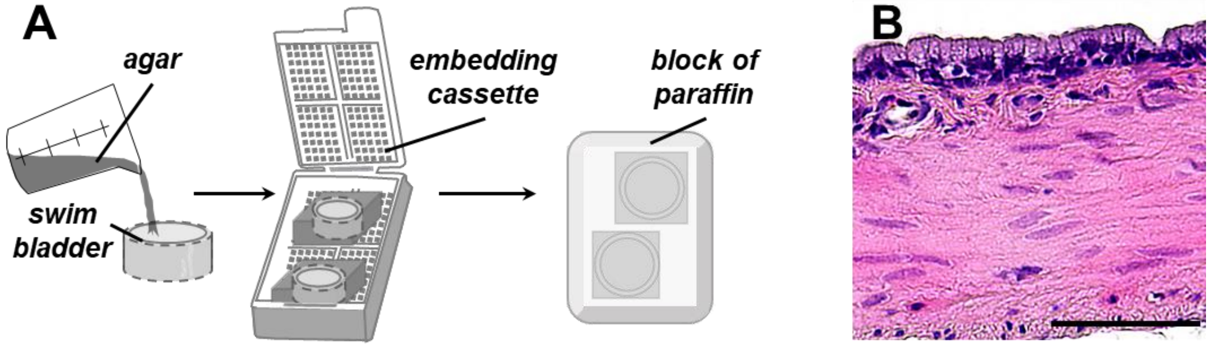

Figure 28. Histology of the rainbow trout`s swim bladder. A. Immersion fixation and embedding of histopathological swim bladder specimens. For the embedding of the histopathological specimens in paraffin, the position and orientation of the swim bladder wall is maintained by embedding the ring-shaped specimens in agar in advance. **B.** Swim bladder wall histology. Cross section of the swim bladder wall. FF-PE. HE. Bar = 50µm.

Time requirements

Approximately 2-3 minutes are to be scheduled for dissection, macroscopic examination/weighing, sampling and further processing of swim bladder tissue sample. This estimate does not include the time needed for killing the fish, dissection of gills and viscera, prearrangement of sampling instruments and materials, or the further processing of fixed/frozen specimens.

A comparison of the proposed sampling scheme for routine analyses with previously published studies

According to the authors knowledge, the explicit examination of trout swim bladder tissue has not yet been strongly highlighted in ecotoxicological studies and also previously performed carcinogenicity studies examining the carcinogenic potential of different chemicals on the rainbow trout swim bladder tissue do not provide precise information regarding the number and location of the analyzed swim bladder specimens [76].
Considering the comparably homogenous composition of swim bladder tissue, the numbers and sizes of the swim bladder samples as well as the sampling locations proposed in the present guidelines are regarded to provide sufficiently representative specimen for examination of most (qualitative) morphological swim bladder tissue alterations and molecular analyses of the swim bladder.

2.4 Adipose tissue

Relevant anatomical features/preparation

The adipose tissue of the poikilotherm rainbow trout is stored in several depots. The visceral adipose tissue (VAT) is located in the peritoneal cavity and surrounds the gastrointestinal tract (**Figures 29A** and **30**). The subcutaneous adipose tissue (scAT) is especially prominent in the dorsal region cranial to the dorsal fin (**Figure 29B**) and in the abdominal wall ventral to the fish bones, as part of the belly flaps [77-80]. Additionally, lipids are also stored in the liver and the red and white musculature [78, 80, 81]. The fat content of the different adipose tissue depots of teleosts may vary depending on the fish species, age, season or diet (the proportion of the VAT in the total body weight can range from 2 to 25%) [80, 82-84]. The several fat depots differ in their lipid composition as well as in their function [78-80]. The VAT tissue nearly completely consists of (unilocular, white) adipocytes while intramuscular fat comprises adipocytes as well as intracellular lipid droplets within the muscle cells [78, 80, 85, 86]. VAT as primary fat depot has a high lipid storage capacity and is therefore suitable for long-term storage of lipids; during starvation situations, lipids are mobilized primarily from the VAT [78, 82]. Next to energy storage, the VAT with its resident leucocyte population is also of immunological function [87].
At necropsy, the VAT is removed from the abdominal cavity together with the gastrointestinal tract (GIT) and the spleen after excision of the gills and liver. After removal, macroscopic examination and (photo-) documentation, the samples for histopathological and molecular analyses are generated. The sample for subsequent molecular analyses is excised from the most caudal part of the fresh (*i.e.,* unfixed) VAT. The sample for histopathological analyses is generated from the posterior part of the VAT (**Figures 29A** and **30B**). A further histopathological sample of the scAT is generated from the dorsal trunk region between the head and the dorsal fin (**Figure 29**) together with skin and the underlying epaxial skeletal musculature. After sampling and storage of the samples for histopathological adipose tissue analyses in neutrally buffered 4% formaldehyde solution, remaining adipose tissue and GIT are preserved (*i.e.,* immediately transferred to an adequate fixative) to ensure that sufficient sample material is available for new/expanded scientific issues arising from the analyses.

General examination parameters

The VAT is briefly dabbed dry with a laboratory paper towel before macroscopic examination. The VAT as well as the scAT tissue visible at the cutting surfaces is examined for pathological alterations and corresponding findings are (photo-) documented. If present, samples of altered tissue locations are taken for subsequent histopathological, molecular or microbiological analyses, as appropriate.

2.4 Adipose tissue

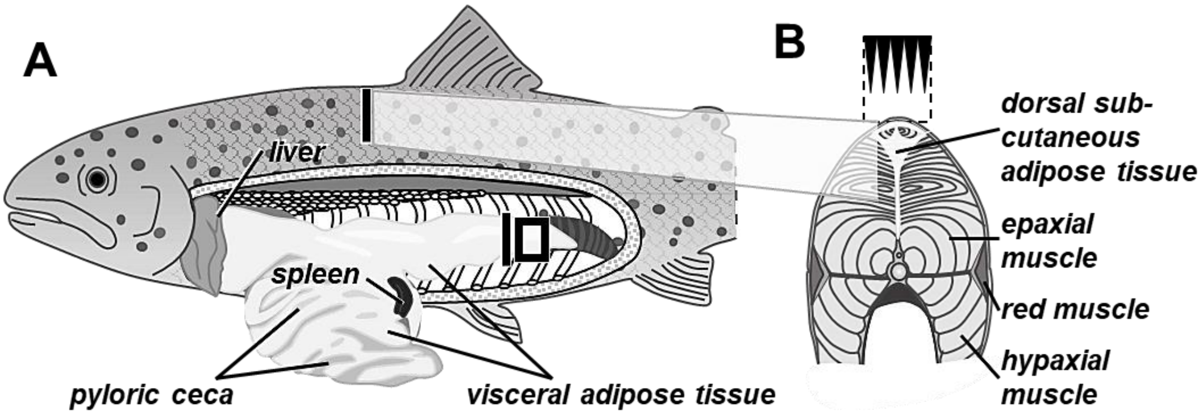

Figure 29. Schematic illustration of the sampling for histopathological and molecular analyses of the adipose tissue of the rainbow trout. A. Lateral aspect of the VAT and embedded viscera *in situ,* after removal of the left body wall. The sample position of the molecular analysis sample (black rectangle) as well as sample locations and section plane orientations of the samples for histopathological analyses (black lines) are indicated. The sample for molecular analyses is excised after removal of the gills and excision of the liver and the GIT (together with the spleen and VAT) from the peritoneal cavity. For sampling of the caudal portion of the VAT not containing pyloric ceca, a specimen of VAT is cut out transversely after removal of the rearmost 0.5 cm. Samples for histopathological adipose tissue analyses are generated after immersion fixation, the sample of the VAT is cut transversely from the caudal portion of the mesenterial fat not containing pyloric ceca directly cranial to the sample location of the specimen for molecular analyses. **B.** Schematic illustration of the dorsal aspect of the trunk cross section ~1 cm cranial to the dorsal fin, containing the dorsal scAT depot. The sampling location and section plane orientation of the histopathological analysis sample is indicated (black dotted rectangle with triangles, representing the cutting level parallel to the picture plane). A block of skin, dorsal scAT and underlying epaxial skeletal musculature is excised and a transversal section plane is generated.

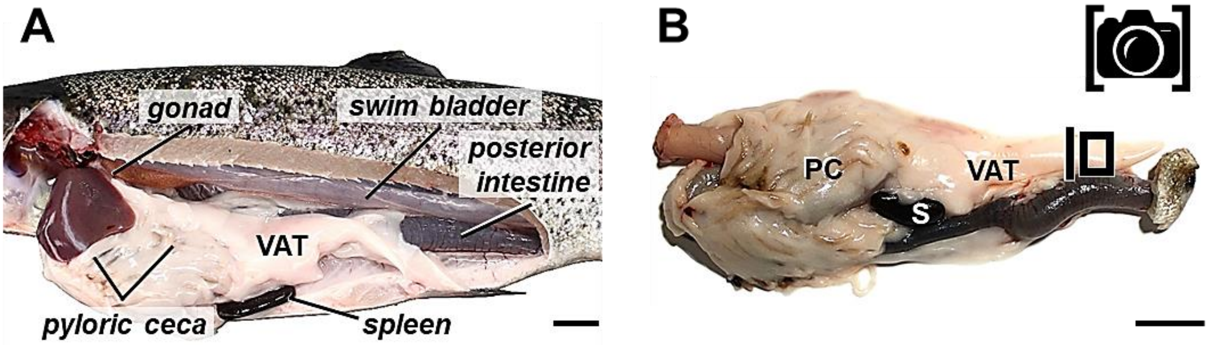

Figure 30. Photographic illustration of sampling of the visceral adipose tissue of the rainbow trout. A. Lateral aspect of the VAT and viscera *in situ*. **B.** Lateral aspect of the freshly excised visceral adipose tissue (**VAT**) together with the embedded gastro-intestinal tract (the pyloric ceca are indicated (**PC**)) and the spleen (**S**). The position and section plane orientation of the samples for molecular and histopathological analyses of the VAT are indicated, the samples are generated from the most caudal portion of the mesenterial fat. The rearmost portion (~0.5 cm) of the fresh (*i.e.,* unfixed) VAT is removed by a transverse incision and a homogenous VAT specimen is obtained by a second transverse section ~1 cm cranial to the first section for molecular analyses. The sample for histopathological analyses of the VAT is generated directly cranial to the sample location of the specimen for molecular analyses, a tissue block of approximately 0.5 cm thickness is cut transversely from the caudal portion of the mesenterial adipose tissue not containing pyloric ceca. Bars = 1 cm.

Sampling scheme for routine analyses of rainbow trout adipose tissue

1. Sample for molecular analyses of the adipose tissue

Location:	The sample location is illustrated in **Figures 29A** and **30B**. The adipose tissue sample is cut from the caudal portion of the VAT, ~0.5 cm cranial to the posterior end of the visceral fat.
Number of samples:	One.
Sample size:	Approximately 1 cm x 0.7 cm x 0.3 cm (length x width x height).
Remarks:	A homogeneous adipose tissue sample not containing any pyloric ceca is cut from the VAT.
Processing:	The sample is frozen (liquid nitrogen) and stored at -20°C or -80°C (short-term storage) depending on the intended analysis (chemical or molecular analysis). For prolonged storage of the tissue sample for subsequent RNA- and protein analyses, storage at -150°C is recommended.
Downstream analyses:	DNA-, RNA-, protein-, and other OMICS profiling- or targeted analyses requiring cryo-conserved specimen as well as chemical (analytical) analyses.

2. Samples for histopathological examination of the adipose tissue

Location & orientation of sections:	Sampling locations and orientations are indicated in **Figures 29** and **30B**. For histopathological examination, the VAT is sampled immediately cranial to the sample site for molecular analyses. The posterior part of the VAT is cut transversely over the full width. For histopathological examination of the scAT, a transverse section of the dorsal depot (with overlying skin and surrounding musculature) is generated.
Number of samples:	Two.
Section plane size:	VAT: Approximately 1 cm x 0.3 cm (length x width).<br>scAT: Approximately 1 cm x 1 cm (length x width).
Fixation & embedding:	FF-PE.

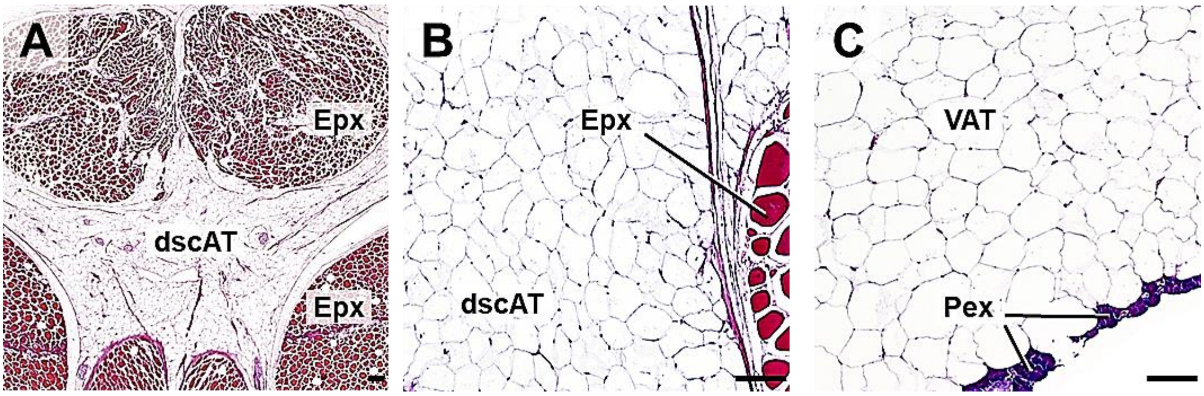

Figure 31. Histology of the rainbow trout adipose tissue. A. Transverse section of the dorsal subcutaneous adipose tissue depot (**dscAT**). The abundant dorsal adipose tissue is located around the transversal septum and adjacent to the white epaxial musculature (**Epx**). **B.** Detail enlargement of the dorsal subcutaneous tissue depot (**dscAT**). Epaxial skeletal musculature (**Epx**) is visible at the lower right corner. **C.** Transverse section of the visceral adipose tissue depot (**VAT**). Exocrine pancreatic tissue (**Pex**) is distributed within the VAT. FF-PE. HE. Bars = 100 µm.

Time requirements

3-5 minutes are to be scheduled for dissection, macroscopic examination, sampling and further processing of the rainbow trout adipose tissue samples for histopathological and molecular analyses. This estimate does not include the time needed for killing the fish, dissection of gills and liver, prearrangement of sampling instruments and materials, or the further processing of fixed/frozen specimens.

A comparison of the proposed sampling scheme for routine analyses with previously published studies

Previously published ecotoxicological studies examining trout adipose tissue either sample the VAT depot [7, 88] or sample subcutaneous, intramuscular or intrahepatic lipids together with the tissues/organs storing them [89-92].
According to the authors knowledge, the explicit examination of trout adipose tissue has not yet been strongly highlighted in ecotoxicological studies. Nevertheless, these guidelines specify the sampling of adipose tissue for molecular and histopathological routine analyses, since lipophilic contaminants taken up from the environment may be stored in the adipose tissue (and be released in the course of *e.g.,* gonadal development or mobilization of energy reserves, as fat is the predominant energy source for fish) [80, 93, 94]. Histopathological examination should be performed, since it is a valuable tool for the identification of morphological adipose tissue alterations that may be present. The locations and orientations of the samples for histopathological analyses of the rainbow trout adipose tissue are chosen so that both adipose tissue depots (scAT and VAT) are represented in the histological sections. The sample location of the adipose tissue specimens for molecular analyses is selected to ensure that pure adipose tissue without adherent muscle tissue or intestinal components is available for examination. The number and sizes of tissue samples as well as the sampling locations proposed in the present guidelines are regarded to provide sufficiently representative tissue specimens for the examination of the most (qualitative) morphological tissue alterations and molecular analyses of the rainbow trout adipose tissue.

2.5 Spleen

Relevant anatomical features/preparation

The rainbow trout`s spleen is situated in the left lower abdomen, attached to (and often embedded in) the visceral adipose tissue (**Figure 32**). It is a soft, dark-red, compact organ whose size can vary due to antigen exposure (*e.g.,* bacterial or viral infections) or changes in the splenic erythrocyte reservoir volume (*e.g.,* due to stress) [2, 95]. In rainbow trout, additional smaller accessory spleens may be present [4]. In physiological state, the spleen margins are sharp-edged [3]. In teleost fish, the spleen functions as a hematopoietic organ (*i.e.,* site of erythropoiesis) and erythrocyte reservoir [2, 36, 42]. In rainbow trout, the spleen also has immunological function by acting as an "antigen trap" and by removing aged or infected blood cells by phagocytosis [2-4]. A thin, fibrous capsule is surrounding the spleen matrix composed of the red and white pulp, which are not as distinctly separated as in mammals [2, 36, 42]. A distinct feature of the spleen of teleost fish is the melanomacrophage centers (MMC), typically located next to splenic blood vessels [2-4].

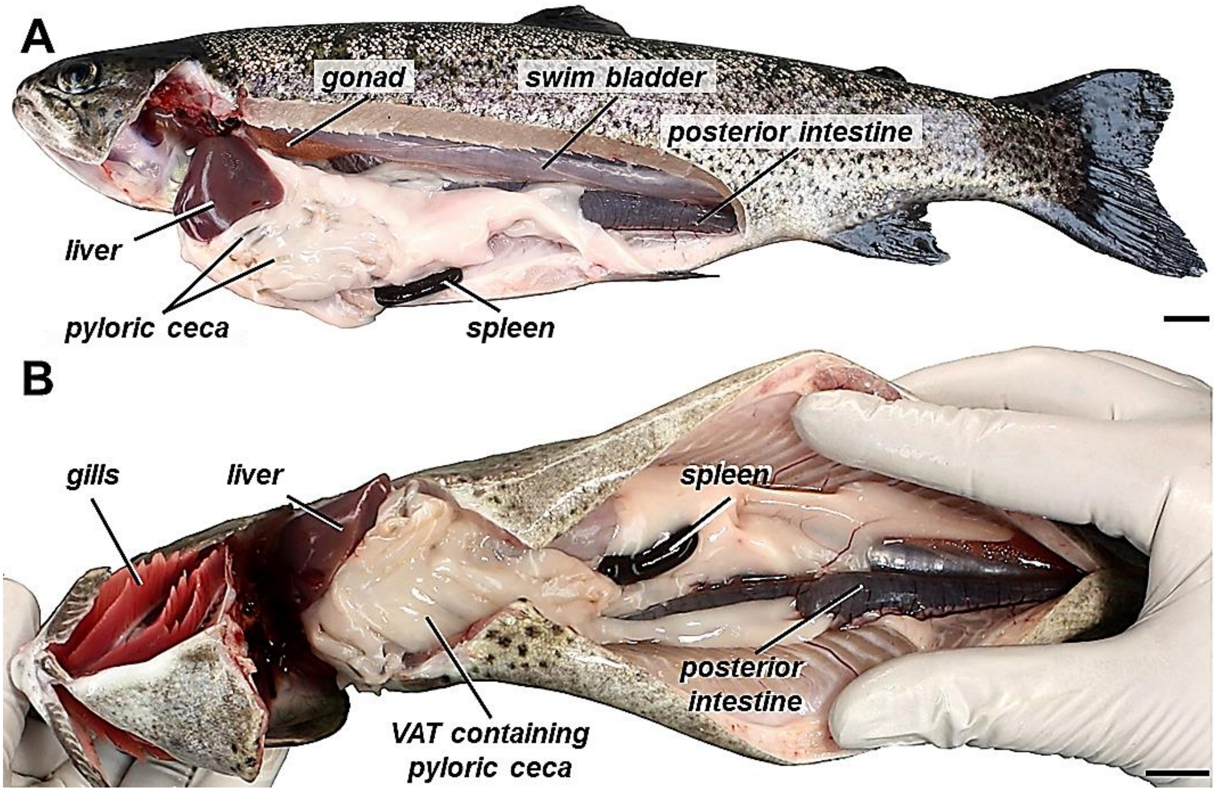

Figure 32. Photographic illustration of the viscera *in situ*. A. Lateral aspect of the viscera of a rainbow trout after removal of the lateral body wall and excision of the gills. **B.** Ventral aspect of the viscera of a rainbow trout after opening of the abdominal cavity by a transverse section in the ventral midline. The spleen is attached to and partly embedded in the visceral adipose tissue (**VAT**). Bars = 1 cm.

At necropsy, the spleen is removed from the abdominal cavity together with the gastrointestinal tract (GIT) and the visceral adipose tissue (VAT) after excision of the gills and the liver. The abdominal cavity is opened by transverse section in the ventral midline, the liver is dissected and the GIT is excised by severing the cranial esophagus and cutting out the vent (urogenital papilla). The connection between the visceral aspect of the spleen and the visceral adipose tissue is separated. After sampling for molecular and histopathological analyses (**Figure 33B**) and transfer of the histopathology sample to neutrally buffered 4% formaldehyde solution, the remaining caudal pole of the spleen is also preserved (*i.e.,* immediately transferred to an adequate fixative), to ensure that sample material is available for new/expanded scientific issues arising from the analyses.

General examination parameters

The dissected spleen is briefly dabbed dry with a laboratory paper towel and weighed to the nearest mg. The spleen is macroscopically examined for pathological alterations, corresponding findings are (photo-) documented. If present, samples of altered tissue locations are taken for subsequent histopathological, molecular or microbiological analysis, as appropriate.

Sampling scheme for routine analyses of the rainbow trout spleen

1. Sample for molecular analyses of the spleen

Location:	Sampling location is indicated in **Figure 33B**.
Number of samples:	One.
Sample size:	Entire cranial portion of the spleen from the cranial margin to the location of the widest organ extension (*i.e.,* the sample location for histopathological analyses).
Remarks:	A homogenous sample is cut from the spleen.
Processing:	The sample is frozen (liquid nitrogen) and stored at -20°C or -80°C (short-term storage) depending on the intended analysis (chemical or molecular analysis). For prolonged storage of the tissue sample for subsequent RNA- and protein analyses, storage at -150°C is recommended.
Downstream analyses:	DNA-, RNA-, protein-, and other OMICS profiling- or targeted analyses requiring cryo-conserved specimen as well as chemical (analytical) analyses.

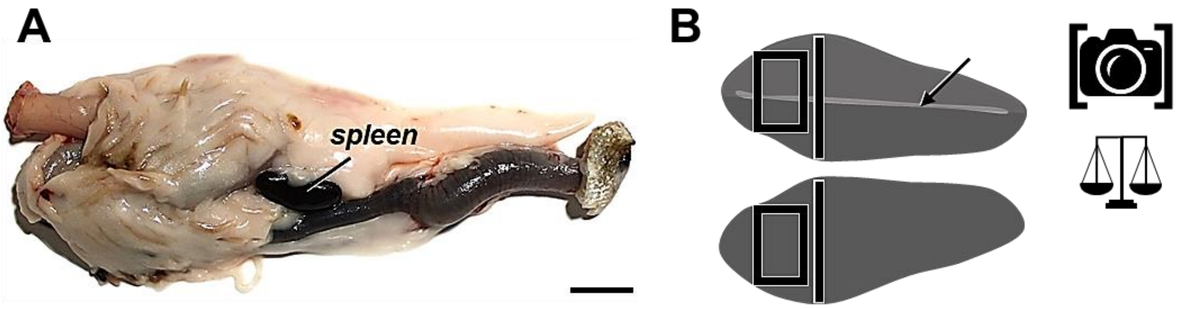

Figure 33. Sampling for histopathological examination and molecular analyses of the spleen. A. Excised gastrointestinal tract and spleen attached to the visceral adipose tissue. The peritoneal attachment at the visceral aspect of the spleen (arrow in **B**) is dissected using scissors. Bar = 1 cm. **B.** Schematic illustration of the excised spleen with indicated sampling locations for routine histopathological and molecular analyses (upper image: visceral aspect; lower image: parietal aspect). The sampling location of the specimen for molecular analysis (black rectangles), as well as sampling location and orientation of the specimen for histopathological examination (black lines) are indicated. Lines in the upper and lower image indicate the identical sampling locations and section plane orientations for routine histopathological and molecular analyses. Both samples contain the spleen capsule.

2. Sample for histopathological examination of the spleen

Location & orientation of sections:	Sampling location and orientation is indicated in **Figure 33B**. A transverse splenic tissue section is cut from the spleen at the location of the widest organ extension.
Number of samples:	One.
Section plane size:	The sample size depends on organ size/diameter, thickness of the tissue sample is ~0.2 cm.
Fixation & embedding:	FF-PE.

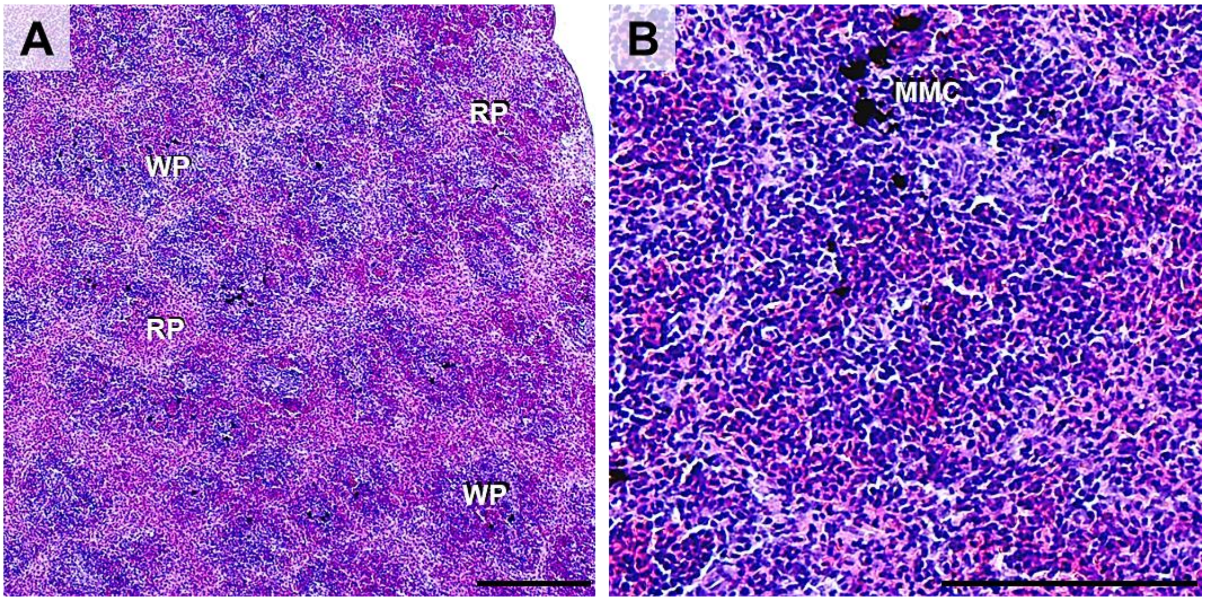

Figure 34. Histology of the rainbow trout spleen. A. Transverse section of rainbow trout spleen parenchyma, enclosed by a thin splenic capsule (a part of the splenic capsule is apparent in the upper right corner). The parenchyma is composed of the red (**RP**) and white (**WP**) pulp. **B.** Transversely sectioned spleen parenchyma. A feature of the rainbow trout spleen is the presence of melanomacrophage centers (**MMC**), composed of pigment-laden macrophages which are involved in the spleen`s immunological function. FF-PE. HE. Bars = 100 µm.

Time requirements

2-3 minutes are to be scheduled for dissection of the GIT and the attached spleen, macroscopic examination of the spleen, sampling and further processing of the histological and molecular samples. This estimate does not include the time needed for killing the fish, dissection of gills/liver, prearrangement of sampling instruments and materials, or the further processing of fixed/frozen specimens.

A comparison of the proposed sampling scheme for routine analyses with previously published studies

Previously published ecotoxicological studies examining rainbow trout spleen either sample the whole organ [96-98] or examine spleen subsamples without distinct information regarding sample number and size [99, 100].

The sampling location for histopathological analyses of the rainbow trout spleen is chosen in accordance with the study by Schwindt et al. [101], where a tissue sample from the middle of the spleen is generated for histological examination. Due to the homogenous composition of the splenic parenchyma, the number and sizes of tissue samples as well as the sampling locations proposed in the present guidelines are regarded to provide sufficiently representative tissue specimens for the examination of most of the qualitative splenic tissue alterations and molecular analyses of the rainbow trout spleen.

2.6 Reproductive system

2.6.1 Testes

Relevant anatomical features/preparation

The paired testes of the rainbow trout present as flattened structures, bilaterally and ventrally located to the swim bladder and attached to the dorsal peritoneum and the serosa of the swim bladder by the mesorchium. They can extend over the entire length of the peritoneal cavity (**Figure 35**) [1, 2]. According to Wünnemann et al. [102], testes weighing <500 mg and presenting as small, pale pink strands are considered prepubertal. Testes weighing >500 mg with a whitish appearance and an enlargement of the anterior testicular part are considered pubertal (**Figure 36**). In sexual mature male trout the testes present as large organs and macroscopic sex determination can be made (*inter alia* based on the size/weight and white color of the testes due to spermatogenesis), the sex of premature rainbow trout cannot be identified macroscopically but can *e.g.,* be determined via measurement of the plasma vitellogenin concentration or via molecular analyses [2, 102, 103]. Size and shape of the trout testes depend on the spermatogenetic stage, season, and age; note that there are differences in the maturity age of rainbow trout individuals [103-105]. Trout`s sperm duct is evident at the posterior-dorsal aspect of the testes and does end at the urogenital papilla. In spawning season, milt is released through pores located at the urogenital papilla next to the urinary- and the anal pore. In contrast to other vertebrates, the sperm-conducting structures occur independently of the ureteral structures [2, 42, 103]. From the testicular capsule, septa of connective tissue extend into the parenchyma and divide it into tubules (**Figure 38**) [42, 103]. Histologically, the tubules are composed of a germinal epithelium, where the spermatogenesis occurs in so-called sperm-cysts (*i.e.,* groups of synchronously maturing germ cells, surrounded by Sertoli cells which build the blood-testis barrier), from which the spermatozoa are released in the tubular lumen, which is connected to the efferent (sperm) duct system [2, 4, 42, 103, 105]. Next to the germinal compartment, the testes are composed of an interstitial compartment between the lobules, comprising Leydig cells and connective tissue with *e.g.,* fibroblasts, blood vessels and nerve fibers [4, 42, 105].

At necropsy, the testes are removed after excision of the gills, the liver and the gastrointestinal tract. Prepubertal testes are removed together with the swim bladder. Pubertal testes are dissected by carefully severing the craniodorsal attachment of testes and swim bladder *in situ* using scissors, care is taken to not perforate the swim bladder. After macroscopic examination and sampling of fresh (unfixed) tissue specimen for molecular analyses, the testes are immersion fixed in an adequate fixative (*e.g.,* Davidson´s fixative for ~12 hours with subsequent transfer to neutrally buffered 4% formaldehyde solution). After sampling for histopathological analyses, the remaining tissue is preserved to ensure that sufficient sample material is available for new/expanded scientific issues arising from the analyses.

General examination parameters

After excision and separation from the swim bladder, both testes are briefly dabbed dry and the weight is recorded to the nearest mg. By means of the gonadosomatic index (GSI) (*i.e.,* the percentage of the body weight that is related to the weight of the testes) the reproductive status may be monitored [2]. The testes are macroscopically examined for pathological alterations and corresponding findings are (photo-) documented. Samples of altered tissue locations are generated for histopathological, microbiological or molecular analyses, as appropriate.

2.6.1 Testes

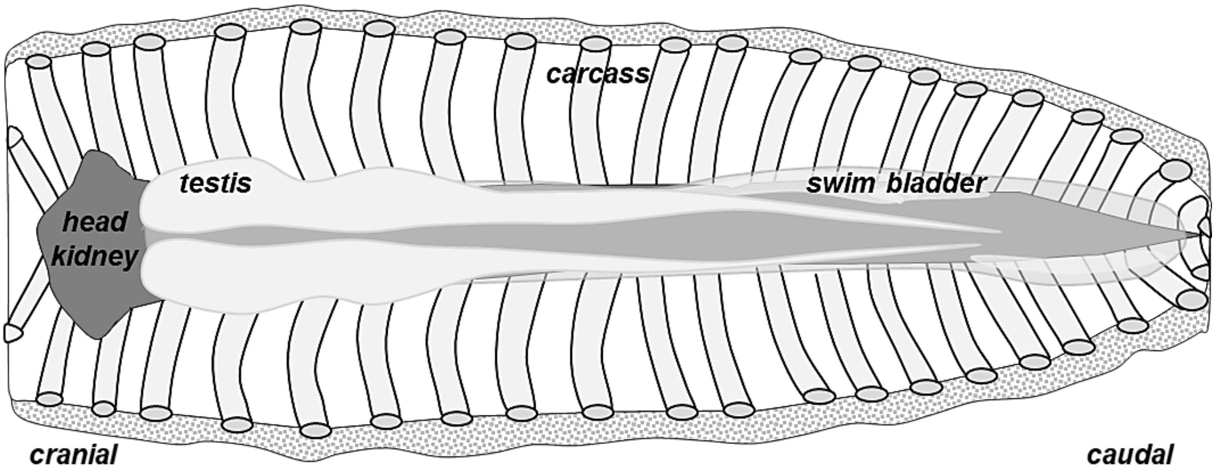

Figure 35. Schematic illustration of the *in situ* location of the testes. Ventral aspect of the abdominal cavity after excision of gills, liver, gastrointestinal tract and spleen.

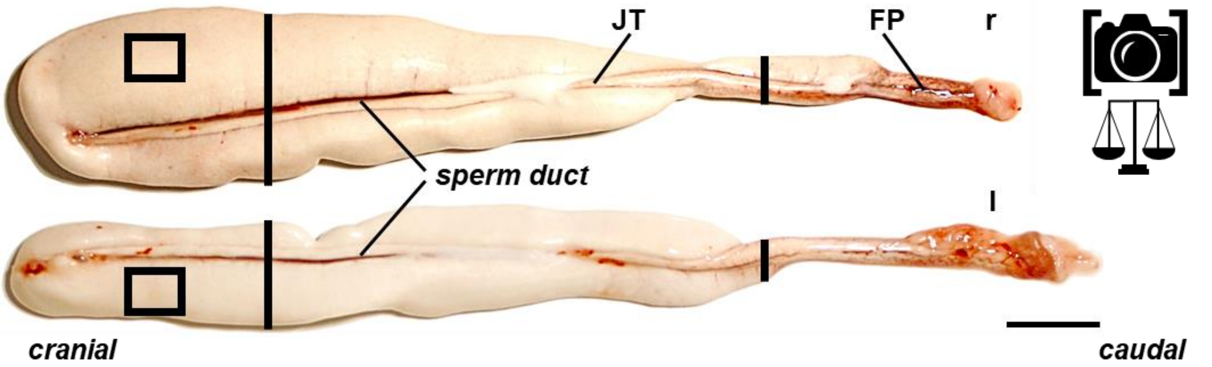

Figure 36. Photographic illustration of the rainbow trout testes and sampling for histopathological and molecular analyses. Dorsal aspect of the freshly excised right (**r**) and left (**l**) testis of an adult male rainbow trout. The sperm duct is subdivided in two parts, the juxta-testicular part (**JT**) adjacent to the testes and the “free” part (**FP**) connecting the posterior testis with the urogenital papilla [42, 103]. Locations and orientations of histopathological (black lines) and molecular samples (black rectangles) are indicated. Bar = 1 cm.

Sampling scheme for routine analyses of the rainbow trout testes

1. Samples for molecular analyses of the testes

Location:	Sampling locations are indicated in **Figures 36** and **37**.
Number of samples:	Two.
Sample size:	Two specimens of ~0.5 cm x 0.5 cm (length x width) are generated, including the dorsal and ventral testicular capsule and the testicular parenchyma; therefore, the sample height depends on testicular size.
Remarks:	Homogenous samples of the testicular parenchyma are cut from the anterior part of the left and the right testis, approximately 1 cm apart from the cranial testicular end.
Processing:	Samples are frozen (liquid nitrogen) and stored at -20°C or -80°C (short-term storage) depending on the intended analysis (chemical or molecular analysis). For prolonged storage of tissue samples for subsequent RNA- and protein analyses, storage at -150°C is recommended.
Downstream analyses:	DNA-, RNA-, protein-, and other OMICS profiling- or targeted analyses requiring cryo-conserved specimen as well as chemical (analytical) analyses.

2. Samples for histopathological examination of the testes

Location & orientation of sections:	Sampling locations and orientations are indicated in **Figures 36** and **37**. After immersion fixation, two transverse section per testis are prepared, from both the anterior and posterior testicular part (approximately 2 cm apart from the cranial and caudal testicular end). The sections contain testicular parenchyma, the sperm duct and the entire parenchyma-surrounding testicular capsule.
Number of samples:	Four.
Section plane size:	All histopathological specimens are transverse sections of the entire testicular cross diameter, with a thickness of approximately 0.3 cm.
Fixation & embedding:	Davidson`s-fixed and paraffin-embedded (DF-PE)*.

**The OECD Guidance Document for the Diagnosis of Endocrine-Related Histopathology of Fish Gonads [106], dedicated to the performance of ecotoxicological studies using Fathead minnow (Pimephales promelas), Japanese medaka (Oryzias latipes) and zebrafish (Danio rerio), recommends overnight immersion fixation with Davidson´s fixative and subsequent transfer to neutrally buffered 4% formaldehyde solution for gonadal histopathology.*

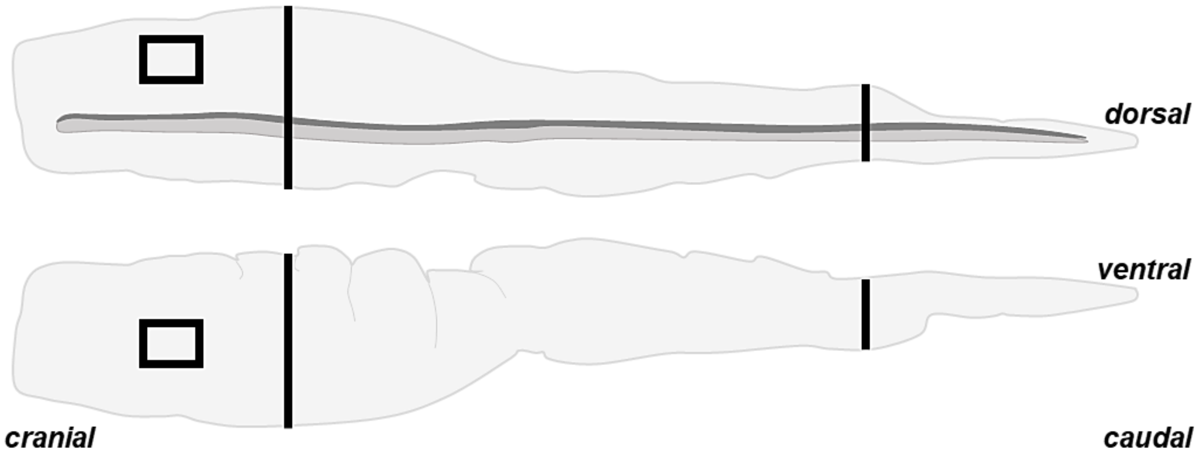

Figure 37. Schematic illustration of the ventral and dorsal aspect of the excised left testis of a male rainbow trout. Both testes are sampled, here the sampling locations for routine analyses are exemplarily shown for the left testis (upper image: dorsal aspect, lower image: ventral aspect). Sample location for molecular analyses (black rectangles) and sample location and orientation for histopathological analyses (black lines) are indicated. The black lines and rectangles indicate the identical sampling locations. All samples contain both the ventral and dorsal testicular capsule (*i.e.*, the samples extend over the entire testicular parenchyma thickness).

Time requirements

Approximately 5 minutes are to be scheduled for dissection of the testes, macroscopic examination, sampling and further processing of testicular tissue samples. This estimate does not include the time needed for killing the fish, dissection of gills and previously excised viscera, prearrangement of sampling instruments and materials, or the further processing of fixed/frozen specimens.

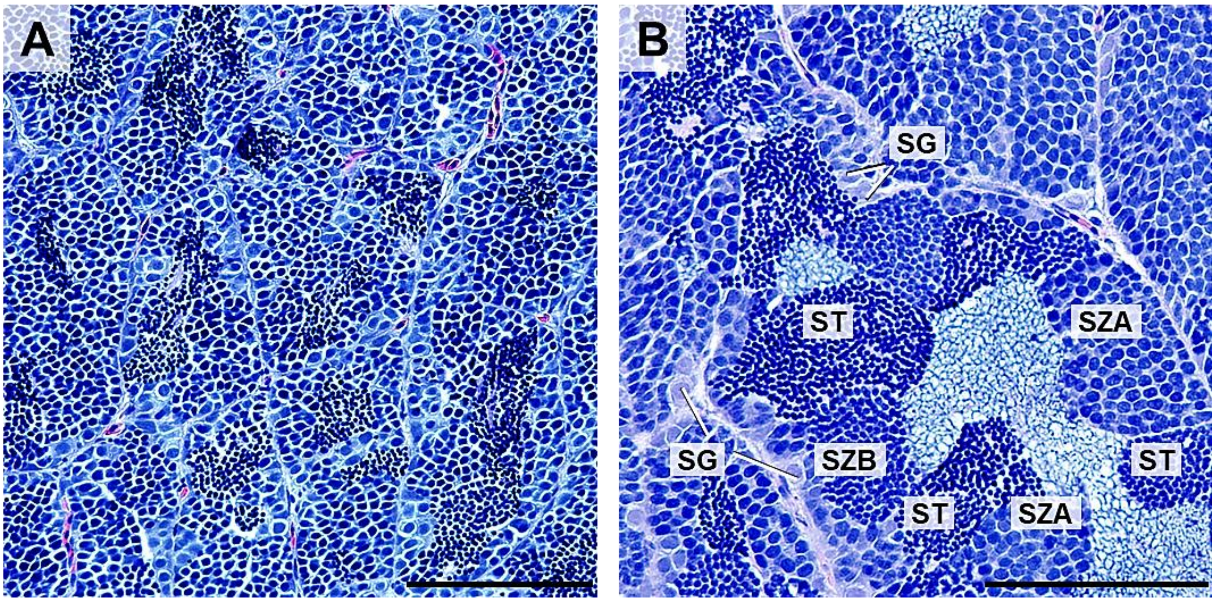

Figure 38. Testicular histology. A. Transverse section of the testicular parenchyma. Note the connective tissue septa, subdividing the parenchyma in distinct tubules. Within the germinal department, different germ cell stages are present. **B.** Transverse section of the testicular parenchyma of a male trout in a progressed stadium of the spermatogenesis cycle. Present germ cell stages are indicated: **SG:** Spermatogonia; **SZA:** Primary spermatocytes; **SZB:** Secondary spermatocytes; **ST:** Spermatids. Cysts of spermatids rupture at the end of spermatogenesis and spermatozoon enter the tubule lumen. FF-PE. HE. Bars = 100 µm.

A comparison of the proposed sampling scheme for routine analyses with previously published studies

In previously performed ecotoxicological studies on trout testes, only few studies provide a specific description of the sampling locations, such as midportions of the testes [107], the anterior, middle and posterior part of the testes [108] or the testes *in toto* [109]. In previously performed ecotoxicological studies using trout as experimental animals, the number of analyzed testicular tissue samples per fish, if indicated, ranges from one [110] to three samples [108]. For the performance of unbiased quantitative morphological analyses of testicular tissue in ecotoxicological studies using rainbow trout, a comprehensive systematic uniform random sampling (SURS) protocol is published by Wünnemann et al. [102].

Related to the homogenous composition of the testicular tissue, the number and sizes of samples as well as the sampling locations proposed in the present guidelines for routine analyses of the testes are regarded to provide sufficiently representative tissue specimens for histopathological and molecular examination of rainbow trout´s testes in ecotoxicological studies.

2.6.2 Ovaries

Relevant anatomical features/preparation

The paired ovaries of the rainbow trout present as elongated structures, bilaterally and ventrally located to the swim bladder and attached to the dorsal peritoneal wall and swim bladder by the mesovarium. They can extend over the entire length of the dorsal peritoneal cavity [2, 4]. In subadult trout, sex determination depends on *e.g.,* histological differentiation, determination of the plasma vitellogenin concentration or molecular analyses; a macroscopic sex determination in female trout is feasible based on the developing ovarian lamellae during maturation (which is often the first indication of sex differentiation) [2, 102]. In maturing trout, the thin ovaries present as organs composed of small orange-white spheres. The ovaries and ova grow in size towards the spawning season and the transversal lamellar folds of the ovaries become more prominent. Mature ovaries with macroscopically visible eggs may account for up to 50-70% of the body weight, while in non-spawning season the weight of the ovaries accounts for only a small proportion of the body weight. Note that there are differences in the maturity age of rainbow trout individuals [2, 3, 104, 111]. An ovocoel and an ovarian duct are missing in salmonids, the eggs are released in the peritoneal cavity while ovulating. During spawning season the eggs are released through pores located at the urogenital papilla next to the urinary and the anal pore [2, 42, 111]. Histologically, the ovarian parenchyma is organized in lamellae which are supported by connective tissue septa and are covered by germinal epithelium, comprising germ cells and somatic cells. The latter are epithelial cells, which initiate the folliculogenesis and become follicular granulosa cells. The ovarian follicle is the main ovarian feature, containing the oocyte and follicle cells. The rainbow trout follicles are differentiated in previtellogenic and vitellogenic follicles, and histologically divided into different types depending on the developmental stage. A permanent pool of oogonia for population renewal is present in contrast to mammalian ovaries. A basement membrane separates the germinal epithelium from the mesenchymal stroma [2, 36, 103, 111, 112].

At necropsy, the ovaries are removed after excision of the gills, liver, spleen and gastrointestinal tract. Depending on the size of the ovaries, the ovaries are removed together with the swim bladder (**Figure 40B**) or the craniodorsal mesovarian attachment of the ovaries is dissected *in situ* using scissors. Care is taken to not perforate the swim bladder. After macroscopic examination and sampling of fresh (unfixed) tissue specimen for molecular analyses, the ovaries are immersion fixed in neutrally buffered 4% formaldehyde solution. After sampling for histopathological analyses, the remaining tissue is preserved to ensure that sufficient sample material is available for new/expanded scientific issues arising from the analyses.

General examination parameters

After separation from the swim bladder, both ovaries are briefly dabbed dry and the weight is recorded to the nearest mg. The reproductive status can be monitored by means of the gonadosomatic index (GSI) (*i.e.,* the percentage of the body weight that is related to the weight of the gonads). Both ovaries are macroscopically examined for pathological alterations, corresponding findings are (photo-) documented. If required, samples of altered tissue locations are generated for histopathological, molecular or microbiological analyses.

2.6.2 Ovaries

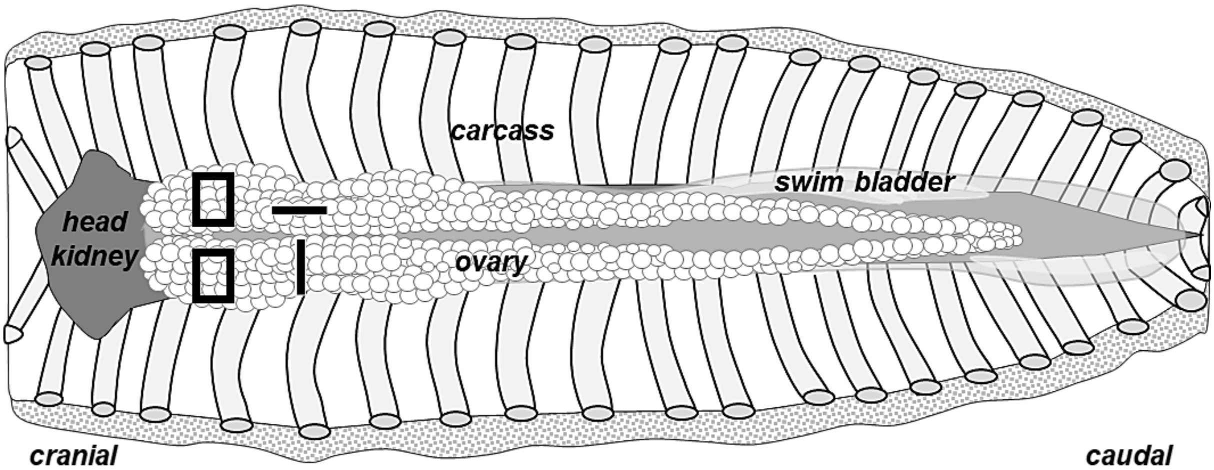

Figure 39. Schematic illustration of the ovaries (as *in situ*). Ventral aspect of the ovaries of the rainbow trout, bilaterally and ventrally located to the swim bladder. Ovaries are removed after excision of gills, liver, gastrointestinal tract and spleen. Both ovaries are sampled, location of molecular samples (black rectangles) as well as location and sample orientation of histopathological analyses specimens (black lines) are indicated.

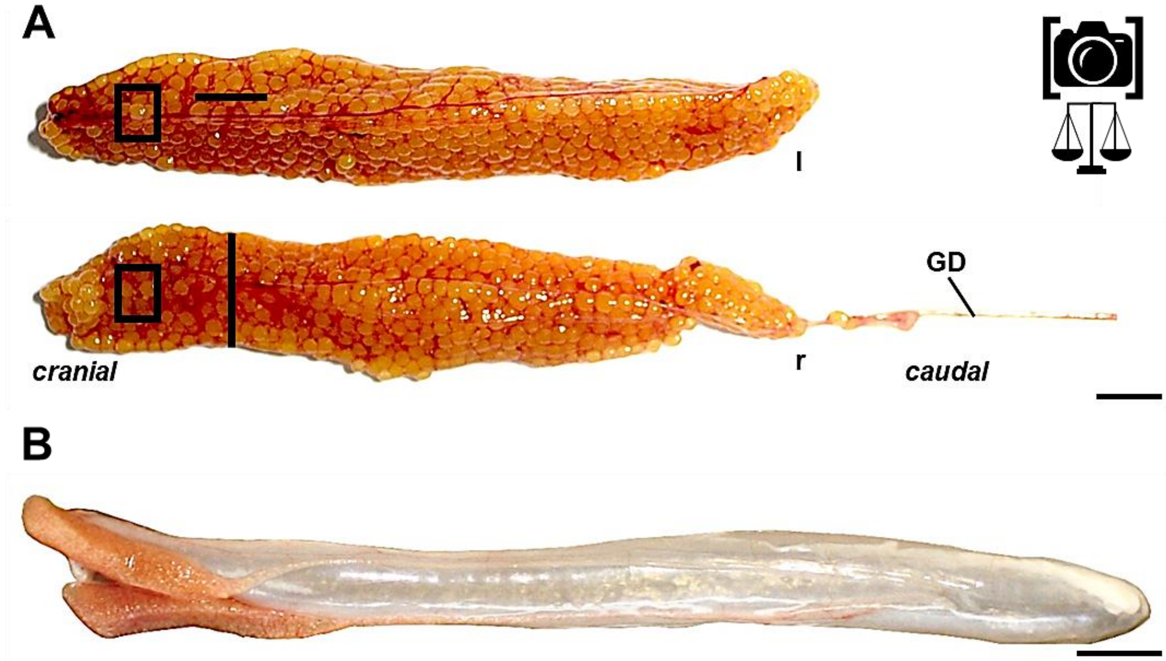

Figure 40. Photographic illustration of the rainbow trout ovaries. A. Ventral aspect of the freshly excised left (**l**) and right (**r**) mature ovary of a female rainbow trout. Note the orange color and the granular appearance due to enclosed, pigmented eggs. Locations of the molecular specimens (black rectangles) as well as sample locations and sample orientations of histopathological specimens (black lines) are indicated. **GD:** Gonaduct, build by the peritoneum, ending at the urogenital sinus and opened to the abdominal cavity [112]. **B.** Freshly excised swim bladder with ventro-laterally attached ovaries. Bars = 1 cm.

Sampling scheme for routine analyses of the rainbow trout ovaries

1. Samples for molecular analyses of the ovaries

Location:	Sampling locations are indicated in **Figures 39** and **40A**.
Number of samples:	Two. Both ovaries are sampled approximately 1 cm apart from the cranial end of the ovaries, one sample per side.
Sample size:	Approximately 0.4 cm x 0.4 cm (length x width). Samples are taken over the entire ovary height.
Remarks:	Homogenous samples of the ovary parenchyma are cut from each ovary.
Processing:	Samples are frozen (liquid nitrogen) and stored at -20°C or -80°C (short-term storage) depending on the intended analysis (chemical or molecular analysis). For prolonged storage of tissue samples for subsequent RNA- and protein analyses, storage at -150°C is recommended.
Downstream analyses:	DNA-, RNA-, protein-, and other OMICS profiling- or targeted analyses requiring cryo-conserved specimen as well as chemical (analytical) analyses.

2. Samples for histopathological examination of the ovaries

Location & orientation of sections:	After immersion fixation, one specimen per ovary is sampled approximately 2 cm apart from the cranial end of the ovary. One transverse section and one sagittal section is prepared as indicated in **Figures 39** and **40A**. Both sections contain ovarian parenchyma and the dorsal and ventral mesovarium.
Number of samples:	Two.
Section plane size:	Both histopathological sections (transverse and sagittal) are taken over the entire ovary height, with a thickness of approximately 0.3 cm. Width of the sagittal section: ~1 cm.
Fixation & embedding:	FF-PE*.

**The OECD Guidance Document for the Diagnosis of Endocrine-Related Histopathology of Fish Gonads [106] recommends overnight immersion fixation with Davidson´s fixative and subsequent transfer to neutrally buffered 4% formaldehyde solution for gonadal histopathology of fathead minnow, Japanese medaka and zebrafish. In the authors experience, neutrally buffered 4% formaldehyde solution is also suitable and recommended for the preparation of histological sections, as Davidson's fixative has the potential to harden the ovarian tissue (and particularly mature eggs) and therefore preparation of histological sections may be challenging.*

2.6.2 Ovaries

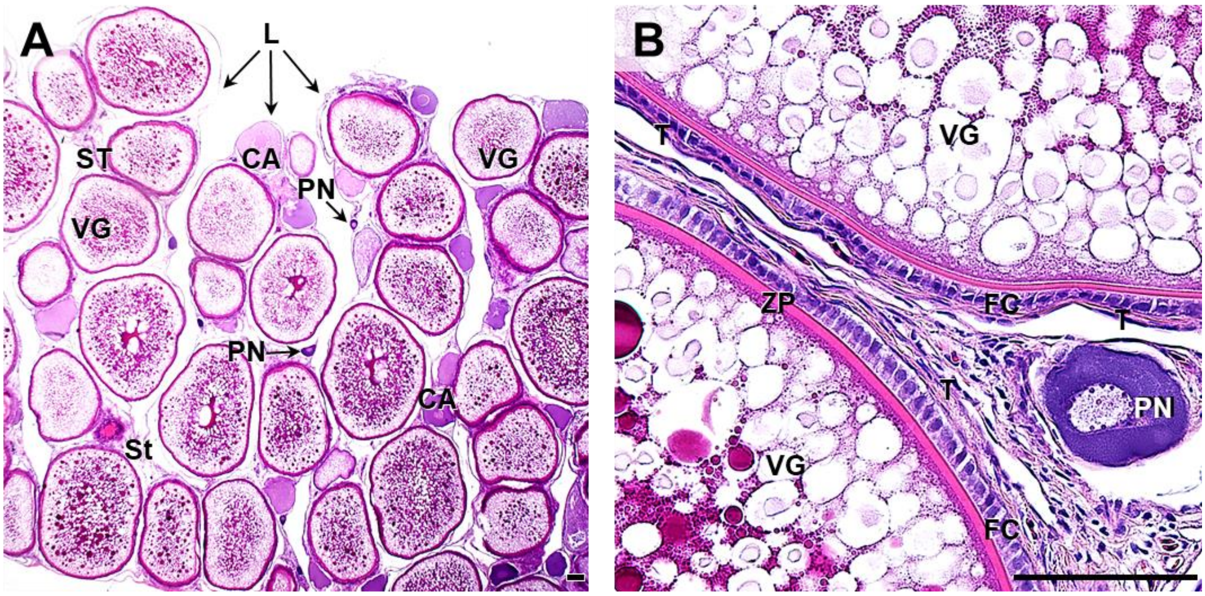

Figure 41. Ovary histology. A. Sagittal section of the ovary parenchyma. Note the ovarian organization in transversal lamellae. Different stages of oocyte development are present. Structures of interest are indicated: **L:** Lamellae; **St:** Stroma; **VG:** Vitellogenic oocyte; **CA:** Cortical alveolar oocyte; **PN:** Previtellogenic (perinucleolar) oocyte. **B.** Section of two vitellogenic oocytes (**VG**) with numerous cortical alveoli, and one previtellogenic (perinucleolar) oocyte (**PN**). Structures of oocyte envelope are indicated: **ZP:** Zona pellucida; **FC:** Follicle cells (granulosa); **T:** Theca. FF-PE. HE. Bars = 100 µm.

Time requirements

Approximately 5 minutes are to be scheduled for dissection of the ovaries, macroscopic examination, sampling and further processing of ovarian tissue samples. This estimate does not include the time needed for killing the fish, dissection of gills and previously excised viscera, prearrangement of sampling instruments and materials, or the further processing of fixed/frozen specimens.

A comparison of the proposed sampling scheme for routine analyses with previously published studies

In previously performed ecotoxicological studies on trout, only few studies provide a specific description of the ovarian sampling locations, such as a sample the middle portion of the ovary [9] or dividing the ovaries in two parts [108]. In previously performed ecotoxicological studies using rainbow trout as experimental animals, the number of analyzed ovarian tissue samples per fish, if indicated, ranges from one [9, 113] to two samples (either subsamples [114] or both ovaries *in toto* with generation of three to six sections [115]).

Related to the homogenous composition of the ovarian tissue, the number and sizes of samples, as well as the sampling locations proposed in the present guidelines for routine analyses of the ovaries are regarded to provide sufficiently representative tissue specimens for histopathological and molecular examination of rainbow trout´s ovaries in ecotoxicological studies.

2.7 Kidneys

Relevant anatomical features/preparation

The rainbow trout kidneys are located retroperitoneally, directly ventral to the spine. They consist of two histologically and functionally different parts, the posterior kidney (*i.e.,* the trunk kidney (TK)) and the anterior kidney (*i.e.,* the head kidney (HK)). Main function of the posterior (excretory) kidney is the diuresis (*i.e.,* excretion and osmoregulation), whereas the anterior kidney is of hematopoietic and immunological function [2, 4, 36]. Macroscopically, the border between the anterior and the posterior kidney, as well as the boundary between the bilateral kidneys cannot directly be identified [2-4, 116]. For practical purposes, the border between the broad anterior and the narrow posterior part of the kidney can be defined as an imaginary transversal line at the level of the craniodorsal gonadal attachment (**Figures 42**-**44**). Histologically, a clear organization in cortex and medulla (as in mammals) is missing. The head kidney comprises hematopoietic, lymphoid (**Figure 45A**) and endocrine tissue (*i.e.,* corticosteroid-producing interrenal tissue (**Figure 45B**) and suprarenal tissue with catecholamine-producing chromaffin cells) [2, 4, 42]. The trunk kidney is composed of glomerular nephrons, embedded in loose interstitial tissue comprised of hematopoietic cells, melanomacrophage centers and fibroblasts (**Figure 46A**), endocrine corpuscles of Stannius are embedded in the parenchyma of the trunk kidney (**Figure 46C**). The rainbow trout nephron is of the freshwater type, composed of renal corpuscles, a renal tubules system and a collecting tubule-collecting duct system connecting the nephrons with the archinephric duct (*i.e.,* the mesonephric duct or ureter) (**Figure 46B**) [2, 4, 36, 42, 116]. The paired archinephric duct fuses to the urinary bladder, which ends in the urogenital papilla [3, 42]. The rainbow trout's kidneys have a renal portal system [2, 42].

If deemed beneficial for the study purpose, vascular perfusion fixation may be performed, the technique of perfusion fixation of rainbow trout tissue is described in **Chapter 2.3.2** and elsewhere [13]. At necropsy, the kidneys are assessed after removal of the other viscera (gills, heart, gastrointestinal tract, liver, spleen, swim bladder and gonads). Due to the delicate, soft consistency of kidney tissue, samples of fresh kidney tissue are only taken for subsequent molecular analyses, whereas the rest of the kidney remains connected to the spine as *in situ* for fixation. To enhance immersion fixation of the renal tissue, the spine is cut cranially and caudally of the kidney and adjacent skeletal muscles are spaciously removed (**Figure 42**) before the tissue is transferred to the fixation solution (neutrally buffered 4% formaldehyde solution).

General examination parameters

After fixation, the kidney is carefully dissected from the adjacent tissue, briefly dabbed dry with laboratory paper towel to remove surplus fixation solution and weighed to the nearest mg. The kidney is macroscopically examined for pathological alterations, corresponding findings are (photo-) documented. If present, samples of altered tissue locations are taken for subsequent histopathological, virological and microbiological analysis, as appropriate.

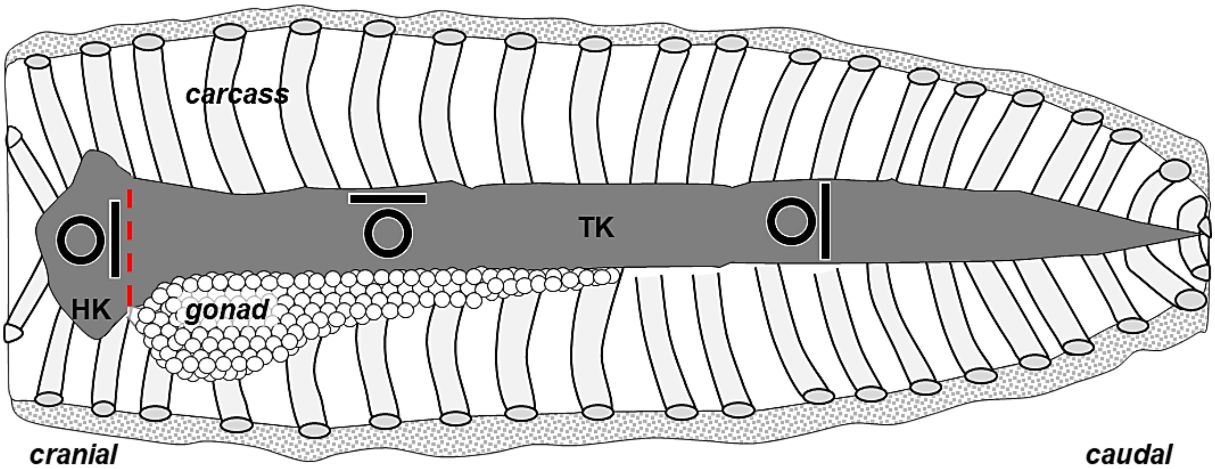

Figure 42. Schematic illustration of the ventral aspect of the head kidney (HK) and trunk kidney (TK). Sample positions of molecular analysis samples (black circles) as well as sample locations and section plane orientations of samples for histopathological analyses (black lines) are indicated. The border between the anterior and the posterior part of the kidney is defined as an imaginary transversal line at the level of the craniodorsal gonadal attachment (red dashed line). The kidney remains connected to the carcass, the spine is cut cranially and caudally of the kidney and adjacent skeletal muscles are spaciously removed for enhanced immersion fixation.

Sampling scheme for routine analyses of the rainbow trout head and trunk kidney

1. Samples for molecular analyses of the head and trunk kidney

Head kidney (HK)

Number of samples:	One.
Location:	The sampling location is indicated in **Figures 42** and **43B**.
Sample size:	A full thickness tissue cylinder with 0.4 cm diameter is excised by biopsy punch.
Remarks:	A homogenous specimen of (fresh, unfixed) HK parenchyma is taken from the ventral aspect of the HK. The weight of the specimen is recorded.

Trunk kidney (TK)

Number of samples:	Two.
Location:	Sampling locations are indicated in **Figures 42** and **43B**.
Sample size:	Full thickness tissue cylinders with 0.4 cm diameter are excised by biopsy punch.
Remarks:	Homogenous specimens of (fresh, unfixed) TK parenchyma are taken from the ventral aspect of the TK. The weight of the specimens is recorded.
Processing (HK & TK):	Freezing (liquid nitrogen) and stored at -20°C or -80°C (short-term storage) depending on the intended analysis (chemical or molecular analysis). For prolonged storage of tissue samples for subsequent RNA- and protein analyses, storage at -150°C is recommended.
Downstream analyses:	DNA-, RNA-, protein-, and other OMICS profiling- or targeted analyses requiring cryo-conserved specimen as well as chemical (analytical) analyses.

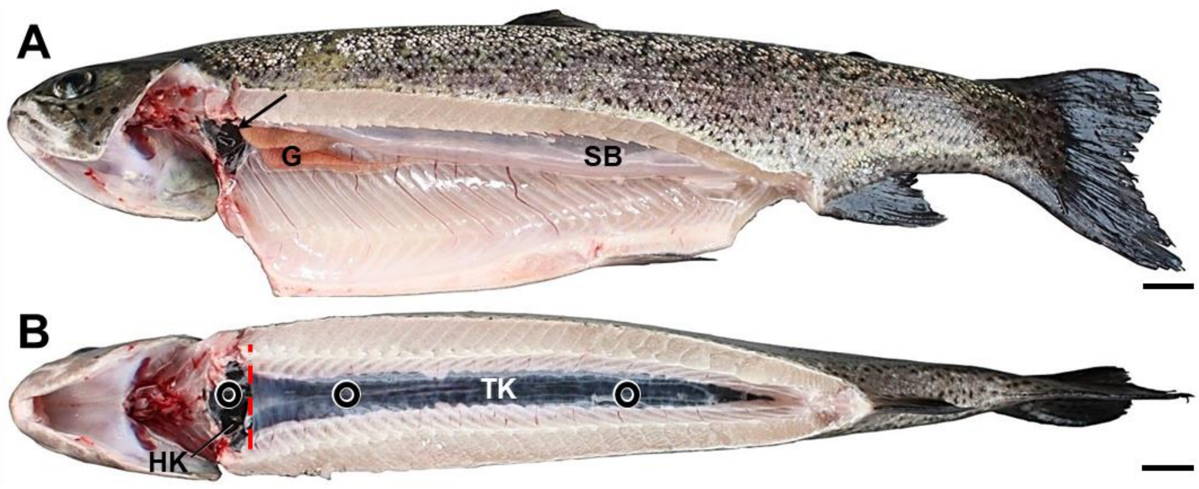

Figure 43. Sampling of head and trunk kidney specimens for molecular analyses in ecotoxicological studies. A. Ventro-lateral aspect of the necropsy situs after removal of the gills, the gastrointestinal tract, liver and spleen. The border between the head kidney and trunk kidney, defined by the cranial attachment of the gonads (**G**) to the kidney, is indicated by an arrow. **SB:** Swim bladder. **B.** Ventral aspect of the *in situ* location of the head- and trunk kidney after removal of the gonads and the swim bladder. The red dashed line indicates the border between the head- and the trunk kidney. Sampling locations for molecular analysis samples are indicated (black circles). **HK:** Head kidney; **TK:** Trunk kidney. Bars = 1 cm.

2. Samples for histopathological examination of the head and trunk kidney

Head kidney (HK)

Number of samples:	One.
Location & orientation of sections:	The sampling location and orientation is indicated in **Figures 42** and **44**. After immersion fixation, the adherent skin and musculature is removed from the kidney and the HK is separated from the TK. One transverse section plane is generated, containing the ventral and the dorsal organ capsule.
Section plane size:	Approximately 0.4 cm x 0.6 cm (length x width).
Fixation & embedding:	FF-PE.

Trunk kidney (TK)

Number of samples:	Two.
Location & orientation of sections:	Sampling locations and orientations are indicated in **Figures 42** and **44**. After immersion fixation, the adherent skin and musculature are removed and the TK is separated from the carcass. The weight of the TK tissue is recorded. One cross and one longitudinal section plane is generated from the TK parenchyma, including both the ventral and dorsal organ capsule.
Section plane size:	Approximately 0.5 cm x 0.6 cm (length x width).
Fixation & embedding:	FF-PE.

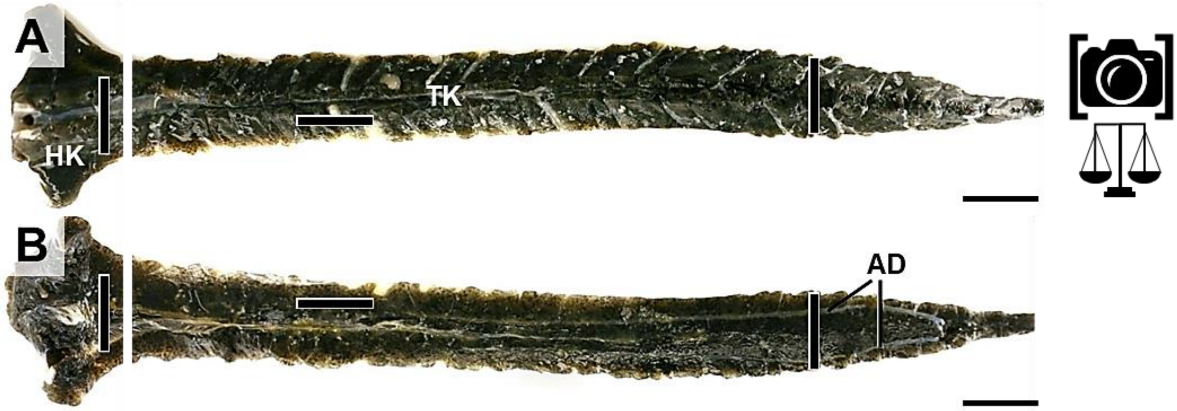

Figure 44. Dorsal (A) and ventral (B) aspect of a formalin-fixed kidneys (after dissection from the adjacent carcass tissue) with indicated sampling locations for histopathological analyses in routine ecotoxicological studies. Lines in **A** and **B** indicate the identical sampling locations and section plane orientations for routine histopathological analyses. All samples contain both the ventral and dorsal kidney capsule. The archinephric ducts (also called mesonephric ducts or ureters) on the ventral aspect of the excretory kidney are indicated (**AD**). **HK:** Head kidney; **TK:** Trunk kidney. Bars = 1 cm.

Time requirement

Approximately 7-10 minutes are to be scheduled for the dissection of the head- and trunk kidney and further processing of kidney tissue samples for molecular and histopathological analyses. This estimate neither includes the time needed for killing the fish, the prearrangement of sampling instruments and materials and the removal of the other viscera, nor the further processing of fixed/frozen specimens. If vascular perfusion fixation is performed, at least 15 minutes should be scheduled additionally.

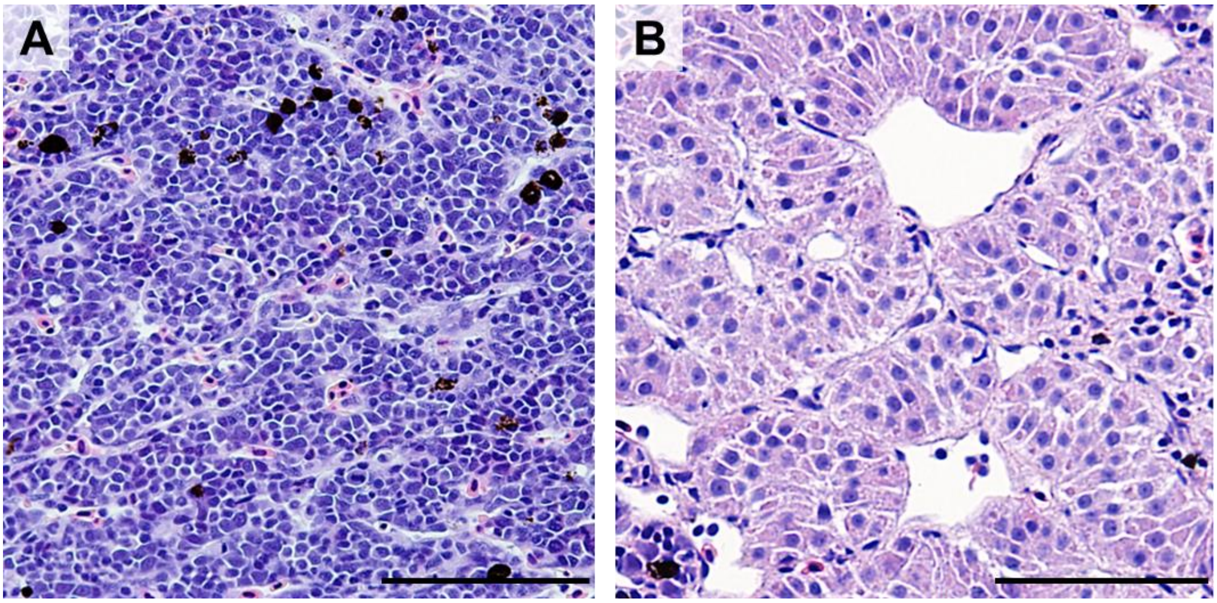

Figure 45. Histology of the head kidney. A. Hematopoietic tissue of the anterior (head) kidney. **B.** Interrenal tissue. Corticosteroid-producing cells, arranged around posterior cardinal veins. The venous vascular space is free of cellular blood compartments due to vascular perfusion fixation, a detailed description of vascular perfusion fixation in rainbow trout is given in **Chapter 2.3.2** or Fiedler et al. [13]. FF-PE. HE. Bars = 100 µm.

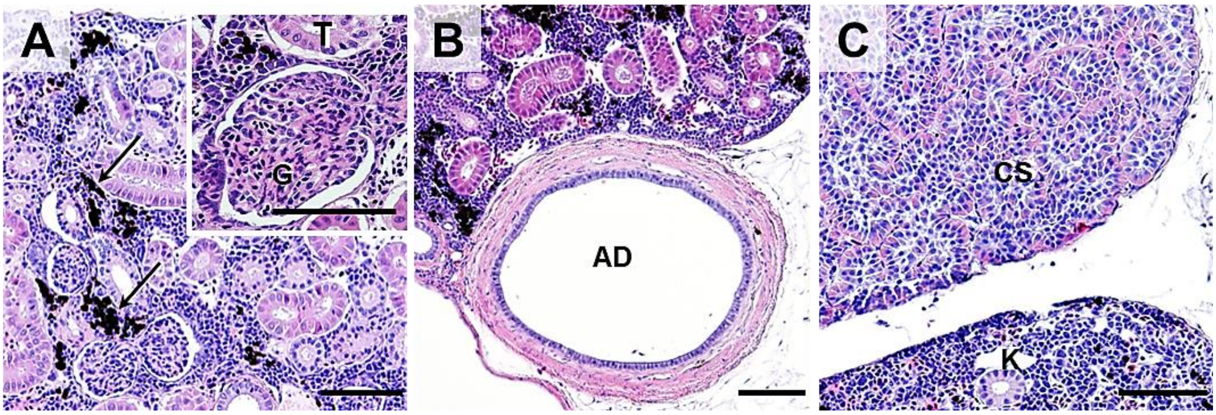

Figure 46. Histology of the trunk kidney. A. Trunk kidney parenchyma. Note that the trout trunk kidney tissue displays no separation in cortex and medulla as *e.g.,* in mammals. Nephrons are embedded in loose interstitial tissue consisting of hematopoietic cells, melanomacrophage centers (indicated by black arrows) and fibroblasts. Inset: Enlargement of a glomerulus (**G:** Glomerulus; **T:** Tubule). **B.** Cross section of the archinephric duct (**AD**). **C**. Corpuscle of Stannius (**CS**) on the dorsal aspect of the excretory kidney (**K**). FF-PE. HE. Bars = 100 µm.

A comparison of the proposed sampling scheme for routine analyses with previously published studies

Only few previously performed ecotoxicological studies examining rainbow trout trunk kidney (TK) samples provide a precise description of the sampling locations. Samples of the TK are *e.g.,* taken in the posterior part [117] or the middle portion of the posterior kidney [55]. In previously performed ecotoxicological studies, the number of analyzed TK samples per fish, if indicated, ranges from one (*i.e.,* the entire organ) [58] to four samples [59]. In previously performed ecotoxicological studies on trout head kidney (HK), the HK is either analyzed *in toto* [56] or no specific sampling locations and numbers are provided for the HK analysis. For the performance of unbiased quantitative morphological analysis of the TK in ecotoxicological studies using rainbow trout, a comprehensive systematic uniform random sampling (SURS) protocol is published by Wünnemann et al. [102].

Considering the functional and histomorphological differences between the hematopoietic and lymphatic HK tissue and the excretory TK, the anterior and posterior kidney are sampled and analyzed separately. The sample number and sizes, as well as the sampling locations submitted in the present guidelines are regarded to provide sufficiently representative specimen for examination of the most qualitative (morphological) and functional HK and TK alterations. The size and localization of the generated samples are selected so that they contain hematopoietic, lymphatic and endocrine tissue components (HK) or excretory renal tissue and sections of the archinephric ducts (TK). The endocrine tissues located in the parenchyma of the head- and trunk kidney (*i.e.,* the inter- and suprarenal tissue and the corpuscles of Stannius) are addressed in detail in **Chapters 2.13.4** and **2.13.5**. Sampling of the urinary bladder or urogenital papilla is not included in the present guidelines, if sampling and analyses of these structures is scheduled, the sampling protocol has to be adapted accordingly.

2.8 Central nervous system: Brain and spinal cord

Relevant anatomical features/preparation

The rainbow trout`s central nervous system (CNS) is composed of the brain and the spinal cord as in other vertebrates [36]. The CNS is surrounded by the primitive meninx, *i.e.,* a single meningeal layer [2, 118]. The brain is subdivided in three major divisions: the forebrain (prosencephalon), the midbrain (mesencephalon) and the hindbrain (rhombencephalon). The forebrain is composed of the paired telencephalon (with the olfactory bulb, the dorsal pallium and the ventral subpallium) and the unpaired diencephalon (with the epithalamus (for sampling of the pineal body refer to **Chapter 2.13.6**), the dorsal and ventral thalamus and the hypothalamus). The pituitary gland is located ventral to the hypothalamus. The midbrain comprises the tectum (with the prominent optic tectum) and the tegmentum. The hindbrain is composed of the rostral metencephalon (with the ventral pons and the dorsal cerebellum, which in salmonids forms an anteriorly directed valvula cerebelli) and the ventro-caudally located medulla oblongata (myelencephalon) [2-4, 42, 118]. The posterior transition of the medulla oblongata in the spinal cord is indistinct, the spinal cord runs the entire length of the vertebral canal and is composed of the central grey and peripheral white matter [3, 118]. The neurons of the teleost CNS resemble those of other mammals in their ultrastructure, with some exceptions such as the giant Mauthner cells (**Figure 50F**) [3, 4, 118].

If deemed beneficial for the study purpose, vascular perfusion fixation may be performed. The technique of perfusion fixation of rainbow trout tissue is described in **Chapter 2.3.2** and elsewhere [13]. At necropsy, the CNS is removed after excision of the gills and the viscera (except the kidneys), the head remains connected to the body so as to avoid damaging the head kidney (refer to **Chapter 2.7**). The cranial vault and supraorbital bones as well as the dorsal aspects of the vertebral column with overlying skin and muscles are carefully removed using scalpel and scissors or Liston forceps (**Figure 47A**). The two optic nerves are either severed (**Figure 49**) or the circumorbital bones are carefully removed and the eyeballs dissected from the orbit, so that eyes and optic nerves are excised together with the CNS (**Figure 47B**&**C**). The spinal cord is severed by a cross section about 1 cm caudal to the hindbrain. After excision, macroscopic examination and (photo-) documentation (if appropriate), the samples for histopathological and molecular analyses of the spinal cord are generated (**Figure 47A**). The freshly excised brain is longitudinally halved in the midline (**Figure 49A**). The right brain half is transferred to neutrally buffered 4% formaldehyde solution for subsequent histopathological analysis, the left (unfixed) brain half is sampled for molecular analyses.

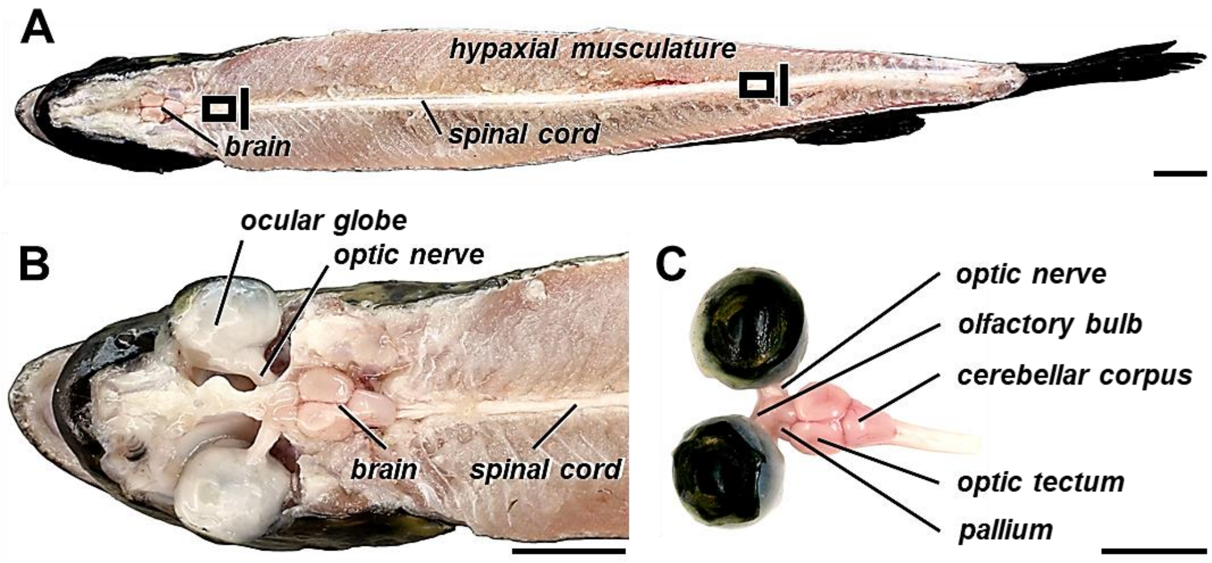

Figure 47. Photographic illustration of the central nervous system. A. Dorsal aspect of the central nervous system (brain and spinal cord) of a rainbow trout after removal of dorsal body musculature, dorsal aspects of the scull and the vertebral neural spines. Sample positions of molecular analysis samples (black rectangles) as well as sample locations and section plane orientations of samples for histopathological analyses (black lines) of the spinal cord are indicated. **B.** Detail enlargement of the exposed spinal cord, brain, optic nerves and ocular globes. **C**. Dorsal aspect of the dissected brain and anterior part of the spinal cord. The ocular globes were carefully removed from the orbit and are still connected to the brain via the optic nerves. Macroscopically visible structures are indicated. Bars = 1 cm.

General examination parameters

The dissected brain and spinal cord (optic nerves are severed and spinal cord is transected about 1 cm caudal to the hindbrain) are briefly dabbed dry with a laboratory paper towel. The brain and spinal cord are macroscopically examined for pathological alterations, corresponding findings are (photo-) documented. The trunk spinal cord is sampled for histopathological and molecular samples directly caudal to the hindbrain, subsequently the sole brain is weighed to the nearest mg. If present, samples of altered tissue locations are taken for subsequent histopathological, molecular or microbiological analysis, as appropriate.

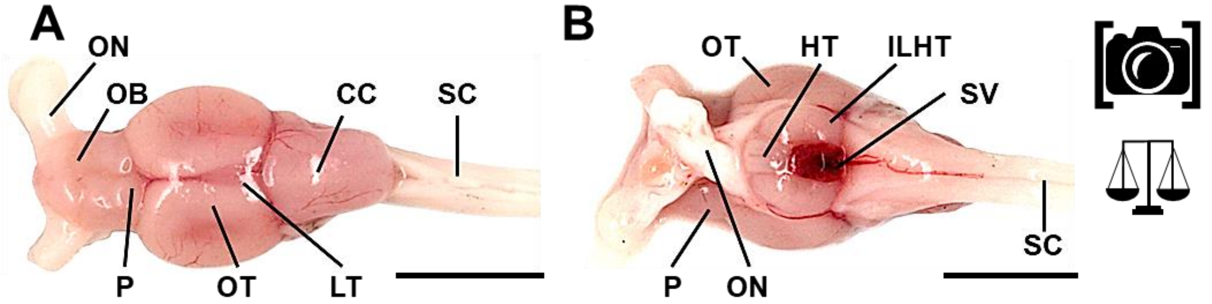

Figure 48. Photographic illustration of the freshly excised brain. A. Dorsal aspect of the excised brain with the attached optic nerves and spinal cord. Important morphological structures are indicated: **ON:** Optic nerve; **OB:** Olfactory bulb; **CC:** Cerebellar corpus; **SC:** Spinal cord; **P:** Pallium; **OT:** Optic tectum; **LT:** Longitudinal torus. **B.** Ventral aspect of the excised brain. Important morphological structures are indicated: **OT:** Optic tectum; **HT:** Hypothalamus; **ILHT:** Inferior lobe of the hypothalamus; **SV:** Saccus vasculosus (choroid plexus which secrets cerebrospinal fluid for the third ventricle); **P:** Pallium; **ON:** Optic nerve; **SC:** Spinal cord. Bars = 0.5 cm.

Sampling scheme for routine analyses of rainbow trout brain and spinal cord

1. Samples for molecular analyses of the brain and spinal cord

Brain

Number of samples:	Three.
Location:	The sampling locations are indicated in **Figure 49B.**
Sample size:	**Sample No. 1:** ~0.3 cm x 0.3 cm x 0.3 cm (length x width x height).<br>**Sample No. 2:** ~0.8 cm x 0.6 cm x 0.6 cm.<br>**Sample No. 3:** ~0.6 cm x 0.5 cm x 0.4 cm.
Remarks:	Specimens of (fresh, unfixed) brain tissue are taken from the left brain half:<br>**Sample No. 1:** Telencephalon.<br>**Sample No. 2:** Diencephalon and mesencephalon. If advantageous for the research issue, the sampling scheme may be adapted by separating the two compartments for independent analyses.<br>**Sample No. 3:** Rhombencephalon (including valvula cerebelli).

Spinal cord

Number of samples:	Two.
Location:	Sampling locations are indicated in **Figure 47A.** Molecular samples are taken directly caudal to the sample sites of the histopathological specimens.
Sample size:	Cylindrical specimens of (fresh, unfixed) spinal cord are generated from the trunk and the caudal region of the spinal cord with a cylinder height of approximately 0.5 cm.
Remarks:	Musculature and dorsal aspects of the neural spines of the vertebrae are carefully removed and homogenous cylindrical specimens of spinal cord are excised from the vertebral column.
Processing (Brain & spinal cord):	Freezing (liquid nitrogen) and stored at -20°C or -80°C (short-term storage) depending on the intended analysis (chemical or molecular analysis). For prolonged storage of tissue samples for subsequent RNA- and protein analyses, storage at -150°C is recommended.
Downstream analyses:	DNA-, RNA-, protein-, and other OMICS profiling- or targeted analyses requiring cryo-conserved specimen as well as chemical (analytical) analyses.

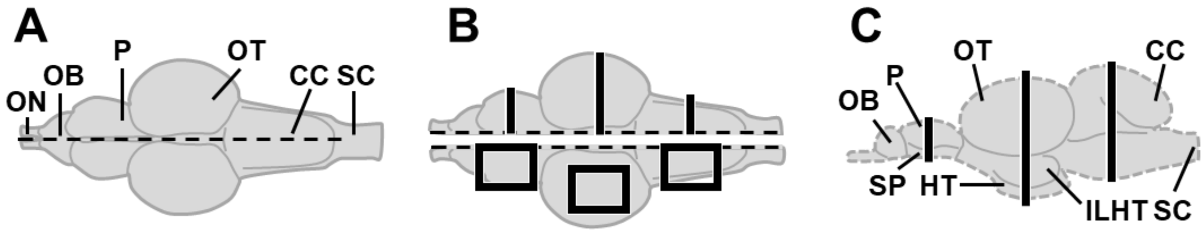

Figure 49. Sampling for histopathological examination and molecular analyses of the brain. A. Dorsal aspect of the excised brain and adherent spinal cord. Important morphological structures are indicated: **ON:** Olfactory nerve; **OB:** Olfactory bulb; **P:** Pallium; **OT:** Optic tectum; **CC:** Cerebellar corpus; **SC:** Spinal cord. The dotted line indicates the separation line of the right and left brain hemisphere. **B.** Illustration of the excised and midsagittally halved brain with indicated sampling locations for routine histopathological and molecular analyses. The sampling location of the specimen for molecular analysis (black rectangles, left brain half), as well as sampling location and orientation of the specimen for histopathological examination (black lines, right brain half) are indicated. Note that samples for molecular analyses are generated from the fresh, unfixed brain tissue, whereas histopathological samples are generated from formalin-fixed brain tissue. **C.** Lateral aspect of the right brain half with indicated sample locations and section plane orientations for histopathological analyses. Important morphological structures are indicated: **OB:** Olfactory bulb; **P:** Pallium; **OT:** Optic tectum; **CC:** Cerebellar corpus; **SP:** Subpallium; **HT:** Hypothalamus; **ILHT:** Inferior lobe of the hypothalamus; **SC:** Spinal cord.

2. Samples for histopathological examination of the brain and spinal cord

Brain

Number of samples:	Three.
Location & orientation of sections:	The sampling locations and orientations are indicated in **Figure 49B**&**C**. After midsagittal division of the brain and immersion fixation of the right brain half, three transverse section planes are generated from three defined levels:<br>**1st level**: Telencephalon (pallium and subpallium).<br>**2nd level**: Mesencephalon (optic tectum, tegmentum) and diencephalon.<br>**3rd level**: Rhombencephalon (cerebellum and medulla oblongata).
Section plane size:	All transversal section planes contain the entire level section profile of the right hemisphere, sample height is approximately 0.3 cm.
Fixation & embedding:	FF-PE.

Spinal cord

Number of samples:	Two.
Location & orientation of sections:	Sampling locations and orientations are indicated in **Figure 47A**. After removal of musculature and dorsal aspects of the vertebral column and subsequent immersion fixation, a transverse (*i.e.,* cross) section plane is generated of each of the two regions of the spinal cord (trunk and caudal region).
Section plane size:	Cylindrical specimens of fixed spinal cord are generated, with a height of ~0.3 cm. The section plane size is approximately 0.15 cm x 0.15 cm.
Fixation & embedding:	FF-PE.

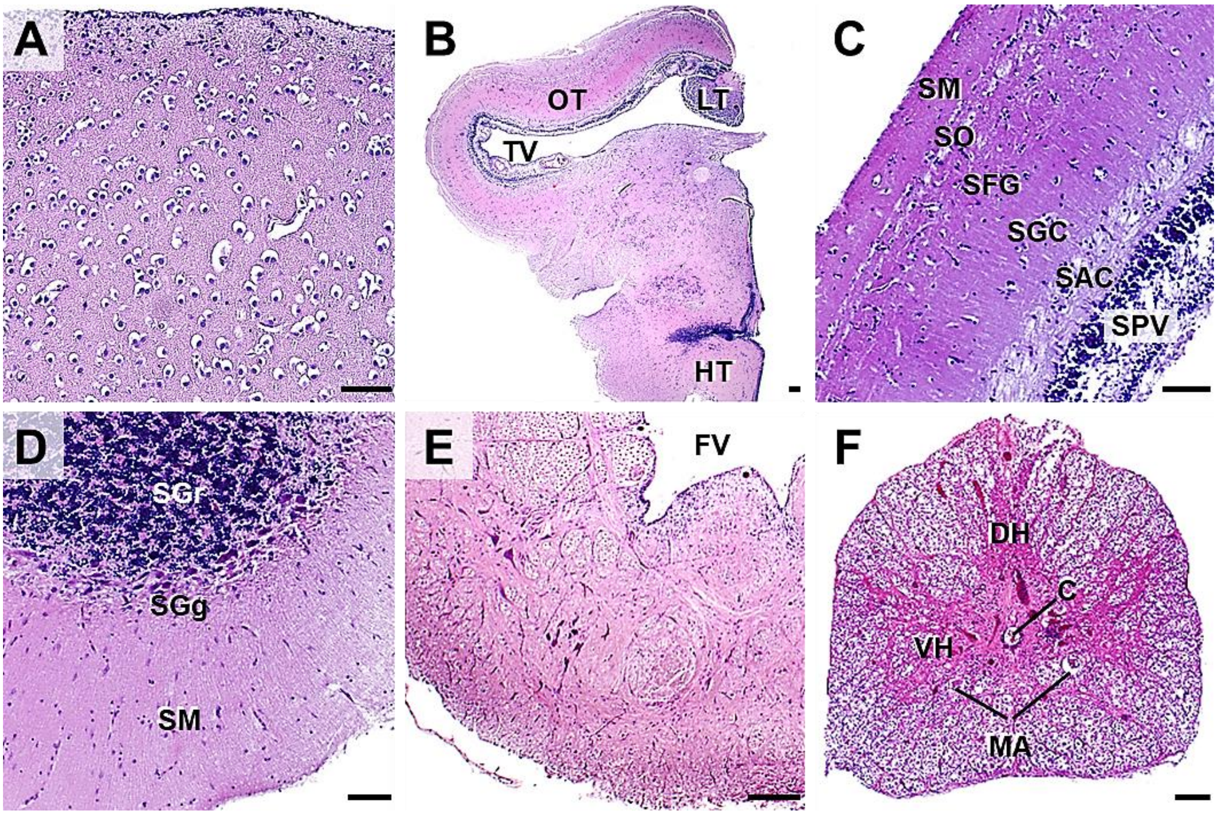

Figure 50. Histology of the rainbow trout brain and spinal cord (transverse sections). A. Pallium (telencephalon). **B.** Mesencephalon with the rostro-dorsal optic tectum (**OT**) and the longitudinal torus (**LT**). The optic tectum provides the roof of the tectal (third) ventricle (**TV**), the tegmentum forms the ventricle floor. Ventrally attached is the hypothalamus (**HT**), which is usually strongly developed in fish and has different regions (*e.g.,* the paraventricular parts as portion of the paraventricular organ composed of excretory cells or the inferior lobe of the hypothalamus). **C.** Detail enlargement of the optic tectum, a structure that mainly receives visual input. The laminae of the optic tectum are indicated: **SM:** Stratum marginale; **SO:** Stratum opticum; **SFG:** Stratum fibrosum et griseum superficiale; **SGC:** Stratum griseum centrale; **SAC:** Stratum album centrale; **SPV:** Stratum periventriculare. **D.** Cortex of the corpus cerebelli with the inner stratum granulosum (**SGr**), the middle stratum ganglionare (**SGg**) and the outer stratum moleculare (**SM**). **E.** Cross-section of the ventral aspect of the medulla oblongata at the level of the 3rd section (compare to **Figure 49B**&**C**). The lateral and ventral aspects are more highly developed, while the dorsal aspect of the fourth ventricle (**FV**) is only limited by thin choroid plexus. **F.** Cross section of the trunk spinal cord, grey and white matter is well demarcated. The dorsal horns of the grey matter are undivided and fuse to a single midsagittal horn, the dorsal and ventral roots fuse to the spinal nerves. Important (histo-) morphological structures are indicated: **DH:** Dorsal horn; **VH:** Ventral horn; **C:** Central canal; **MA:** Axons of the Mauthner cells. FF-PE. HE. Bars = 100 µm.

Time requirements

Approximately 20 minutes are to be scheduled for the extraction and the macroscopic examination of the brain and spinal cord and the sampling and further processing of the histopathological and molecular samples. This estimate does not include the time needed for killing the fish, dissection of other organs/tissues, prearrangement of sampling instruments and materials, or the further processing of fixed/frozen specimens. If vascular perfusion fixation is performed, at least additional 15 minutes should be scheduled.

A comparison of the proposed sampling scheme for routine analyses with previously published studies

Previous publications of ecotoxicological studies that specify the sampling procedure of rainbow trout brain samples in more detail, either describe the sampling of the whole organ [58, 119] or the sampling of individual brain structures (physically) separated from each other, *e.g.,* medulla, cerebellum and optic tecta [120] or preoptic area, telencephalon, optic tectum, hypothalamus, midbrain, cerebellum and hindbrain [121]. In previous ecotoxicological studies examining rainbow trout, the number of analyzed brain tissue samples per fish, if indicated, ranges between one [58, 119] to seven [121] samples.

The number and sizes of tissue samples as well as the sampling locations proposed in the present guidelines are regarded to provide sufficiently representative tissue specimens for the histopathological examination of the most (qualitative) morphological tissue alterations and molecular analyses of the rainbow trout CNS, since they are based on the highly complex brain architecture and the large number of functionally and morphologically different brain regions. The brain is midsagittally halved and the right brain half is transferred to fixative for subsequent histopathological analysis, while the left brain half is sampled for molecular analyses. This approach is advantageous since it enables the collection of molecular and histopathological analysis brain samples from the identical fish. The sampling locations for histopathological analyses of the brain are chosen in such a manner that all brain divisions are available for histopathological analysis.

Note that the histopathological examination of only one hemisphere of the brain requires careful and accurate sample processing. Furthermore, it should be noted that the histopathological examination of only the right hemisphere can falsify the assessment of unilaterally and/or asymmetrically occurring lesions. Therefore, if there is evidence of unilateral lesions (*e.g.,* clinical signs or abnormal necropsy findings) or if the study design requires special investigation of particular brain areas, the sampling protocol may need to be modified accordingly. The sampling of the endocrine organs/tissues associated with the CNS (*i.e.,* the pineal gland, the urophysis and the pituitary gland) is not in the scope of this chapter. For sampling of these structures for histopathological analyses, the interested reader is referred to **Chapter 2.13**.

2.9 Integument: Scaled and non-scaled skin

Relevant anatomical features/preparation

The rainbow trout skin basically resembles that of vertebrates both in the organization (epidermis and dermis) and in function. In addition to the protective function, the skin of teleosts is considered to play an important role *e.g.*, in the maintenance of the ion- and water balance, excretion of nitrogenous waste, sensory perception and communication [2, 42, 122]. The skin surface is covered by the cuticle, *i.e.,* a mucopolysaccharide coating layer which is one component of the skin`s defense system and composed of mucus secreted by superficial epithelial cells and bioactive immune molecules [2, 3, 122, 123]. The epidermis coats the body surface (including the fins) and is a multilayered squamous epithelium of variable thickness, depending on *e.g.,* age, season, sex or body location. In trout, the epidermis is thinner in scaled regions than in non-scaled regions such as *e.g.,* the fins or the head [1, 3, 4, 124]. Unlike in mammals, the cells of all epithelial layers of the epidermis are mitotically and metabolically active [3, 4]. The epidermis of the rainbow trout comprises several cell types, *inter alia* the epithelial (malphigian) cells as main cellular epidermal feature or the exocrine goblet cells (**Figure 53A**) [2, 3, 42]. Separated from the epidermis by an acellular basal lamina lies the dermis, which is composed of an upper stratum spongiosum and a lower stratum compactum [36, 42]. The former is composed of a loose collagen- and reticulin fiber network as well as blood vessels, nerves, different cellular components (*e.g.,* fibroblasts, leukocytes, “scale pocket” cells and pigment cells (chromatophores)) and scales [2, 3, 36, 42]. The stratum compactum is composed of densely packed collagen fiber bundles with few scattered cells (*e.g.,* fibroblasts); its inner boundary is lined by the dermal endothelium. The dermis is delimited from the skeletal muscle by the hypodermis, which is especially prominent in the flank region [1, 2, 36, 42].

The scales are part of the dermal skeleton of fish [2]. In trout, numerous fine and flexible scales originate in the “scale pockets” located in the connective tissue of the dermis, pointing caudally and imbricating each other. The structure of fish scales differs from that of the scales of *e.g.,* reptiles/birds, since they are mineralized dermal collagenous structures instead of keratinized epidermal material. Rainbow trout scales are elasmoid scales (*i.e.,* mainly composed of collagenous tissue, which mineralizes superficially in concentric circles (circuli)) of the cycloid type [1, 2, 42, 125].

At necropsy, the skin samples are generated after excision of the gills and the viscera (except the kidneys); if analysis of the central nervous system is scheduled, the scale-less skin samples are generated in advance. For the generation of skin samples in routine analyses, the rainbow trout is consistently placed on the right body side and the scaled skin samples for subsequent histopathological and molecular analyses are collected from the left body side after evisceration, macroscopic examination and (photo-) documentation. The sample of the scale-less skin is generated from the integument covering the cranial vault (**Figures 51B** and **52A**); the scale-including skin sample is generated at the level of the cranial margin of the dorsal fin and centered between the lateral line and the dorsal median (**Figures 51A** and **52A**). The skin samples for subsequent molecular analyses are excised from the fresh (*i.e.,* unfixed) rainbow trout carcass together with underlying muscle using a biopsy punch. Before the samples are frozen and adequately stored, the skin is separated from the underlying muscle by severing the loose hypodermal fatty tissue (sampling location for molecular analysis of white skeletal muscle and skin is identical, refer to **Chapter 2.10.1**). For generation of skin samples for subsequent histopathological analysis, the carcass (including the kidneys) is trimmed for enhanced tissue fixation by two cross sections (one caudal to the adipose fin removing the tail, the other cranial to the head kidney, removing the head from the body) and removal of the right hypaxial musculature. The remaining trunk is subsequently transferred to neutrally buffered 4% formaldehyde solution. After immersion fixation, the samples for histopathological skin analysis are generated using scalpel and forceps. The remaining trunk (including the kidneys) as well as head, tail and the removed body walls are preserved (*i.e.,* immediately transferred to an adequate fixative), to ensure that sample material is available for new/expanded scientific issues arising from the analyses.

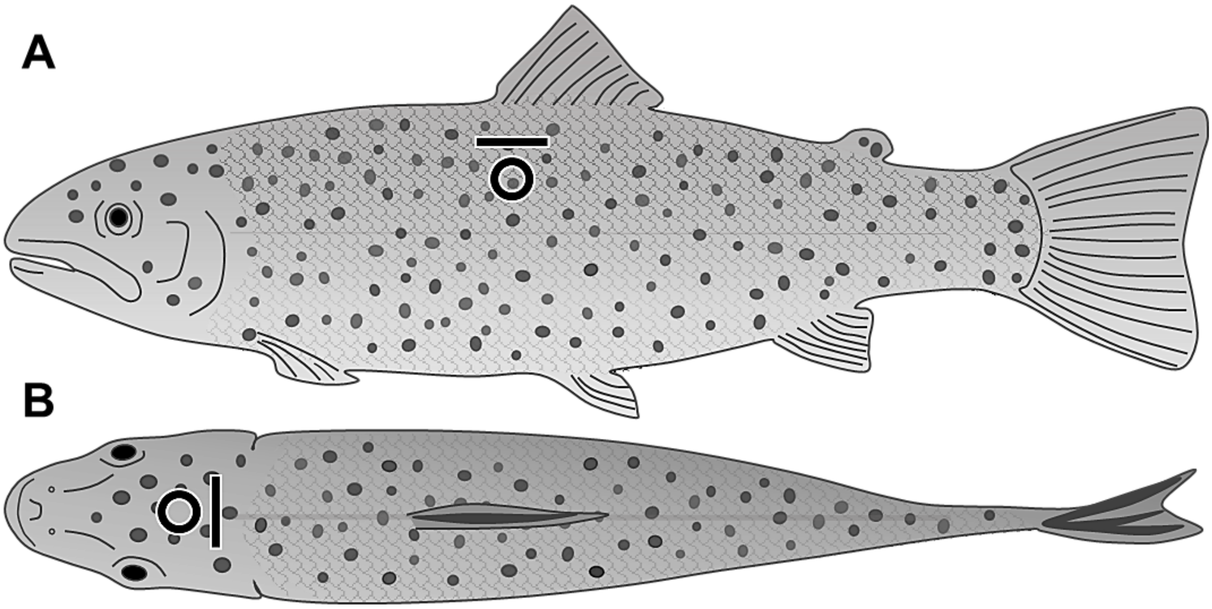

Figure 51. Schematic illustration of sampling for histopathological and molecular analyses of the skin of the rainbow trout. Sample positions of molecular analysis samples (black circles) as well as sample locations and section plane orientations of samples for histopathological analyses (black lines) are indicated. The samples for molecular analyses are excised by biopsy punch from the freshly sacrificed fish after excision of the gills and viscera (except the kidneys). Subsequently, the spine is cut cranially and caudally of the kidney and adjacent hypaxial musculature is spaciously removed for enhanced immersion fixation of head and trunk. Samples for histopathological analyses are generated from the immersion-fixed trunk/head using scalpel and forceps. **A.** Lateral aspect of the left body side of a rainbow trout. The sampling location for molecular and histopathological analysis samples of scale-containing skin is located at the level of the cranial margin of the dorsal fin and centered between the dorsal midline and the lateral line. **B.** Dorsal aspect of the rainbow trout. The sampling locations for molecular and histopathological analysis samples of non-scaled skin are located in the dorsal midline of the skin covering the cranial vault.

General examination parameters

The integument is macroscopically examined for pathological alterations by inspection of the skin covering the head, trunk, tail and fins, corresponding findings are (photo-) documented, if appropriate. Samples for subsequent histopathological, microbiological or parasitological analysis are taken from the altered locations, if required. If mucous coat and scales are to be examined, it is recommended to collected mucus and scales from the dorsolateral fish integument by gently scraping with a spatula from cranial to caudal (refer to Handy et al. [126]) before sampling and immersion fixation, as the cuticle is usually removed during processing for histopathological examinations [123]. Scales can also be used for fish aging [1].

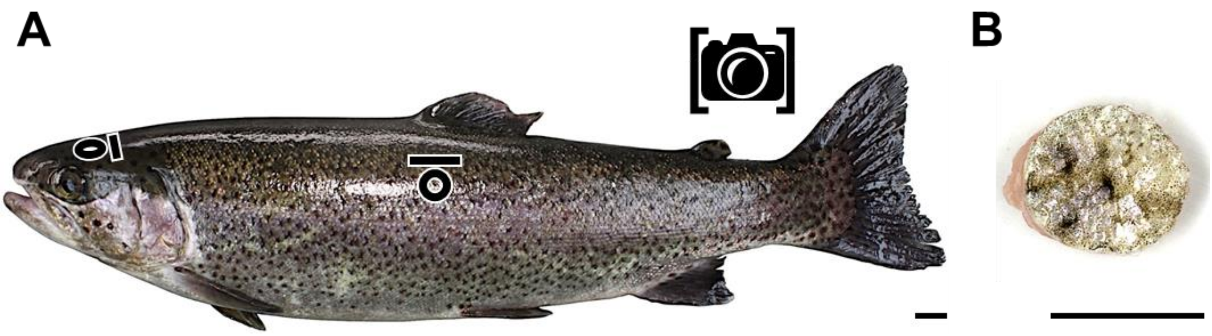

Figure 52. Photographic illustration of the sampling for histopathological and molecular analyses of the skin of the rainbow trout. A. Lateral aspect of the left body side of an intact rainbow trout. (Note that the trout in this figure is remained intact for demonstration purposes, skin specimens are collected after removal of the left abdominal wall, gills and viscera.) Sample positions of molecular analysis samples (black circles) as well as sample locations and section plane orientations of samples for histopathological analyses (black lines) are indicated. The entire integument (*i.e.,* the skin covering the head, trunk, tail and fins) is macroscopically examined for pathological alterations, corresponding findings are (photo-) documented. **B.** Freshly excised scaled skin sample with underlying musculature. The skin samples for molecular analysis are excised by biopsy punch. The skin is subsequently separated from the underlying muscle by severing the loose hypodermal fatty tissue. Bars = 1 cm.

Sampling scheme for routine analyses of the rainbow trout skin

1. Samples for molecular analyses of the skin

Location:	The sampling locations are illustrated in **Figures 51** and **52A**. The sample of the scale-including skin is generated at the midpoint of the imaginary line between the lateral line and the cranial margin of the dorsal fin (**Figure 51A**). The sample of the scale-less skin is generated from the integument covering the cranial vault (**Figure 51B**).
Number of samples:	Two.
Sample size:	Samples are generated using a biopsy punch of 1 cm diameter. For generation of the scaled skin specimen, a block containing skeletal muscle and overlying skin is excised (approx. 1.5 cm x 1.5 cm x 1 cm (length x width x height)), the specimen is placed on the worktop with the skin facing downwards. The biopsy punch is placed on the musculature and not on the skin surface to minimize the loss of scales due to the rotational movement of the biopsy punch.
Remarks:	The skin is separated from the underlying skeletal muscle using scalpel and tweezers.
Processing:	Samples are frozen (liquid nitrogen) and stored at -20°C or -80°C (short-term storage) depending on the intended analysis (chemical or molecular analysis). For prolonged storage of tissue samples for subsequent RNA- and protein analyses, storage at -150°C is recommended.
Downstream analyses:	DNA-, RNA-, protein-, and other OMICS profiling- or targeted analyses requiring cryo-conserved specimen as well as chemical (analytical) analyses*.

**If deemed advantageous for subsequent molecular analyses, mucus is carefully removed using a soft laboratory paper towel (refer to Raj et al. [127]).*

2. Samples for histopathological examination of the skin

Location & orientation of sections:	Sampling locations and orientations are indicated in **Figures 51** and **52A**. The horizontal section of the scale-including skin is generated at the midpoint of the imaginary line between the lateral line and the cranial margin of the dorsal fin (**Figure 51A**). For a satisfactory histopathological scale-containing skin sample it is recommended to remove a block of skin and underlying musculature of approx. 1 cm x 1 cm x 0.5 cm (length x width x height), which is subsequently trimmed to appropriate sample size with straight cutting edges orthogonal to the skin surface. The transverse section of the scale-less skin is generated from the integument covering the cranial vault (**Figure 51B**).
Number of samples:	Two.
Section plane size:	Non-scaled skin: Approx. 1 cm x 0.1 cm (length x width).<br>Scale-containing skin: Approx. 1 cm x 0.5 cm (length x width).
Fixation & embedding:	Non-scaled skin: FF-PE, Scaled skin: FF-GME (formalin-fixed and embedded in glycol methacrylate/methyl methacrylate (GMA/MMA))*.

**If the preconditions for plastic medium embedding are not met, paraffin embedding (as an exception) is suitable after the scale-containing skin sample is fixed in 10% formalin and then decalcified with a slow-acting decalcification solution (e.g., Decalcifier DC1, histological decalcifier, slow-acting, Q Path, VWR Chemicals) for approx. 24-48 hours.*

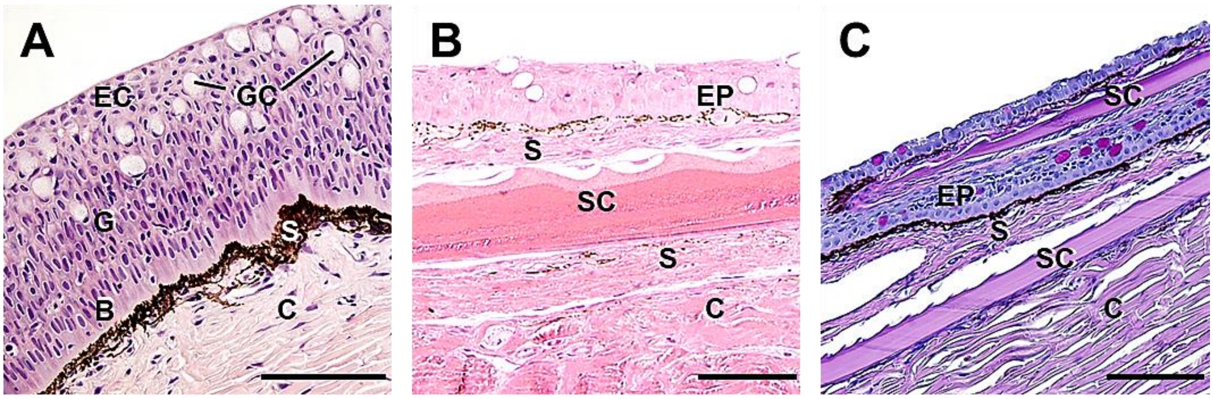

Figure 53. Histology of the rainbow trout skin. A. Transverse section of the non-scaled skin. The multilayered squamous epithelium of the epidermis is composed of a stratum basale (**B**), a stratum germinativum (**G**) and a superficial epithelial cell layer. The epidermis mainly comprises epithelial cells (**EC**), but also mucous goblet cells (**GC**). The epidermis is separated from the dermis by a basal lamina, which is directly adjacent to the underlying stratum spongiosum (**S**) of the dermis. The stratum compactum (**C**), mainly composed of densely packed collagen fiber bundles, is adjoining the stratum spongiosum. FF-PE. HE. **B, C.** Horizontal section of the scale-containing skin. The epidermis (**EP**) is thinner compared to the non-scaled skin covering the cranial vault. Scales (**SC**) originate in “scale pockets” located in the connective tissue of the dermis and point caudally, imbricating each other. Note the bony ridges of the circuli. Beneath the stratum spongiosum (**S**) lies the stratum compactum (**C**). The dermis terminates with a dermal endothelium (*i.e.,* a cell sheet composed of modified fibrocytes) adjacent to the hypodermis (not shown). Note the bright pink staining of the epithelial mucous goblet cells in the periodic acid-Schiff (PAS) staining. FF-GME. HE (**B**). FF-PE. PAS (**C**). Bars = 100 µm.

Time requirements

3-5 minutes are to be scheduled for dissection, macroscopic examination, sampling and further processing of the rainbow trout skin samples for histological and molecular analyses. This estimate does not include the time needed for killing the fish, dissection of gills and viscera, prearrangement of sampling instruments and materials, or the further processing of fixed/frozen specimens.

A comparison of the proposed sampling scheme for routine analyses with previously published studies

Previously published ecotoxicological studies examining rainbow trout skin samples usually sample the skin covering the dorsal parts of the trunk, like the dorsolateral part of the neck [128] or the skin centrally located between the dorsal median and the lateral line with the cranial insertion of the dorsal fin as caudal limit [129, 130]. Sample size in ecotoxicological studies ranges, if indicated, from 0.75 cm^2 [128] to 1 cm^2 [131].

The locations and orientations of the samples for histopathological analyses of the rainbow trout skin in the present guidelines are chosen so that both scaled and non-scaled skin is represented in the histological section. The sample locations of the skin specimens are selected to address the varying composition and difference in the epidermal thickness between scaled and non-scaled skin separately. The number and sizes of tissue samples as well as the sampling locations proposed in the present guidelines are regarded to provide sufficiently representative tissue specimens for the examination of most of the (qualitative) histopathological tissue alterations and molecular analyses of the rainbow trout skin. Since, according to the authors investigations, the explicit examination of rainbow trout scales is not strongly highlighted in ecotoxicological studies, a scale sampling scheme is not included in these guidelines. If deemed necessary, individual scales (rough guide: 12 scales) can easily be removed from the skin located beneath the dorsal fin (refer to Lennquist et al. [132]).

2.10 Locomotor system

2.10.1 Skeletal musculature

Relevant anatomical features/preparation

The rainbow trout skeletal musculature can be subdivided in the cephalic musculature (*i.e.,* the mandibular- and the hyoid muscle plate with the muscles of the cheek region and the hyoid apparatus, as well as the branchial muscle plates with the dorsal and ventral gill arch musculature) and the fin- and body musculature. The latter is subdivided in the ventral hypaxial-, the dorsal epaxial musculature and a lateral superficial muscle [2, 133]. The striated musculature of the trunk- and caudal region (accounting for over 50% of the total body weight) is moderately perfused and composed of myomeres (*i.e.,* w-shaped folded and interlocked blocks of muscle tissue, separated by tendinous myocommata (*i.e.,* myosepta) and one interdigitating with each other), symmetrically arranged on the left and right side of the body (**Figure 54B**) [2, 4, 25, 42]. Two tendinous myosepta separate the body musculature in four quadrants. The dorsal epaxial and the ventral hypaxial musculature of the trunk are separated by one horizontal myoseptum. The left and right side of the trunk musculature are separated by one median vertical myoseptum (**Figure 55B**) [2, 3, 25]. The streamlined shape of the fish as well as the arrangement of the muscle fibers in myomeres enable the fish to move through the water by body undulations. The myomeres are folded and interlocked in a way that muscle contraction is transferred to adjacent myomeres and wide parts of the vertebral column. This arrangement allows strong lateral undulating body movements [2, 3, 25]. The epaxial and hypaxial myomeres are composed of white fast-contracting muscle fibers, whereas the highly vascularized lateral superficial musculature consists of red slow-contracting muscle fibers [1-4, 134]. The lateral superficial muscle is a wedge-shaped band of muscle fibers located on both body sides beneath the lateral line organ (*i.e.,* superior to the epaxial and the hypaxial muscles at the level of the horizontal septum) (**Figure 55B**). This red muscle is designed for long-lasting activities, has a high lipid content and is rich in myoglobin [1, 2, 4, 135]. In salmonids, there are also pink muscle fibers located between the white and red muscle fiber divisions (**Figure 55B**) [4, 135].

At necropsy, the skeletal muscle samples are generated after excision of the gills, the viscera (except the kidneys) and the central nervous system (CNS), the muscle samples are generated together with the samples for skin and lateral line analyses. After evisceration, macroscopic examination and (photo-) documentation, the samples for histopathological and molecular analyses of white and red skeletal musculature are taken (the white muscle sample for molecular analyses is collected together with sample for molecular analyses of the scaled skin (**Chapter 2.9**), the specimen for histopathological analyses of the red muscle is identical with the sample for histopathological analysis of the lateral line system (**Chapter 2.12.3**). For generation of muscle samples in routine analyses, the rainbow trout is consistently placed on the right body side and samples are collected from the left body side. White musculature specimens are taken from the epaxial muscles, the samples of the red (dark) musculature are generated from the lateral superficial muscle (**Figure 54A**). The muscle samples for subsequent molecular analyses are excised from the fresh (*i.e.,* unfixed) rainbow trout carcass using a biopsy punch of 1 cm diameter. Before the samples are frozen and adequately stored, the skin is separated from the muscle samples by severing the loose hypodermal tissue. For generation of muscle samples for subsequent histopathological analyses, the remaining carcass (including the kidneys) is trimmed for enhanced tissue fixation by two cross sections (one cranial to the head kidney, removing the head from the body and one caudal to the adipose fin removing the tail) and removal of the right hypaxial musculature and is subsequently transferred to neutrally buffered 4% formaldehyde solution. After immersion fixation of the trunk, the samples for histopathological muscle analyses are generated using scalpel and forceps. The remaining trunk (including the kidneys), head, tail and the removed body walls are preserved (*i.e.,* immediately transferred to an adequate fixative), to ensure that sample material is available for new/expanded scientific issues arising from the analyses.

2.10.1 Skeletal musculature

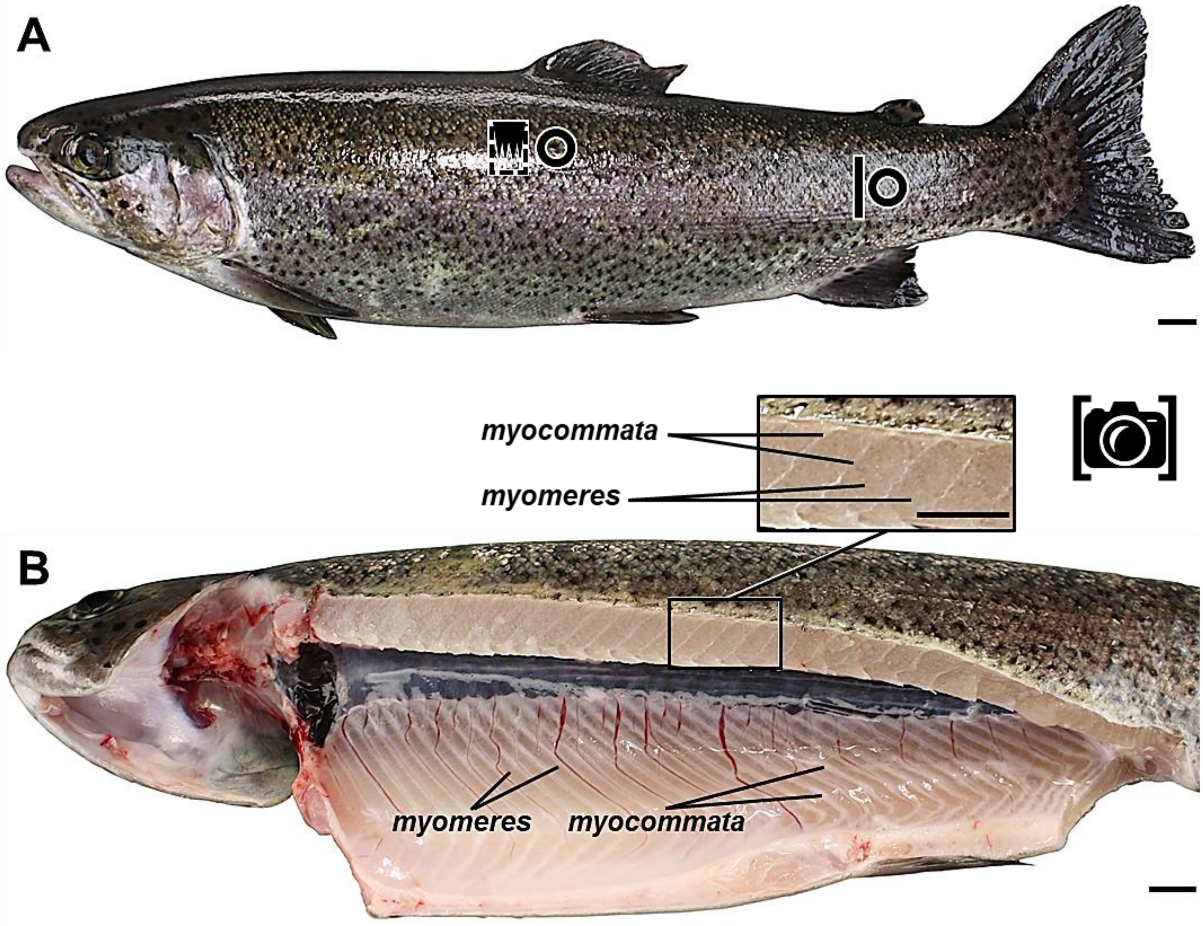

Figure 54. Photographic illustration of the skeletal musculature sampling sites for histopathological and molecular analyses. A. Lateral aspect of the left body side of a freshly sacrificed, unfixed rainbow trout. (Note that the muscle specimens are collected after removal of the left abdominal wall, gills, viscera and CNS. For demonstration purposes the fish shown in the present figure is remained intact.) Sample positions of molecular analysis samples (black circles) as well as sample locations and section plane orientations of samples for histopathological analyses (black line: cross section of the red musculature; white dotted rectangle with triangles: parasagittal section of the white musculature) are indicated. The samples for molecular analyses are excised from the freshly killed and eviscerated fish using a biopsy punch. Subsequently the spine is cut cranially and caudally of the kidneys and adjacent hypaxial muscles are spaciously removed for enhanced immersion fixation. Samples for histopathological analyses are generated from the immersion-fixed trunk using scalpel and forceps. **B.** Ventro-lateral aspect of the necropsy situs after removal of the gills and the viscera (except the kidneys). The myomeres and myocommata of the hypaxial musculature can be assessed beneath the transparent peritoneum. The white skeletal musculature is macroscopically examined for pathological alterations, corresponding findings are (photo-) documented. The detail enlargement shows the organization of the hypaxial musculature in myomeres, separated by myocommata. Bars = 1 cm.

General examination parameters

The white skeletal trunk musculature is macroscopically examined for pathological alterations by inspection of the hypaxial musculature of the left and right abdominal wall (*i.e.,* by inspection of the walls of the peritoneal cavity (refer to **Figure 54B**) as well as of the removed hypaxial muscle of the left body side). If required, conspicuous findings are photographed and additional samples for subsequent histopathological, molecular or microbiological analysis are taken from the altered locations.

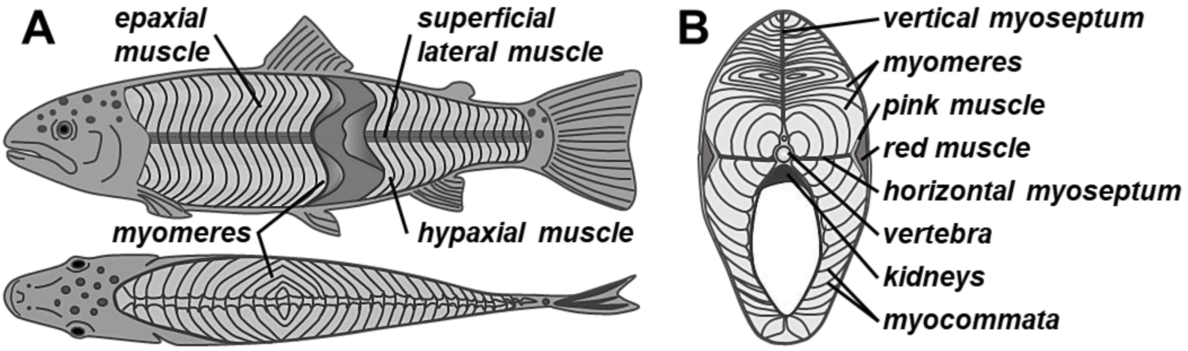

Figure 55. Schematic illustration of the rainbow trout musculature. A. Upper image: Lateral aspect of the trunk- and caudal skeletal musculature. The overlying skin is removed and the myomeres of the epaxial and hypaxial white muscles are visible. Two myomeres are removed to demonstrate the three-dimensional w-shaped structure of the axial myomeres (*i.e.,* two thin arms extend antero-dorsally and antero-ventrally, the central large cone points anteriorly, the two outer smaller cones point posteriorly. Laterally, the white musculature is covered by a band of red muscle fibers at the level of the horizontal septum, *i.e.,* the superficial lateral muscle. Lower image: Dorsal aspect of the exposed myomeres of the epaxial musculature. **B.** Cross section through the trunk musculature. The white trunk musculature is organized in myomeres, separated by tendinous myocommata. The dorsal muscle portion (*i.e.,* the epaxial muscle) is separated from the ventral muscle portion (*i.e.,* the hypaxial muscle) by the horizontal myoseptum, while the vertical myoseptum divides the musculature of the left and right body side of the fish. The red superficial lateral muscle is a wedge-shaped band of muscle fibers located on both body sides beneath the lateral line organ. In salmonids, there are also pink muscle fibers located between the white and red muscle compartments.

Sampling scheme for routine analyses of the rainbow trout skeletal muscles

1. Samples for molecular analyses of the skeletal muscles

Location:	Sample locations are illustrated in **Figure 54A**. The white muscle sample is excised from the left dorsal epaxial musculature, in the midpoint of the imaginary line between the lateral line and the cranial margin of the dorsal fin. The red muscle sample is taken from the left lateral superficial muscle at the level of the adipose fin.
Number of samples:	Two.
Sample size:	Samples are generated using a cylindrical biopsy punch of 1 cm diameter. If the skin and scales are to be preserved, a block containing skeletal muscle and overlying skin is excised (approx. 1.5 cm x 1.5 cm x 1 cm (length x width x height)) and the specimen is placed on the worktop with the skin facing downwards so that the biopsy punch is placed on the musculature and not on the skin surface. The sample height (after removal of the skin) is ~1 cm for the epaxial muscle specimen and ~0.5 cm for the lateral superficial muscle.
Remarks:	Homogenous muscle samples are generated, the skin is separated from the muscle samples using scalpel and tweezers.
Processing:	Samples are frozen (liquid nitrogen) and stored at -20°C or -80°C (short-term storage) depending on the intended analysis (chemical or molecular analysis). For prolonged storage of tissue samples for subsequent RNA- and protein analyses, storage at -150°C is recommended.
Downstream analyses:	DNA-, RNA-, protein-, and other OMICS profiling- or targeted analyses requiring cryo-conserved specimen as well as chemical (analytical) analyses.

2. Samples for histopathological examination of the skeletal muscles

Location & orientation of sections:	Sampling locations and orientations are indicated in **Figure 54A**. A transverse section of the (red) superficial longitudinal muscle is generated, including the skin and the lateral line canal. A parasagittal section of the (white) dorsal epaxial musculature is generated, including the overlying skin. Tissue blocks of ~1.5 cm x 1 cm x 0.7 cm (length x width x height) are generated.
Number of samples:	Two.
Section plane size:	Approximately 1.5 cm x 1 cm (length x width).
Fixation & embedding:	FF-PE.

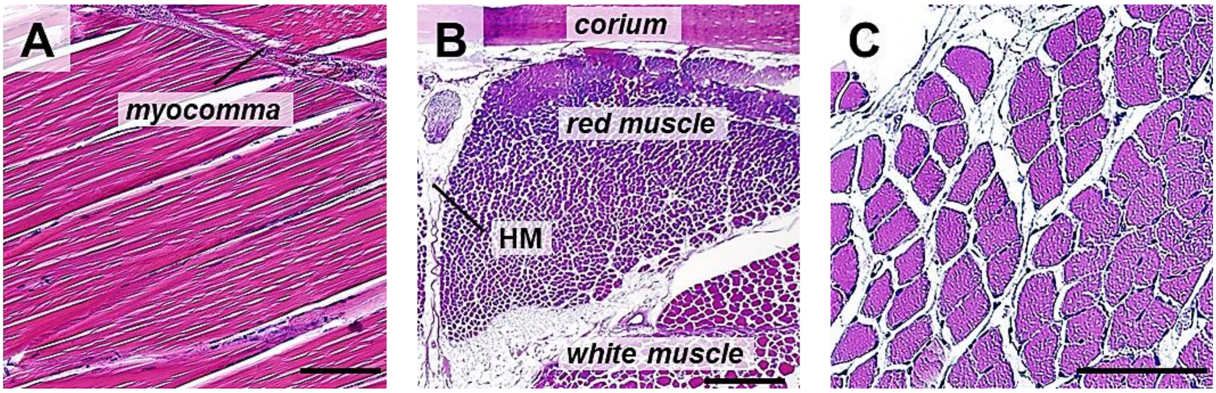

Figure 56. Histology of the rainbow trout skeletal musculature. A. Longitudinally sectioned white muscle fibers, separated by myocommata. **B.** Cross section of the red muscle fibers of the lateral superficial muscle, adjacent to the horizontal myoseptum (**HM**), flanked by the corium of the integument and the white skeletal musculature on the other side. **C.** Cross section of the red muscle fibers. FF-PE. HE. Bars = 100 µm in **A**&**C** and = 500 µm in **B**.

Time requirements

Approximately 5 minutes are to be scheduled for dissection, macroscopic examination, sampling and further processing of the histological and molecular samples of the rainbow trout skeletal musculature. This estimate does not include the time needed for killing the fish, dissection of gills, viscera or CNS, prearrangement of sampling instruments and materials, or the further processing of fixed/frozen specimens.

A comparison of the proposed sampling scheme for routine analyses with previously published studies

Previously published ecotoxicological studies examining rainbow trout samples of white muscle usually sample the dorsal musculature [136, 137]. If indicated more precisely, the epaxial muscle is sampled adjacent to the anterior dorsal fin insertion [138] or behind the dorsal fin [139]. Red muscle tissue is sampled between the pectoral and caudal fins from the lateral line [139]. Sample weight ranges from 0.8-1 g [139] up to 10 g [63]. The locations and orientations of the samples for histopathological analyses of the rainbow trout skeletal musculature are chosen so that white and red muscle fibers are represented in the histological sections. The sample locations of the specimens for molecular analyses are selected to address differently composed and vascularized muscle tissue compartments of white and red muscle separately. The location for the red muscle sample was chosen according to the publication of Lefèvre et al. [140], which states that red muscle is most abundant at the level of the adipose fin. The number and sizes of tissue samples as well as the sampling locations proposed in the present guidelines are regarded to provide sufficiently representative tissue specimens for the examination of most of the (qualitative) morphological tissue alterations and molecular analyses of the rainbow trout skeletal musculature.

2.10.2 Bones and cartilage

Relevant anatomical features/preparation

The **skeleton** of the rainbow trout is composed of the head skeleton (*i.e.,* the chondro-/neurocranium as the primary braincase, the bones of the viscero-/splanchnocranium (with *e.g.,* the hyoid bars or the branchial arches) and the bones of the dermatocranium (*e.g.,* the opercular bones)), the vertebral column, and the appendicular skeleton (*i.e.,* the osseous fin support system and the pectoral- and pelvic girdle, which articulate with the paired pectoral or rather pelvic fin support system) [2, 42, 141, 142]. An additional part of the trout`s musculoskeletal system are the intermuscular fish bones (*e.g.,* the dorsal or epineural ribs) [2, 42, 142]. In contrast to real ribs, these structures are located in the myoseptal connective tissue and are built by desmal- instead of chondral ossification [2, 42]. The rainbow trout skeleton is of various functions, such as the protection of the viscera, locomotion, buoyancy or maintenance of physiological processes (*e.g.,* calcium homeostasis) [2]. An important feature of the fish skeleton is the lack of hematopoietic bone marrow. The medullary cavities found in some bones (*e.g.,* the vertebrae or the bones of the cranium) are filled with adipose tissue [2, 4, 42]. Bone formation in fish occurs either as the (perichondral and endochondral) ossification of a hyaline cartilaginous template (*i.e.,* the endoskeleton) or the direct formation of bony structures in the dermis without a cartilaginous template (*i.e.,* the dermal skeleton). Examples for dermal bones are the bones of the opercular series or fin rays [1, 2, 141]. In salmonids, the skeleton is composed of cellular bones, consisting of osteocytes in lacunae surrounded by ossified bone matrix [1, 36, 42].

In bony fishes, there is a huge range of **cartilaginous tissues**, most of them not resembling the “classic” hyaline mammalian cartilage [143-145]. The teleost cartilaginous tissue is subdivided into several categories with according subtypes. Each category has special histological staining characteristics and light microscopy appearance by which it can be identified. The fish cartilage types also differ in their matrix macromolecules [2, 143-146]. Some cartilage types are permanent and persist in adult fish, while others are degraded due to ossification process [2, 145, 147].

At necropsy, bone samples are generated after excision of the gills, the viscera (except the kidneys) and the sampling of muscles and skin. If analysis of the central nervous system (CNS) is also scheduled, the samples of the vertebral bone (**Figures 57** and **58**) are generated after sampling the spinal cord. For sampling of the spinal cord, the neural spines of the vertebrae are carefully removed up to the level of the adipose fin, so that both the spinal cord can be sampled and the vertebrae caudal to the adipose fin remain intact as specimens for histopathological (and molecular) bone analyses (**Figure 58B**&**C**). The sample for subsequent molecular bone analysis (together with cartilage-related tissue *i.e.,* the notochord) is excised from the freshly sacrificed rainbow trout by two cross sections immediately posterior to the adipose fin, using scalpel and/or knife. Tissue adhering to the vertebrae is removed using scalpel and scissors, and if compatible with the analysis method, the bone sample with adhering tissue can be placed in 30% hydrogen peroxide for approximately 5 minutes in advance [148]. For generation of samples for histopathological bone analyses, the samples are generated after immersion fixation of the head and tail in neutrally buffered 4% formaldehyde solution. For an enhanced immersion fixation (and to ensure that sample material is available for further analysis of other organs/tissues or new/expanded scientific issues arising from the analyses), the carcass is trimmed by a cross section cranial to the head kidney removing the head from the trunk and removal of the right hypaxial musculature, before transfer to neutrally buffered 4% formaldehyde solution. Trunk, head, tail (with caudal fin) and the removed body walls are preserved (*i.e.,* immediately transferred to an adequate fixative). For histopathological analysis of hyaline cartilage, the outermost (first) gill of the right body side is removed as a whole and immersion-fixed, subsequently the gill arch is separated from the holobranch and midsagittally sectioned (relative to the gill axis, refer to **Figures 57C** and **58D**). If gill analyses are scheduled, the first gill arch is separated from the holobranch after sampling for molecular gill analyses and subsequently transferred to neutrally buffered 4% formaldehyde solution.

2.10.2 Bones and cartilage

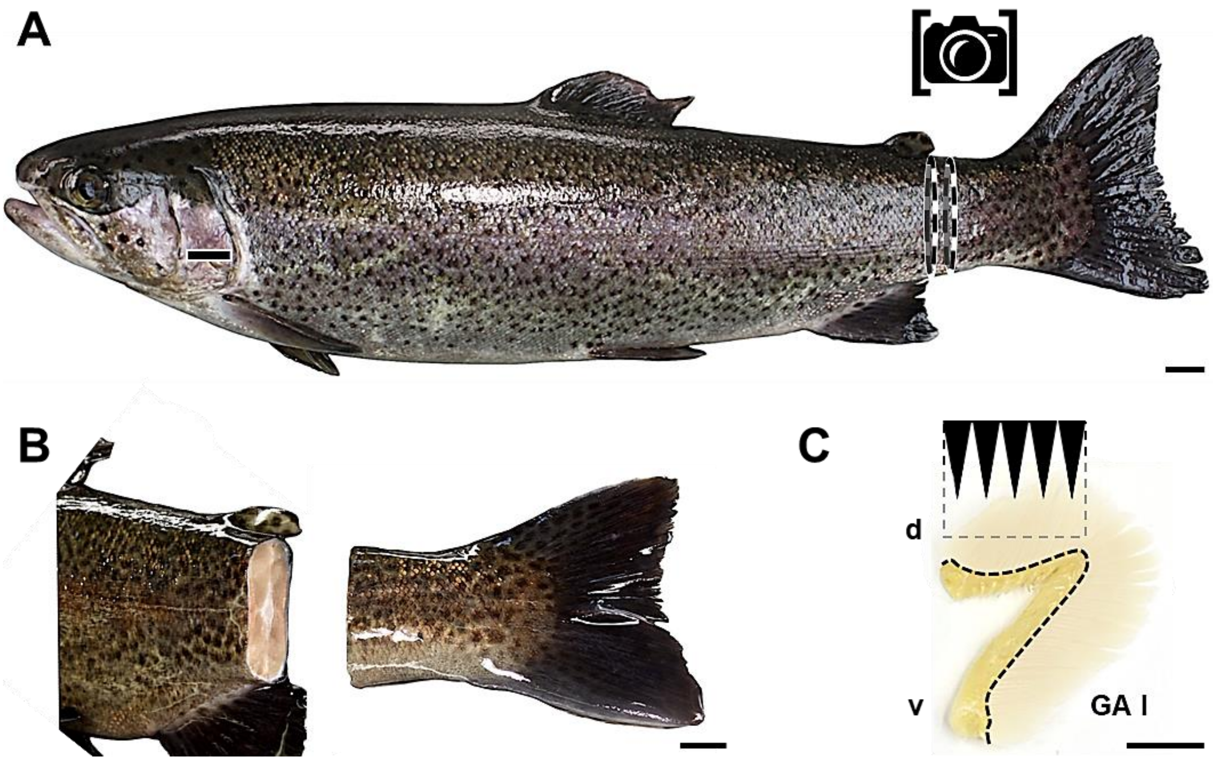

Figure 57. Photographic illustration of the sample locations for histopathological and molecular analyses of the skeleton of the rainbow trout. A, B. Sampling of the dermal and endochondral bones. After sampling of the gills, viscera (except the kidneys), CNS, muscles and skin, the operculum as well as the vertebrae are sampled for subsequent analyses, to adequately address the two bone types of the rainbow trout (*i.e.,* dermal bones or bones of the endoskeleton). The location and orientation of the histopathological sample section plane of the dermal bone is indicated (black line) in **A**. For sampling of the vertebrae, the caudal body portion is separated from the trunk by a cross section caudal to the adipose fin (indicated by the anterior dashed circle). A slab of fresh (*i.e.,* unfixed) tissue of ~1 cm thickness and containing trunk musculature and vertebrae is subsequently generated by means of a second cross section (indicated by the posterior dashed circle). The specimen containing trunk musculature and vertebrae is subsequently trimmed as illustrated in **Figure 58B**. The bone samples for subsequent histopathological analyses are generated after immersion fixation in neutrally buffered 4% formaldehyde solution of the trimmed carcass. The sample for histopathological analysis of the vertebral bone is generated from the separated caudal portion at the level of the posterior dashed circle, a cross section through the vertebra is generated. **C.** Medial aspect of the dissected, formalin-fixed first gill of the right body side (**GA I**). The gill arch is separated from the holobranchs (dashed black line) and sagittally sectioned (relative to the plane of the gill arch). The dashed rectangle with the black triangles is indicating the section plane orientation parallel to the picture plane. **d:** Dorsal; **v:** Ventral. Bars = 1 cm.

General examination parameters

All fins, the skull and the trunk are macroscopically examined for pathological alterations that indicate pathological changes in the skeletal system (*e.g.,* asymmetries, circumferential growth or axis deviations), corresponding findings are (photo-) documented and bones of altered locations are exposed and examined in more detail. While trimming the rainbow trout trunk or generation of CNS samples, portions of the skull and the vertebral column are exposed and available for macroscopic examination. Samples for subsequent histopathological and molecular or microbiological analyses are taken from the altered locations, if required.

Sampling scheme for routine analyses of the rainbow trout bone and cartilage

1. Sample for molecular analyses of the bone and cartilage

Location:	Sample location is illustrated in **Figure 58A**&**B**. The sample for molecular analysis is generated from the centrum of the vertebrae included in the trunk tissue slab cut out posterior to the adipose fin.
Number of samples:	One.
Sample size:	The sample is generated using scalpel/knife and forceps. Sample size is approximately 0.5 cm x 0.3 cm x 0.3 cm (length x width x height).
Remarks:	A bone (and notochord) sample is generated, adhering muscles, vessels, spinal cord and connective tissue are removed.
Processing:	Sample is frozen (liquid nitrogen) and stored at -20°C or -80°C (short-term storage) depending on the intended analysis (chemical or molecular analysis). For prolonged storage of the tissue sample for subsequent RNA- and protein analyses, storage at -150°C is recommended.
Downstream analyses:	DNA-, RNA-, protein-, and other OMICS profiling- or targeted analyses requiring cryo-conserved specimen as well as chemical (analytical) analyses.

2. Samples for histopathological examination of the bone and cartilage

Location & orientation of sections:	Sampling locations and orientations are indicated in **Figures 57** and **58C**&**D**. A horizontal section of the dermal bone of the opercular series is generated at the level of the most caudally extending part of the convex posterior margin of the gill cover. The section includes the full thickness of the operculum.<br>A cross section of the bone of the vertebral body (built by endochondral ossification) is generated.<br>For histopathological analysis of the cartilage, a midsagittal section of the gill arch of the first right gill is generated.
Number of samples:	Three.
Section plane size:	Approximately 1.2 cm x 0.1 cm (length x width) for the section of the operculum and 1.5 cm x 1.5 cm (length x width) for the vertebra section. The isolated gill arch is sectioned in its entirety.
Fixation & embedding:	FF-PE*.

**Due to the mineralized bone tissue, the samples are decalcified with a slow-acting decalcification solution for approximately 24-48 hours (opercular and gill arch specimen) and approximately 4-5 days (vertebral specimen).*

2.10.2 Bones and cartilage

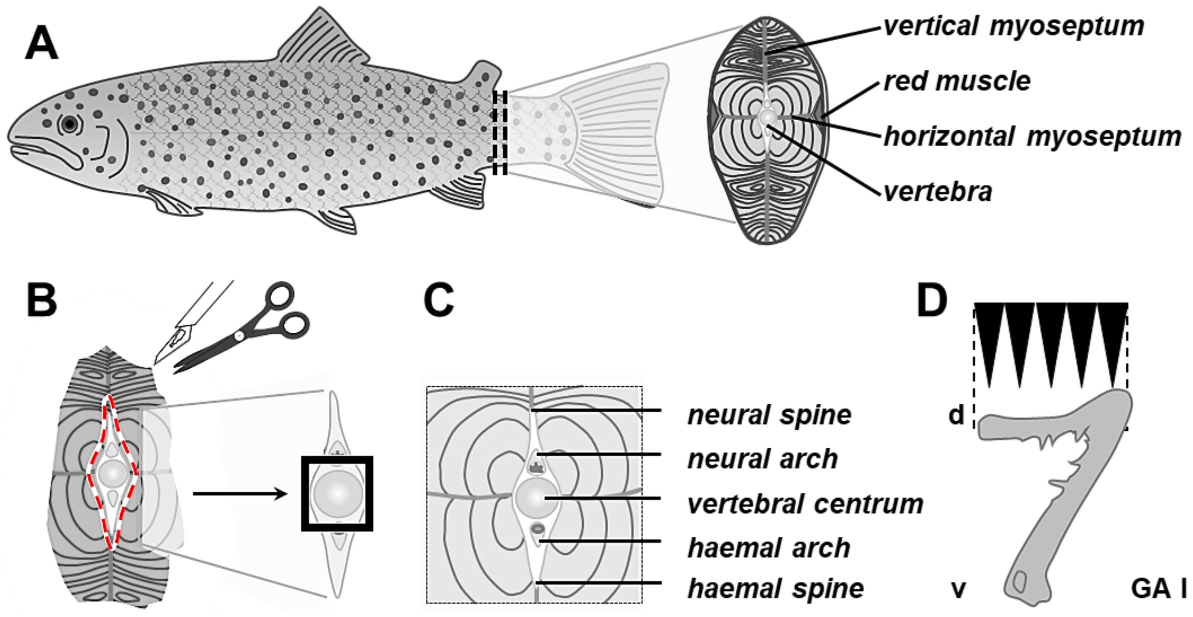

Figure 58. Schematic illustration of the sampling for histopathological and molecular analyses of the rainbow trout vertebral bone and hyaline cartilage. A. As illustrated in **Figure 57**, two cross sections are performed on the trunk caudal to the adipose fin. **B.** The trunk tissue slab, containing red and white trunk musculature and vertebrae, is processed in fresh, unfixed condition. Adhering trunk musculature and connective tissue is removed. The haemal and neural spines (and therefore the neural arch containing the spinal cord and the haemal arch containing the dorsal aorta) are removed, so that the remaining centrum (*i.e.,* bone and notochord tissue) is processed for molecular analyses (sample location is indicated by the black rectangle). **C.** After immersion fixation of the tail, a second tissue slab (thickness ~0.3 cm) is sectioned (*i.e.,* posterior to the former cross section) and a square-shaped transversal tissue specimen containing a section of the entire vertebral profile is excised for histopathological analysis. **D.** Medial aspect of the dissected gill arch, separated from the gill filaments of the first gill arch of the right body side (**GA I**). The holobranch and the adhering soft tissue are removed and the immersion-fixed gill arch is sagittally sectioned (relative to the plane of the gill arch). The dashed rectangle with the black triangles is indicating the section plane orientation parallel to the picture plane. **d:** Dorsal; **v**: Ventral.

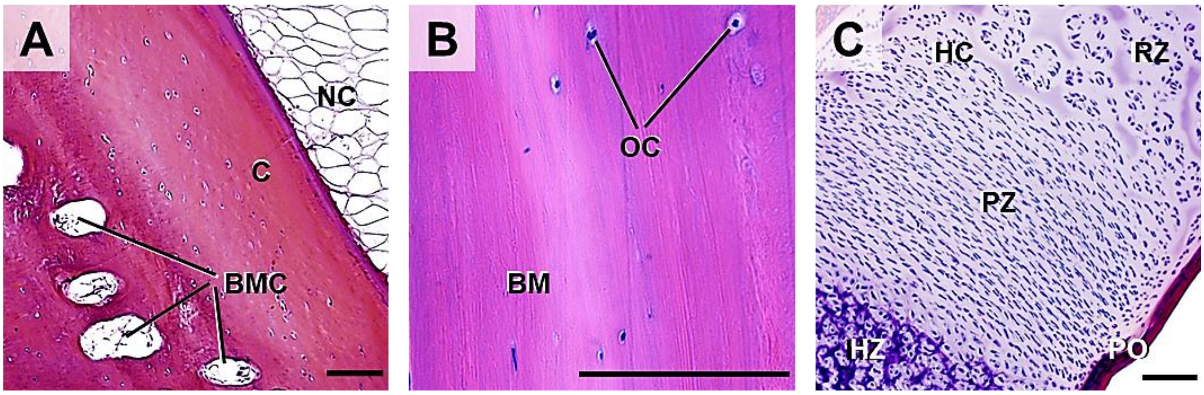

Figure 59. Histology of the rainbow trout bones. A. Transverse section of the vertebral body. The spongy vertebral bone is built by endochondral ossification. In the center of the vertebral body lies the notochord (**NC**) (*i.e.,* a special primitive cartilage and an intermediate form of cartilage and connective tissue [1]), which is delineated from the surrounding bone of the vertebral centrum (**C**) by chorda sheats. The bone medullary cavities (**BMC**) are filled with adipose tissue. **B.** Horizontal section of the opercular bone. The dermal bones of the opercular series are part of the cover of the orobranchial chamber and are overlaid by non-scaled skin on the outer surface. The opercular bones are flat bones comprising little to no bone cavities. A few osteocytes (**OC**) in their lacunae are present in the ossified bone matrix (**BM**). The bone of the salmonids is of the cellular type. **C.** Sagittal section of the gill arch. The supporting gill arch is composed of hyaline cartilage (**HC**), here a cell-rich hyaline cartilage is shown, surrounded by a bone collar due to perichondral ossification (**PO**). The cartilage is undergoing hypertrophy and resorption; a resting zone (**RZ**), a proliferation zone (**PZ**) and a zone of hypertrophic chondrocytes (**HZ**) is visible. In the lower left corner, chondrocyte hypertrophy due to cartilage degradation in the context of endochondral ossification is present. FF-PE. HE. Bars = 100 µm.

Time requirements

Approximately 5-7 minutes are to be scheduled for dissection, macroscopic examination, sampling and further processing of the histopathological and molecular samples of the rainbow trout bones. This estimate does not include the time needed for killing the fish, dissection of gills, viscera, CNS and skin- and muscle specimens, prearrangement of sampling instruments and materials, or the further processing of fixed/frozen specimens.

A comparison of the proposed sampling scheme for routine analyses with previously published studies

According to the authors investigations, previously published ecotoxicological studies examining trout bone tissue – if indicated – *inter alia* analyze the carcass [149, 150] or the vertebrae [136, 148], without distinct information regarding sample number and size.

The sampling locations of the specimens for molecular analyses as well as the locations and orientations of the samples for histopathological analyses of the rainbow trout bones are chosen so that both types of bone (*i.e.,* dermal bone and bone of the endoskeleton) are represented and addressed separately. The number and sizes of tissue samples as well as the sampling locations proposed in the present guidelines are regarded to provide sufficiently representative tissue specimens for the examination of most of the (qualitative) morphological tissue alterations and molecular analyses of the rainbow trout bones. For histopathological cartilage analysis, the gill arch specimen and the section plane orientation are chosen so that several subtypes of hyaline cartilage can be assessed. If the study design requires the examination of additional categories of cartilage, cartilaginous tissue can also be examined in the histopathological sections of *e.g.,* the gills (**Chapter 2.1**), the tongue (**Chapter 2.3.1**) or the eye ball (**Chapter 2.12.4**). Given that isolated examination of cartilage tissue has not yet played a dedicated role in ecotoxicological studies using rainbow trout, these guidelines are limited to the histopathological examination of cartilage tissue. For a more extensive analysis of cartilage tissue, the sampling protocol must be adapted accordingly by the investigator.

2.10.3 Fins

Relevant anatomical features/preparation

The body of the rainbow trout is subdivided in the head-, trunk- and tail region. The paired pelvic and pectoral fins and the unpaired dorsal, anal and caudal fins (**Figure 60**) represent the extremities of the trout. In salmonids, there is a second dorsal fin (*i.e.,* the adipose fin) which lacks fin rays and is no true extremity [2, 42]. Fins are important features for body stabilization, propulsion and maneuvering locomotion [2, 25, 151]. The fleshy adipose fin is suggested as a flow sensory organ with hydrodynamic function [152, 153]. Additionally, the adipose fin of *Oncorhynchus* species is sexually dimorphic and a secondary sexual characteristic [154, 155]. All fins (except for the adipose fin) are supported by muscles (body and intrinsic fin musculature) and bony structures such as the flexible fin rays (lepidotrichia), which are composed of paired hemitrichia [2, 3, 151]. The median unpaired dorsal and anal fin are supported by the pterygiophores, *i.e.,* bones or cartilaginous structures, located between the neural or haemal vertebral spines and extending into the fins, articulating with the fin rays [2]. The pectoral fins originate bilaterally on the cranio-ventral trunk and are attached to the pectoral girdle by a group of bony pectoral radials, which articulate with the osseous pectoral fin support system [2, 42]. The pelvic fins are bilaterally attached to the pelvic by the fin rays, which insert at the basipterygia of the pelvic girdle located in the hypaxial trunk musculature [42, 151]. The caudal fin is supported by the structurally modified caudal vertebrae; the hypurals (*i.e.,* haemal arches, modified to flat, bony plates) articulate with the caudal fin rays [2].

At necropsy, fin samples are generated after excision of the gills, the viscera (except the kidneys), the central nervous system (CNS) and the sampling of skin, musculature and bones. After evisceration, macroscopic examination and (photo-) documentation, the samples for histopathological and molecular analyses are collected from the adipose fin and the caudal fin (**Figure 60**). Fin samples for molecular analyses are excised from the fresh (*i.e.,* unfixed) fish using scissors and forceps. Fin samples for histopathological analyses are excised after immersion fixation of the trunk and tail in neutrally buffered 4% formaldehyde solution, using scalpel and forceps. For enhanced immersion fixation and to ensure that sample material is available for further analysis of other organs/tissues (*e.g.,* kidney), the remaining carcass (after evisceration and removal of the gills, the viscera and the CNS and the sampling for molecular analyses (*e.g.,* of skin, muscles, bones and fins) is trimmed by two transverse sections (one cranial to the head kidney, removing the head from the body and one caudal to the adipose fin removing the tail) and removal of the right hypaxial musculature and subsequently transferred to neutrally buffered 4% formaldehyde solution. Trunk, head, tail (with caudal fin) and the removed body walls are preserved (*i.e.,* immediately transferred to an adequate fixative) after sampling, to ensure that sufficient sample material is available for new or expanded scientific issues arising from the analyses.

2.10.3 Fins

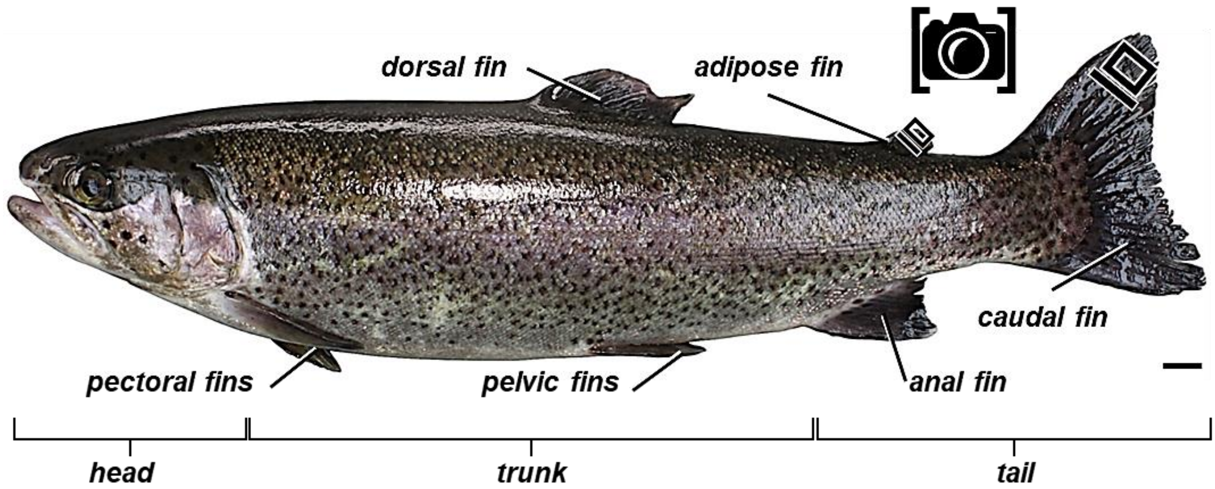

Figure 60. Photographic illustration of the fins of the rainbow trout and the sampling for histopathological and molecular fin analyses. The fusiformly shaped rainbow trout body is separated in 3 body regions: head (from the snout to the caudal margin of the operculum), trunk (from the caudal margin of the operculum to the urogenital papilla) and tail region (from the urogenital papilla to the caudal margin of the caudal fin). All fins except the adipose fin are essential features for body stabilization and maneuvering locomotion and are supported by trunk- and fin musculature and bony elements, such as the flexible fin rays. The fleshy, non-rayed adipose fin is of mechanosensory and hydrodynamic function. Additionally, it is sexual dimorphic in salmonids and a secondary sexual characteristic. Note, that the fin specimens are collected after removal of the left abdominal wall, gills, viscera (except the kidneys) and CNS, for demonstration purposes, the fish shown in the present figure is remained intact. Sample positions of molecular analysis samples (black rectangles) as well as sample locations and section plane orientations of samples for histopathological analyses (black lines) are indicated. The samples for molecular analyses are excised from the freshly sacrificed and eviscerated fish using scissors, subsequently the trunk is cut cranially and caudally to the kidneys and hypaxial muscles are spaciously removed for enhanced immersion fixation. Samples for histopathological analyses are generated from the immersion-fixed trunk and tail using scalpel and forceps. Bar = 1 cm.

General examination parameters

All fins are macroscopically examined for pathological alterations, corresponding findings are (photo-) documented, if appropriate. Particular attention is paid to the occurrence of fin erosion, which appeared as a consequence of aquatic pollution in several studies [156]. It should be noted, however, that fin erosions can be due to multiple other causes, such as physical injury or infectious conditions [4]. Samples for subsequent histopathological and microbiological analysis are taken from the altered locations, if required.

Sampling scheme for routine analyses of the rainbow trout fins

1. Samples for molecular analyses of the fins

Location:	Sample locations are illustrated in **Figure 60**. The sample for molecular analyses of the caudal fin is generated from the tip of the caudo-dorsal fin section. The adipose fin sample is generated from the uppermost fin portion.
Number of samples:	Two.
Sample size:	Samples are generated using scissors and forceps. Sample size is approximately 1 cm x 1 cm (caudal fin) respectively 1 cm x 0.6 cm (adipose fin) (length x width), sample height depends on the fin thickness.
Remarks:	Full thickness fin samples are generated using scissors and tweezers. For generation of the molecular adipose fin sample, the uppermost fin portion is cut off ~0.6 cm below the fin tip.
Processing:	Samples are frozen (liquid nitrogen) and stored at -20°C or -80°C (short-term storage) depending on the intended analysis (chemical or molecular analysis). For prolonged storage of tissue samples for subsequent RNA- and protein analyses, storage at -150°C is recommended.
Downstream analyses:	DNA-, RNA-, protein-, and other OMICS profiling- or targeted analyses requiring cryo-conserved specimen as well as chemical (analytical) analyses.

2. Samples for histopathological examination of the fins

Location & orientation of sections:	Sampling locations and orientations are indicated in **Figure 60**. A horizontal section (relative to the fin axis) of the adipose fin is generated, including the full fin thickness. A transverse section (relative to the fin rays) of the dorsal portion of the caudal fin is generated, including the left and right epidermal coating of the fin.
Number of samples:	Two.
Section plane size:	Approximately 1 cm x 0.2 cm (length x width) for the adipose fin and 1 cm x 0.1 cm (length x width) for the dorsal fin section.
Fixation & embedding:	FF-PE*.

**Since the dorsal fin sample contains the ossified fin ray, the sample is decalcified with a slow-acting decalcification solution for approximately 24-48 hours.*

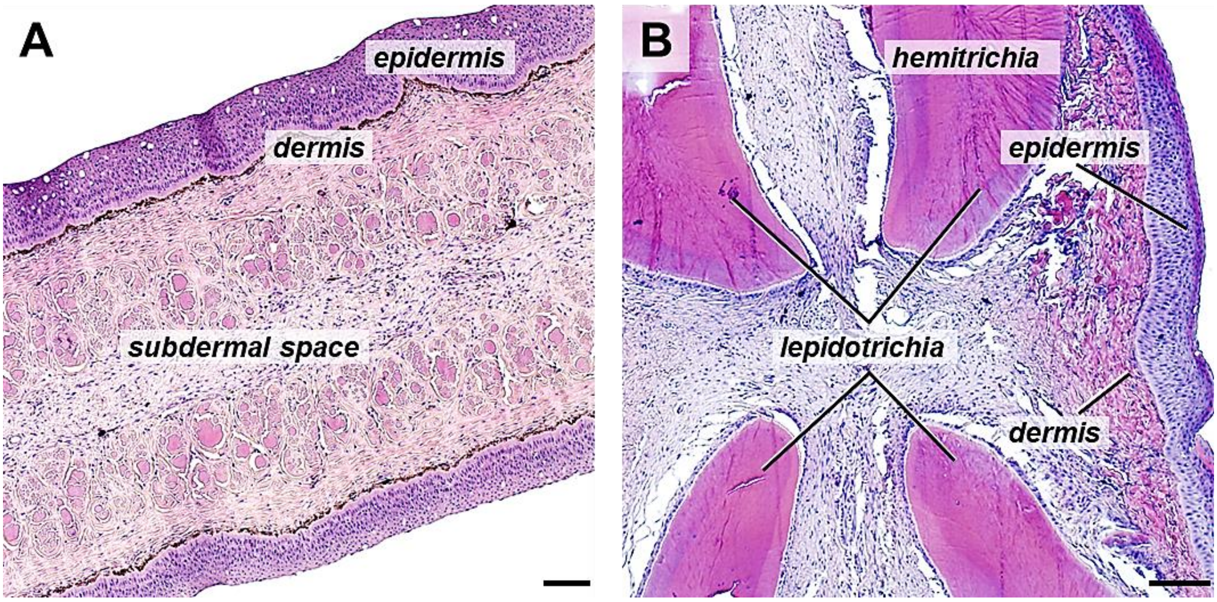

Figure 61. Histology of the adipose fin and the caudal fin. A. Horizontal section (relative to the fin axis) of the adipose fin. The adipose fin is covered by the integument composed of the multilayered stratified epidermal epithelium and the underlying dermis. The subdermal space is composed of loose connective tissue, collagen bundles, blood vessels, nerves and cellular components (*e.g.,* fibrocytes and astrocyte-like cells). The fleshy adipose fin lacks fin rays. **B.** Transverse section (relative to the axis of the fin rays) of the dorsal portion of the caudal fin. The fins are supported by special bony structures (*i.e.,* ossified fin rays (lepidotrichia), which are composed of paired hemitrichia) and are overlaid with a stratified squamous epithelium. FF-PE. HE. Bars = 100 µm.

Time requirements

Approximately 2-3 minutes are to be scheduled for dissection, macroscopic examination of all fins and the sampling and further processing of the histological and molecular samples of the rainbow trout dorsal- and adipose fin. This estimate does not include the time needed for killing the fish, dissection of gills, viscera, CNS, skin, bone tissue or musculature, prearrangement of sampling instruments and materials, or the further processing of fixed/frozen specimens.

A comparison of the proposed sampling scheme for routine analyses with previously published studies

Previously published ecotoxicological studies examining trout fins either sample several [157, 158] or specified individual fins (*e.g.,* the caudal fin) [159], without distinct information regarding sample number and size.

The sample locations of the specimens for molecular analyses as well as the locations and orientations of the samples for histopathological analyses of the rainbow trout fins are chosen so that both fin types (*i.e.,* the non-rayed adipose fin with mechanosensory function and the rayed fin as feature for body stabilization and locomotion) are represented and addressed separately. The number and sizes of tissue samples as well as the sampling locations proposed in the present guidelines are regarded to provide sufficiently representative tissue specimens for the examination of most of the (qualitative) morphological tissue alterations and molecular analyses of the rainbow trout fins.

2.11 Pseudobranchs

Relevant anatomical features/preparation

The rainbow trout pseudobranchs are vestigial gill arches, situated in the orobranchial cavity at the inner surface of the opercula (**Figure 62**). As reduced mandibular gill arches they are positioned cranial to the main gills and are covered by the opercular epithelium. Therefore, they have no direct contact with the aquatic environment [2, 26, 36, 160]. The pseudobranch is composed of one row of gill filaments (*i.e.,* a hemibranch) with numerous lamellae, which in contrast to the lamellae of the main respiratory gills are fused and supplied with oxygenated blood by post-gill arteries [26, 160, 161]. The rainbow trout pseudobranchs are mainly composed of lamellae and connective tissue, but also filament cartilage and blood vessels. The lamellae comprise pseudobranchial cells, pillar cells, blood spaces and lacunar tissue (*i.e.,* tissue filling the interlamellar space) (**Figure 63**) [26, 160]. Even if several pseudobranchial functions (*e.g.,* osmoregulation, association with vision or endocrine function) have been assumed, the physiological role is still unclear and remains subject of further experimental studies [3, 4, 26, 160, 162]. A respiratory function can be excluded, since the pseudobranchs are perfused by oxygenated arterial blood and have no contact to the aquatic environment [4, 26, 160].

If deemed beneficial for the study purpose, vascular perfusion fixation may be performed. The technique of perfusion fixation of rainbow trout tissue is described in **Chapter 2.3.2** and elsewhere [13]. At necropsy, the pseudobranch samples are generated after excision of the gills, the viscera (except the kidneys), and after sampling of the central nervous system (CNS), the integument and the locomotor system. For the generation of pseudobranch samples in routine analyses, the rainbow trout head is separated from the trunk by a transverse section cranial to the head kidney. After macroscopic examination of the pseudobranchs (and (photo-) documentation, if indicated), the fresh (*i.e.,* unfixed) pseudobranch tissue sample for molecular analyses is generated (**Figure 62A**) by cutting the opercular epithelium around the right pseudobranch and lifting off the pseudobranch from the underlying muscle, using scalpel and forceps. The opercular epithelium is removed and the specimen is frozen (liquid nitrogen) and adequately stored. For generation of the pseudobranch specimen for subsequent histopathological analysis, the remaining head portion is subsequently transferred to neutrally buffered 4% formaldehyde solution (**Figure 62B**). After immersion fixation, the entire left pseudobranch is carefully excised for histopathological analysis.

General examination parameters

After evisceration and separation of the head, the epithelium covering the pseudobranchs is blotted dry and the pseudobranchs are macroscopically examined for pathological alterations. Corresponding findings are (photo-) documented, if appropriate. Weight of the excised right pseudobranch is recorded and samples for subsequent histopathological, microbiological or parasitological analysis are taken from the altered locations, if required.

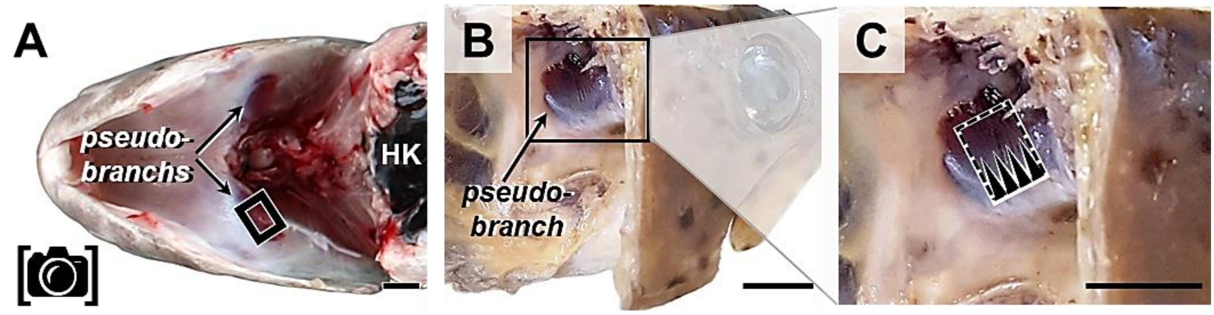

Figure 62. Photographic illustration of the sampling for histopathological and molecular analyses of the pseudobranchs of the rainbow trout. The sampling location of the molecular analysis sample (black rectangle) as well as sampling location and section plane orientation of the sample for histopathological analyses (black dotted rectangle with triangles) are indicated. **A.** Ventral aspect of the roof of the orobranchial cavity and the head kidney (**HK**) after excision of the gills (together with the tongue and the ventral wall of the orobranchial cavity) and after removal of the viscera. The pseudobranchs are located on the inside of the opercula. In fresh (*i.e.,* unfixed) condition they appear as red gill-like structures covered by the opercular epithelium. After macroscopic examination and (photo-) documentation, the right pseudobranch is removed as specimen for molecular analyses. **B.** Caudo-lateral aspect of the orobranchial cavity of the immersion-fixed rainbow trout after removal of gills, viscera, CNS and the right operculum. **C.** Detail enlargement of the left pseudobranch after immersion fixation in neutrally buffered 4% formaldehyde solution. The left pseudobranch as the specimen for histopathological analyses is carefully detached from the inside of the operculum. The orientation of the sample section plane is indicated (black dotted rectangle with triangles: the pseudobranch is sectioned parallel to the pseudobranch filaments). Bars = 1 cm.

Sampling scheme for routine analyses of the trout pseudobranchs

1. Sample for molecular analyses of the pseudobranchs

Location:	The sampling location is illustrated in **Figure 62A**. The right pseudobranch is taken as specimen for molecular analyses.
Number of samples:	One.
Sample size:	Entire right pseudobranch.
Remarks:	The right pseudobranch is carefully detached from the inside of the operculum using scalpel and forceps, the opercular epithelium is removed.
Processing:	The sample is frozen (liquid nitrogen) and stored at -20°C or -80°C (short-term storage) depending on the intended analysis (chemical or molecular analysis). For prolonged storage of the tissue sample for subsequent RNA- and protein analyses, storage at -150°C is recommended.
Downstream analyses:	DNA-, RNA-, protein-, and other OMICS profiling- or targeted analyses requiring cryo-conserved specimen as well as chemical (analytical) analyses.

2. Sample for histopathological examination of the pseudobranchs

Location & orientation of sections:	Sampling location and orientation is indicated in **Figure 62C**. The immersion-fixed left pseudobranch is separated from the inner side of the operculum and is embedded flatly with the visceral side facing downwards (*i.e.,* a sagittal section plane relative to the pseudobranch axis).
Number of samples:	One.
Section plane size:	Entire pseudobranch profile.
Fixation & embedding:	FF-PE.

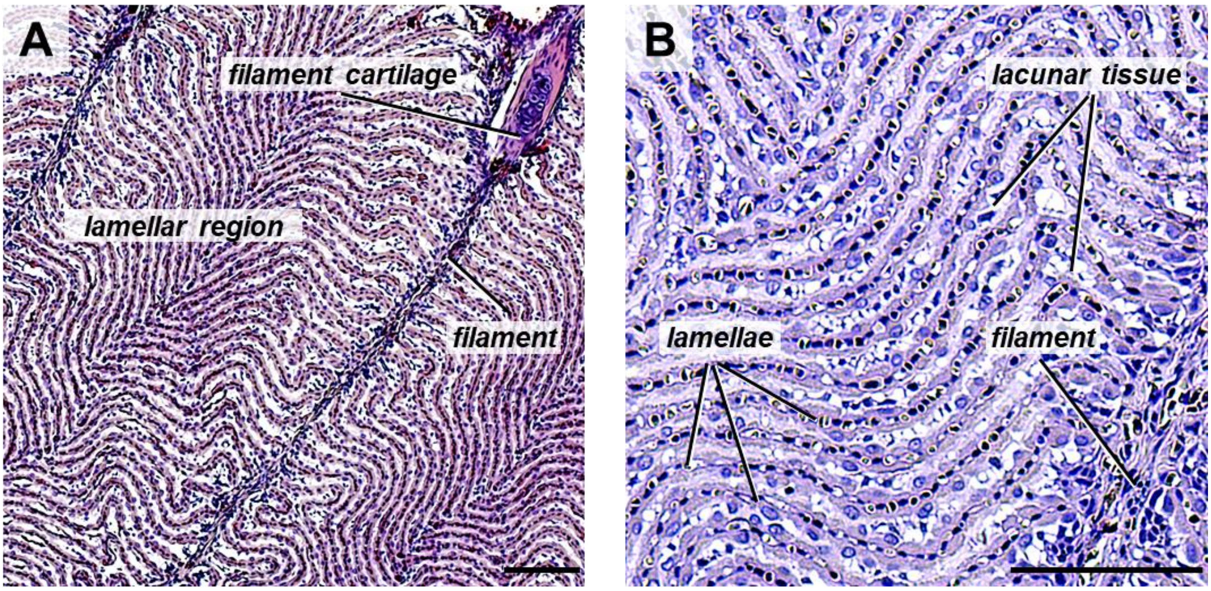

Figure 63. Histology of the rainbow trout pseudobranch. A. Sagittal section of the pseudobranch. The teleost pseudobranch is composed of gill-like structures, mainly of lamellae, but also filaments with filament cartilage, blood vessels and connective tissue. **B.** Detail enlargement of a filament and fused lamellae. The lamellar region comprises pseudobranch epithelial cells, lamellar blood spaces, pillar cells and lacunar tissue. FF-PE. HE. Bars = 100 µm.

Time requirements

2-3 minutes are to be scheduled for dissection, macroscopic examination, sampling and further processing of the rainbow trout pseudobranch samples for histopathological and molecular tissue analyses. This estimate does not include the time needed for killing the fish, dissection of gills, viscera, CNS, eyes, skin and locomotor system, prearrangement of sampling instruments and materials, or the further processing of fixed/frozen specimens. If vascular perfusion fixation is performed, at least additional 15 minutes should be scheduled.

A comparison of the proposed sampling scheme for routine analyses with previously published studies

Previously published ecotoxicological studies examining rainbow trout either sample the pseudobranchs without distinct information regarding sample number and size [163, 164] or use trout of small body sizes so that the whole fish is sectioned and the pseudobranch section profile is present in the histological section [165].

According to the authors knowledge, in ecotoxicological studies using rainbow trout the pseudobranch has so far not been subject of detailed analyses. However, since pathological pseudobranch tissue alterations are seen after exposure to several toxicants, *e.g.,* herbicides, pesticides or heavy metals [4], the present guidelines contain a sampling regime for histopathological and molecular pseudobranch analyses. The number and sizes of pseudobranch samples as well as the sampling locations and section plane orientations proposed in the present guidelines are regarded to provide sufficiently representative tissue specimens for the examination of most of the qualitative histopathological tissue alterations and molecular analyses of the rainbow trout pseudobranch. Note that the sampling protocol may need to be modified, if there is evidence of unilateral lesions (*e.g.,* abnormal necropsy findings) since histopathological and molecular examination of only one pseudobranch could falsify the assessment of unilaterally occurring tissue alterations.

2.12 Sensory system

A division is made in the acoustico lateralis system (consisting of the ear (hearing and equilibrium) and the lateral line system (perception of vibration and pressure)), the chemoreception- (*i.e.,* the olfaction and gustation) and the visual system. The following chapter contains a detailed sampling protocol of the olfactory tissue (**Chapter 2.12.1**), the sampling for the histopathological analysis of the taste buds as part of the gustatory system is illustrated in **Chapter 2.3.1**. The sampling of the inner ear (**Chapter 2.12.2**) as well as the lateral line system (**Chapter 2.12.3**) for histopathological analyses is illustrated in the present chapter. The sampling of the eye for analyses of the optic tissue is illustrated in **Chapter 2.12.4**.

2.12.1 Olfactory system

Relevant anatomical features/preparation

The olfactory system is a sensory system responsible for the reception of odorants (*i.e.,* environmental chemical stimuli) and their transmission to the central nervous system for nerval processing [2, 25, 166]. In fish, fundamental behavior patterns like avoidance, feeding, location of spawning grounds or reproduction are based on the perception and identification of different odorants (*e.g.,* pheromones or amino acids) in the aquatic environment [25, 166, 167]. In rainbow trout, the nostrils lead to the nasal pits, which are located latero-dorsally on the snout and contain the olfactory epithelium (**Figure 64**). The nasal cavities and the olfactory system are not associated with the respiratory system in teleosts [2, 3, 42]. The nostrils are each divided by a skin flap and are therefore composed of an anterior inflow- and a posterior outflow opening, allowing a permanent water circulation within the lumen of the nasal cavity during swimming (**Figure 64C**) [2, 3, 36, 42]. The floor of the nasal cavity is uplifted by several lamellae which develop from caudal to rostral and form an oval olfactory rosette (**Figures 64** and **65A**). In adult salmonids, additionally 5-10 secondary folds per lamella are present. The olfactory rosette is directly exposed to the surrounding water and is the major component of the peripheral olfactory system [2, 3, 168]. The primary sensory neurons of the olfactory system are located in the epithelium of the lamellae of the olfactory rosette (**Figure 65**). The epithelium is supported by a stromal sheet composed of connective tissue, blood vessels and axon bundles separated from the epithelium by a basement membrane. In salmonids, the olfactory epithelium is subdivided in a sensory and a non-sensory region. The former consists of a columnar epithelium composed of different cell types, such as olfactory receptor cells, non-sensory ciliated cells, goblet cells and basal cells. The sensory epithelium is consistently interrupted by a non-sensory stratified squamous epithelium containing mucous goblet cells [2, 36, 42]. There are three different types of bipolar olfactory neurons: ciliated sensory neurons, microvillous sensory neurons and crypt cells. These sensory neurons are differently distributed within the layers of the olfactory epithelium and differ in their cell morphology. It is assumed, that morphologically different sensory neurons also differ in the odorant reception and the mediated behavior patterns [166, 169, 170]. Via the olfactory nerve, the axons of the bipolar olfactory sensory cells terminate in the olfactory bulb, which is closely situated to the telencephalon [2, 166].

At necropsy, the olfactory rosette samples are generated after excision of the gills, pseudobranchs and the viscera (except the kidneys), and after sampling of the central nervous system (CNS), eyes, skin and locomotor system. For the generation of olfactory rosette samples in routine analyses, the rainbow trout`s head is separated from the trunk by a transverse section cranial to the head kidney. After macroscopic examination of the nostrils, the skin fold separating the anterior and posterior openings of each nostril is transected and the fresh (*i.e.,* unfixed) olfactory rosette situated within the left nasal pit tissue is excised after macroscopic evaluation. As specimen for molecular analyses, the left olfactory rosette is frozen (liquid nitrogen) and adequately stored. For generation of the specimen for subsequent histopathological analysis, the remaining head is subsequently transferred to neutrally buffered 4% formaldehyde solution. After immersion fixation, the entire right olfactory rosette is carefully excised for histopathological analysis.

General examination parameters

After evisceration and separation of the head, the nasal openings are blotted dry and macroscopically examined before severing the skin fold separating the anterior and posterior openings of each nostril. The olfactory rosettes are macroscopically examined for pathological alterations and corresponding findings are (photo-) documented, if appropriate. Samples for subsequent histopathological, microbiological or parasitological analysis are taken from the altered locations, if required.

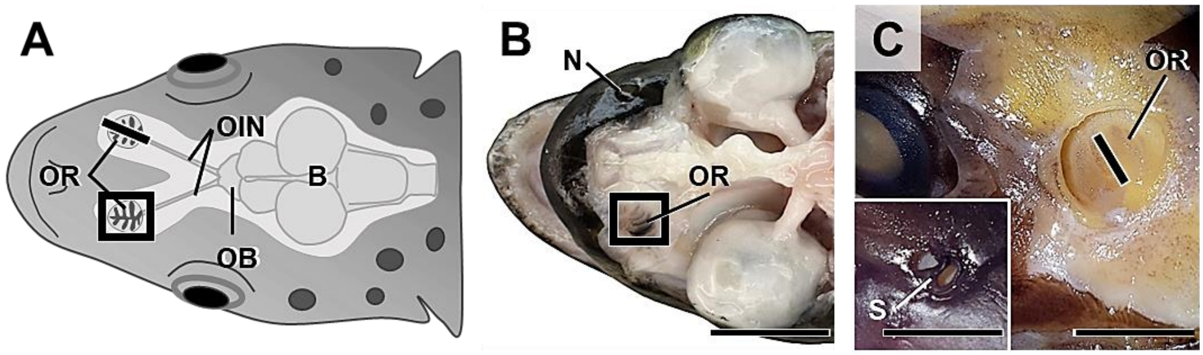

Figure 64. Illustration of the sampling for histopathological and molecular analyses of the olfactory rosettes of the rainbow trout. Sample position of the molecular analysis sample (black rectangles) as well as sample location and section plane orientation of the specimen for histopathological analyses (black lines) are indicated. (For demonstration purposes, the ocular globes and the brain remain intact and as *in situ* in the present figure. At necropsy, the sampling of the olfactory rosettes occurs after removal of the brain and eye globes.) **A.** Schematic illustration of the dorsal aspect of the exposed brain (**B**) with the olfactory bulbs (**OB**), olfactory nerves (**OlN**) and the olfactory rosettes (**OR**). The left olfactory rosette is sampled immediately after killing and necropsy for molecular analyses, whereas the right olfactory rosette as specimen for histopathological analyses is sampled after immersion fixation. **B.** Photographic illustration of the sampling for molecular analyses of the olfactory rosette. At necropsy, brain and eye balls are removed, the nasal folds of both nostrils (**N**) are transected for macroscopic examination, and the left olfactory rosette (**OR**) is dissected in fresh and unfixed state for molecular analyses. **C.** Photographic illustration of the rostro-dorsal aspect of the nasal cavity and the sampling for histopathological analyses of the immersion-fixed olfactory rosette (**OR**). The inset shows the left nostril before transection of the skin fold (**S**) separating the anterior and posterior openings. The skin folds are severed for macroscopic examination and enhanced immersion fixation of the olfactory rosette. After immersion fixation, the surrounding skin and soft tissue are removed using a scalpel and the olfactory rosette is dissected using scissors and forceps for subsequent histopathological analyses. Bars = 1 cm.

Sampling scheme for routine analyses of the trout olfactory rosettes

1. Sample for molecular analyses of the olfactory rosettes

Location:	Sampling location is illustrated in **Figure 64A**&**B**. The left olfactory rosette is excised as specimen for molecular analyses.
Number of samples:	One.
Sample size:	Entire olfactory rosette.
Remarks:	The left olfactory rosette is carefully excised from the nasal pit, using scissors and forceps.
Processing:	The sample is frozen (liquid nitrogen) and stored at -20°C or -80°C (short-term storage) depending on the intended analysis (chemical or molecular analysis). For prolonged storage of the tissue sample for subsequent RNA- and protein analyses, storage at -150°C is recommended.
Downstream analyses:	DNA-, RNA-, protein-, and other OMICS profiling- or targeted analyses requiring cryo-conserved specimen as well as chemical (analytical) analyses.

2. Sample for histopathological examination of the olfactory rosettes

Location & orientation of sections:	Sampling location and orientation is indicated in **Figure 64A**&**C**. The immersion-fixed right olfactory rosette is carefully separated from the nasal pit. A mid-sagittal section plane in relation to the olfactory rosette axis is generated.
Number of samples:	One.
Section plane size:	Approximately 0.4 cm x 0.3 cm (length x width).
Fixation & embedding:	FF-PE.

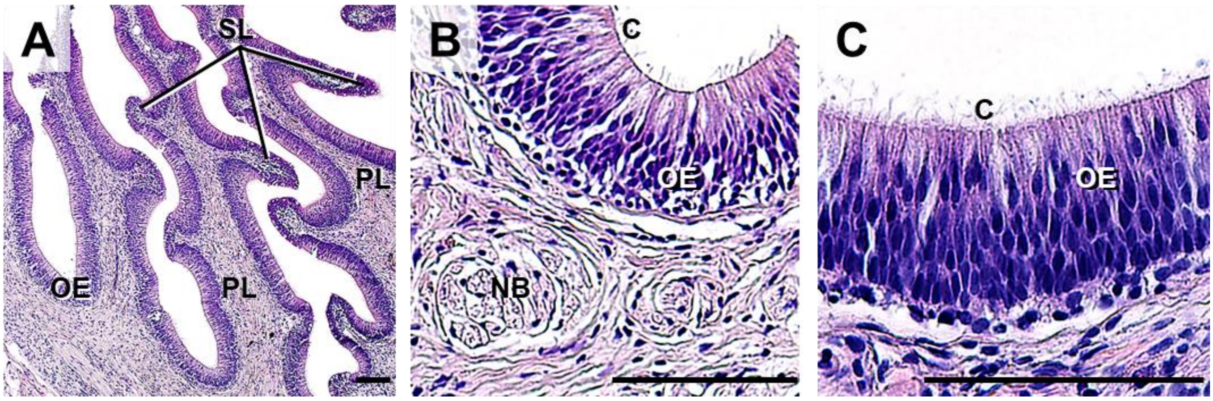

Figure 65. Histology of the rainbow trout olfactory rosette. A. Section of the lamellae of the olfactory rosette, rising from the nasal cavity floor. Several primary olfactory rosette lamellae (**PL**) are present, bearing numerous secondary olfactory rosette lamellae (**SL**). The surface of the olfactory rosette is covered by the olfactory epithelium (**OE**). **B, C.** Detail enlargement of the sensory epithelium of the olfactory rosette. The olfactory epithelium (**OE**) is a sensory, pseudostratified, columnar epithelium, composed of ciliated sensory cells (*i.e.,* receptor cells) and ciliated non-sensory cells, as well as basal- or goblet cells. The sensory receptor cells are bipolar primary neurons, the dendrites terminate as cilia (**C**) at the epithelial surface. The axons of the sensory receptor cells form nerval axon bundles (**NB**) in the submucosa, which run posteriorly merging to the olfactory nerve which ends in the olfactory bulb. FF-PE. HE. Bars = 100 µm.

Time requirements

3-5 minutes are to be scheduled for dissection, macroscopic examination, sampling and further processing of the rainbow trout olfactory rosette samples for histopathological and molecular analyses. This estimate does not include the time needed for killing the fish, dissection of gills, viscera, CNS, eyes, skin and locomotor system, prearrangement of sampling instruments and materials, or the further processing of fixed/frozen specimens.

A comparison of the proposed sampling scheme for routine analyses with previously published studies

Previously published ecotoxicological studies examining rainbow trout usually sample the entire isolated olfactory rosettes [171-174].
Since various toxicants have the potential to alter morphology and function of the olfactory sensory cells [175], the present guidelines provide a sampling regime for histopathological and molecular olfactory rosette analyses. The number and sizes of olfactory rosette samples as well as the sampling locations and section plane orientations proposed in the present guidelines are regarded to provide sufficiently representative tissue specimens for the examination of most of the (qualitative) histopathological tissue alterations and molecular analyses of the rainbow trout olfactory epithelium. Note that the sampling protocol may need to be modified, if there is evidence of unilateral lesions (*e.g.,* abnormal necropsy findings), since histopathological and molecular examination of only one olfactory rosette could falsify the assessment of unilaterally occurring tissue alterations. The focus of the present chapter for sampling of the olfactory system is placed on the olfactory rosette, sampling for molecular and histopathological analyses of the olfactory bulbs is described in detail in **Chapter 2.8**. If additional or more advanced olfactory system analyses are scheduled (*e.g.,* the analysis of the olfactory nerve), the sampling regime has to be adapted accordingly.

2.12.2 Inner ears

Relevant anatomical features/preparation

Fish have well-developed inner ears, involved in sound detection and the localization of sound sources, as well as in the perception and processing of angular- and linear acceleration or gravity force (and therefore the maintenance of the equilibrium) [2, 3, 25, 176-178]. The auditory- and vestibular end organs of the fish ear are important components in *e.g.,* communication, orientation, navigation, eye movement control or avoidance of predators [176]. The fish inner ears are bilaterally located on the posterior angles of the cranium, an osseous labyrinth within the skull skeleton encloses the membranous labyrinth [176]. Compared to terrestrial vertebrates, fish lack both the external and middle ear as well as the cochlea [176, 177]. The inner ear of the rainbow trout is a statoacoustic organ, composed of the three semicircular canals and the three otolithic organs (*i.e.,* the utricle, the saccule and the lagena) (**Figure 66C**). The semicircular canals and otolithic chambers are inter-connected with each other, lined by a single-layered epithelium and filled with viscous fluid (*i.e.,* the endolymph) [1, 42, 176]. The base of the semicircular canals is expanded to the ampullae containing the cristae ampullares, *i.e.,* ridges of connective tissue, bearing the sensory epithelium which is composed of sensory hair cells and ciliated supporting cells and is covered by a gelatinous cupula (**Figure 67A**) [2, 42, 176]. The three otolithic end organs each contain a sensory epithelium (*i.e.,* the macula, also composed of sensory hair cells and supporting cells) (**Figure 67B**&**C**), covered by an otolithic membrane and an overlying calcareous structure (*i.e.,* the otolith) [2, 42, 176, 179]. Otoliths are "ear-stones", acellular calcified structures that are of fish species-specific shape and grow throughout life time. By means of the periodic growth rings, the age of the fish can be estimated [176, 179-181]. The epithelium of the sensory end organs contains ciliated supporting cells and hair cells (*i.e.,* mechanoreceptors, bearing several stereocilia and one kinocilium) and is covered by a gelatinous cupula (semicircular canals) or an otolithic membrane and otolith (otolithic chambers) [2, 25, 176]. Both, the cupula and the otoliths are closely situated upon the sensory epithelial surface and mechanically coupled to the supporting cells and the kinocilia of the hair cells. The cilia of the sensory cells of the epithelium project into the endolymph and are therefore sheared off by cupula/otolith movements due to their mechanic coupling to the cupula/otolithic membrane [2, 25, 42, 176]. The relevant stimulus of the hair cells depends on the associated end organ, within the ampullae of the semicircular canals the stimulus perceived by the hair cells is the angular acceleration in different spatial directions and the resulting cupula movements. In contrast, the hair cell cilia of the maculae of the otolithic end organs are sheared off due to linear acceleration (*e.g.,* due to gravity or as a consequence of passive motion due to acoustic underwater waves) [2, 181].

At necropsy, the inner ears of the rainbow trout are sampled after sampling of the gills, viscera, the central nervous system (CNS), the skin, the pseudobranchs, the locomotor- and remaining sensory system. The inner ear end organs are enclosed by the osseous labyrinth, which is bilaterally situated within the posterior skull, caudal to the orbita. The head is removed from the trunk by a cross section cranial to the head kidney, subsequently the lower jaw is removed using knife or scissors, the anterior portion of the remaining skull is removed by a transversal section immediately caudal to the orbits. The remaining specimen containing skin, bone, osseous labyrinth and the inner ear structures is immersion-fixed in neutrally buffered 4% formaldehyde solution and decalcified.

2.12.2 Inner ears

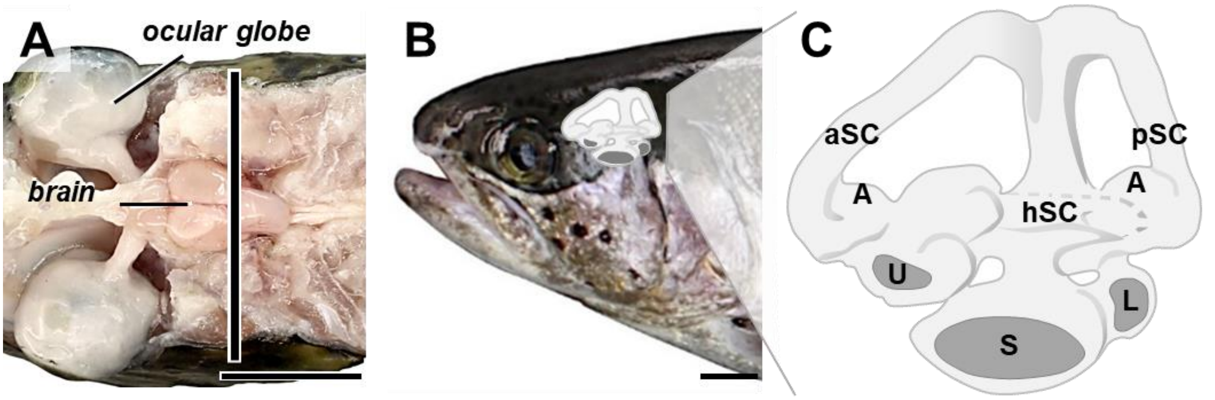

Figure 66. Schematic illustration of the sampling for histopathological analysis of the inner ear of the rainbow trout. A. Dorsal aspect of the ocular globes and brain after removal of the cranial vault and dorsal portion of the orbital bones. The sample location and section plane orientation of the sample for histopathological analyses of the inner ear (black line) is indicated, both inner ears are included in the histological section. Note that the specimen of the inner ear is sampled after sampling of *e.g.,* the skin, CNS, eye and olfactory system. For demonstration purposes the ocular globes and the brain remained intact and as *in situ* in the present figure. **B.** Lateral aspect of the left side of the head of the rainbow trout. An external and middle ear is missing, the position of the inner ear is schematically indicated. **C.** Schematic illustration of the lateral aspect of the left inner ear, isolated from the osseous labyrinth. The inner ear of the rainbow trout is composed of three semicircular canals and three otolithic organs, a cochlea is missing. Important morphological structures are indicated: **aSC:** Anterior semicircular canal; **pSC:** Posterior semicircular canal; **hSC:** Horizontal semicircular canal; **A:** Ampulla; **U:** Utricle; **S:** Saccule; **L:** Lagena. The grey structures within the otolithic chambers represent the otoliths. The otolith of the saccule is named sagitta, the otolith of the utricle is called lapillus and the otolith of the lagena is named asteriscus. Bars = 1 cm.

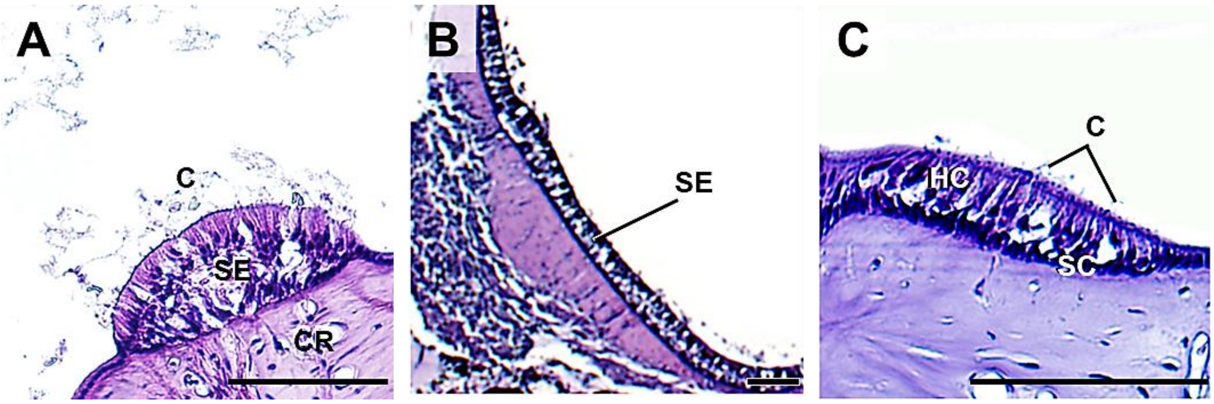

Figure 67. Histology of the sensory end organs of the inner ear of the rainbow trout. A. Histology of the sensory epithelium of the crista ampullaris, the sensory end organ of the semicircular canals. The ampullae semicirculares contain ridges of connective tissue (**CR**), bearing the sensory epithelium (**SE**) which is composed of sensory hair cells and ciliated supporting cells and is covered by a gelatinous cupula (**C**), which usually shrinks due to the processing for histological embedding. **B, C.** Histology of the sensory epithelium (**SE**) of the sagitta (*i.e.,* the macula). The sensory epithelium is composed of supporting cells (**SC**) and mechanosensory hair cells (**HC**). The cilia (**C**) of the hair cells and ciliated supporting are mechanically linked to the otolithic membrane and otolith (not present here due to the immersion fixation and decalcification of the specimen). FF-PE. HE. Bars = 100 µm.

General examination parameters

In advance, attention should be paid to behavior patterns indicating morphological alterations of the auditory system and/or an impairment of the (mechano-) sensory system by environmental pollutants (*e.g.,* abnormal swimming behavior), corresponding findings should be recorded. The posterior head region as well as the inner aspect of the cranium (after removal of the CNS) is macroscopically examined, corresponding findings are (photo-) documented, if appropriate. Samples for subsequent molecular, histopathological or microbiological analysis are taken from the altered locations, if required.

Sampling scheme for routine histopathological analyses of the inner ear of the rainbow trout

Location & orientation of sections:	Sampling location and orientation is indicated in **Figure 66A**. A transverse section of the head, ~0.3 cm posterior to the orbits is generated, containing both inner ears, the overlying skin and the enclosing bone tissue. A tissue block of ~2.5 cm x 2 cm x 1 cm (length x width x height) is generated.
Number of samples:	One.
Section plane size:	Approximately 2.5 cm x 2 cm (length x width).
Fixation & embedding:	FF-PE*.

**The sample is fixed in 10% formalin and then decalcified with a slow-acting decalcification solution for approx. 5-7 days.*

Time requirements

2-3 minutes are to be scheduled for dissection, macroscopic examination, sampling and further processing of the rainbow trout inner ear sample for histopathological analyses. This estimate does not include the time needed for killing the fish, dissection of other organs/tissues, prearrangement of sampling instruments and materials, or the further processing of fixed/frozen specimens.

A comparison of the proposed sampling scheme for routine analyses with previously published studies

According to the authors knowledge, the examination of the trout inner ear is not standard practice in ecotoxicological studies. If ecotoxicological studies using trout are conducted, usually the sagittal otoliths are removed (*e.g.,* for subsequent laser ablation inductively coupled plasma mass spectrometric analyses) [182-184]. Since otoliths grow continuously, lack of resorption and are metabolically inert, the otolith microchemistry may serve as a useful tool in evaluation of environmental exposure history or environmental risk assessment [182, 183, 185]. Toxicity studies examining the inner ear end organs of other teleost fish, indeed analyze the response of the sensory epithelium to the ototoxicity (*e.g.,* loss or damage of hair cells) of several chemicals and drugs [186]. Therefore, these guidelines recommend the generation of inner ear end organ samples for standard histopathological analyses. Using light microscopy, the sensory epithelium of each inner ear end organ is of the same composition (*i.e.,* it contains the two basic cell types (hair cells and supporting cells), an electron microscope analysis is needed for identification of the different hair cell types). The sampling location and section plane orientation proposed in the present guidelines is regarded to provide a sufficient representative tissue specimen for the examination of the (qualitative) histopathological tissue alterations of the rainbow trout inner ear sensory end organs. Note that the sampling protocol needs to be adapted accordingly, if more advanced investigations of the structures of the inner ear (*e.g.,* molecular analyses) are intended, since the generation of specimens for molecular analyses is not included in the present guidelines.

2.12.3 Lateral line system

Relevant anatomical features/preparation

The lateral line organ is involved in a majority of the known behaviors of teleosts [25]. Due to the mechanosensory lateral line system, fish are capable of *rheotaxis*, *i.e.,* behavioral orientation in accordance with water displacements, mediated by hydrodynamic sensory organs [187-189]. Furthermore, it contributes to *e.g.,* schooling, prey detection or communicational behavior [25, 189-193]. The lateral line canals in trout are comprised of the head portion with several bilateral (temporal, supraorbital, suborbital and preopercular-mandibular) canals and the trunk lateral line canal, bilaterally running the trunk at level of the horizontal septum (**Figure 68**) [4, 188, 194]. Short tubular epithelial-lined segments located in the lateral line scales (*i.e.,* special scales supporting the lateral line canal on the teleost trunk) form the fluid-filled lateral line canal. These overlapping and linearly arranged scales appear as flattened plates with mounted tubes, which are connected via pores (infrascalar and suprascalar), additional pores on the dorsal scale surfaces connect the canal lumen and the external aquatic environment [2, 4, 187, 194]. The neuromasts are the sensory organs of the lateral line system, in teleosts they are classified into superficial neuromasts and neuromasts of the subdermal lateral line canals [25, 188, 189]. In trout, the canal neuromasts are more abundant than the superficial neuromasts [195]. Neuromasts with their sensory hair cells are situated at the basis of the canal chambers (**Figure 69**) and one neuromast is usually located between one pair of pores. The cilia of the hair cells are embedded in the overlying gelatinous cupula [1, 42, 189, 194, 196]. The hair cell receptors of the lateral line system respond to aquatic environment disturbances (water movements created *e.g.,* by moving objects or animals) relative to the fish body surface. Water movements lead to a shift of the cupula and the hair cell cilia are sheared off, the shearing movement is triggering a neural response of the underlying nerve fibers [42, 189, 190, 194, 196]. The sensory hair cells are located in the upper part of the neuromasts, the number of hair cells as well as the neuromast size increase in correlation with increasing fish size [197]. The sensory cells are surrounded by supporting cells and mantle cells. These non-sensory cells secret the gelatinous cupula substance or build the boundary of the neuromasts to the surrounding lateral line epithelium [1, 2, 189, 196, 197].

At necropsy, the trunk lateral line canal is examined histopathologically on the same specimen sampled for histopathological analyses of the red skeletal muscle. The specimen is therefore sampled according to the sampling protocol given in **Chapter 2.10.1**. Briefly, the sample is generated from the left body side after macroscopic examination, photographic documentation and excision of gills, viscera (except the kidneys) and central nervous system (refer to **Figure 68)**. If analysis of further organs or tissues is scheduled, it may be advantageous to trim the carcass for subsequent immersion fixation in neutrally buffered 4% formaldehyde solution before sampling. The remaining trunk (including the kidneys), head, tail and the removed body walls are preserved (*i.e.,* immediately transferred to an adequate fixative), to ensure that sample material is available for new/expanded scientific issues arising from the analyses.

2.12.3 Lateral line system

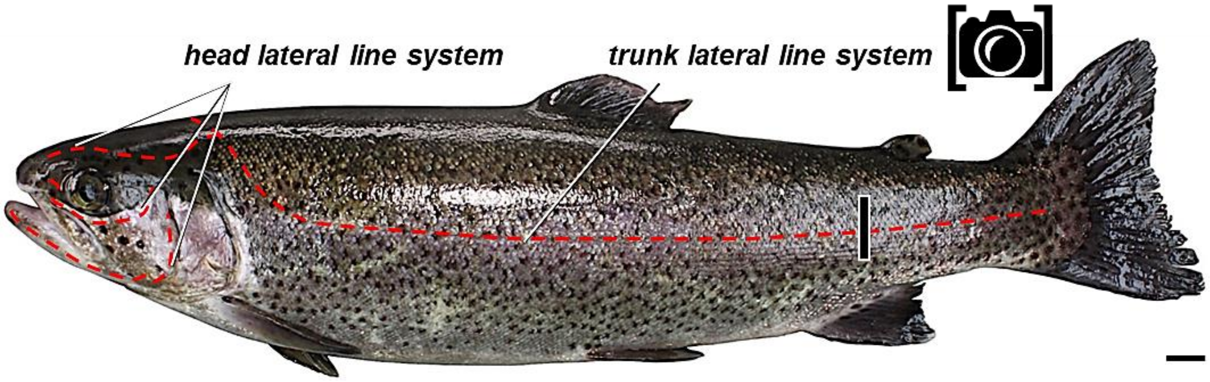

Figure 68. Schematic illustration of the sampling for histopathological analysis of the lateral line system of the rainbow trout. Lateral aspect of the left body side of an intact rainbow trout. The position of the head and trunk lateral line canals is indicated by the red dashed lines. (Note that the lateral line specimen is collected after removal of the left abdominal wall and sampling of gills, viscera (except the kidneys), central nervous system and locomotor system. The fish in this figure is remained intact for demonstration purposes.) The sample location and section plane orientation of the sample for histopathological analyses is indicated (black line). Bar = 1 cm.

General examination parameters

In advance, attention should be paid to behavior patterns indicating morphological alterations of the lateral line system and/or an impairment of the (mechano-) sensory system by environmental pollutants, corresponding findings should be recorded. The integument covering the head- and trunk lateral line canals is macroscopically examined for pathological alterations by inspection of the skin and lateral line scales, corresponding findings are (photo-) documented, if appropriate. While sampling, the lateral line canal profile on the section surfaces is also macroscopically examined. Samples for subsequent histopathological, molecular or microbiological analysis are taken from the altered locations, if required.

Sampling scheme for routine histopathological analyses of the rainbow trout lateral line system

Location & orientation of sections:	Sampling location and orientation is indicated in **Figure 68**. A transverse section of the longitudinal superficial muscle and the overlying skin is generated, including the lateral line scales and the lateral line canal. A tissue blocks of ~1.5 cm x 0.4 cm x 0.7 cm (length x width x height) is generated.
Number of samples:	One.
Section plane size:	Approximately 1.5 cm x 0.7 cm (length x width).
Fixation & embedding:	FF-PE*.

**Due to the scale-containing skin, the sample is fixed in 10% formalin and then decalcified with a slow-acting decalcification solution for approx. 24-48 hours.*

2.12.3 Lateral line system

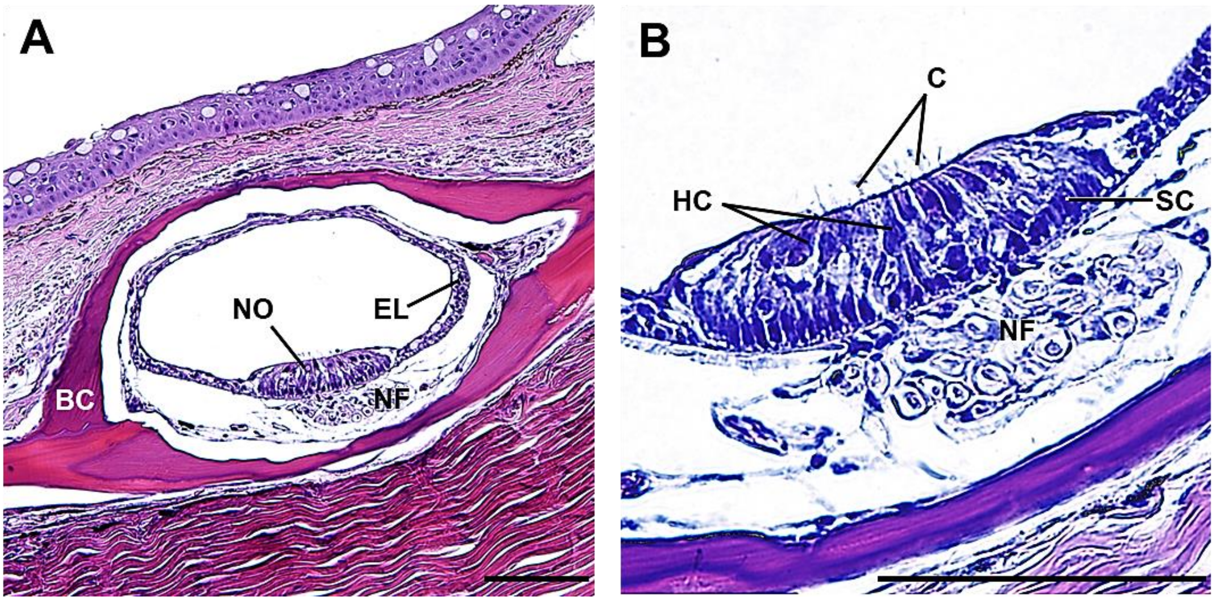

Figure 69. Histology of the rainbow trout trunk lateral line canal. A. Cross section of the lateral line canal. The lateral line is enveloped by a bony canal (**BC**) and lined by a thin epithelium (**EL**). The mechanosensory organs (*i.e.,* the neuromast organs (**NO**)) of the lateral line are situated on the floor of the canal, innervated by some nerve fibers (**NF**) of the lateral line nerve. **B.** Detail enlargement of a neuromast organ. The neuromasts are composed of the hair cells (**HC**) with their apical cilia (**C**) as basic feature of the neuromast function. The cilia project into the gelatinous cupula and are sheared off by cupula movement. This way water movements are transmitted to the sensory cells and a neural response of the underlying lateral line nerve fibers (**NF**) is triggered. The gelatinous cupula covering the neuromast hair cells usually shrinks due to the processing for histological embedding [42]. Next to the hair cells, the neuromast organs are composed of supporting cells (**SC**) which are secretory active. FF-PE. HE. Bars = 100 µm.

Time requirements

2-3 minutes are to be scheduled for dissection, macroscopic examination, sampling and further processing of the rainbow trout lateral line canal sample for histopathological analyses. This estimate does not include the time needed for killing the fish, dissection of gills, viscera and central nervous system, prearrangement of sampling instruments and materials, or the further processing of the fixed specimen.

A comparison of the proposed sampling scheme for routine analyses with previously published studies

According to the authors knowledge, the examination of the lateral line system is not standard practice in ecotoxicological studies of rainbow trout. On the other hand, toxicity studies examining other teleost fish (*e.g.,* the zebrafish (*Danio rerio*) or the sea bass (*Dicentrarchus labrax*)) indeed analyze the response of the lateral line system on environmental contaminants (*e.g.,* heavy metals) [198-200]. The lateral line system is directly exposed to the aquatic environment and therefore vulnerable to the effects of waterborne pollutants [201]. Consequently, these guidelines recommend the sampling of the lateral line system for standard histopathological analyses, the generation of specimens for molecular analyses is not included. Since the morphological differences between the neuromasts of the head- and trunk canal are minimal [202], the sampling location proposed in the present guidelines is regarded to provide a sufficient representative tissue specimen for the examination of the (qualitative) histopathological tissue alterations of the rainbow trout`s lateral line system. If more advanced investigations of the lateral line system are intended, the sampling protocol needs be adapted accordingly.

2.12.4 Eyes

Relevant anatomical features/preparation

The trout eye anatomy and histology basically resemble that of mammals, but in adaptation to the aquatic environment it is endowed with some special features [203]. The eyes are situated in the orbit region of the neurocranium, the eyelids are rudimentarily developed (in trout, there is a fatty membrane surrounding the eye globe) [1, 4, 204]. The ocular globes display a flat and ellipsoidal shape, the visual (*i.e.,* antero-posterior) axis is shorter than the transverse axis [204, 205] (refer to **Figure 70A**). Refraction capability is not provided by the flattened thick cornea, but by the spherical lens, which is also responsible for accommodation (*i.e.,* the lens is moved within the ocular globe by the retractor lentis muscle) [2-4, 203, 204]. The cornea, the limbus and the sclera with its supporting cartilaginous plate form the fibrous tunic of the eyeball [4]. The vascular tunic is composed of the immobile iris, a rudimental ciliar body and the choroid with the choroidal gland and the falciform process. The choroid gland is a choroidal counter-current network of blood capillaries adjacent to the optic nerve, which is in communication with the pseudobranch and is assumed to elevate arterial blood oxygen. The falciform process is a choroid fold protruding through the retina, which is origin of the retractor lentis muscle [3, 4, 204, 205]. The nervous tunic of the rainbow trout eyeball consists of the retina, a multilayered and highly differentiated neuroepithelial structure which contains the photoreceptor cells (*i.e.,* rods and cones) [2, 3, 204, 205]. As a protective mechanism and for adaptation to varying light intensity, the photoreceptors can be retracted into the retinal pigment epithelial layer (*i.e.,* the retinomotor response) [4, 203, 205]. Axons of the retinal ganglion cells form the optic nerves, which intermingle at the optic chiasm [2].

At necropsy, the eyes are removed after excision of the gills and the viscera (except the kidneys) and after sampling of the central nervous system, the integument and the locomotor system. The head remains connected to the body so as to avoid damaging the head kidney. Enucleation is performed according to the method described by Stoskopf [1]. Beginning at the corner of the eye, the palpebral conjunctiva is grasped with tweezers and circularly dissected between the bony orbit and the eyeball using fine curved scissors. Connective and adipose tissue is loosened by blunt separation; eye muscles and optic nerves are dissected with fine curved preparation scissors. Muscles and adipose tissue adhering to the enucleated eye globe are carefully removed without damaging the fibrous tunic. If an examination of the brain/central nervous system (CNS) is also required, eyes and brain can be removed connectedly, as described in **Chapter 2.8**, the eyes are subsequently separated by severing the optic nerves. After excision, macroscopic examination, weighing and photographic documentation (if appropriate), the samples for histopathological and molecular analyses of the eyes are generated. The right eye is transferred to neutrally buffered 4% formaldehyde solution for subsequent histopathological analysis. Since the transfer of the eye into fixation solution leads to a shrinkage of the eyeball, fixative is injected into the anterior eye chamber using an insulin syringe, as illustrated in Reimann [206] and in **Figure 72A**, to prevent a sinking of the cornea and to maintain the physiological cornea curvature. The left (unfixed) eye is sampled for molecular analyses. Remaining tissue is preserved (*i.e.,* immediately transferred to an adequate fixative), to ensure that sample material is available for new/expanded scientific issues arising from the analyses.

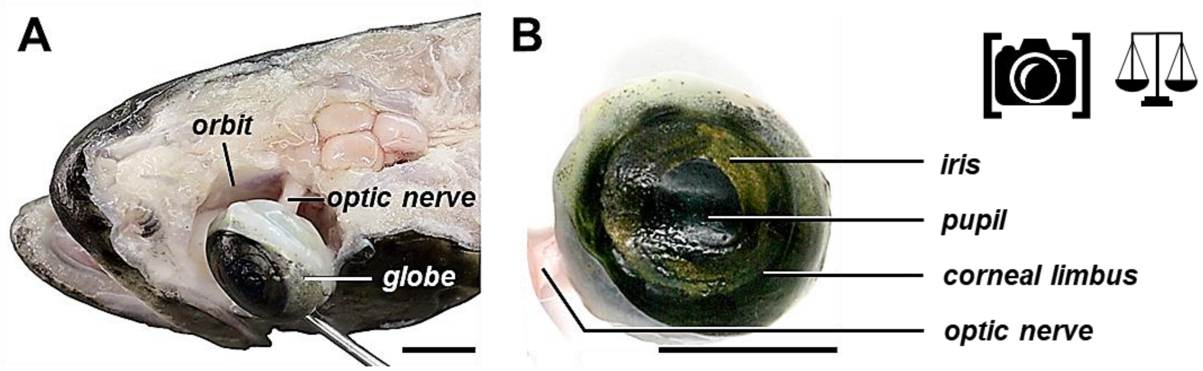

Figure 70. Photographic illustration of the rainbow trout eyes. A. Cranio-dorsal aspect of the left eye globe of a rainbow trout after removal of the cranial vault and supraorbital bones. For excision of the eyes together with the brain/central nervous system (CNS), the optic nerves remain intact and eyes, optic nerves and brain are dissected as illustrated in **Chapter 2.8**. If excision of brain/CNS is not scheduled, eyes are dissected according to the method illustrated in Stoskopf [1] (*i.e.,* the palpebral conjunctiva is circularly dissected, connective and fatty tissue is loosened by blunt separation and eye muscles and optic nerves are dissected with fine curved preparation scissors). **B.** Cranio-dorsal aspect of the ocular globe, carefully dissected from the orbit. Macroscopically visible structures of the eyes are indicated. Bars = 1 cm.

General examination parameters

The dissected eyes are briefly dabbed dry with a laboratory paper towel and weighed to the nearest mg. Both eyes are macroscopically examined for pathological alterations, corresponding findings are (photo-) documented. Samples for subsequent histopathological, molecular or microbiological analysis are taken from the altered locations, if required.

Sampling scheme for routine analyses of the rainbow trout eyes

1. Samples for molecular analyses of the eye

Location:	Sampling procedure is illustrated in **Figure 71**.
Number of samples:	All samples (three tissue samples, one sample of vitreous humour, if indicated) for molecular analyses of the different eye structures are collected from the left globe.
Remarks:	A sample of vitreous humour (approx. 0.1 ml) is taken using an appropriately sized syringe and cannula and transferred to a centrifuge tube for subsequent freezing (**Figure 71A**). The cornea is severed along the limbus and adequately stored, the globe is then separated into its anterior and posterior portion and the lens is removed (**Figure 71B**&**C**). The retina is carefully removed from the pigment epithelium using tweezers and scissors (**Figure 71D**). According to Negishi et al. [207], dark-adaption of the fish for ~1 hour facilitates retina isolation.
Processing:	Samples are frozen (liquid nitrogen) and stored at -20°C or -80°C (short-term storage) depending on the intended analysis (chemical or molecular analysis). For prolonged storage of tissue samples for subsequent RNA- and protein analyses, storage at -150°C is recommended.
Downstream analyses:	DNA-, RNA-, protein-, and other OMICS profiling- or targeted analyses requiring cryo-conserved specimen as well as chemical (analytical) analyses.

2.12.4 Eyes

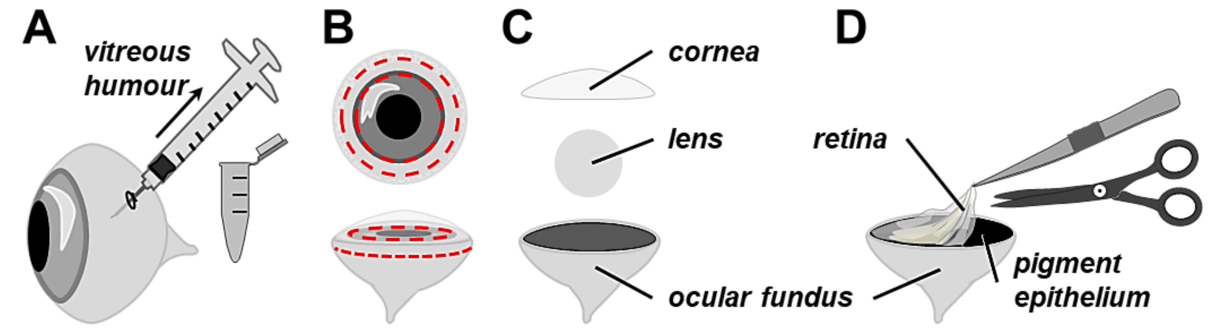

Figure 71. Schematic illustration of the sampling for molecular analyses of the eyes. A. Freshly excised (left) eye globe. If required, a sample of vitreous humour is taken, using an appropriately sized syringe and cannula and transferred to a centrifuge tube. **B.** Upper image: Frontal aspect of the enucleated eye globe; lower image: Lateral aspect of the enucleated eye globe. The cornea is dissected by a circular section along the limbus (inner red dashed line), subsequently the anterior portion of the eye globe is carefully separated from the posterior portion by a second circular section (outer red dashed line). Both cuts are made using scalpel and forceps. **C.** The cornea and the lens are sampled in their entirety and appropriately processed/frozen for subsequent molecular analyses. **D.** The retina is carefully grasped using fine forceps and completely separated from the pigment epithelium using scissors and subsequently appropriately processed/frozen.

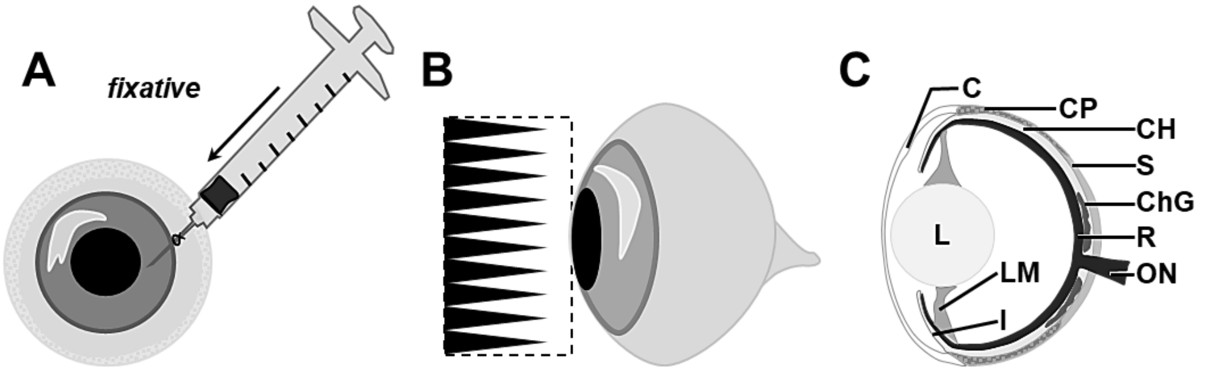

Figure 72. Schematic illustration of the sampling for histopathological examination of the eyes. A. Freshly excised (right) eye globe. To preserve the physiological shape of the ocular structures, appropriate fixative is injected in the anterior eye chamber (refer to Reimann [206]). The eye globe is subsequently transferred to neutrally buffered 4% formaldehyde solution. **B.** The sampling location and orientation of the specimen for histopathological examination is indicated (black dotted rectangle with black triangles, indicating the cutting level parallel to the picture plane). The fixed eye globe is midsagittally (*i.e.,* antero-posteriorly) halved, the optic nerve is mid-axially sectioned. For optimal paraffin embedding, a second cut parallel to the first one is made in the periphery. **C.** Schematic illustration of the histological section profile of the eye globe (compare to **Figure 73**). Important morphological structures are indicated: **C:** Cornea; **L:** Lens; **CP:** Cartilage plate; **CH**: Choroid; **S:** Sclera; **ChG:** Choroidal gland; **R:** Retina; **ON:** Optic nerve; **LM:** Lens muscle; **I:** Iris.

2. Sample for histopathological examination of the eye

Location & orientation of sections:	Sampling location and orientation is indicated in **Figure 72**. The right globe is opened midsagittally using a microtome blade. The section plane contains all tunics of the eyeball, the lens and the intraorbital portion of the optic nerve. The right half of the eye globe is embedded in paraffin.
Number of samples:	One.
Section plane size:	The sample size depends on eye diameter, the entire right half of the right eyeball (including the lens) is embedded.
Fixation & embedding:	FF-PE*.

**Since the transfer of the eye into a container with fixation solution leads to a shrinkage of the eyeball, fixative is isovolemically and fractionally injected into the anterior eye chamber using an insulin syringe. After paracentesis, a small amount of aqueous humour (approximately 0.1 ml) is removed from the anterior eye chamber and is replaced by fixative solution until the intraocular pressure is regained. For an impeccable result, the injection of the fixative has to be repeated (e.g., after 20 min, 1 h, 12 h and 24 h) to maintain the physiological cornea curvature.*

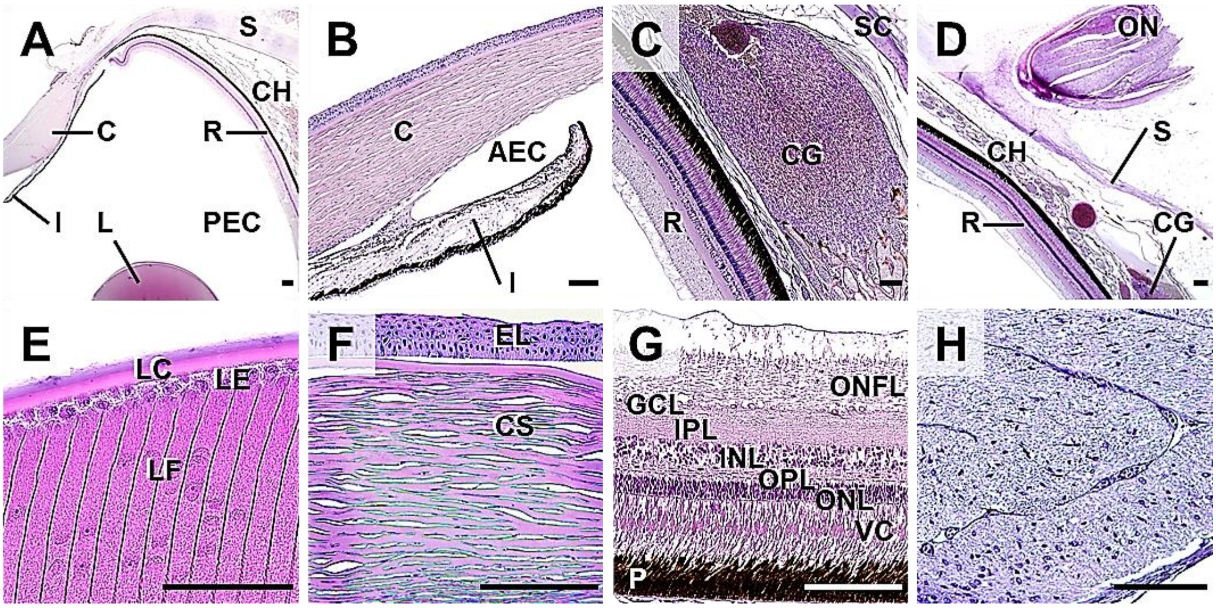

Figure 73. Histology of the rainbow trout eye. Midsagittal section of the (right) rainbow trout eye. **A.** Section of the globe posterior eye chamber (**PEC**) with the spherical lens (**L**). Section profiles of the fibrous tunic (**C:** Cornea; **S:** Sclera), the vascular tunic (**I:** Iris; **CH:** Choroid) and the nervous tunic (**R:** Retina) are shown. **B.** Section of the angle of the anterior eye chamber (**AEC**), bounded by the cornea (**C**) and the immobile iris (**I**). The cornea is thickened in the periphery and covered by a thick epithelium. An annular ligament in the angle of the anterior eye chamber interconnects iris and cornea. **C.** Section of the ocular fundus. The sclera is supported by a cartilaginous plate (**SC**). A feature of the rainbow trout eye is the choroid gland (**CG**) in the choroid, a rete mirabile providing oxygen and functioning as "pressure pad". The retina (**R**) (*i.e.,* a layered neuroepithelial structure) is adjacent to the vitreous body. **D.** Section of the ocular fundus composed of retina (**R**), choroid (**CH**) and sclera (**S**). The optic nerve (**ON**) runs from the optic disc at the ocular fundus to the optic chiasm. The choroid gland (**CG**) is located around the optic nerve. **E.** The spherical lens of the teleost eye has a high refractive index and is moved by the retractor lentis muscle. The lens is composed of a lens capsule (**LC**), a lens epithelium (**LE**) and a stroma with lens fibers (**LF**). **F.** The rainbow trout cornea is composed of a thick multi-layered squamous epithelium (**EL**) and a homogenous corneal stroma (**CS**) composed of collagen fiber bundles (*i.e.,* corneal lamellae). The thickness of the cornea varies between the periphery and the center. **G.** The retina is a neuroepithelial structure, converting visual signals in neuronal action potentials. It is composed of different layers: **ONFL:** Optic nerve fiber layer; **GCL:** Ganglion cell layer; **IPL:** Inner plexiform layer; **INL:** Inner nuclear layer; **OPL:** Outer plexiform layer; **ONL:** Outer nuclear layer; **VC:** Visual cells (rods and cones); **P:** Pigment epithelium. **H.** Section of the optic nerve. The nerve fibers appear as broad folded structures in the tubular sheet. FF-PE. HE. Bars = 100 µm.

Time requirements

Approximately 5-7 minutes are to be scheduled for dissection, macroscopic examination, sampling and further processing of the histopathological and molecular samples of the rainbow trout eyes. This estimate does not include the time needed for killing the fish, dissection of gills/viscera/CNS/integument/locomotor system, prearrangement of sampling instruments and materials, or the further processing of fixed/frozen specimens.

A comparison of the proposed sampling scheme for routine analyses with previously published studies

Previously published (eco)toxicological studies examining rainbow trout eye samples usually excise and sample the whole eye globes [94, 208, 209] or remove distinct eye structures (*e.g.,* lens or vitreous humour) from the eye for separate processing and examination [210, 211].
The location and orientation of the sample for histopathological analyses of the rainbow trout eye is chosen so that all relevant eye structures are represented in the histological section. The specimens for molecular analyses are selected to address the complex eye structure comprised of several differently composed tissues. The number and sizes of tissue samples as well as the sampling locations proposed in the present guidelines are regarded to provide sufficiently representative tissue specimens for the examination of most of the (qualitative) tissue alterations and molecular analyses of the rainbow trout eyes. Note that the sampling protocol may need to be modified, if there is evidence of unilateral lesions (*e.g.,* abnormal necropsy findings or clinical signs), since histopathological and molecular examination of only one eye could falsify the assessment of unilaterally occurring tissue alterations.

2.13 Endocrine system

Several environmental pollutants, such as oral contraceptives, heavy metals or pesticides, have the potential to negatively affect the fish endocrine system (*e.g.,* by acting as hormone agonists/antagonists or enzyme inhibitors/inducers) and, as a consequence, to adversely affect fish health, reproduction and behavior [212, 213]. For many countries, the identification of the potential impact of so-called endocrine-disrupting chemicals (EDCs) in ecological risk assessment is a regulatory mandate [214]. Next to other fish species, the rainbow trout is a commonly used model system suitable for ecotoxicological studies and therefore also for the identification of the morphological and functional effects of the EDC exposure [9, 212, 213, 215-219]. Therefore, this chapter includes information on the anatomy, histology, species-specific features and the sampling scheme for the following compartments of the endocrine system of the rainbow trout: pituitary gland (hypophysis), endocrine pancreas, thyroid gland, interrenal- and suprarenal tissue, corpuscles of Stannius, epiphysis (pineal gland), urophysis (caudal neurohypophysis) and ultimobranchial gland (**Figure 74**). Due to the particular importance of the gonads in ecotoxicological studies, a separate chapter (**Chapter 2.6**) is dedicated to the sampling of the testes and ovaries. For convenience only and in the sake of user comfort, the sampling of the endocrine pancreas is included in **Chapter 2.3.4** (pancreas), and the sampling of the inter- and suprarenal tissue is illustrated in **Chapter 2.7** (kidneys). Therefore, for these organs/tissues the present chapter rather serves as a supplement, including the most important anatomical and histological features. Details on tissue-/organ-specific sampling methods are only specified if deemed necessary (*e.g.,* for more extensive analysis). For the other endocrine organs/tissues, sampling for histopathological analyses is illustrated, whereas the generation of specimens for molecular analyses is not featured. According to the authors knowledge, previously published ecotoxicological studies examining endocrine organs/tissues of rainbow trout either sample the whole organs/tissues or lack distinct information regarding sample number and size. Therefore, if the study design requires molecular analyses of a certain endocrine tissue or organ, the authors recommend sampling the corresponding organ/tissue *in toto,* unless otherwise stated. Samples for molecular analyses are frozen (liquid nitrogen or dry ice) and stored at -20°C or -80°C (short-term storage) depending on the intended analysis (analytic chemistry or molecular analysis). For prolonged storage of tissue samples for subsequent RNA- and protein analyses, storage at -150°C is recommended.

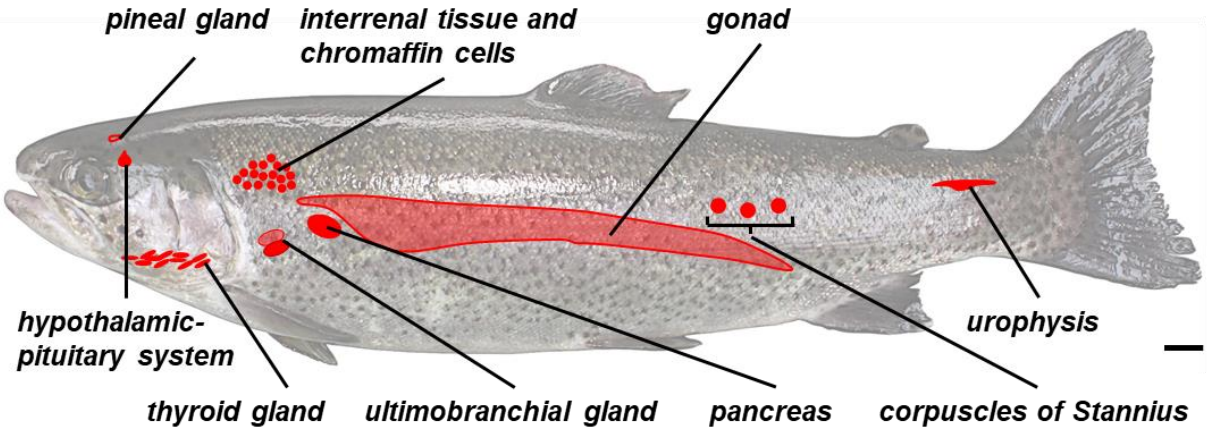

Figure 74. Schematic illustration of the endocrine system of the rainbow trout. The location of the endocrinologically active organs and tissues of the rainbow trout are schematically indicated. The endocrine system of rainbow trout resembles that of other teleosts. Compared to terrestrial vertebrates, the fish endocrine system exhibits some (functional and morphological) modifications in adaption to the aquatic environment (*e.g.,* the formation of the urophysis and corpuscles of Stannius, which are both involved in the regulation of the calcium homeostasis). In contrast to mammals, the thyroid gland and the adrenal gland are not organized in discrete and compact glands. Bar = 1 cm.

2.13.1 Pituitary gland (Hypophysis)

Relevant anatomical features

The pituitary gland or hypophysis of the rainbow trout is located ventral to the diencephalon and comprised of two histologically and functionally differing parts: the neurohypophysis (pars nervosa), which is of neural origin, derives from the ventral aspect of the diencephalon and is situated in the hypophyseal center; and the adenohypophysis, which is of ectodermal origin and derives from the pharyngeal epithelium [2, 3, 25, 36, 65].
The neurohypophysis comprises a core composed of glial cells (*i.e.,* pituicytes) and ependymal cells and a stalk with neurosecretory axonal fibers of hypothalamic neurons (*i.e.,* the neurohypophyseal tract), which extends into the neurohypophyseal center [65]. Known hormones of the teleost neurohypophysis are vasotocin (*i.e.,* a vasopressin-like peptide) and isotocin (*i.e.,* an oxytocin-like peptide) [2, 4, 25]. The adenohypophysis is organized in the rostral and proximal pars distalis and the pars intermedia, all arranged around the neurohypophyseal core. The pars intermedia encloses the major part of the neurohypophyseal core and accounts for up to 60% of the pituitary volume in salmonids [2, 42, 65]. The teleost adenohypophysis differs from that of many other vertebrates, since the different hormone-producing cell types are located in defined regions [25, 65]. The adenohypophysis is source of different hormones. Adrenocorticotropic hormone (ACTH) and prolactin are produced in different cells of the rostral pars distalis. In the proximal pars distalis, two gonadotropins (GT-1 and GT-2) (in trout, gonadotrops also appear in the rostral pars distalis), growth hormone (GH) and thyroid-stimulating hormone (TSH) are produced, whereas the pars intermedia is origin of somatolactin and melanocyte-stimulating-hormone (α-MSH) [2, 4, 36, 65, 220]. The functions of the hypophyseal hormones range from regulation of hormone synthesis and secretion (*e.g.,* of corticosteroids, thyroid hormones or melanin) to involvement in growth, up to reproduction or ionic- and metabolic regulation [2, 4, 25, 65, 220].

Macroscopic examination, preparation and sampling scheme for routine histopathological analyses of the rainbow trout pituitary gland

At necropsy, the pituitary gland is excised together with the central nervous system (CNS), as illustrated in detail in **Chapter 2.8**. Briefly, the CNS is removed after excision of the gills and the viscera (except the kidneys). The cranial vault, supraorbital bones and dorsal aspects of the vertebrae are carefully removed and the spinal cord is severed by a cross section ~1 cm caudal to the hindbrain. **Chapter 2.8** illustrates sampling and processing of all brain structures for histopathological and molecular analyses. After excision, macroscopic examination, weighing and (photo-) documentation (if appropriate), the specimens of the spinal cord and brain are generated. Through cautious dissection it is possible to remove the pituitary gland attached to the brain (**Figure 75A**), but in most cases the pituitary gland detaches during dissection and needs to be carefully removed separately, using fine forceps. For the histopathological examination of the pituitary gland independent of the sampling and processing of the brain specimens, it is recommended to generate a midsagittal section of the formalin-fixed and paraffin-embedded (FF-PE) (isolated) pituitary gland for the proper presentation of both the neuro- and the adenohypophysis (**Figure 75B**). The pituitary gland is embedded in agar prior to the processing for paraffin embedding, to maintain the orientation of the pituitary gland and ensure proper midsagittal section plane orientation. The FF-PE section is either stained with hematoxylin and eosin (HE) or with staining methods enabling an enhanced differentiation of the various cell types, *e.g.,* periodic acid-Schiff (PAS) or Heidenhain-Azan [36]. For an even more comprehensive investigation, some previously published studies examining rainbow trout also make use of serial sectioning of the pituitary gland [9, 221].

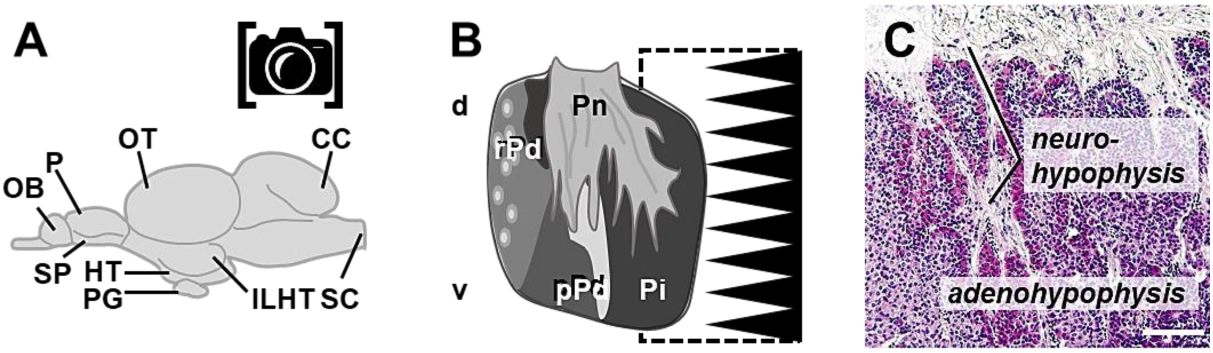

Figure 75. Illustration of the sampling for histopathological analyses of the pituitary gland of the rainbow trout. A. Schematic illustration of the lateral aspect of the excised brain with the ventrally attached pituitary gland. Important morphological structures are indicated: **OB:** Olfactory bulb; **P:** Pallium; **OT:** Optic tectum; **CC:** Cerebellar corpus; **SP:** Subpallium; **HT:** Hypothalamus; **PG:** Pituitary gland; **ILHT:** Inferior lobe of the hypothalamus; **SC:** Spinal cord. **B.** Schematic illustration of the midsagittally sectioned pituitary gland of the rainbow trout, the cranial aspect is to the left. The midsagittal section plane is indicated (black dashed rectangle with black triangles). The neurohypophysis/pars nervosa (**Pn**) is situated in the hypophyseal center and is source of *e.g.,* isotocin or vasotocin. The adenohypophysis is composed of a rostral (**rPd**) and proximal (**pPd**) pars distalis and a pars intermedia (**Pi**), all regions are source of different hormones: **rPd:** Adrenocorticotropic hormone (ACTH), endorphin or prolactin; **pPd:** Gonadotropin 1 and 2 (GT-1 and GT-2), growth hormone (GH) and thyroid-stimulating hormone (TSH); **Pi:** Somatolactin, β-endorphin or melanocyte-stimulating-hormone (α-MSH). **v:** Ventral; **d:** Dorsal. **C.** Midsagittal section of the neurohypophysis and the adenohypophysis of the pituitary gland of the rainbow trout. FF-PE. HE. Bar = 100 µm.

2.13.2 Endocrine pancreas

Relevant anatomical features

The rainbow trout pancreas is of the actinopterygian type, *i.e.,* it is scattered through the abdominal cavity. The exocrine pancreatic tissue is mainly located within the visceral adipose tissue between the pyloric ceca [4, 42, 65]. Endocrine pancreatic islets of Langerhans are scattered through the exocrine pancreatic tissue, additionally some pancreatic islets are fused to the "islet organ" or "Brockmann body" situated nearby the gall bladder. The aggregated islets of the islet organ are surrounded by exocrine pancreatic tissue, which in larger fish may invade the endocrine pancreatic tissue and subdivide it in smaller groups [4, 25, 42, 64, 65]. This compact structure is usually macroscopically visible, as illustrated in **Figure 76A**. The hormone synthesis and secretion of the endocrine pancreas proceeds in histologically and functionally distinct cell types: α-cells, β-cells, δ-cells and F-cells. The endocrine pancreatic hormones resemble that of other vertebrates: glucagon, insulin, somatostatin and pancreatic polypeptide [25]. In physiological state, islet changes may occur due to *e.g.,* age, migration, season or diet [3, 42, 65].

Macroscopic examination, preparation and sampling scheme for routine histopathological analyses of rainbow trout endocrine pancreas

The excision, macroscopic examination, sampling for histopathological and molecular analyses and further processing of the exocrine and endocrine pancreas is illustrated in detail in **Chapter 2.3.4**. Briefly, the pancreatic tissue is excised together with the gastrointestinal tract. For histopathological analysis of the endocrine islets scattered through the exocrine pancreatic tissue, two cross sections of the visceral adipose tissue located between the pyloric ceca are generated. The sampling regime proposed in **Chapter 2.3.4** is recommended for analyses of both compartments of the pancreas. If the primary focus is on the endocrine pancreas, it is recommended to divide the freshly excised islet organ by a transverse section in an anterior and a posterior portion. This way, a transverse section plane for histopathological examination is generated, the posterior half of the Brockmann body is immersion-fixed in neutrally buffered 4% formaldehyde solution, embedded in paraffin and the histological section is stained with HE. The remaining anterior half of the islet organ is either processed for further

analyses (*e.g.,* molecular analyses) or is preserved (*i.e.,* immediately transferred to an adequate fixative), to ensure that sample material is available for new/expanded scientific issues arising from the analyses.

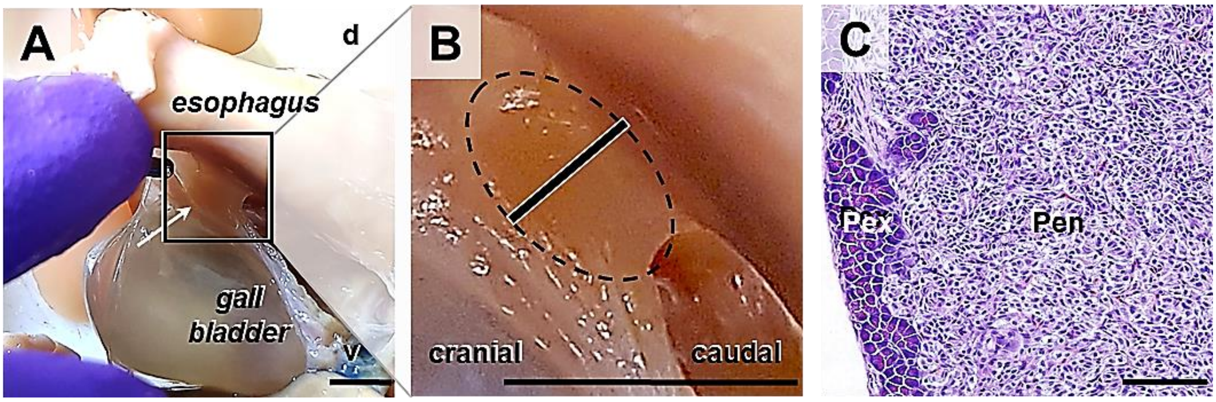

Figure 76. Illustration of the sampling for histopathological analyses of the endocrine pancreatic tissue of the rainbow trout. A, B. Lateral aspect of the dissected and formalin-fixed gall bladder and the esophagus. The Brockmann body is macroscopically visible within the mesentery (arrow). Note that the purpose of this figure is only to demonstrate the *in situ* location of the Brockmann body. In routine histopathological (and molecular) analyses, the specimen is generated in fresh and unfixed condition. The sample location and section plane orientation for histopathological analysis of the Brockmann body is indicated (black line) in the detail enlargement of the islet organ (encircled by the black dashed line) in **B**. **v:** Ventral; **d:** Dorsal. Bars = 1 cm. **C.** Histology of the Brockmann body. The Brockmann body is composed of endocrine pancreatic tissue (**Pen**), surrounded by exocrine pancreatic tissue (**Pex**). FF-PE. HE. Bar = 100 µm.

2.13.3 Thyroid gland

Relevant anatomical features

The thyroid gland of the rainbow trout is not a compact gland but consists of diffusely distributed thyroid follicles, predominantly localized in the ventral pharyngeal region adjacent to the ventral aorta [2-4]. As in mammals, the basic unit of the thyroid gland of adult rainbow trout are roundly shaped thyroid follicles, composed of colloid-filled follicular cavities lined by a simple cuboidal epithelium (in juvenile stages, rather tubular elements may be found) [4, 25, 42]. The histological staining characteristics of the colloid substance as well as the shape of the epithelial cells may vary depending on the glandular activity [36]. Under stimulation by adenohypophyseal TSH, synthesis and secretion of (predominantly) thyroxine (T4) occurs in the secretory active epithelial cells. The thyroid hormones are involved *inter alia* in regulation of growth, metabolism, reproduction and development [2, 4, 36].

Macroscopic examination, preparation and sampling scheme for routine histopathological analyses of the rainbow trout thyroid gland

In the external macroscopic examination, special attention should be paid to the skull and oral cavity, since various abnormalities in this region (*e.g.,* distortion of skull bones) may indicate pathological alterations of the thyroid gland [4]. At necropsy, the specimen for histopathological analysis of the thyroid follicle is generated from the floor of the gill basket (*i.e.,* the base of the gill arches) (**Figure 77A**), therefore the left and right gill arches are excised as illustrated in Fiedler et al. [13] and in **Chapter 2.1**. For isolation of the floor of the gill basket from the remaining gill apparatus, the ventral aspects of the gill arches are carefully severed, care is taken to not damage the fragile gill filaments. Additionally, the tongue is also separated from the floor of the gill basket by a transverse section (refer to **Chapter 2.3.1**). Subsequently, the gill basket base is transferred to an adequate fixative (neutrally buffered 4% formaldehyde solution) and subsequently to a slow-acting decalcification solution for ~2-3 days. A midsagittal section is generated for histopathological analyses. The formalin-fixed, decalcified and

paraffin-embedded (FF-PE) histological section is stained with hematoxylin and eosin (HE) (**Figure 77B**).

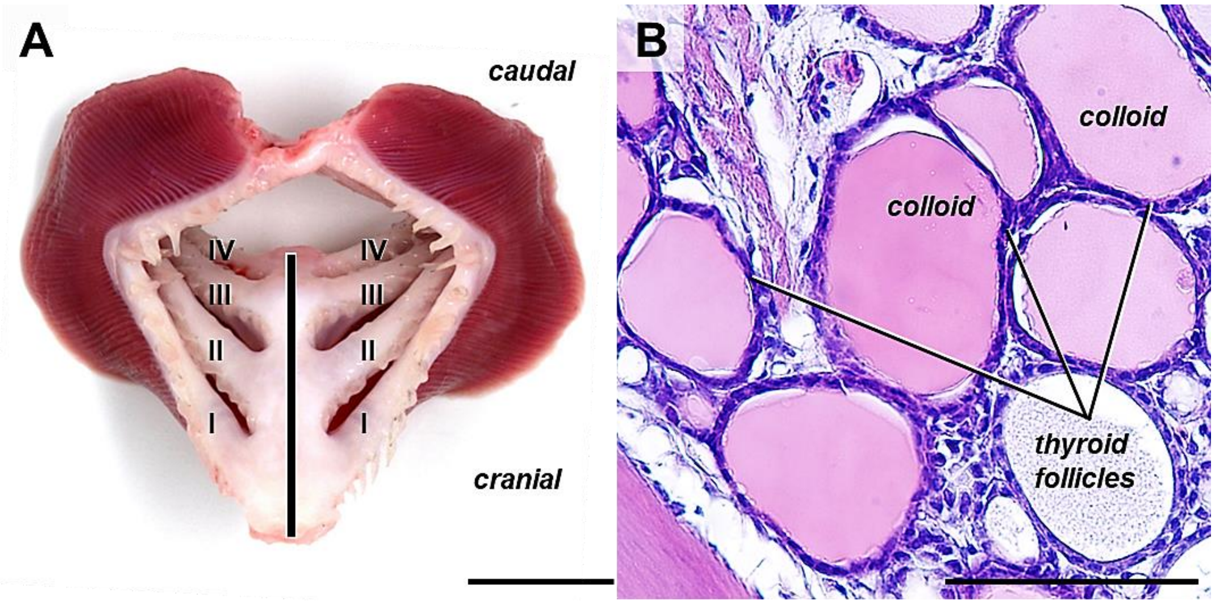

Figure 77. Illustration of the sampling for histopathological analyses of the thyroid gland. A. Cranio-dorsal aspect of the freshly excised gill basket. The four gill arches are numbered (**I-IV**) and the sample location and section plane orientation for histopathological analysis of the thyroid gland is indicated (black line). Bar = 1 cm. **B.** Histology of the thyroid follicles, composed of a single epithelium of cuboidal cells, surrounding the colloid-filled lumen. FF-PE. HE. Bar = 100 µm.

2.13.4 Interrenal and suprarenal tissue

Relevant anatomical features

A discrete, compact adrenal gland is missing in the rainbow trout. The analog of the mammalian adrenal cortex is the interrenal tissue, composed of corticosteroid hormone-producing cells. The interrenal cells are closely associated with the chromaffin (suprarenal) cells, which are the analog of the adrenal medullary cells and produce catecholamines. The inter- and suprarenal cells built a diffuse organ residing within the head kidney tissue, which is closely associated with the postcardinal veins [1, 2, 4, 25, 42]. Histologically, the steroidogenic cells appear as pale cells with round nuclei and prominent nucleoli and are partially arranged in an epithelial-like pattern (**Figure 78A**). Their histological appearance may vary during the reproduction cycle or *e.g.,* due to stress, pharmaceuticals or changes of the salinity of the aquatic environment [4, 42]. Compared to the steroidogenic cells, catecholaminergic cells histologically stain weaker and the cell nuclei are less uniformly shaped (**Figure 78B**), but their appearance also varies in dependence on different exogenous and endogenous influence factors [4, 36, 42]. The interrenal cells mainly produce and secrete cortisol, which is an important component of *e.g.,* the stress response, ion- and water balance or energy metabolism. The chromaffin cells produce *e.g.,* adrenalin, noradrenalin or dopamine, which are *inter alia* involved in the regulation of the blood supply of various organs or the regulation of cardiac contraction and heart rate [2, 4, 25].

Macroscopic examination, preparation and sampling scheme for routine histopathological analyses of rainbow trout interrenal and suprarenal tissue

At necropsy, the inter- and suprarenal tissue is excised together with the head kidney, the preparation and sampling scheme for routine histopathological analyses of the rainbow trout kidney is illustrated in detail in **Chapter 2.7**. Briefly, the head kidney is assessed after removal of the other viscera. After macroscopic examination, the sample for molecular analyses is generated. Due to the soft consistency of the kidney, the remaining renal tissue stays connected to the spine (as *in situ*) after trimming of the trunk for enhanced immersion fixation.

The tissue is transferred to an adequate fixation solution (neutrally buffered 4% formaldehyde solution), for histopathological analyses of the inter- and suprarenal tissue, a transverse section plane is generated, containing the ventral and the dorsal organ capsule (**Chapter 2.7**). The FF-PE histological section is stained with HE or with Masson`s trichrome stain, if the different histochemical characteristics of the inter- and suprarenal cells are to be displayed more prominently [36].

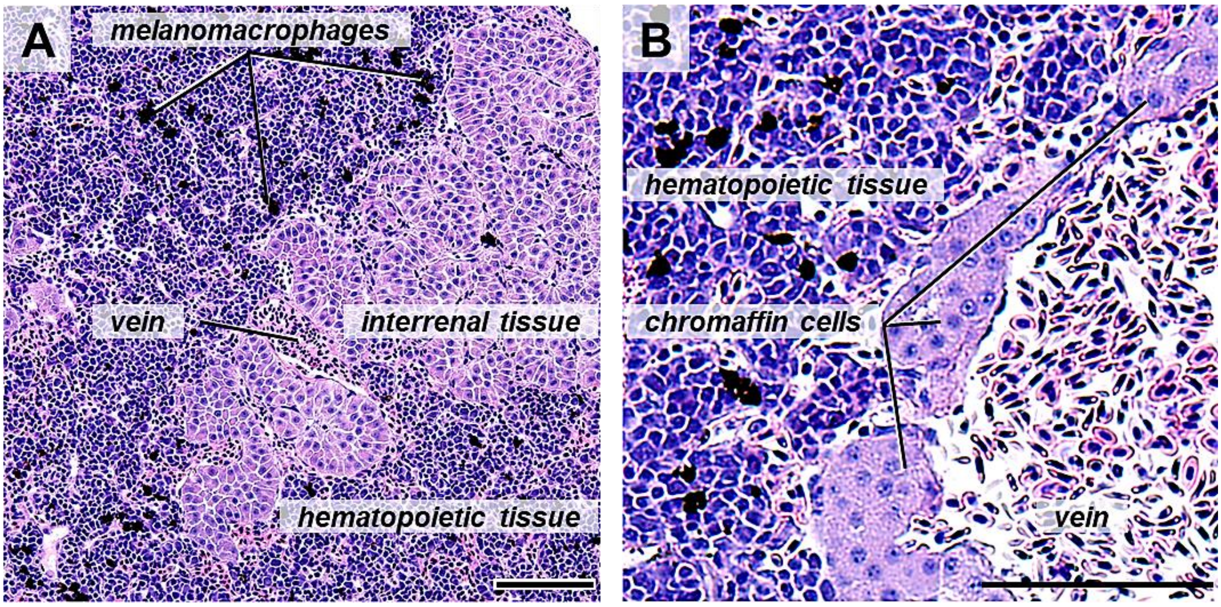

Figure 78. Histopathology of the interrenal and suprarenal tissue of the rainbow trout. A. Transverse section of the interrenal tissue residing within the anterior (head) kidney of the rainbow trout. The corticosteroid-producing cells of the interrenal tissue are situated within the hematopoietic tissue, in association with the postcardinal veins. **B.** Transverse section of the chromaffin cells of the suprarenal tissue. The chromaffin cells usually are located around the postcardinal veins in close association with the steroidogenic cells, but may also appear as isolated islets within the tissue of the head kidney. FF-PE. HE. Bars = 100 µm.

2.13.5 Corpuscles of Stannius

Relevant anatomical features

The corpuscles of Stannius (CS) are exclusively found in teleosts (and holostean fish). The CS of salmonids are discrete structures situated in the parenchyma of the trunk kidney, surrounded by a capsule. In rainbow trout, the CS number usually ranges between 2 to 6, and they are commonly visible as whitish nodular structures on the kidney surface (**Figure 79**) [3, 4, 42]. Septa of connective tissue organize the glandular tissue into lobules which comprise epithelial cells surrounding the central cavities of the lobules (*i.e.,* the pseudolumina). The CS are richly supplied with blood vessels and nerves, excretory ducts are missing [1, 4, 36, 42] (**Figure 80**). The hormone produced and secreted by the CS is stanniocalcin, a glycoprotein involved in the control and regulation of the calcium homeostasis [2, 4, 25].

Macroscopic examination, preparation and sampling scheme for routine histopathological analyses of the rainbow trout corpuscles of Stannius

At necropsy, the sample for the histopathological analyses of the corpuscles of Stannius is excised from the trunk kidney, the preparation and sampling scheme for routine histopathological analyses of the rainbow trout trunk kidney is illustrated in detail in **Chapter 2.7**. Briefly, the kidneys are assessed after removal of the other viscera, the samples for molecular analyses are generated after macroscopic examination from the fresh (*i.e.,* unfixed) tissue. Due to the soft consistency of the renal tissue, the remaining kidneys stay connected to the spine (as *in situ)* and the trunk is trimmed for an enhanced immersion fixation. The tissue is transferred to an adequate fixation solution (neutrally buffered 4% formaldehyde

solution) and for histopathological analyses of the CS, a transverse section plane is generated, containing the CS together with the entire section profile of the trunk kidney. The CS are usually macroscopically visible as small whitish structures situated in the renal parenchyma (**Figure 79**). Their number and anatomical distribution is quite variable, so no fixed sample location is determined.

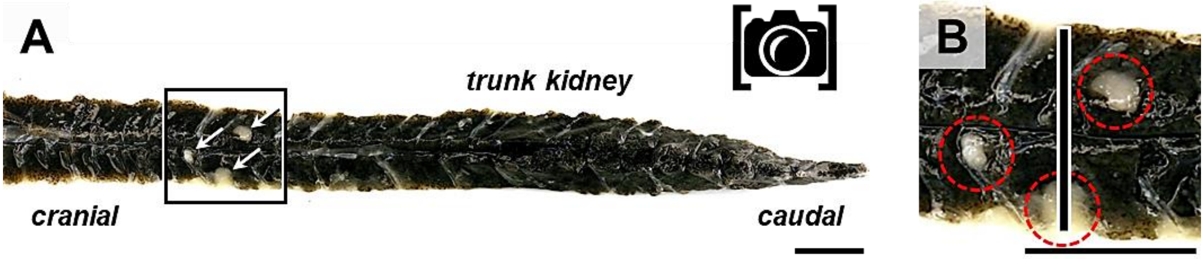

Figure 79. Illustration of the sampling for histopathological analyses of the corpuscles of Stannius of the rainbow trout. A. Dorsal aspect of the formalin-fixed, isolated trunk kidney of the rainbow trout. Three corpuscles of Stannius are visible as whitish, nodular structures embedded in the trunk kidney parenchyma (white arrows). The black rectangle marks the trunk kidney region containing the corpuscles of Stannius (refer to **B**). **B.** Detail enlargement of the trunk kidney region containing three macroscopically visible corpuscles of Stannius (red dashed circles). The sample location and section plane orientation for histopathological analysis of the corpuscles of Stannius is indicated (black line). Bars = 1 cm.

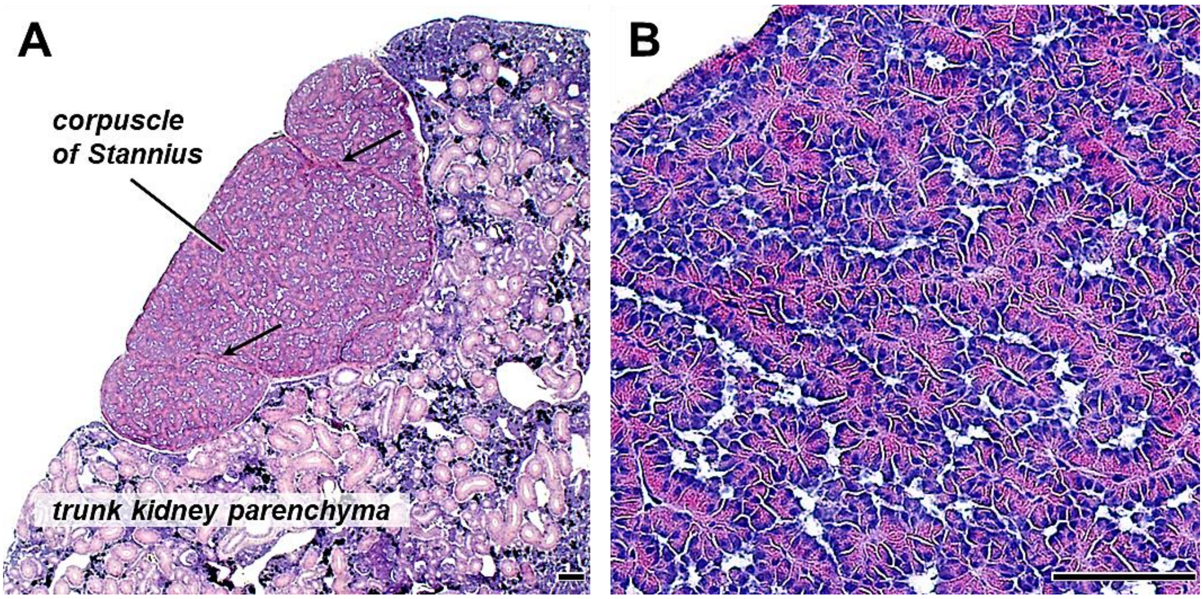

Figure 80. Illustration of the sampling for histopathological analyses of the corpuscles of Stannius of the rainbow trout. A. Transverse section of the corpuscle of Stannius (CS), embedded in the trunk kidney parenchyma. The glandular CS tissue is organized into lobules by septa of connective tissue (black arrows). **B.** Detail enlargement of the glandular CS tissue. FF-PE. HE. Bars = 100 µm.

2.13.6 Pineal gland (Epiphysis)

Relevant anatomical features

The pineal gland (epiphysis) of rainbow trout is a neuroendocrine structure, situated in the median dorsal to the diencephalon and anterior to the tectum of the mesencephalon (**Figure 81A**) [4, 36, 42]. It is surrounded by a capsule of connective tissue, and comprises a broadened saccular body (*i.e.,* the pineal end-vesicle) and a pineal stalk whose lumen is continuous with the third brain ventricle [25, 42, 222]. The pineal organ is composed of pinealocytes (*i.e.,* photosensitive and secretory active receptor cells), ependymal cells and neurons, and is highly vascularized [25, 42, 222]. The pineal gland is a photosensory organ. The photoperiod information modulates the pineal synthesis and release of melatonin, an indoleamine which is involved in *e.g.,* growth- or reproduction processes or the regulation of circadian locomotor activity [2, 25, 222].

Macroscopic examination, preparation and sampling scheme for routine histopathological analyses of the rainbow trout pineal gland

At necropsy, the pineal gland is excised together with the central nervous system (CNS), as illustrated in detail in **Chapter 2.8**. Briefly, the CNS is removed after excision of the gills and viscera. The dorsal aspects of the vertebrae, the cranial vault and supraorbital bones are removed, care is taken to not damage the pineal gland which is situated adjacent to the dorsal cranial roof. The spinal cord is severed by a cross section ~1 cm caudal to the hindbrain. After excision, the brain and the attached pineal gland are macroscopically examined and alterations are photographically documented, if appropriate. The sampling regime for histopathological and molecular analyses of the spinal cord and brain presented in **Chapter 2.8** is not suitable for optimal histopathological presentation of the pineal gland. Therefore, if no analyses of further brain structures are scheduled, the whole brain with the attached pineal gland is immersion-fixed using an adequate fixative (neutrally buffered 4% formaldehyde solution) to retain the *in situ* orientation and localization of the epiphysis, and a midsagittal section of the FF-PE pineal gland and brain is generated (**Figure 81A**). If immersion fixation of the entire brain is not in conformity with the study design, the epiphysis is carefully detached from the brain (as illustrated in a previous ecotoxicological study using rainbow trout by for molecular analyses [217]) and immersion-fixed separately. A midsagittal section of the FF-PE pineal tissue is generated and stained with HE.

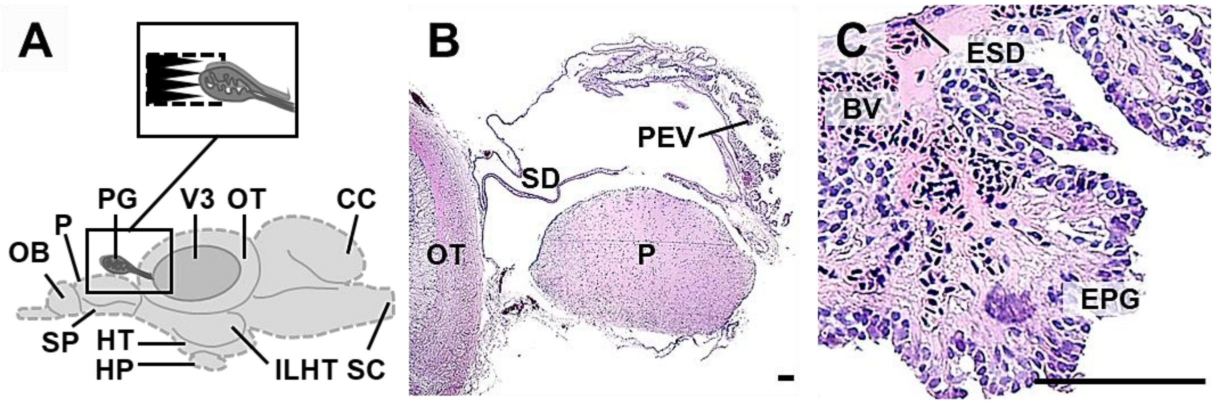

Figure 81. Illustration of the sampling for histopathological analyses of the pineal gland of the rainbow trout. A. Schematic illustration of the lateral aspect of the midsagittally sectioned brain of the rainbow trout with the attached pineal gland. Important morphological structures are indicated: **OB:** Olfactory bulb; **P:** Pallium; **PG:** Pineal gland; **V3:** Third brain ventricle; **OT:** Optic tectum; **CC:** Cerebellar corpus; **SP:** Subpallium; **HT:** Hypothalamus; **HP:** Hypophysis (pituitary gland); **ILHT:** Inferior lobe of the hypothalamus; **SC:** Spinal cord. The illustration represents a section of the brain with attached pineal gland and maintained physiological position of the epiphysis. The detail enlargement of the epiphysis (black frame) illustrates the sampling for histopathological analyses of a pineal gland detached from the brain, the black dashed rectangle with the black triangles indicates the section plane orientation parallel to the paper plane and the anterio-posterior axis of the pineal gland.

B, C: Histopathology of the pineal gland of the rainbow trout. Midsagittal section of the pineal gland, important morphological structures are indicated: **OT:** Optic tectum; **P:** Pallium; **SD:** Saccus dorsalis; **PEV:** Pineal end-vesicle; **BV:** Blood vessel; **ESD:** Epithelium of the saccus dorsalis; **EPG:** Epithelium of the pineal gland. FF-PE. HE. Bars = 100 µm.

2.13.7 Urophysis

Relevant anatomical features

The urophysis (also called neuro-hemal complex) is a fish-specific neurosecretory system with a structure similar to the pituitary neurohypophysis [2, 4, 25, 42]. The urophysis is a small, highly vascularized expansion of the ventral aspect of the posterior end of the spinal cord at the level of the last vertebrae. It is composed of the axons of the Dahlgren cells (*i.e.,* specialized neurosecretory neurons situated in the spinal cord), which terminate in the urophysis and secrete urotensin hormones (urotensin I and II) [1, 2, 4, 42]. The axons are in close association to blood capillaries and the urophysis is surrounded by loose connective tissue [42]. The urophyseal hormones are *inter alia* involved in osmoregulation and are of vasoactive, lipogenic and ionoregulatory effect [2, 4].

Macroscopic examination, preparation and sampling scheme for routine histopathological analyses of the rainbow trout urophysis

At necropsy, the specimen for histopathological analyses of the urophysis is excised after sampling of the gills, viscera, central nervous system (CNS), integument, locomotor- and sensory system. For histopathological analyses of the neurohemal organ, the most posterior ~1.5 cm of the spine (the urophysis sample is generated directly posterior to the sample location for vertebral bone analyses) is excised by two cross sections. Adhering skin and skeletal muscle are spaciously removed for subsequent immersion fixation in neutrally buffered 4% formaldehyde solution and decalcification with a slow-acting decalcification solution for approximately 5-7 days. The lateral aspects of the centra of the formalin-fixed and decalcified vertebrae are removed using a microtome blade and the remaining vertebral column is embedded in paraffin. A midsagittal section of the urophysis within the neural arch of the neural spine is generated and stained with HE.

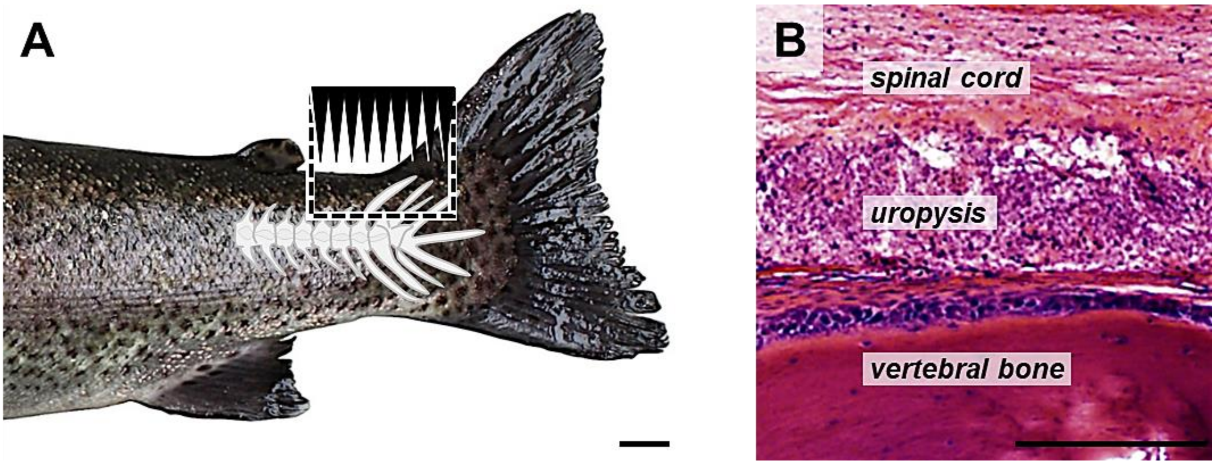

Figure 82. Illustration of the sampling for histopathological analyses of the urophysis of the rainbow trout. A. Lateral aspect of the caudal region of the rainbow trout with the schematically indicated posterior vertebral column and caudal fin support. The sample location and section plane orientation are indicated (black dashed rectangle with black triangles, indicating the section plane orientation parallel to the picture plane), the neural spine with the neural arch containing the urophysis (ventral to the spinal cord), is sectioned midsagittally. Bar = 1 cm. **B.** Histology of the urophysis. Sagittal section of the urophysis, situated ventral to the spinal cord within the neural canal of the neural spine. FF-PE. HE. Bar = 100 µm.

2.13.8 Ultimobranchial gland

Relevant anatomical features

The ultimobranchial gland (also called suprapericardial, postbranchial or subesophagial gland/body) of the rainbow trout is assumed to be the functional equivalent to the mammalian parathyroid gland, which is missing in fish [1, 3, 223]. In salmonids, the ultimobranchial gland is a paired gland, situated in the transverse septum ventral to the esophagus [4, 42, 224]. The ultimobranchial bodies are surrounded by a capsule of connective tissue, a thin sheet of connective tissue separates the two lobes in the midline. An epithelium composed of columnar epithelial cells and some goblet cells is lining irregularly shaped cysts/follicles within the glandular tissue [1, 4, 42, 224, 225]. The ultimobranchial gland is responsible for the synthesis and secretion of calcitonin, which is assumed to be a calcium-regulating hormone [2, 4, 225].

Macroscopic examination, preparation and sampling scheme for routine histopathological analyses of the rainbow trout ultimobranchial gland

At necropsy, the ultimobranchial bodies are sampled after excision and sampling of the gills (illustrated in detail in **Chapter 2.1**). For excision of the gill apparatus, the transverse septum (*i.e.,* the septum separating the heart from the abdominal cavity (**Figure 83A**)) is excised from the body of the rainbow trout after transection of the esophagus. The gill apparatus is separated from the attached organs/tissues (*e.g.,* heart, pharynx, cranial portion of the esophagus and transverse septum). After removal of the heart, the transverse septum with the cranial portion of the esophagus and a portion of the sinus venosus is immersion-fixed in neutrally buffered 4% formaldehyde solution. Since the glandular bodies cannot always be accurately identified macroscopically, the entire ventral portion of the septum (with portions of esophagus and sinus venosus) is embedded in paraffin, with the front side down. This way, a transverse section plane is created. The FF-PE section is stained with HE.

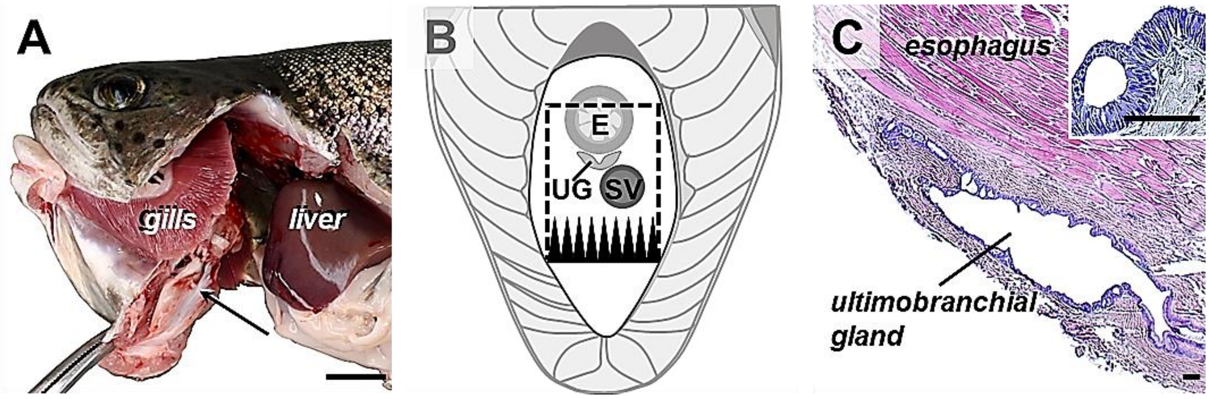

Figure 83. Photographic and schematic illustration of the sampling for histopathological analyses of the ultimobranchial gland of the rainbow trout. A. Photographic illustration of the *in situ* localization of the transverse septum (black arrow) after opening of the abdominal cavity and transection of the cranial portion of the esophagus. The transverse septum separates the abdominal cavity and the pericardial vault. Bar = 1 cm. **B.** Schematic illustration of the cranial aspect of the transverse septum (as *in situ*), containing a portion of the esophagus (**E**) and of the sinus venosus (**SV**). The paired ultimobranchial gland (**UG**) is located within the transverse septum, ventral to the esophagus and dorsal to the sinus venosus. The sample location and section plane orientation of the specimen for histopathological analyses are indicated, the black dashed rectangle with the black triangles indicates the section plane orientation parallel to the paper plane. **C.** Histology of the ultimobranchial gland. A detail enlargement of the epithelium of the ultimobranchial gland is shown in the inset. Bars = 100 µm.

3. References

1. Stoskopf MK. Fish Medicine. 1st ed. Philadelphia: W. B. Saunders Company; 1993.
2. Ostrander GK. The Laboratory Fish. 1st ed. San Diego: Academic Press; 2000.
3. Roberts RJ. Fish Pathology. 4th ed. Oxford: Blackwell Publishing Ltd.; 2012.
4. Ferguson HW. Systemic Pathology of Fish: A Text and Atlas of Normal Tissues in Teleosts and their Response in Disease. 2nd ed. London: Scotian Press; 2006.
5. Danion M, Le Floch S, Pannetier P, Van Arkel K, Morin T. Transchem project – Part I: Impact of long-term exposure to pendimethalin on the health status of rainbow trout (Oncorhynchus mykiss L.) genitors. Aquatic Toxicology. 2018;202: 207-215. doi: 10.1016/j.aquatox.2018.07.002.
6. De Boeck G, Ngo TTH, Van Campenhout K, Blust R. Differential metallothionein induction patterns in three freshwater fish during sublethal copper exposure. Aquatic Toxicology. 2003;65(4): 413-424. doi: 10.1016/S0166-445X(03)00178-4.
7. Domoradzki JY, Sushynski JM, Thackery LM, Springer TA, Ross TL, Woodburn KB, et al. Metabolism of ^{14}C-octamethylcyclotetrasiloxane ([^{14}C]D_4) or ^{14}C-decamethylcyclopentasiloxane ([^{14}C]D_5) orally gavaged in rainbow trout (Oncorhynchus mykiss). Toxicology Letters. 2017;279(Suppl 1): 115-124. doi: 10.1016/j.toxlet.2017.03.025.
8. Giesy JP, Jones PD, Kannan K, Newsted JL, Tillitt DE, Williams LL. Effects of chronic dietary exposure to environmentally relevant concentrations to 2,3,7,8-tetrachlorodibenzo-p-dioxin on survival, growth, reproduction and biochemical responses of female rainbow trout (Oncorhynchus mykiss). Aquatic Toxicology. 2002;59(1-2): 35-53. doi: 10.1016/S0166-445X(01)00235-1.
9. Ruby SM, Hull R, Anderson P. Sublethal Lead Affects Pituitary Function of Rainbow Trout During Exogenous Vitellogenesis. Archives of Environmental Contamination and Toxicology. 2000;38(1): 46-51. doi: 10.1007/s002449910006.
10. Schwaiger J, Mallow U, Ferling H, Knoerr S, Braunbeck T, Kalbfus W, et al. How estrogenic is nonylphenol? A transgenerational study using rainbow trout (Oncorhynchus mykiss) as a test organism. Aquatic Toxicology. 2002;59(3-4): 177-189. doi: 10.1016/S0166-445X(01)00248-X.
11. Geiger T. Untersuchung zur Wirkung oral aufgenommener PVC-Mikroplastikpartikel bei Regenbogenforellen (Oncorhynchus mykiss). Doctoral Thesis, Ludwig-Maximilians-Universität München. 2021. Available from: https://edoc.ub.uni-muenchen.de/28405/.
12. Karlsson-Norrgren L, Runn P, Haux C, Förlin L. Cadmium-induced changes in gill morphology of zebrafish, Brachydanio rerio (Hamilton–Buchanan), and rainbow trout, Salmo gairdneri Richardson. Journal of Fish Biology. 1985;27(1): 81-95. doi: 10.1111/j.1095-8649.1985.tb04011.x.
13. Fiedler S, Wünnemann H, Hofmann I, Theobalt N, Feuchtinger A, Walch A, et al. A practical guide to unbiased quantitative morphological analyses of the gills of rainbow trout (Oncorhynchus mykiss) in ecotoxicological studies. PLoS ONE. 2020;15(12): e0243462. doi: 10.1371/journal.pone.0243462.
14. Nagy ZT. A hands-on overview of tissue preservation methods for molecular genetic analyses. Organisms Diversity & Evolution. 2010;10(1): 91-105. doi: 10.1007/s13127-010-0012-4.
15. Howard CV, Reed GR. Unbiased Stereology: Three-dimensional Measurement in Microscopy. 2nd ed. Abingdon: Garland Science/BIOS Scientific Publishers; 2005.
16. Gundersen HJG, Mirabile R, Brown D, Boyce RW. Chapter 8 - Stereological Principles and Sampling Procedures for Toxicologic Pathologists. In: Haschek WM, Rousseaux CG, Wallig MA, editors. Haschek and Rousseaux's Handbook of Toxicologic Pathology. Boston: Academic Press; 2013. pp. 215-286.
17. Weibel ER. Stereological Methods: Practical Methods for Biological Morphometry. 1st ed. London: Academic Press; 1979.
18. Nyengaard JR. Stereologic Methods and Their Application in Kidney Research. Journal of the American Society of Nephrology. 1999;10(5): 1100-1123. doi: 10.1681/ASN.V1051100.
19. Hsia CCW, Hyde DM, Ochs M, Weibel ER. An Official Research Policy Statement of the American Thoracic Society/European Respiratory Society: Standards for Quantitative Assessment of Lung Structure. American Journal of Respiratory and Critical Care Medicine. 2010;181(4): 394-418. doi: 10.1164/rccm.200809-1522ST.
20. Slomianka L. Basic quantitative morphological methods applied to the central nervous system. Journal of Comparative Neurology. 2021;529(4): 694-756. doi: 10.1002/cne.24976.
21. Albl B, Haesner S, Braun-Reichhart C, Streckel E, Renner S, Seeliger F, et al. Tissue Sampling Guides for Porcine Biomedical Models. Toxicologic Pathology. 2016;44(3): 414-420. doi: 10.1177/0192623316631023.
22. Blutke A, Wanke R. Sampling Strategies and Processing of Biobank Tissue Samples from Porcine Biomedical Models. Journal of Visualized Experiments. 2018(133): e57276. doi: 10.3791/57276.

23. Abbott A. Inside the first pig biobank. Nature. 2015;519(7544): 397-398. doi: 10.1038/519397a.
24. Evans DH, Piermarini PM, Choe KP. The multifunctional fish gill: dominant site of gas exchange, osmoregulation, acid-base regulation, and excretion of nitrogenous waste. Physiological Reviews. 2005;85(1): 97-177. doi: 10.1152/physrev.00050.2003.
25. Evans DH, Clairborne JB. The physiology of fishes. 3rd ed. Boca Raton: CRC Press Taylor & Francis Group; 2006.
26. Waser WP, Schmitz A, Perry SF, Wobschall A. Stereological Analysis of Blood Space and Tissue Types in the Pseudobranch of the Rainbow Trout (Oncorhynchus mykiss). Fish Physiology and Biochemistry. 2005;31(1): 73-82. doi: 10.1007/s10695-006-0002-9.
27. Wilson JM, Laurent P. Fish gill morphology: Inside out. Journal of Experimental Zoology. 2002;293(3): 192-213. doi: 10.1002/jez.10124.
28. Boyle D, Al-Bairuty GA, Ramsden CS, Sloman KA, Henry TB, Handy RD. Subtle alterations in swimming speed distributions of rainbow trout exposed to titanium dioxide nanoparticles are associated with gill rather than brain injury. Aquatic Toxicology. 2013;126: 116-127. doi: 10.1016/j.aquatox.2012.10.006.
29. Strzyżewska-Worotyńska E, Szarek J, Babińska I, Gulda D. Gills as morphological biomarkers in extensive and intensive rainbow trout (Oncorhynchus mykiss, Walbaum 1792) production technologies. Environmental Monitoring and Assessment. 2017;189(12): 611. doi: 10.1007/s10661-017-6278-7.
30. Dethloff GM, Schlenk D, Hamm JT, Bailey HC. Alterations in Physiological Parameters of Rainbow Trout (Oncorhynchus mykiss) with Exposure to Copper and Copper/Zinc Mixtures. Ecotoxicology and Environmental Safety. 1999;42(3): 253-264. doi: 10.1006/eesa.1998.1757.
31. Pane EF, Haque A, Goss GG, Wood CM. The physiological consequences of exposure to chronic, sublethal waterborne nickel in rainbow trout (Oncorhynchus mykiss): exercise vs resting physiology. Journal of Experimental Biology. 2004;207(Pt 7): 1249-1261. doi: 10.1242/jeb.00871.
32. Beijer K, Jönsson M, Shaik S, Behrens D, Brunström B, Brandt I. Azoles additively inhibit cytochrome P450 1 (EROD) and 19 (aromatase) in rainbow trout (Oncorhynchus mykiss). Aquatic Toxicology. 2018;198: 73-81. doi: 10.1016/j.aquatox.2018.02.016.
33. Ellesat KS, Holth TF, Wojewodzic MW, Hylland K. Atorvastatin up-regulate toxicologically relevant genes in rainbow trout gills. Ecotoxicology. 2012;21(7): 1841-1856. doi: 10.1007/s10646-012-0918-z.
34. Nolan DT, Spanings FAT, Ruane NM, Hadderingh RH, Jenner HA, Wendelaar Bonga SE. Exposure to water from the lower Rhine induces a stress response in the rainbow trout Oncorhynchus mykiss. Archives of Environmental Contamination and Toxicology. 2003;45(2): 247-257. doi: 10.1007/s00244-002-0068-z.
35. Poppe TT, Johansen R, Gunnes G, Tørud B. Heart morphology in wild and farmed Atlantic salmon Salmo salar and rainbow trout Oncorhynchus mykiss. Diseases of Aquatic Organisms. 2003;57(1-2): 103-108. doi: 10.3354/dao057103.
36. Yasutake WT, Wales JH. Microscopic Anatomy of Salmonids: An Atlas. 1st ed. Washington, D.C: United States Deptartment of the Interior, Fish and Wildlife Service; 1983.
37. Graham MS, Farrell AP. Environmental influences on cardiovascular variables in rainbow trout, Oncorhynchus mykiss (Walbaum). Journal of Fish Biology. 1992;41(5): 851-858. doi: 10.1111/j.1095-8649.1992.tb02713.x.
38. Cao Z, Tanguay RL, McKenzie D, Peterson RE, Aiken JM. Identification of a putative calcium-binding protein as a dioxin-responsive gene in zebrafish and rainbow trout. Aquatic Toxicology. 2003;63(3): 271-282. doi: 10.1016/s0166-445x(02)00184-4.
39. Ates B, Orun I, Talas ZS, Durmaz G, Yilmaz I. Effects of sodium selenite on some biochemical and hematological parameters of rainbow trout (Oncorhynchus mykiss Walbaum, 1792) exposed to Pb^{2+} and Cu^{2+}. Fish Physiology and Biochemistry. 2008;34(1): 53-59. doi: 10.1007/s10695-007-9146-5.
40. Incardona JP, Carls MG, Holland L, Linbo TL, Baldwin DH, Myers MS, et al. Very low embryonic crude oil exposures cause lasting cardiac defects in salmon and herring. Scientific Reports. 2015;5: 13499. doi: 10.1038/srep13499.
41. Abbate F, Guerrera MC, Levanti M, Laurà R, Aragona M, Mhalhel K, et al. Anatomical, histological and immunohistochemical study of the tongue in the rainbow trout (Oncorhynchus mykiss). Anatomia, Histologia, Embryologia. 2020;49(6): 848-858. doi: 10.1111/ahe.12593.
42. Blüm V, Casado J, Lehmann J, Mehring E. Farbatlas der Histologie der Regenbogenforelle: Begleitheft mit Einführung in die Makroskopische Anatomie der Regenbogenforelle. Einführung in die Gewebelehre. Färbevorschriften. 1st ed. Berlin Heidelberg: Springer; 1989.
43. Kasumyan AO. The taste system in fishes and the effects of environmental variables. Journal of Fish Biology. 2019;95(1): 155-178. doi: 10.1111/jfb.13940.

44. Meyer-Rochow VB. Fish tongues - surface fine structures and ecological considerations. Zoological Journal of the Linnean Society. 1981;71(4): 413-426. doi: 10.1111/j.1096-3642.1981.tb01137.x.
45. Berkovitz BKB, Moore MH. Tooth Replacement in the Upper Jaw of the Rainbow Trout (Salmo gairdneri). Journal of Experimental Zoology. 1975;193(2): 221-234. doi: 10.1002/jez.1401930211.
46. Hampton JA, McCuskey PA, McCuskey RS, Hinton DE. Functional units in rainbow trout (Salmo gairdneri) liver: I. Arrangement and histochemical properties of hepatocytes. The Anatomical Record. 1985;213(2): 166-175. doi: 10.1002/ar.1092130208.
47. Gage GJ, Kipke DR, Shain W. Whole Animal Perfusion Fixation for Rodents. Journal of Visualized Experiments. 2012(65): e3564. doi: 10.3791/3564.
48. Hinton DE. Perfusion Fixation of Whole Fish for Electron Microscopy. Journal of the Fisheries Research Board of Canada. 1975;32(3): 416-422. doi: 10.1139/f75-051.
49. Hille S. A literature review of the blood chemistry of rainbow trout, Salmo gairdneri Rich. Journal of Fish Biology. 1982;20(5): 535-569. doi: 10.1111/j.1095-8649.1982.tb03954.x.
50. Kiceniuk JW, Jones DR. The Oxygen Transport System in Trout (Salmo Gairdneri) During Sustained Exercise. Journal of Experimental Biology. 1977;69(1): 247-260.
51. Wood CM, Shelton G. Cardiovascular dynamics and adrenergic responses of the rainbow trout in vivo. Journal of Experimental Biology. 1980;87(1): 247-270. doi: 10.1242/jeb.87.1.247.
52. Olson KR. Preparation of fish tissues for electron microscopy. Journal of Electron Microscopy Technique. 1985;2(3): 217-228. doi: 10.1002/jemt.1060020307.
53. Speilberg L, Evensen Ø, Nafstad P. Liver of Juvenile Atlantic Salmon, Salmo salar L.: A Light, Transmission, and Scanning Electron Microscopic Study, With Special Reference to the Sinusoid. The Anatomical Record. 1994;240(3): 291-307. doi: 10.1002/ar.1092400302.
54. Stevens ED, Randall DJ. Changes in Blood Pressure, Heart Rate and Breathing Rate During Moderate Swimming Activity in Rainbow Trout. Journal of Experimental Biology. 1967;46(2): 307-315. doi: 10.1242/jeb.46.2.307.
55. Triebskorn R, Casper H, Scheil V, Schwaiger J. Ultrastructural effects of pharmaceuticals (carbamazepine, clofibric acid, metoprolol, diclofenac) in rainbow trout (Oncorhynchus mykiss) and common carp (Cyprinus carpio). Analytical and Bioanalytical Chemistry. 2007;387(4): 1405-1416. doi: 10.1007/s00216-006-1033-x.
56. Mehinto AC, Hill EM, Tyler CR. Uptake and biological effects of environmentally relevant concentrations of the nonsteroidal anti-inflammatory pharmaceutical diclofenac in rainbow trout (Oncorhynchus mykiss). Environmental Science & Technology. 2010;44(6): 2176-2182. doi: 10.1021/es903702m.
57. Woźny M, Brzuzan P, Luczyński MK, Góra M, Wolińska L, Bukowski R, et al. CYP1A expression in liver and gills of rainbow trout (Oncorhynchus mykiss) after short-term exposure to dibenzothiophene (DBT). Chemosphere. 2010;79(1): 110-112. doi: 10.1016/j.chemosphere.2010.01.063.
58. Frew JA, Brown JT, Fitzsimmons PN, Hoffman AD, Sadilek M, Grue CE, et al. Toxicokinetics of the neonicotinoid insecticide imidacloprid in rainbow trout (Oncorhynchus mykiss). Comparative Biochemistry and Physiology Part C: Toxicology & Pharmacology. 2018;205: 34-42. doi: 10.1016/j.cbpc.2018.01.002.
59. Petala M, Kokokiris L, Samaras P, Papadopoulos A, Zouboulis A. Toxicological and ecotoxic impact of secondary and tertiary treated sewage effluents. Water Research. 2009;43(20): 5063-5074. doi: 10.1016/j.watres.2009.08.043.
60. Chowdhury MJ, Baldisserotto B, Wood CM. Tissue-Specific Cadmium and Metallothionein Levels in Rainbow Trout Chronically Acclimated to Waterborne or Dietary Cadmium. Archives of Environmental Contamination and Toxicology. 2005;48(3): 381-390. doi: 10.1007/s00244-004-0068-2.
61. Woźny M, Obremski K, Hliwa P, Gomułka P, Różyński R, Wojtacha P, et al. Feed contamination with zearalenone promotes growth but affects the immune system of rainbow trout. Fish and Shellfish Immunology. 2019;84: 680-694. doi: 10.1016/j.fsi.2018.10.032.
62. Alves LC, Wood CM. The chronic effects of dietary lead in freshwater juvenile rainbow trout (Oncorhynchus mykiss) fed elevated calcium diets. Aquatic Toxicology. 2006;78(3): 217-232. doi: 10.1016/j.aquatox.2006.03.005.
63. Chowdhury MJ, McDonald DG, Wood CM. Gastrointestinal uptake and fate of cadmium in rainbow trout acclimated to sublethal dietary cadmium. Aquatic Toxicology. 2004;69(2): 149-163. doi: 10.1016/j.aquatox.2004.05.002.
64. Wagner GF, McKeown BA. Immunocytochemical localization of hormone-producing cells within the pancreatic islets of the rainbow trout (Salmo gairdneri). Cell and Tissue Research. 1981;221(1): 181-192. doi: 10.1007/bf00216580.
65. Hoar WS, Randall DJ. Fish Physiology Volume II: The Endocrine System. 1st ed. New York, London: Academic Press; 1969.

66. Capkin E, Birincioglu S, Altinok I. Histopathological changes in rainbow trout (Oncorhynchus mykiss) after exposure to sublethal composite nitrogen fertilizers. Ecotoxicology and Environmental Safety. 2009;72(7): 1999-2004. doi: 10.1016/j.ecoenv.2009.05.007.
67. Liu Q, Rise ML, Spitsbergen JM, Hori TS, Mieritz M, Geis S, et al. Gene expression and pathologic alterations in juvenile rainbow trout due to chronic dietary TCDD exposure. Aquatic Toxicology. 2013;140-141: 356-368. doi: 10.1016/j.aquatox.2013.06.018.
68. Helder T. Effects of 2,3,7,8-tetrachlorodibenzo-dioxin (TCDD) on early life stages of rainbow trout (Salmo gairdneri, Richardson). Toxicology. 1981;19(2): 101-112. doi: 10.1016/0300-483x(81)90092-5.
69. Conde-Sieira M, Patiño MAL, Míguez JM, Soengas JL. Glucosensing capacity in rainbow trout liver displays day-night variations possibly related to melatonin action. Journal of Experimental Biology. 2012;215(Pt 17): 3112-3119. doi: 10.1242/jeb.069740.
70. Nozaki M, Miyata K, Oota Y, Gorbman A, Plisetskaya EM. Colocalization of glucagon-like peptide and glucagon immunoreactivities in pancreatic islets and intestine of salmonids. Cell and Tissue Research. 1988;253(2): 371-375. doi: 10.1007/bf00222293.
71. Polakof S, Míguez JM, Moon TW, Soengas JL. Evidence for the presence of a glucosensor in hypothalamus, hindbrain, and Brockmann bodies of rainbow trout. American Journal of Physiology - Regulatory, Integrative and Comparative Physiology. 2007;292(4): R1657-1666. doi: 10.1152/ajpregu.00525.2006.
72. Boscher SK, McLoughlin M, Le Ven A, Cabon J, Baud M, Castric J. Experimental transmission of sleeping disease in one-year-old rainbow trout, Oncorhynchus mykiss (Walbaum), induced by sleeping disease virus. Journal of Fish Diseases. 2006;29(5): 263-273. doi: 10.1111/j.1365-2761.2006.00716.x.
73. Mulei IR, Nyaga PN, Mbuthia PG, Waruiru RM, Njagi LW, Mwihia EW, et al. Infectious pancreatic necrosis virus isolated from farmed rainbow trout and tilapia in Kenya is identical to European isolates. Journal of Fish Diseases. 2018;41(8): 1191-1200. doi: 10.1111/jfd.12807.
74. Taksdal T, Olsen AB, Bjerkås I, Hjortaas MJ, Dannevig BH, Graham DA, et al. Pancreas disease in farmed Atlantic salmon, Salmo salar L., and rainbow trout, Oncorhynchus mykiss (Walbaum), in Norway. Journal of Fish Diseases. 2007;30(9): 545-558. doi: 10.1111/j.1365-2761.2007.00845.x.
75. Fänge R. Physiology of the swimbladder. Physiological Reviews. 1966;46(2): 299-322. doi: 10.1152/physrev.1966.46.2.299.
76. Hendricks JD, Shelton DW, Loveland PM, Pereira CB, Bailey GS. Carcinogenicity of Dietary Dimethylnitrosomorpholine, N-methyl-N'-nitro-N-nitrosoguanidine, and Dibromoethane in Rainbow Trout. Toxicologic Pathology. 1995;23(4): 447-457. doi: 10.1177/019262339502300402.
77. Kiessling A, Storebakken T, Åsgård T, Kiessling KH. Changes in the structure and function of the epaxial muscle of rainbow trout (Oncorhynchus mykiss) in relation to ration and age: I. Growth dynamics. Aquaculture. 1991;93(4): 335-356. doi: 10.1016/0044-8486(91)90225-V.
78. Sheridan MA. Regulation of lipid metabolism in poikilothermic vertebrates. Comparative Biochemistry and Physiology Part B: Comparative Biochemistry. 1994;107(4): 495-508. doi: 10.1016/0305-0491(94)90176-7.
79. Kiessling A, Pickova J, Johansson L, Åsgård T, Storebakken T, Kiessling KH. Changes in fatty acid composition in muscle and adipose tissue of farmed rainbow trout (Oncorhynchus mykiss) in relation to ration and age. Food Chemistry. 2001;73(3): 271-284. doi: 10.1016/S0308-8146(00)00297-1.
80. Weil C, Lefèvre F, Bugeon J. Characteristics and metabolism of different adipose tissues in fish. Reviews in Fish Biology and Fisheries. 2013;23(2): 157-173. doi: 10.1007/s11160-012-9288-0.
81. Sheridan MA. Lipid dynamics in fish: aspects of absorption, transportation, deposition and mobilization. Comparative Biochemistry and Physiology Part B: Comparative Biochemistry. 1988;90(4): 679-690. doi: 10.1016/0305-0491(88)90322-7.
82. Jezierska B, Hazel JR, Gerking SD. Lipid mobilization during starvation in the rainbow trout, Salmo gairdneri Richardson, with attention to fatty acids. Journal of Fish Biology. 1982;21(6): 681-692. doi: 10.1111/j.1095-8649.1982.tb02872.x.
83. Kiessling A, Åsgård T, Storebakken T, Johansson L, Kiessling KH. Changes in the structure and function of the epaxial muscle of rainbow trout (Oncorhynchus mykiss) in relation to ration and age: III. Chemical composition. Aquaculture. 1991;93(4): 373-387. doi: 10.1016/0044-8486(91)90227-X.
84. Washburn BS, Frye DJ, Hung SSO, Doroshov SI, Conte FS. Dietary effects on tissue composition, oogenesis and the reproductive performance of female rainbow trout (Oncorhynchus mykiss). Aquaculture. 1990;90(2): 179-195. doi: 10.1016/0044-8486(90)90340-S.
85. Nanton DA, Vegusdal A, Rørå AMB, Ruyter B, Baeverfjord G, Torstensen BE. Muscle lipid storage pattern, composition, and adipocyte distribution in different parts of Atlantic salmon (Salmo

salar) fed fish oil and vegetable oil. Aquaculture. 2007;265(1-4): 230-243. doi: 10.1016/j.aquaculture.2006.03.053.
86. Shindo K, Tsuchiya T, Matsumoto JJ. Histological Study on White and Dark Muscles of Various Fishes. Nippon Suisan Gakkaishi. 1986;52(8): 1377-1399. doi: 10.2331/suisan.52.1377.
87. Pignatelli J, Castro R, González Granja A, Abós B, González L, Jensen LB, et al. Immunological Characterization of the Teleost Adipose Tissue and Its Modulation in Response to Viral Infection and Fat-Content in the Diet. PLoS ONE. 2014;9(10): e110920. doi: 10.1371/journal.pone.0110920.
88. Kleeman JM, Olson JR, Chen SM, Peterson RE. Metabolism and disposition of 2,3,7,8-tetrachlorodibenzo-p-dioxin in rainbow trout. Toxicology and Applied Pharmacology. 1986;83(3): 391-401. doi: 10.1016/0041-008X(86)90221-8.
89. Bachour G, Failing K, Georgii S, Elmadfa I, Brunn H. Species and Organ Dependence of PCB Contamination in Fish, Foxes, Roe Deer, and Humans. Archives of Environmental Contamination and Toxicology. 1998;35(4): 666-673. doi: 10.1007/s002449900429.
90. Cravedi JP, Tulliez J. Metabolism of n-alkanes and their incorporation into lipids in the rainbow trout. Environmental Research. 1986;39(1): 180-187. doi: 10.1016/s0013-9351(86)80020-2.
91. Parejko R, Johnston R, Keller R. Chlorohydrocarbons in Lake Superior Lake Trout (Salvelinus namaycush). Bulletin of Environmental Contamination and Toxicology. 1975;14(4): 480-488. doi: 10.1007/BF01705516.
92. Sparling J, Safe S. The effects of ortho chloro substituents on the retention of PCB isomers in rat, rabbit, Japanese quail, guinea pig and trout. Toxicology Letters. 1980;7(1): 23-28. doi: 10.1016/0378-4274(80)90080-6.
93. Babin PJ, Vernier JM. Plasma lipoproteins in fish. Journal of Lipid Research. 1989;30(4): 467-489.
94. Ferreira-Leach AMR, Hill EM. Bioconcentration and distribution of 4-tert-octylphenol residues in tissues of the rainbow trout (Oncorhynchus mykiss). Marine Environmental Research. 2001;51(1): 75-89. doi: 10.1016/s0141-1136(00)00256-7.
95. Pearson MP, Stevens ED. Size and hematological impact of the splenic erythrocyte reservoir in rainbow trout, Oncorhynchus mykiss. Fish Physiology and Biochemistry. 1991;9(1): 39-50. doi: 10.1007/bf01987610.
96. Pane EF, Richards JG, Wood CM. Acute waterborne nickel toxicity in the rainbow trout (Oncorhynchus mykiss) occurs by a respiratory rather than ionoregulatory mechanism. Aquatic Toxicology. 2003;63(1): 65-82. doi: 10.1016/S0166-445X(02)00131-5.
97. Roberts KS, Cryer A, Kay J, Solbe JF, Wharfe JR, Simpson WR. The effects of exposure to sub-lethal concentrations of cadmium on enzyme activities and accumulation of the metal in tissues and organs of rainbow and brown trout (Salmo gairdneri, Richardson and Salmo trutta Fario L.). Comparative Biochemistry and Physiology Part C: Comparative Pharmacology. 1979;62C(2): 135-140. doi: 10.1016/0306-4492(79)90001-7.
98. Spitsbergen JM, Schat KA, Kleeman JM, Peterson RE. Interactions of 2,3,7,8-tetrachlorodibenzo-p-dioxin (TCDD) with immune responses of rainbow trout. Veterinary Immunology and Immunopathology. 1986;12(1-4): 263-280. doi: 10.1016/0165-2427(86)90130-3.
99. Hoeger B, Koellner B, Kotterba G, van den Heuvel MR, Hitzfeld B, Dietrich DR. Influence of Chronic Exposure to Treated Sewage Effluent on the Distribution of White Blood Cell Populations in Rainbow Trout (Oncorhynchus mykiss) Spleen. Toxicological Sciences. 2004;82(1): 97-105. doi: 10.1093/toxsci/kfh250.
100. Walter GL, Jones PD, Giesy JP. Pathologic alterations in adult rainbow trout, Oncorhynchus mykiss, exposed to dietary 2,3,7,8-tetrachlorodibenzo-p-dioxin. Aquatic Toxicology. 2000;50(4): 287-299. doi: 10.1016/s0166-445x(00)00095-3.
101. Schwindt AR, Truelove N, Schreck CB, Fournie JW, Landers DH, Kent ML. Quantitative evaluation of macrophage aggregates in brook trout Salvelinus fontinalis and rainbow trout Oncorhynchus mykiss. Diseases of Aquatic Organisms. 2006;68(2): 101-113. doi: 10.3354/dao068101.
102. Wünnemann H, Weiß K, Arndt D, Baumann M, Weiß R, Ferling H, et al. Umweltqualitätsnormen für Binnengewässer: Überprüfung der Gefährlichkeit neuer bzw. prioritärer Substanzen. Dessau-Roßlau: Umweltbundesamt; 2020.
103. Billard R. Reproduction in rainbow trout: sex differentiation, dynamics of gametogenesis, biology and preservation of gametes. Aquaculture. 1992;100(1-3): 263-298. doi: 10.1016/0044-8486(92)90385-X.
104. Nævdal G, Lerøy R, Møller D. Variation in growth rate and age at first maturation in rainbow trout. FiskDir Skr Ser HavUnders. 1981;17: 71-78. doi: 11250/114814.
105. Uribe MC, Grier HJ, Mejía-Roa V. Comparative testicular structure and spermatogenesis in bony fishes. Spermatogenesis. 2014;4(3): e983400. doi: 10.4161/21565562.2014.983400.

106. Johnson R, Wolf J, Braunbeck T. OECD Guidance Document for the Diagnosis of Endocrine-Related Histopathology of Fish Gonads. Paris: Organization for Economic Co-operation and Development; 2009.
107. Schultz IR, Skillman A, Nicolas J-M, Cyr DG, Nagler JJ. Short-term exposure to 17α-ethynylestradiol decreases the fertility of sexually maturing male rainbow trout (Oncorhynchus mykiss). Environmental Toxicology and Chemistry. 2003;22(6): 1272-1280. doi: 10.1002/etc.5620220613.
108. Bjerregaard LB, Madsen AH, Korsgaard B, Bjerregaard P. Gonad histology and vitellogenin concentrations in brown trout (Salmo trutta) from Danish streams impacted by sewage effluent. Ecotoxicology. 2006;15(3): 315-327. doi: 10.1007/s10646-006-0061-9.
109. Le Gac F, Thomas JL, Mourot B, Loir M. In vivo and in vitro effects of prochloraz and nonylphenol ethoxylates on trout spermatogenesis. Aquatic Toxicology. 2001;53(3-4): 187-200. doi: 10.1016/S0166-445X(01)00165-5.
110. Chiang G, Barra R, Díaz-Jaramillo M, Rivas M, Bahamonde P, Munkittrick KR. Estrogenicity and intersex in juvenile rainbow trout (Oncorhynchus mykiss) exposed to Pine/Eucalyptus pulp and paper production effluent in Chile. Aquatic Toxicology. 2015;164: 126-134. doi: 10.1016/j.aquatox.2015.04.025.
111. Grier HJ, Uribe MC, Parenti LR. Germinal Epithelium, Folliculogenesis, and Postovulatory Follicles in Ovaries of Rainbow Trout, Oncorhynchus mykiss (Walbaum, 1792) (Teleostei, Protacanthopterygii, Salmoniformes). Journal of Morphology. 2007;268(4): 293-310. doi: 10.1002/jmor.10518.
112. van den Hurk R, Peute J. Cyclic Changes in the Ovary of the Rainbow Trout, Salmo gairdneri, with Special Reference to Sites of Steroidogenesis. Cell and Tissue Research. 1979;199(2): 289-306. doi: 10.1007/bf00236140.
113. van den Heuvel MR, Ellis RJ, Tremblay LA, Stuthridge TR. Exposure of Reproductively Maturing Rainbow Trout to a New Zealand Pulp and Paper Mill Effluent. Ecotoxicology and Environmental Safety. 2002;51(1): 65-75. doi: 10.1006/eesa.2001.2130.
114. Loncar J, Popović M, Zaja R, Smital T. Gene expression analysis of the ABC efflux transporters in rainbow trout (Oncorhynchus mykiss). Comparative Biochemistry and Physiology Part C: Toxicology & Pharmacology. 2010;151(2): 209-215. doi: 10.1016/j.cbpc.2009.10.009.
115. Carlson DB, Curtis LR, Williams DE. Salmonid sexual development is not consistently altered by embryonic exposure to endocrine-active chemicals. Environmental Health Perspectives. 2000;108(3): 249-255. doi: 10.1289/ehp.00108249.
116. Anderson BG, Loewen RD. Renal morphology of freshwater trout. American Journal of Anatomy. 1975;143(1): 93-114. doi: 10.1002/aja.1001430105.
117. Wilhelm S, Henneberg A, Köhler H-R, Rault M, Richter D, Scheurer M, et al. Does wastewater treatment plant upgrading with activated carbon result in an improvement of fish health? Aquatic Toxicology. 2017;192: 184-197. doi: 10.1016/j.aquatox.2017.09.017.
118. Butler AB, Hodos W. Comparative Vertebrate Neuroanatomy: Evolution and Adaptation. 2nd ed. Hoboken: John Wiley & Sons, Inc.; 2005.
119. Zhang L, Nawata CM, Wood CM. Sensitivity of ventilation and brain metabolism to ammonia exposure in rainbow trout, Oncorhynchus mykiss. Journal of Experimental Biology. 2013;216(Pt 21): 4025-4037. doi: 10.1242/jeb.087692.
120. Rademacher DJ, Steinpreis RE, Weber DN. Short-term exposure to dietary Pb and/or DMSA affects dopamine and dopamine metabolite levels in the medulla, optic tectum, and cerebellum of rainbow trout (Oncorhynchus mykiss). Pharmacology, Biochemistry and Behavior. 2001;70(2-3): 199-207. doi: 10.1016/s0091-3057(01)00597-4.
121. Melnyk-Lamont N, Best C, Gesto M, Vijayan MM. The Antidepressant Venlafaxine Disrupts Brain Monoamine Levels and Neuroendocrine Responses to Stress in Rainbow Trout. Environmental Science & Technology. 2014;48(22): 13434-13442. doi: 10.1021/es504331n.
122. Reverter M, Tapissier-Bontemps N, Lecchini D, Banaigs B, Sasal P. Biological and Ecological Roles of External Fish Mucus: A Review. Fishes. 2018;3(4): 41. doi: 10.3390/fishes3040041.
123. Whitear M. The skin surface of bony fishes. Journal of Zoology. 1970;160(4): 437-454. doi: 10.1111/j.1469-7998.1970.tb03091.x.
124. Pickering AD. Seasonal changes in the epidermis of the brown trout Salmo trutta (L.). Journal of Fish Biology. 1977;10(6): 561-566. doi: 10.1111/j.1095-8649.1977.tb04088.x.
125. Whitear M, Schliwa M. The skin of fishes including cyclostomes. In: Bereiter-Hahn J, Matoltsy AG, Richards KS, editors. Biology of the Integument - 2 Vertebrates. Berlin Heidelberg: Springer; 1986. pp. 8-77.
126. Handy RD, Eddy FB. Surface absorption of aluminium by gill tissue and body mucus of rainbow trout, Salmo gairdneri, at the onset of episodic exposure. Journal of Fish Biology. 1989;34(6): 865-874. doi: 10.1111/j.1095-8649.1989.tb03370.x.

127. Raj VS, Fournier G, Rakus K, Ronsmans M, Ouyang P, Michel B, et al. Skin mucus of Cyprinus carpio inhibits cyprinid herpesvirus 3 binding to epidermal cells. Veterinary Research. 2011;42(1): 92. doi: 10.1186/1297-9716-42-92.
128. Burkhardt-Holm P, Wahli T, Meier W. Nonylphenol Affects the Granulation Pattern of Epidermal Mucous Cells in Rainbow Trout, Oncorhynchus mykiss. Ecotoxicology and Environmental Safety. 2000;46(1): 34-40. doi: 10.1006/eesa.1999.1871.
129. Birzle CF. Etablierung und Validierung quantitativ-morphologischer Parameter bei Regenbogenforellen im Rahmen ökotoxikologischer Fragestellungen. Doctoral Thesis, Ludwig-Maximilians-Universität München. 2015. Available from: https://www.dr.hut-verlag.de/978-3-8439-2059-9.html.
130. McKim JM, Nichols JW, Lien GJ, Hoffman AD, Gallinat CA, Stokes GN. Dermal Absorption of Three Waterborne Chloroethanes in Rainbow Trout (Oncorhynchus mykiss) and Channel Catfish (Ictalurus punctatus). Fundamental and Applied Toxicology. 1996;31(2): 218-228. doi: 10.1006/faat.1996.0094.
131. Hogstrand C, Grosell M, Wood CM, Hansen H. Internal redistribution of radiolabelled silver among tissues of rainbow trout (Oncorhynchus mykiss) and European eel (Anguilla anguilla): the influence of silver speciation. Aquatic Toxicology. 2003;63(2): 139-157. doi: 10.1016/S0166-445X(02)00174-1.
132. Lennquist A, Mårtensson Lindblad LGE, Hedberg D, Kristiansson E, Förlin L. Colour and melanophore function in rainbow trout after long term exposure to the new antifoulant medetomidine. Chemosphere. 2010;80(9): 1050-1055. doi: 10.1016/j.chemosphere.2010.05.014.
133. Winterbottom R. A Descriptive Synonymy of the Striated Muscles of the Teleostei. Proceedings of the Academy of Natural Sciences of Philadelphia. 1973;125: 225-317.
134. Nag AC. Ultrastructure and adenosine triphosphatase activity of red and white muscle fibers of the caudal region of a fish, Salmo gairdneri. Journal of Cell Biology. 1972;55(1): 42-57. doi: 10.1083/jcb.55.1.42.
135. Johnston IA, Ward PS, Goldspink G. Studies on the swimming musculature of the rainbow trout I. Fibre types. Journal of Fish Biology. 1975;7(4): 451-458. doi: 10.1111/j.1095-8649.1975.tb04620.x.
136. Camusso M, Viganò L, Balestrini R. Bioconcentration of Trace Metals in Rainbow Trout: A Field Study. Ecotoxicology and Environmental Safety. 1995;31(2): 133-141. doi: 10.1006/eesa.1995.1053.
137. Kierkegaard A, Chen C, Armitage JM, Arnot JA, Droge S, McLachlan MS. Tissue Distribution of Several Series of Cationic Surfactants in Rainbow Trout (Oncorhynchus mykiss) Following Exposure via Water. Environmental Science & Technology. 2020;54(7): 4190-4199. doi: 10.1021/acs.est.9b07600.
138. Boyle D, Sutton PA, Handy RD, Henry TB. Intravenous injection of unfunctionalized carbon-based nanomaterials confirms the minimal toxicity observed in aqueous and dietary exposures in juvenile rainbow trout (Oncorhynchus mykiss). Environmental Pollution. 2018;232: 191-199. doi: 10.1016/j.envpol.2017.09.033.
139. Prindiville JS, Mennigen JA, Zamora JM, Moon TW, Weber JM. The fibrate drug gemfibrozil disrupts lipoprotein metabolism in rainbow trout. Toxicology and Applied Pharmacology. 2011;251(3): 201-208. doi: 10.1016/j.taap.2010.12.013.
140. Lefèvre F, Aubin J, Louis W, Labbé L, Bugeon J. Moderate hypoxia or hyperoxia affect fillet yield and the proportion of red muscle in rainbow trout. Cybium. 2007;31(2): 237-243. doi: 10.26028/cybium/2007-312-018.
141. Kent GC, Carr RC. Comparative Anatomy of the Vertebrates. 9th ed. Boston: McGraw Hill; 2001.
142. Linzey D. Vertebrate Biology. 3rd ed. Baltimore: Johns Hopkins University Press; 2020.
143. Witten PE, Huysseune A, Hall BK. A practical approach for the identification of the many cartilaginous tissues in teleost fish. Journal of Applied Ichthyology. 2010;26(2): 257-262. doi: 10.1111/j.1439-0426.2010.01416.x.
144. Benjamin M. The cranial cartilages of teleosts and their classification. Journal of Anatomy. 1990;169: 153-172.
145. Benjamin M, Ralphs JR, Eberewariye OS. Cartilage and related tissues in the trunk and fins of teleosts. Journal of Anatomy. 1992;181(Pt 1): 113-118.
146. Benjamin M, Ralphs JR. Extracellular matrix of connective tissues in the heads of teleosts. Journal of Anatomy. 1991;179: 137-148.
147. Benjamin M. The development of hyaline-cell cartilage in the head of the black molly, Poecilia sphenops. Evidence for secondary cartilage in a teleost. Journal of Anatomy. 1989;164: 145-154.
148. Miller JR, Anderson JB, Lechler PJ, Kondrad SL, Galbreath PF, Salter EB. Influence of temporal variations in water chemistry on the Pb isotopic composition of rainbow trout (Oncorhynchus mykiss). Science of the Total Environment. 2005;350(1-3): 204-224. doi: 10.1016/j.scitotenv.2005.01.030.

149. Goeritz I, Falk S, Stahl T, Schäfers C, Schlechtriem C. Biomagnification and tissue distribution of perfluoroalkyl substances (PFASs) in market-size rainbow trout (Oncorhynchus mykiss). Environmental Toxicology and Chemistry. 2013;32(9): 2078-2088. doi: 10.1002/etc.2279.
150. Kamunde C, MacPhail R. Metal-metal interactions of dietary cadmium, copper and zinc in rainbow trout, Oncorhynchus mykiss. Ecotoxicology and Environmental Safety. 2011;74(4): 658-667. doi: 10.1016/j.ecoenv.2010.10.016.
151. Standen EM. Muscle activity and hydrodynamic function of pelvic fins in trout (Oncorhynchus mykiss). Journal of Experimental Biology. 2010;213(5): 831-841. doi: 10.1242/jeb.033084.
152. Buckland-Nicks JA, Gillis M, Reimchen TE. Neural network detected in a presumed vestigial trait: ultrastructure of the salmonid adipose fin. Proceedings of the Royal Society - Biological Sciences. 2012;279(1728): 553-563. doi: 10.1098/rspb.2011.1009.
153. Koll R, Martorell Ribera J, Brunner RM, Rebl A, Goldammer T. Gene Profiling in the Adipose Fin of Salmonid Fishes Supports its Function as a Flow Sensor. Genes. 2019;11(1): 21. doi: 10.3390/genes11010021.
154. Beacham TD, Murray CB. Sexual Dimorphism in the Adipose Fin of Pacific Salmon (Oncorhynchus). Canadian Journal of Fisheries and Aquatic Sciences. 1983;40(11): 2019-2024. doi: 10.1139/f83-231.
155. Westley PAH, Carlson SM, Quinn TP. Among-population variation in adipose fin size parallels the expression of other secondary sexual characteristics in sockeye salmon (Oncorhynchus nerka). Environmental Biology of Fishes. 2008;81(4): 439-446. doi: 10.1007/s10641-007-9236-3.
156. McNeill SA, Arens CJ, Hogan NS, Köllner B, van den Heuvel MR. Immunological impacts of oil sands-affected waters on rainbow trout evaluated using an in situ exposure. Ecotoxicology and Environmental Safety. 2012;84: 254-261. doi: 10.1016/j.ecoenv.2012.07.016.
157. Adam C, Garnier-Laplace J, Baudin JP. Uptake from water, release and tissue distribution of ^{54}Mn in the Rainbow trout (Oncorhynchus mikiss Walbaum). Environmental Pollution. 1997;97(1-2): 29-38. doi: 10.1016/S0269-7491(97)00083-3.
158. Greani S, Lourkisti R, Berti L, Marchand B, Giannettini J, Santini J, et al. Effect of chronic arsenic exposure under environmental conditions on bioaccumulation, oxidative stress, and antioxidant enzymatic defenses in wild trout Salmo trutta (Pisces, Teleostei). Ecotoxicology. 2017;26(7): 930-941. doi: 10.1007/s10646-017-1822-3.
159. Ortiz JA, Rueda A, Carbonell G, Camargo JA, Nieto F, Reoyo MJ, et al. Acute toxicity of sulfide and lower pH in cultured rainbow trout, Atlantic salmon, and coho salmon. Bulletin of Environmental Contamination and Toxicology. 1993;50(1): 164-170. doi: 10.1007/bf00196556.
160. Hoar WS, Randall DJ. Fish Physiology Volume X: Gills - Part B: Ion and Water Transfer. 1st ed. Orlando: Academic Press; 1984.
161. Hyrtl J. Beobachtungen aus dem Gebiete der vergleichenden Gefässlehre. 2. Über den Bau der Kiemen der Fische. Medizinisches Jahrbuch. 1938;15: 232-248.
162. Mölich A, Waser W, Heisler N. The teleost pseudobranch: a role for preconditioning of ocular blood supply? Fish Physiology and Biochemistry. 2009;35(2): 273-286. doi: 10.1007/s10695-008-9207-4.
163. Fischer-Scherl T, Veeser A, Hoffmann RW, Kühnhauser C, Negele R-D, Ewringmann T. Morphological effects of acute and chronic atrazine exposure in rainbow trout (Oncorhynchus mykiss). Archives of Environmental Contamination and Toxicology. 1991;20(4): 454-461. doi: 10.1007/bf01065833.
164. Schwaiger J, Bucher F, Ferling H, Kalbfus W, Negele R-D. A prolonged toxicity study on the effects of sublethal concentrations of bis(tri-n-butyltin)oxide (TBTO): histopathological and histochemical findings in rainbow trout (Oncorhynchus mykiss). Aquatic Toxicology. 1992;23(1): 31-48. doi: 10.1016/0166-445X(92)90010-K.
165. Carvalho PSM, Tillitt DE. 2,3,7,8-TCDD effects on visual structure and function in swim-up rainbow trout. Environmental Science & Technology. 2004;38(23): 6300-6306. doi: 10.1021/es034857i.
166. Hamdani el H, Døving KB. The functional organization of the fish olfactory system. Progress in Neurobiology. 2007;82(2): 80-86. doi: 10.1016/j.pneurobio.2007.02.007.
167. Bett NN, Hinch SG. Olfactory navigation during spawning migrations: a review and introduction of the Hierarchical Navigation Hypothesis. Biological reviews of the Cambridge Philosophical Society. 2016;91(3): 728-759. doi: 10.1111/brv.12191.
168. Espinoza HM, Williams CR, Gallagher EP. Effect of cadmium on glutathione S-transferase and metallothionein gene expression in coho salmon liver, gill and olfactory tissues. Aquatic Toxicology. 2012;110-111: 37-44. doi: 10.1016/j.aquatox.2011.12.012.
169. Bazáes A, Schmachtenberg O. Odorant tuning of olfactory crypt cells from juvenile and adult rainbow trout. Journal of Experimental Biology. 2012;215(Pt 10): 1740-1748. doi: 10.1242/jeb.067264.

170. Zielinski B, Hara TJ. Morphological and Physiological Development of Olfactory Receptor Cells in Rainbow Trout (Salmo gairdneri) Embryos. The Journal of Comparative Neurology. 1988;271(2): 300-311. doi: 10.1002/cne.902710210.
171. Lavado R, Rimoldi JM, Schlenk D. Mechanisms of fenthion activation in rainbow trout (Oncorhynchus mykiss) acclimated to hypersaline environments. Toxicology and Applied Pharmacology. 2009;235(2): 143-152. doi: 10.1016/j.taap.2008.11.017.
172. Lavado R, Aparicio-Fabre R, Schlenk D. Effects of salinity acclimation on the pesticide-metabolizing enzyme flavin-containing monooxygenase (FMO) in rainbow trout (Oncorhynchus mykiss). Comparative Biochemistry and Physiology Part C: Toxicology & Pharmacology. 2013;157(1): 9-15. doi: 10.1016/j.cbpc.2012.08.004.
173. Monod G, Saucier D, Perdu-Durand E, Diallo M, Cravedi JP, Astic L. Biotransformation enzyme activities in the olfactory organ of rainbow trout (Oncorhynchus mykiss). Immunocytochemical localization of cytochrome P4501A1 and its induction by β-naphthoflavone. Fish Physiology and Biochemistry. 1994;13(6): 433-444. doi: 10.1007/bf00004326.
174. Tierney KB, Sampson JL, Ross PS, Sekela MA, Kennedy CJ. Salmon Olfaction is Impaired by an Environmentally Realistic Pesticide Mixture. Environmental Science & Technology. 2008;42(13): 4996-5001. doi: 10.1021/es800240u.
175. Tierney KB, Baldwin DH, Hara TJ, Ross PS, Scholz NL, Kennedy CJ. Olfactory toxicity in fishes. Aquatic Toxicology. 2010;96(1): 2-26. doi: 10.1016/j.aquatox.2009.09.019.
176. Lewis ER, Leverenz EL, Bialek WS. The Vertebrate Inner Ear. 1st ed. Boca Raton: CRC Press Inc; 1985.
177. Lu Z. Neural Mechanisms of Hearing in Fishes. In: von der Emde G, Mogdans J, Kapoor BG, editors. The Senses of Fish - Adaptations for the Reception of Natural Stimuli. New Delhi: Springer; 2004. pp. 147-172.
178. Hoar WS, Randall DJ. Fish Physiology Volume V: Sensory Systems and Electric Organs. 1st ed. New York, London: Academic Press; 1971.
179. Popper AN. Comparative Scanning Electron Microscopic Investigations of the Sensory Epithelia in the Teleost Sacculus and Lagena. The Journal of Comparative Neurology. 1981;200(3): 357-374. doi: 10.1002/cne.902000306.
180. Campana SE. Chemistry and composition of fish otoliths: pathways, mechanisms and applications. Marine Ecology Progress Series. 1999;188: 263-297. doi: 10.3354/meps188263.
181. Fay RR, Popper AN. Comparative Hearing: Fish and Amphibians. 1st ed. New York: Springer; 1999.
182. Friedrich LA, Orr PL, Halden NM, Yang P, Palace VP. Exposure histories derived from selenium in otoliths of three cold-water fish species captured downstream from coal mining activity. Aquatic Toxicology. 2011;105(3-4): 492-496. doi: 10.1016/j.aquatox.2011.07.021.
183. Keller DH, Zelanko PM, Gagnon JE, Horwitz RJ, Galbraith HS, Velinsky DJ. Linking otolith microchemistry and surface water contamination from natural gas mining. Environmental Pollution. 2018;240: 457-465. doi: 10.1016/j.envpol.2018.04.026.
184. Palace VP, Halden NM, Yang P, Evans RE, Sterling G. Determining Residence Patterns of Rainbow Trout Using Laser Ablation Inductively Coupled Plasma Mass Spectrometry (LA-ICP-MS) Analysis of Selenium in Otoliths. Environmental Science & Technology. 2007;41(10): 3679-3683. doi: 10.1021/es0628093.
185. Reash RJ, Friedrich LA, Bock MJ, Halden NM, Palace VP. Selenium and Mercury in Freshwater Fish Muscle Tissue and Otoliths: A Comparative Analysis. Environmental Toxicology and Chemistry. 2019;38(7): 1467-1475. doi: 10.1002/etc.4432.
186. Smith ME, Monroe JD. Causes and Consequences of Sensory Hair Cell Damage and Recovery in Fishes. In: Sisneros J, editor. Fish Hearing and Bioacoustics Advances in Experimental Medicine and Biology, vol 877. Cham: Springer; 2016. pp. 393-417.
187. Montgomery JC, Baker CF, Carton AG. The lateral line can mediate rheotaxis in fish. Nature. 1997;389: 960-963. doi: 10.1038/40135.
188. Montgomery JC, McDonald F, Baker CF, Carton AG, Ling N. Sensory integration in the hydrodynamic world of rainbow trout. Proceedings of the Royal Society - Biological Sciences. 2003;270(Suppl 2): 195-197. doi: 10.1098/rsbl.2003.0052.
189. Münz H. Functional organization of the lateral line periphery. In: Coombs S, Görner P, Münz H, editors. The Mechanosensory Lateral Line: Neurobiology and Evolution. New York: Springer; 1989. pp. 285-297.
190. Montgomery J, Coombs S, Halstead M. Biology of the mechanosensory lateral line in fishes. Reviews in Fish Biology and Fisheries. 1995;5(4): 399-416.

191. Partridge BL, Pitcher TJ. The Sensory Basis of Fish Schools: Relative Roles of Lateral Line and Vision. Journal of Comparative Physiology A - Neuroethology, Sensory, Neural, and Behavioral Physiology. 1980;135(4): 315-325. doi: 10.1007/BF00657647.
192. Satou M, Takeuchi H-A, Nishii J, Tanabe M, Kitamura S, Okumoto N, et al. Behavioral and electrophysiological evidences that the lateral line is involved in the inter-sexual vibrational communication of the himé salmon (landlocked red salmon, Oncorhynchus nerka). Journal of Comparative Physiology A - Neuroethology, Sensory, Neural, and Behavioral Physiology. 1994;174(5): 539-549. doi: 10.1007/BF00217373.
193. Satou M, Takeuchi H-A, Takei K, Hasegawa T, Matsushima T, Okumoto N. Characterization of vibrational and visual signals which elicit spawning behavior in the male himé salmon (landlocked red salmon, Oncorhynchus nerka). Journal of Comparative Physiology A - Neuroethology, Sensory, Neural, and Behavioral Physiology. 1994;174(5): 527-537. doi: 10.1007/BF00217372.
194. Weber DD, Schiewe MH. Morphology and function of the lateral line of juvenile steelhead trout in relation to gas-bubble disease. Journal of Fish Biology. 1976;9(3): 217-233. doi: 10.1111/j.1095-8649.1976.tb04675.x.
195. Engelmann J, Hanke W, Bleckmann H. Lateral line reception in still- and running water. Journal of Comparative Physiology A: Neuroethology, Sensory, Neural, and Behavioral Physiology. 2002;188(7): 513-526. doi: 10.1007/s00359-002-0326-6.
196. Jørgensen JM. Evolution of Octavolateralis Sensory Cells. In: Coombs S, Görner P, Münz H, editors. The Mechanosensory Lateral line: Neurobiology and Evolution. New York: Springer; 1989. pp. 115-145.
197. Siregar YI. Morphology and Growth of Lateral Line Organs of the Rainbow Trout (Oncorhynchus mykiss). Acta Zoologica. 1994;75(3): 213-218. doi: 10.1111/j.1463-6395.1994.tb01209.x.
198. Faucher K, Fichet D, Miramand P, Lagardère JP. Impact of acute cadmium exposure on the trunk lateral line neuromasts and consequences on the “C-start” response behaviour of the sea bass (Dicentrarchus labrax L.; Teleostei, Moronidae). Aquatic Toxicology. 2006;76(3-4): 278-294. doi: 10.1016/j.aquatox.2005.10.004.
199. Linbo TL, Baldwin DH, McIntyre JK, Scholz NL. Effects of water hardness, alkalinity, and dissolved organic carbon on the toxicity of copper to the lateral line of developing fish. Environmental Toxicology and Chemistry. 2009;28(7): 1455-1461. doi: 10.1897/08-283.1.
200. Osborne OJ, Mukaigasa K, Nakajima H, Stolpe B, Romer I, Philips U, et al. Sensory systems and ionocytes are targets for silver nanoparticle effects in fish. Nanotoxicology. 2016;10(9): 1276-1286. doi: 10.1080/17435390.2016.1206147.
201. Faucher K, Fichet D, Miramand P, Lagardère J-P. Impact of chronic cadmium exposure at environmental dose on escape behaviour in sea bass (Dicentrarchus labrax L.; Teleostei, Moronidae). Environmental Pollution. 2008;151(1): 148-157. doi: 10.1016/j.envpol.2007.02.017.
202. Song J, Yan HY, Popper AN. Damage and recovery of hair cells in fish canal (but not superficial) neuromasts after gentamicin exposure. Hearing Research. 1995;91(1-2): 63-71. doi: 10.1016/0378-5955(95)00170-0.
203. Williams DL. Ophthalmology of Exotic Pets. 1st ed. Oxford: John Wiley & Sons Ltd; 2012.
204. Duke-Elder S. System of Ophthalmology. 2nd ed. St. Louis: The C. V. Mosby Company; 1958.
205. Gelatt KN, Gilger BC, Kern TJ. Veterinary Ophthalmology: Two Volume Set. 5th ed. Hoboken: John Wiley & Sons Inc.; 2013.
206. Reimann AS. Anatomisch-makroskopische Untersuchungen von Fischaugen: Eine interaktive Anleitung zur Herstellung und Fotografie von ophthalmologischen Präparaten als Grundlage zur Fischophthalmologie. Doctoral Thesis, Ludwig-Maximilians-Unversität München. 2015. Available from: https://edoc.ub.uni-muenchen.de/18041/.
207. Negishi K, Laufer M, Drujan BD. Drug-Induced Changes in Catecholaminergic Cells of the Fish Retina. Journal of Neuroscience Research. 1980;5(6): 599-609. doi: 10.1002/jnr.490050614.
208. Correia AT, Rebelo D, Marques J, Nunes B. Effects of the chronic exposure to cerium dioxide nanoparticles in Oncorhynchus mykiss: Assessment of oxidative stress, neurotoxicity and histological alterations. Environmental Toxicology and Pharmacology. 2019;68: 27-36. doi: 10.1016/j.etap.2019.02.012.
209. Rodrigues S, Antunes SC, Correia AT, Nunes B. Toxicity of erythromycin to Oncorhynchus mykiss at different biochemical levels: detoxification metabolism, energetic balance, and neurological impairment. Environmental Science and Pollution Research. 2019;26(1): 227-239. doi: 10.1007/s11356-018-3494-9.
210. Larsson P, Ngethe S, Ingebrigtsen K, Tjälve H. Extrahepatic Disposition of ^{3}H-aflatoxin B_1 in the Rainbow Trout (Oncorhynchus mykiss). Pharmacology & Toxicology. 1992;71(4): 262-271. doi: 10.1111/j.1600-0773.1992.tb00981.x.

211. Laycock NLC, Schirmer K, Bols NC, Sivak JG. Optical properties of rainbow trout lenses after in vitro exposure to polycyclic aromatic hydrocarbons in the presence or absence of ultraviolet radiation. Experimental Eye Research. 2000;70(2): 205-214. doi: 10.1006/exer.1999.0774.
212. Clotfelter ED, Bell AM, Levering KR. The role of animal behaviour in the study of endocrine-disrupting chemicals. Animal Behaviour. 2004;68(4): 665-676. doi: 10.1016/j.anbehav.2004.05.004.
213. Walker CH. Organic Pollutants - An ecotoxicological perspective. 2nd ed. Boca Raton: CRC Press Taylor & Francis Group; 2009.
214. Browne P, Van Der Wal L, Gourmelon A. OECD approaches and considerations for regulatory evaluation of endocrine disruptors. Molecular and Cellular Endocrinology. 2020;504: 110675. doi: 10.1016/j.mce.2019.110675.
215. Andersson T, Förlin L, Olsen S, Fostier A, Breton B. Pituitary as a target organ for toxic effects of P4501A1 inducing chemicals. Molecular and Cellular Endocrinology. 1993;91(1-2): 99-105. doi: 10.1016/0303-7207(93)90260-Q.
216. Gesto M, Tintos A, Soengas JL, Míguez JM. Effects of acute and prolonged naphthalene exposure on brain monoaminergic neurotransmitters in rainbow trout (Oncorhynchus mykiss). Comparative Biochemistry and Physiology Part C: Toxicology & Pharmacology. 2006;144(2): 173-183. doi: 10.1016/j.cbpc.2006.08.002.
217. Gesto M, Tintos A, Rodríguez-Illamola A, Soengas JL, Míguez JM. Effects of naphthalene, beta-naphthoflavone and benzo(a)pyrene on the diurnal and nocturnal indoleamine metabolism and melatonin content in the pineal organ of rainbow trout, Oncorhynchus mykiss. Aquatic Toxicology. 2009;92(1): 1-8. doi: 10.1016/j.aquatox.2008.12.008.
218. Palace VP, Pleskach K, Halldorson T, Danell R, Wautier K, Evans B, et al. Biotransformation Enzymes and Thyroid Axis Disruption in Juvenile Rainbow Trout (Oncorhynchus mykiss) Exposed to Hexabromocyclododecane Diastereoisomers. Environmental Science & Technology. 2008;42(6): 1967-1972. doi: 10.1021/es702565h.
219. Palace VP, Park B, Pleskach K, Gemmill B, Tomy G. Altered thyroxine metabolism in rainbow trout (Oncorhynchus mykiss) exposed to hexabromocyclododecane (HBCD). Chemosphere. 2010;80(2): 165-169. doi: 10.1016/j.chemosphere.2010.03.016.
220. Bern HA, Madsen SS. A selective survey of the endocrine system of the rainbow trout (Oncorhynchus mykiss) with emphasis on the hormonal regulation of ion balance. Aquaculture. 1992;100(1-3): 237-262. doi: 10.1016/0044-8486(92)90384-W.
221. Kakizawa S, Kaneko T, Hasegawa S, Hirano T. Activation of Somatolactin Cells in the Pituitary of the Rainbow Trout Oncorhynchus mykiss by Low Environmental Calcium. General and Comparative Endocrinology. 1993;91(3): 298-306. doi: 10.1006/gcen.1993.1130.
222. Ekström P, Meissl H. The pineal organ of teleost fishes. Reviews in Fish Biology and Fisheries. 1997;7(2): 199-284. doi: 10.1023/A:1018483627058.
223. Krawarik F. Über eine bisher unbekannte Drüse ohne Ausführungsgang bei den heimischen Knochenfischen. Zeitschrift für mikroskopisch-anatomische Forschung. 1936;39: 555-609.
224. Robertson DR. Some morphological observations of the ultimobranchial gland in the rainbow trout, Salmo gairdneri. Journal of Anatomy. 1969;105(Pt 1): 115-127.
225. McMillan PJ, Hooker WM, Rods BA, Deftos LJ. Ultimobranchial gland of the trout (Salmo gairdneri) I. Immunohistology and radioimmunoassay of calcitonin. General and Comparative Endocrinology. 1976;28(3): 313-319. doi: 10.1016/0016-6480(76)90183-0.
